# Structure-Guided Design of a KMT9 Inhibitor Prodrug with Cellular Activity

**DOI:** 10.1021/acs.jmedchem.4c02953

**Published:** 2025-06-17

**Authors:** Sheng Wang, Nicolas P. F. Barthes, Sylvia Urban, Viktor I. Hazai, Sebastian O. Klein, Tabea Pappert, Paul Kümmel, Nicolas Heller, Johannes Bacher, Maximilian Staudt, Jan Ruprecht, Ling Peng, Manuela Sum, Christopher Berlin, Daad Sarraf, Pierre Regenass, Robin Warstat, Johannes Walz, Pankaj Mishra, Lin Zhang, Oliver Einsle, Stefan Günther, Bernhard Breit, Eric Metzger, Manfred Jung, Roland Schüle

**Affiliations:** † Klinik für Urologie und Zentrale Klinische Forschung, 14879 Klinikum der Albert-Ludwigs-Universität Freiburg, Freiburg 79106, Germany; ‡ Institute of Pharmaceutical Sciences, 9174 Albert-Ludwigs-Universität Freiburg, Freiburg 79104, Germany; § Institute of Organic Chemistry, 9174 Albert-Ludwigs-Universität Freiburg, Freiburg 79104, Germany; ∥ Institut für Biochemie, 9174 Albert-Ludwigs-Universität Freiburg, Freiburg 79104, Germany; ⊥ German Cancer Consortium (DKTK), Partner site Freiburg, A partnership between DKFZ and Medical Center - University of Freiburg, Freiburg 79106, Germany; # CIBSS Centre of Biological Signalling Studies, University of Freiburg, Freiburg 79106, Germany

## Abstract

Lysine methyltransferase 9 (KMT9), an obligate heterodimer (KMT9α/KMT9β), belongs to the few described Rossmann-fold histone lysine methyltransferases and monomethylates histone H4 at lysine 12 (H4K12me1). KMT9 depletion or inhibition impairs the proliferation of tumors, including prostate, lung, colon, and bladder cancer cells, underscoring its therapeutic potential. Here, we show the development of branched cofactor analogues with a methionine side chain as highly potent KMT9 inhibitors. Through structure-guided design, a basic nitrogen and 4-chlorophenoxy-2-fluorobenzene in the substrate branch contribute most to the high potency and selectivity. Due to the zwitterionic methionine side chain, the inhibitors did not show cellular activity. Importantly, an ethyl ester prodrug **8** exhibits cellular target engagement and effectively blocks the proliferation of colon cancer cell lines, further validating pharmacological inhibition of KMT9 as a promising strategy for cancer therapy.

## Graphical abstract

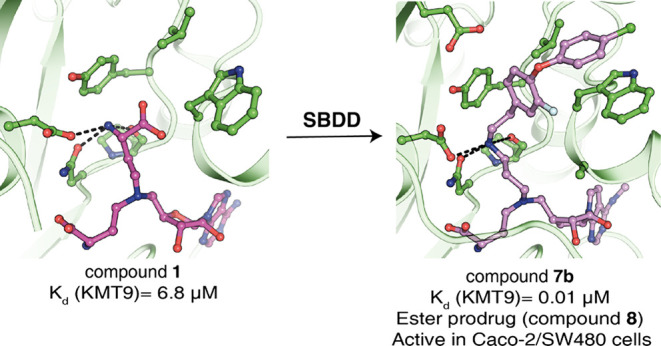

## Introduction

Due to the critical role in gene expression of different cancers, protein methyltransferases (PMTs) have been widely investigated as therapeutic targets.^1–5^ PMTs mainly adopt SET domain or Rossmann-fold as the catalytic domain to methylate different targets.^6,7^

Recently, we identified KMT9 as a histone methyltransferase monomethylating histone H4K12^8^. KMT9 acts as an obligate heterodimer composed of KMT9α and KMT9β, in which KMT9α is the enzymatic subunit containing a typical Rossmann-fold SAM binding pocket and KMT9β functions as a chaperon protein activating the enzymatic activity of KMT9α. In addition to histone lysine methylation, KMT9 was reported to act as a glutamine methyltransferase^9–11^ or an adenine-N6 DNA methyltransferase.^12–15^

KMT9 regulates growth of several types of tumor cells including prostate, lung, and colon cancer cell lines.^8,16,17^ Knocking-down of KMT9 inhibits growth of androgen receptor-dependent as well as castration- and enzalutamide-resistant prostate cancer cells by regulating the expression of cell cycle genes and inducing apoptosis.^8^ As comparison, KMT9 depletion blocks the proliferation of small cell and nonsmall cell lung cancer cells via a nonapoptotic cell death.^16^ In addition, it showed that KMT9 loss impaired maintenance and function of colorectal cancer stem cells.^17^ Furthermore, higher expression of KMT9 was reported to be associated with aggressive basal-like muscle-invasive bladder cancer (MIBC).^18^ Notably, it showed that the antiproliferative effects of KMT9 depletion is dependent on the enzymatic activity of KMT9^8^. Thus, inhibiting the enzymatic activity of KMT9 may offer a potential therapeutic strategy to block KMT9-dependent cancer cell proliferation.

Previously, a N-terminal methyltransferase inhibitor (NAH-C3-GPKK) was reported to bind to KMT9 as well with moderate affinity through a chemoproteomics study.^19^ NAH-C3-GPKK is a bisubstrate peptide inhibitor containing a SAM and GPKK peptide moiety, which has only been tested in vitro. Given its suboptimal physicochemical properties, NAH-C3-GPKK is unlikely to enter cells. Recently, a bisubstrate inhibitor (**KMI169,** Figure S1A) was shown by us to specifically block KMT9 enzymatic activity with cellular activity, providing a proof of concept that pharmacologically targeting KMT9 blocks cancer cell proliferation.^20^ So far, no other SAR studies have been published on KMT9. Here, we report the development of a potent and selective KMT9 inhibitor (compound **8**, Figure 6A) with prodrug properties following a structure guided drug design. Compound **7b** (parental compound of compound **8**, Table 6) shows high specificity for KMT9 with a dissociation constant (K_d_) of 10 nM. Importantly, compound **7b** after release from prodrug compound **8** exhibits cellular target engagement and reduces cellular levels of histone H4K12 monomethylation. Consequently, compound **8** specifically blocks proliferation of colon cancer cells whereas a structurally related control compound, compound **8-N** (Figure 6C), fails to do so, which enables compound **8** as a tool compound to investigate the function of KMT9 in cells. Additionally, the structural information on various ligand-bound KMT9 structures together with the structure activity (SAR) data could provide insights into developing more potent KMT9 inhibitors with improved physico-chemical properties.

## Result

### Identification of a Bisubstrate Hit for KMT9

To initiate the development of potent and selective KMT9 inhibitors, we first designed a bisubstrate compound based on our previously reported crystal structure of KMT9 in complex with S-adenosyl-l-homocysteine (SAH) and histone H4 peptide monomethylated at lysine 12 (H4K12me1) [PDB: 6H1E].^8^ Compound **1** consists of an adenosine scaffold and two amino acid branches designed to bind into the substrate channel and the methionine pocket of KMT9 (Figure 1A). Using Microscale Thermophoresis (MST), we determined a dissociation constant (K_d_) of 6.8 μM for KMT9 binding of compound **1** (Figure 1B). This K_d_ is comparable to the one (K_d_ = 10.9 μM) observed for SAH.^20^ To gain structural insights into ligand binding, we solved cocrystal structures of KMT9 with compound **1** or SAH.

**Figure 1:**
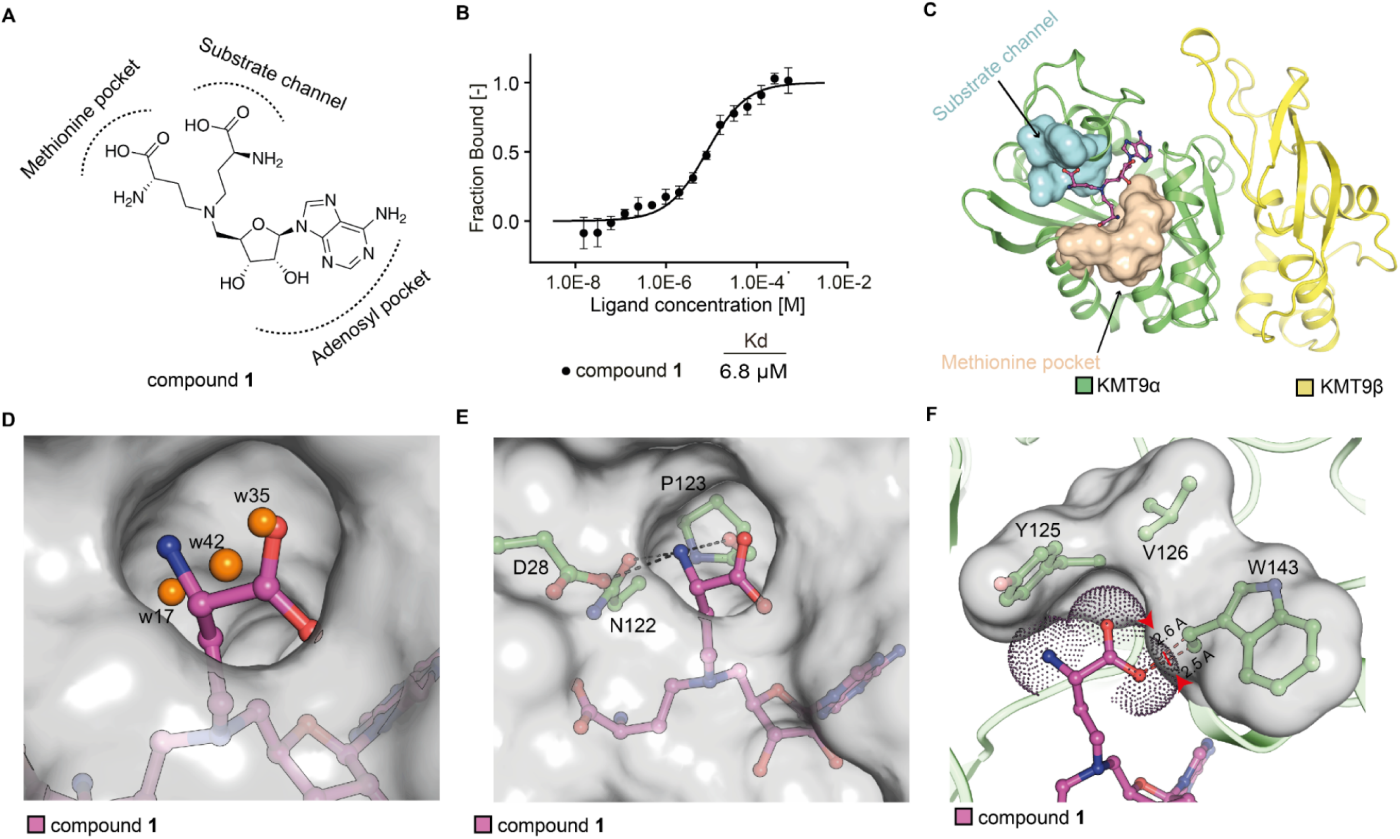
Identification of a hit compound of KMT9. (A) Chemical structure of compound **1** with predicted binding mode. (B) MST assay to determine the dissociation constant (K_d_) of compound **1** binding to KMT9. Data represent means ± s.d (*n* = 3). (C) Overall structure of the KMT9/compound **1** complex (PDB code: 9FIM). KMT9α (green) and KMT9β (yellow) proteins are represented as ribbons. Arrows indicate the substrate channel (cyan) and the methionine pocket (brown) illustrated by surface view. Compound **1** is shown as sticks (magenta). (D) Superimposition of compound **1**/KMT9 and SAH/KMT9 complex structures. Compound **1** (magenta) and SAH (blue) are shown by sticks. Water molecules are shown by spheres (orange). (E) Hydrogen bonds between the amino group of compound **1** (magenta) and KMT9α (green) in the substrate branch. (F) Unfavored contacts between the carbonyl group of compound **1** (magenta) and W142 of KMT9α. Key residues and ligands are depicted as sticks. Water molecules are shown as orange spheres. Contacts are represented by black dashed lines. Van-der-Waals radius of compound **1** are shown as dots. Substate channel is shown as surface. KMT9α is shown as ribbon.

Superimposition of the new and our previously reported KMT9/ligand structures^8^ showed identical binding modes for the SAH backbones of the ligands (Figure S1B), which were well-defined by electron density (Figure S1C,D). As expected, the two amino acid branches of compound **1** occupied the methionine pocket and the H4K12me1 substrate channel (Figure 1C). In the substrate channel, three water molecules observed in the KMT9/SAH complex were replaced by the amino acid branch of compound **1** (Figures 1D and S1E). Furthermore, the amine group of the substrate channel branch formed hydrogen bonds with the side chains of aspartate (D)­28 and asparagine (N)­122 as well as the main chain carbonyl of proline (P)­123 (Figure 1E). For the carboxylate moiety of the substrate branch, we noted a close, potentially unfavorable contact with the side chain of tryptophan (W)­143 of KMT9α (Figure 1F), which might counteract positive binding contributions of the amine moiety. Thus, in the first set of experiments, we identified the bisubstrate inhibitor compound **1** as a hit binding to KMT9 with micromolar affinity.

### Structure–Activity Relationship of Compound **1** Derivatives

To improve the potency of the hit, we focused the structure–activity relationship (SAR) analysis on the substrate channel where the carboxylate of compound **1** was suspiciously too close to the side chain of W142 (Figure 1F). Consequently, we replaced the carboxylate moiety with a series of substituents (Table 1), which were smaller, more hydrophobic, or aimed to allow pi-stacking with the side chain of W142 (phenyl substituent in compound **2a**). First, replacement of the carboxylate with a large phenyl group (compound **2a**) negatively affected potency [K_d_ and half maximal inhibitory concentration (IC_50_)] and melting temperature (Δ*T*_m_) compared to compound **1** (Table 1). On the contrary, introducing smaller moiety [propyl (compound **2b**)] substantially increased the in vitro potencies of the compounds (Table 1). Notably, the smallest substituents, methyl in compound **2c** and hydrogen in compound **2d**, dramatically increased binding by about 1000-fold [K_d_ (compound **2c**) = 5 nM, K_d_ (compound **2d**) = 10 nM] and showed best potencies in the other orthogonal assays (Table 1).

**Table 1:**
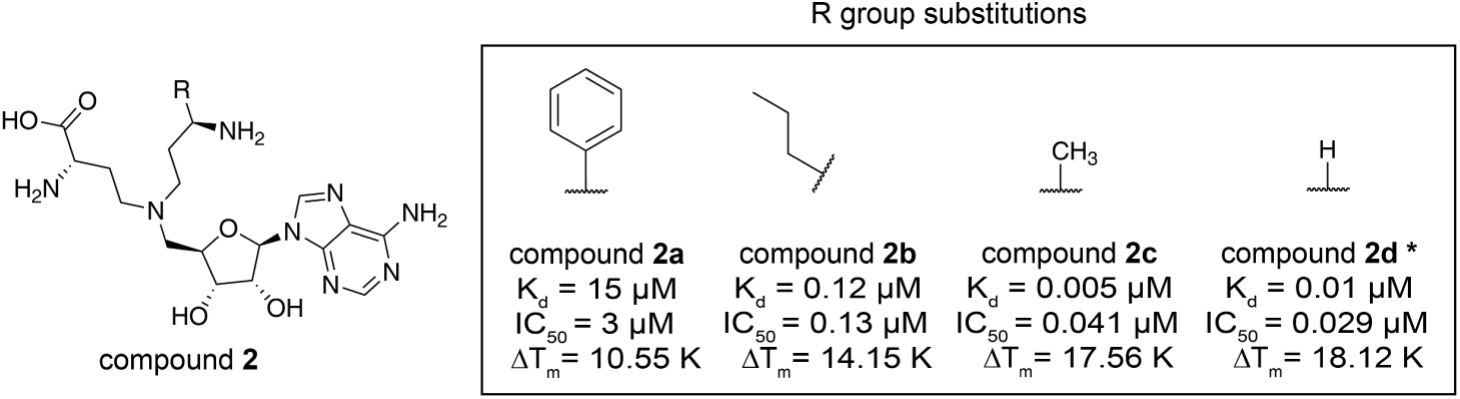
Structure– Activity Relationship of Compound **2** Derivatives^a^

a Chemical structures of compound **2** series. The scaffold structure (left) and the R group substitutions (right) for compound **2** are represented. Measured K_d_, IC_50_, and ΔT_m_ values for each compound are listed. Compound **2d** was taken as a reference from the previous study.^20^

Additional efforts to investigate the structure-activity relationship (SAR) included modifications of the 1-amino group (compounds **3a** and **3b**) and variation of the length of the diamine chain (compounds **3c** and **3d**) (Table 2). Modification of the amine reduced potency by about 10-fold (monomethylamine, compound **3a**), 100-fold (dimethylamine, compound **3b**) compared to compound **2d**, which suggested that the hydrogen bond donating property of the amine was critical for ligand potency. Additionally, the two-carbon linker analogue (compound **3c**) was slightly less potent than compound **3a** (three-carbon linker), whereas a four-carbon linker (compound **3d**) reduced potency about 30-fold, which suggested that the position of the hydrogen bond donating amine was critical for the potency as well.

**Table 2:**
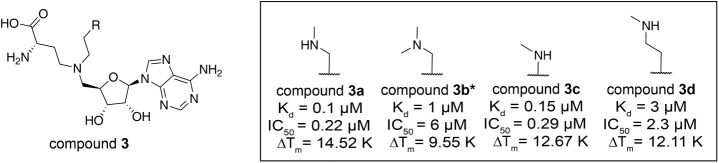
Structure– Activity Relationship of Compound **3** Derivatives^a^

a Chemical structures of compound **3** series. The scaffold structure (left) and the R group substitutions (right) for compound 3 are represented. Measured K_d_, IC_50_, and ΔT_m_ values for each compound are listed. GSK2807 here was named as compound **3b** for a comparison with other analogues.

To understand further the determinants of binding affinity in atomic detail, several key KMT9/ligand cocrystal structures were solved including those for compounds **2a**, **2b**, **2c**, **3a**, and **3b** with well-defined density maps (Figure S2A–E). In the KMT9/compound **2b** complex, the phenyl moiety of the ligand formed unfavorably close contacts with the side chain of W142 instead of expected π-π interaction, which likely explains the weak binding (Figure 2A). The propyl substitution was partially positioned in the substrate channel of KMT9 without forming unfavored contacts by the surrounding residues and part of the alkyl chain extended out of the substrate channel exposing to the solvent (Figure 2B). As a comparison, the methyl moiety of compound **2c** was completely buried in the substrate channel formed favorable hydrophobic interactions with the side chains of W142, Y125, and A141 (Figure 2C). Superposition of KMT9/compound **1**, **2a**, **2b**, **2c**, and **2d** complexes showed that the corresponding ligand superimposed well and the free amine formed similar hydrogen bonds with corresponding D28, N122 and P123 of KMT9 (Figures 2D and S2F,G), which demonstrates that the proper size of the substitution is critical for determining the potency of the compounds. As reported in our recent study, the water-mediated hydrogen bond between the amine group and surrounding residues were supposed to contribute to its high potency for compound **2d**.^20^ Together, the dramatic gain of potency of compounds **2c** and **2d** compared to compound **1** can be mostly explained by the avoidance of unfavorable contacts. In addition, the available space can either be filled with methyl (compound **2c**) forming hydrophobic contacts or with water molecules (compound **2d**) engaging in a hydrogen bonding network.

**Figure 2:**
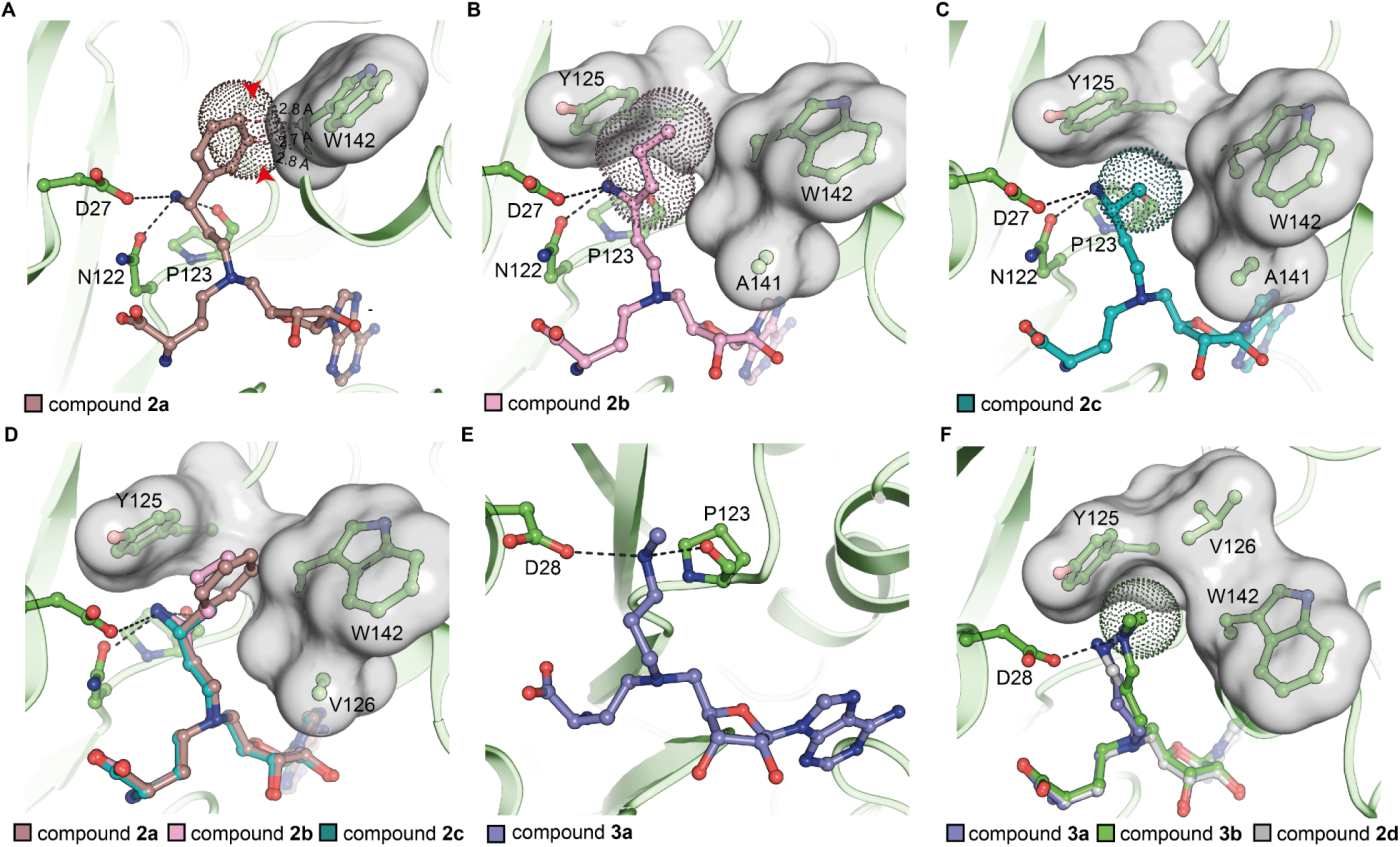
Ligand bound KMT9 structures. (A) Unfavored contacts between compound **2a** and W142 of KMT9α­(PDB code: 9FKG) (B, C) Hydrophobic contacts between compound **2b** (B)**/2c** (C) and KMT9α in the substrate channel (PDB code: 9FKM and 9FKV). (D) Superimposition of compound **2a**-/**2b**-/**2c**- bound KMT9 structures.(E) Hydrogen bonds between the methylamine of compound **3a** and KMT9α (PDB code: 9FKW).(F) Superimposition of compounds **2d-**, **3a-**, and **3b-** bound KMT9 structures. Compound **2a** (brown), **2b** (pink), **2c** (cyan), **2d** (white), **3a** (blue), **3b** (green) and protein residues (green) are represented as sticks. KMT9α is shown as ribbon (green). Part of the substrate channel of KMT9α is shown as surface (gray). Van-der-Waals radius of compounds are shown as dots. Hydrogen bonds between ligands and KMT9 residues are indicated as gray dash lines. Unfavored contacts between compound 2a and W142 of KMT9α are represented as red dash lines.

Notably, methylation of the amine group (compound **3a**) reduced hydrogen bonding with neighboring amino acid side chains (Figure 2E) and caused conformational adaptations of the diamine chain in the substrate channel, which provided a plausible explanation of reduced ligand potency. Accordingly, potency of the dimethyl derivative compound **3b** was even further reduced compared to compound **3a** (Figure 2F). Collectively, we identified lead compounds with low nanomolar potency as well as providing structural insights into the determinants of high affinity KMT9 binding.

### Lead Optimization of Compound **2d**

Bisubstrate SAM analogues were expected to show poor cellular activity due to the unfavored biophysical properties of the SAM backbone (high polarity). To optimize the lead compound **2d**, we considered three parts of the lead compounds: the adenosine moiety, the diamine branch, and the methionine moiety (Figure 3A). First, we focused on the methionine moiety of compound **2d**, which is supposed to be the most polar part in the compound. However, attempts to delete the free amine (compound **4a**) or replace the carboxylate with difluoromethyl (compound **4b**) or trifluoromethyl (compound **4c**) all significantly reduce its potency (Table 3). Similarly, replacing the methionine moiety with different heterocycles also proved to be detrimental to the potency (compounds **4d**–**4g**), which indicates a critical role of the methionine part in maintaining the potency of compound **2d** (Table 3).

**Figure 3:**
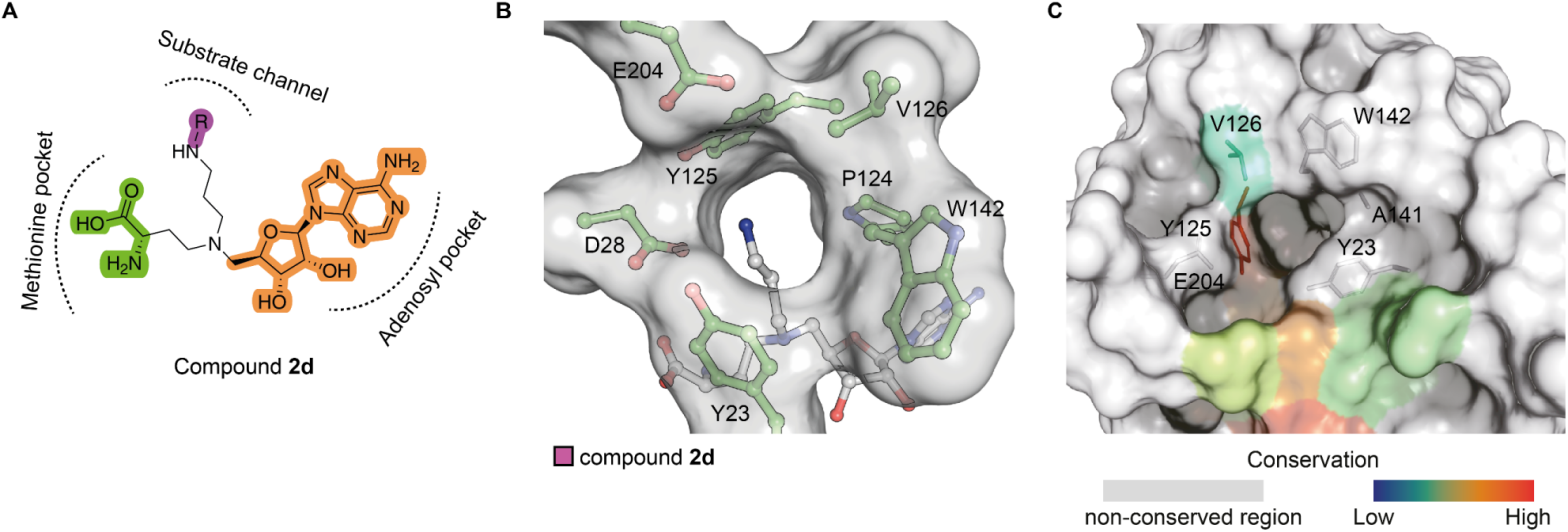
Lead optimization of compound **2d**. (A) Strategies of lead optimization for compound **2g**. (B) Unoccupied substrate pocket in compound **2g**-bound KMT9 complex structure. (C) Structural conservation of KMT9a substrate pocket across Rossmann-fold MTs. Residue conservation is shown with a color scheme ranging from blue (low conservation) to red (high conservation); The residues which are not found in structural conserved region are colored in white.

**Table 3:**
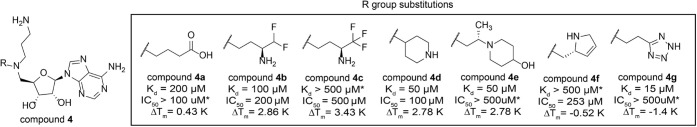
Structure– Activity Relationship of Compound 4 Derivatives^a^

a Chemical structures of the compound **4** series. The scaffold structure (left) and the R group substitutions (right) for compound 4 are represented. Measured K_d_, IC_50_, and ΔT_m_ values for each compound are listed. For compound **4c** and **4f**, The K_d_ could not be determined accurately due to the low potency. For compound **4a**, **4e**, and **4g**, the IC_50_ could not be determined accurately due to the low potency.

Next, we examined the diamine branch of compound **2d** in the crystal structure and noted that there was still unoccupied space in the substrate channel adjacent to the amine moiety, which could be used for further extension of the primary amine (Figure 3B). Notably, structure-based multiple sequence alignments showed that amino acids forming this additional subpocket in KMT9 (Y23, Y125, V126, A141, W142, E204) were not conserved in other closely related Rossmann-fold methyltransferases (Figures 3C and S3A,B). Following a structure-based optimization, we extended the amine of compound **2d** with benzyl (compound **5a**), phenethyl (compound **5b**), or phenylpropyl (compound **5c**) (Table 4). Consequently, compound **5b** exhibited the highest potency (*K*_d_ = 10 nM, IC_50_ = 0.034 nM, Δ*T*_m_ = 20.91 K). We then solved the crystal structure of the KMT9/compound **5b** complex, which confirmed that the phenethyl moiety specifically filled the additional hydrophobic pocket of KMT9 and similar hydrogen bonds were observed between the amine and surrounding residues (Figures 4A and S3C).

**Table 4:**
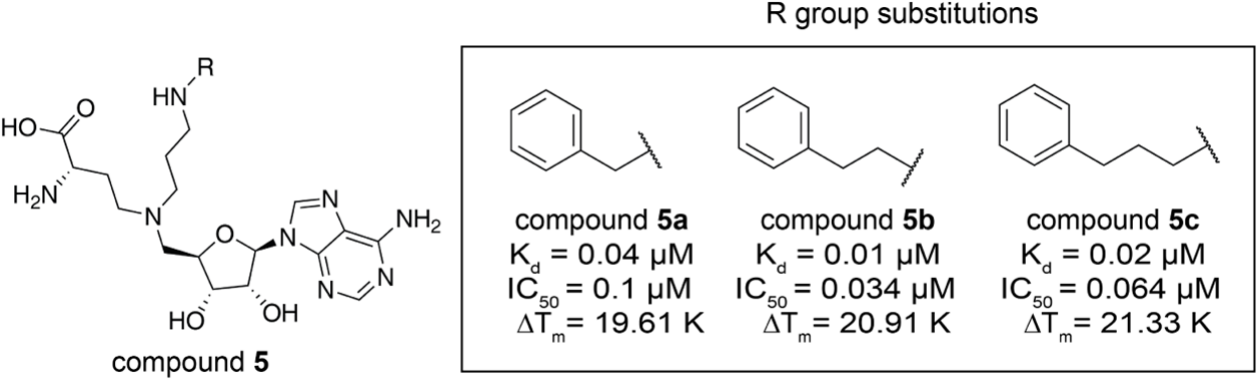
Structure– Activity Relationship of Compound 5 Derivatives^a^

a Chemical structures of compound 5 series. The scaffold structure (left) and the R group substitutions (right) for compound 5 are represented. Measured K_d_, IC_50_, and ΔT_m_ values for each compound are listed.

**Figure 4:**
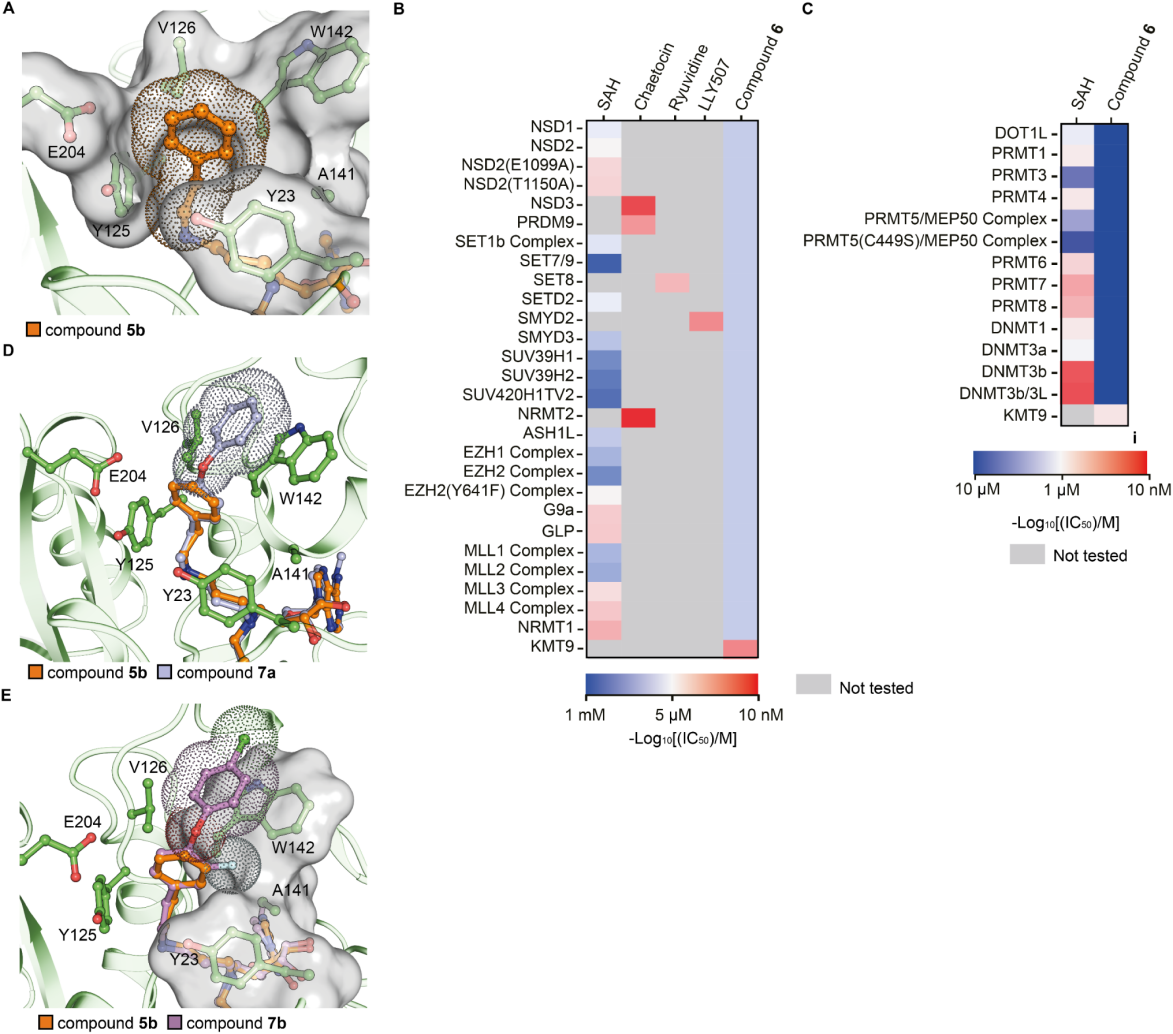
Lead optimization of compound **5b**. (A) Hydrophobic contact between the phenyl moiety of compound **5b** and substrate channel of KMT9 (PDB code: 9FL4). (B, C) Selectivity of compound **6** against SET-domain containing and Rossmann-fold MTs. (D, E) Predicted binding mode of compound **7a-**/**7b-** bound KMT9 structures based on compound **5b**/KMT9 crystal structure. Ligands and key residues are represented by sticks. Substrate channel of KMT9a is shown as surface. Van-der-Waals radius of corresponding moiety are shown as dots.

Crystal structure comparisons showed that in KMT9α the N6-amine of the adenosine moiety forms one hydrogen bond with the side chain of D103, which we previously found to be critical for SAM binding.^8,20^ In contrast, in SET domain methyltransferases, the N6-amine is buried within a very small pocket forming tight contacts with the protein main chain, which is supposed to preclude even small extensions.^21^ Consequently, the N6-amine was monomethylated to preserve the hydrogen bond in KMT9α but potentially introduce a steric clash in SET domain methyltransferases. The 7-nitrogen in the adenine and oxygen in the ribose were replaced by carbon to improve the lipophilicity of the compound as described in our previous study.^20^ Together, introduction of these modifications resulting in compound **6** (Table 5) only moderately affected the in vitro potency (K_d_ = 30 nM). To verify selectivity of compound **6**, we tested it against a panel of 27 SET domain-containing and 14 Rossmann-fold methyltransferases. The data demonstrated that compound **6** is selective for KMT9 against other MTs (Figure 4B,C).

**Table 5:**
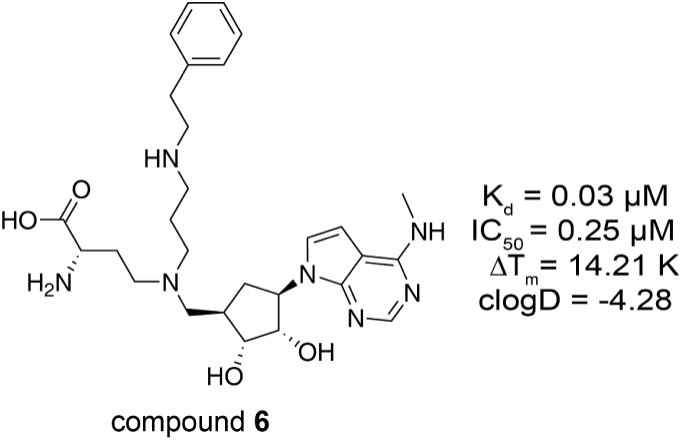
Structure– Activity Relationship of Compound 6^a^

a Chemical structure of compound 6. Measured K_d_, IC_50_, and ΔT_m_ values for each compound are listed. clogD at pH 7.4 is calculated by MarvinSketch.

Next, we aimed to optimize cell membrane permeability and cellular potency of our lead compound. The modifications introduced into compound **6** already strongly improved the calculated logD value (clogD = −4.28) compared to that of compound **2d** (clogD = −7.77). Modeling based on the crystal structure of the KMT9/compound **5b** complex suggested that compound potency and lipophilicity could be further improved by extension of compound **6** with a phenoxy moiety in the substrate branch (compound **7a**), which was predicted to allow parallel π-π interactions with the side chain of W142 (Figure 4D). Moreover, addition of a fluoro and chloro atom (compound **7b**) was predicted to increase binding free energy due to the formation of extra contacts with KMT9α­(Figure 4E). Indeed, compound **7b** showed both improved in vitro potency and lipophilicity compared with compound **6** (Table 6).

**Table 6:**
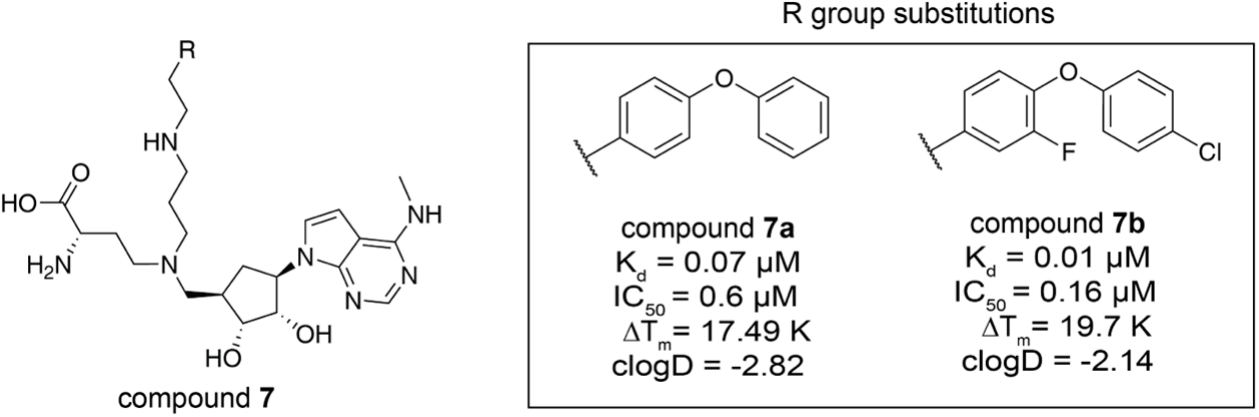
Structure– Activity Relationship of Compound 7 Derivatives^a^

a Chemical structures of compound **7** series. The scaffold structure (left) and the R group substitutions (right) for compound **7** are represented. Measured K_d_, IC_50_, and ΔT_m_ values for each compound are listed. clogD at pH 7.4 is calculated by MarvinSketch.

Alone with biochemical profiling of compound **7b**, we found that compound **7b** significantly increased thermal stability of endogenous KMT9 in SW480 cell lysates by Western blotting (Δ*T*_m_ = 10.9 K, Figure 5A,B). As a negative control, we used KMT5A, which was not stabilized by compound **7b** (Δ*T*_m_ = 1.1 K, Figure S4A,B). To investigate cellular target engagement of compound **7b**, cellular thermal shift assays (CETSA)^22^ using SW480 tumor cells were performed. However, when we treated intact SW480 cells with compound **7b**, almost no Δ*T*_m_ was observed compared to the cell lysates, which suggests a low cell membrane permeability of compound **7b** (Δ*T*_m_ = 1.8 K, Figure 5C,D).

**Figure 5:**
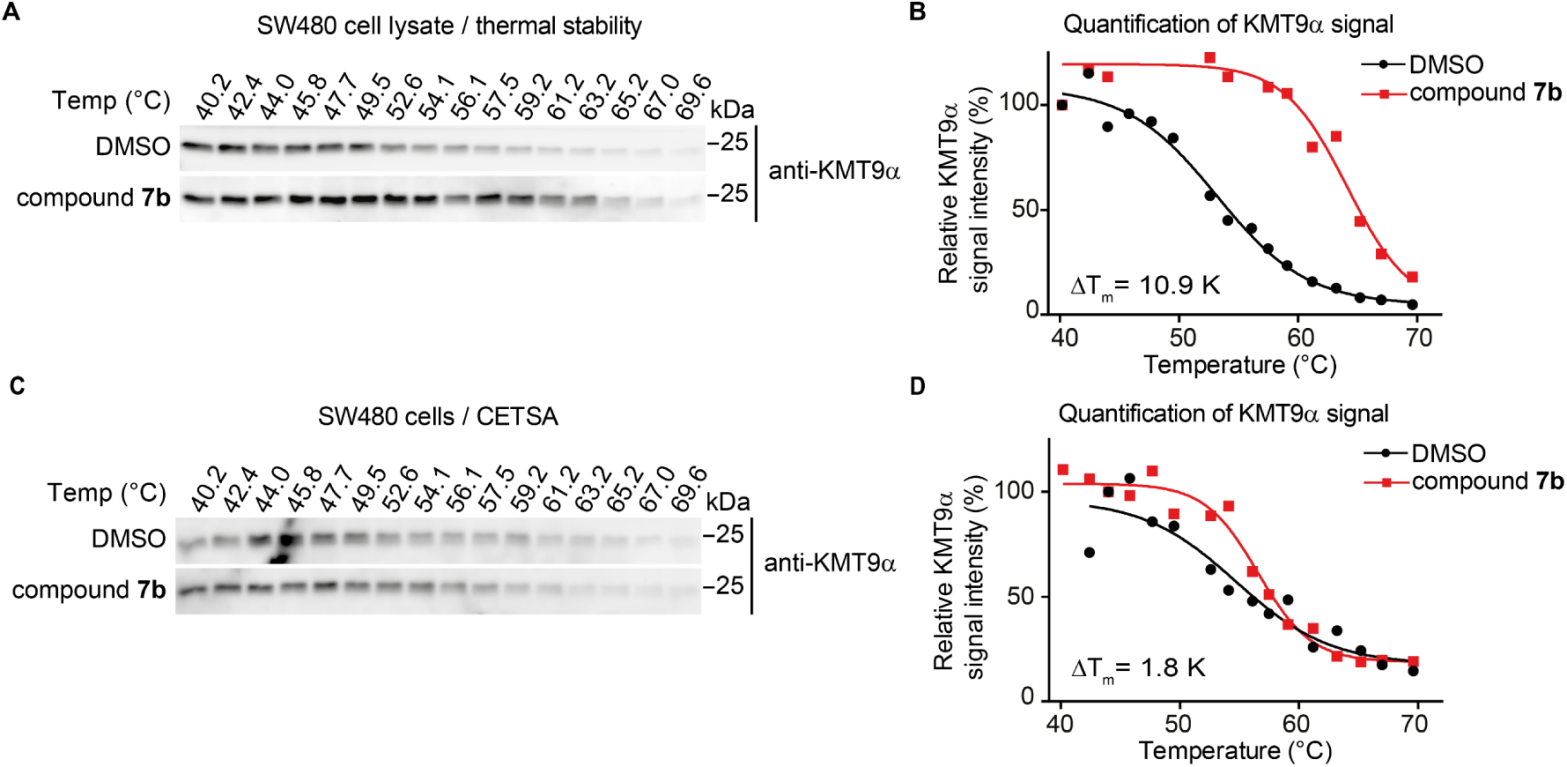
Cellular target engagement of compound **7b**. (A, B) CETSA for KMT9a in SW480 cell lysate treated with vehicle (DMSO), 10 μM compound **7b**. Representative Western blots (A) and quantification (B) showing increased melting temperatures (Δ*T*_m_) of endogenous KMT9a upon treatment with compound **7b** compared to DMSO. (C, D) CETSA for KMT9a in SW480 cells treated with vehicle (DMSO), 10 μM compound **7b**. Representative Western blots (C) and quantification (D) showing almost no increased melting temperatures (Δ*T*_m_) of endogenous KMT9a upon treatment with compound **7b** compared to DMSO.

To increase cell membrane permeability, a prodrug approach was applied.^23^ Accordingly, the carboxylate of compound **7b** was transformed to the more lipophilic ethyl ester, which creates steric hindrance for the direct binding toward KMT9 based on the structure (compound **8**, Figure 6A). As expected, compound **8** showed more than 10,000-fold decreased binding affinity compared to compound 7b (K_d_ > 400 μM, Figure 6B). To generate control compounds, we replaced the “amine anchor” in the substrate branch of compounds **7b** and **8** with a hydrogen bond accepting oxygen resulting in compounds **7b-N** and **8-N**, which is predicted to have improved clogD and thus membrane permeability (Figure 6C). The in vitro potency of compound **7b-N** was, as predicted, more than 1000-fold reduced (K_d_ ∼ 56 μM) compared to compound **7b** (Figure 6D). CETSA assays using intact SW480 cells showed an obviously increased Δ*T*_m_ for KMT9 upon treatment with prodrug compound **8** compared to the negative control compound **8-N** (Δ*T*_m_ = 5 K for compound **8**, Δ*T*_m_ = −0.6 K for compound **8-N**, Figure 6E–H). Given the weak binding of compound **8** to KMT9, it indicates that compound **7b** can be released by the esterase in cells. In comparison, no Δ*T*_m_ was observed for KMT5A in SW480 cells treated with prodrug compound **8** (Δ*T*_m_ = −0.6 K, Figure S4C,D). In addition, CETSA assays were also performed in Caco-2 tumor cell lines, which revealed obvious stabilization of KMT9 after treating the cells with prodrug compound **8** instead of compound **8-N** (Δ*T*_m_ = 6.1 K for compound **8**, Δ*T*_m_ = 0.1 K for compound **8-N**, Figure S4E–H). Together, we successfully developed compound **8** leading to cellular target engagement by using prodrug strategy.

**Figure 6:**
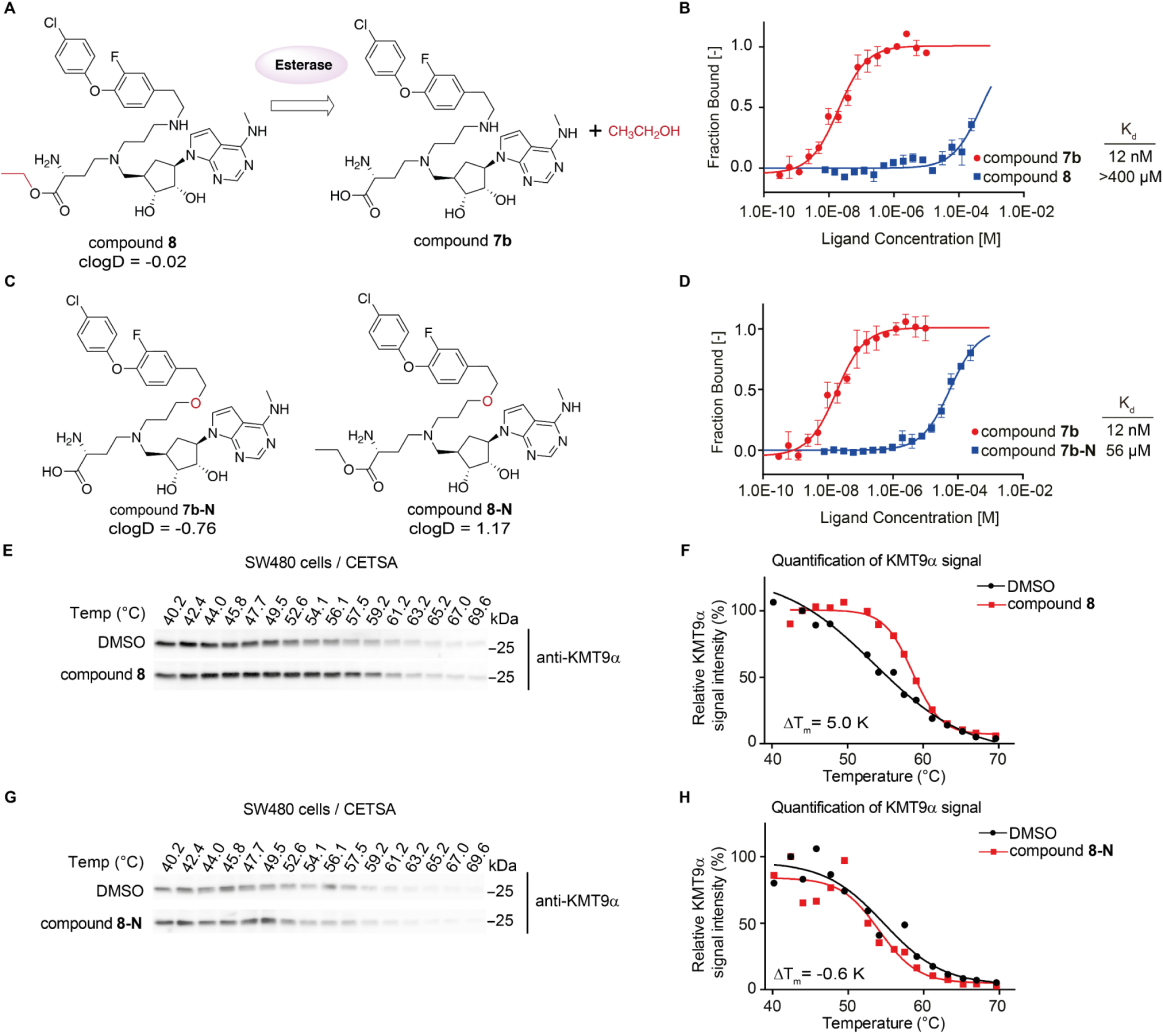
Cellular target engagement of compound **8**. (A) Scheme of the cleavage of ethyl ester prodrug, compound **8**, by esterase. (B) MST assay to determine the *K*_d_ of compound **8** and compound **7b** binding to KMT9. Data represent means ± s.d (*n* = 3). (C) Chemical structures of compound **7b-N** and compound **8-N**. (D) MST assay to determine the *K*_d_ of compound **7b** and compound **7b-N** binding to KMT9. Data represent means ± s.d (*n* = 3). (E, F) CETSA for KMT9a in SW480 cells treated with vehicle (DMSO), 10 μM compound **8**. Representative Western blots (E) and quantification (F) showing increased melting temperatures (Δ*T*_m_) of endogenous KMT9a upon treatment with compound **8** compared to DMSO. (G, H) CETSA for KMT9a in SW480 cells treated with vehicle (DMSO), 10 μM compound **8-N**. Representative Western blots (G) and quantification (H) showing no increased melting temperatures (Δ*T*_m_) of endogenous KMT9a upon treatment with compound **8-N** compared to DMSO.

### Cellular Activity of the Prodrug Compound **8**

Having demonstrated the target engagement to KMT9 after treatment with compound **8** in cells, we investigated whether cellular H4K12me1 levels were reduced in the presence of compound **8** as previously observed upon KMT9 knockdown or inhibition.^8,20^ Indeed, treatment of PC-3 M cells of compound **8** reduced H4K12me1 levels, whereas no effect was observed upon treatment with the control compound **8-N** (Figure 7A).

**Figure 7:**
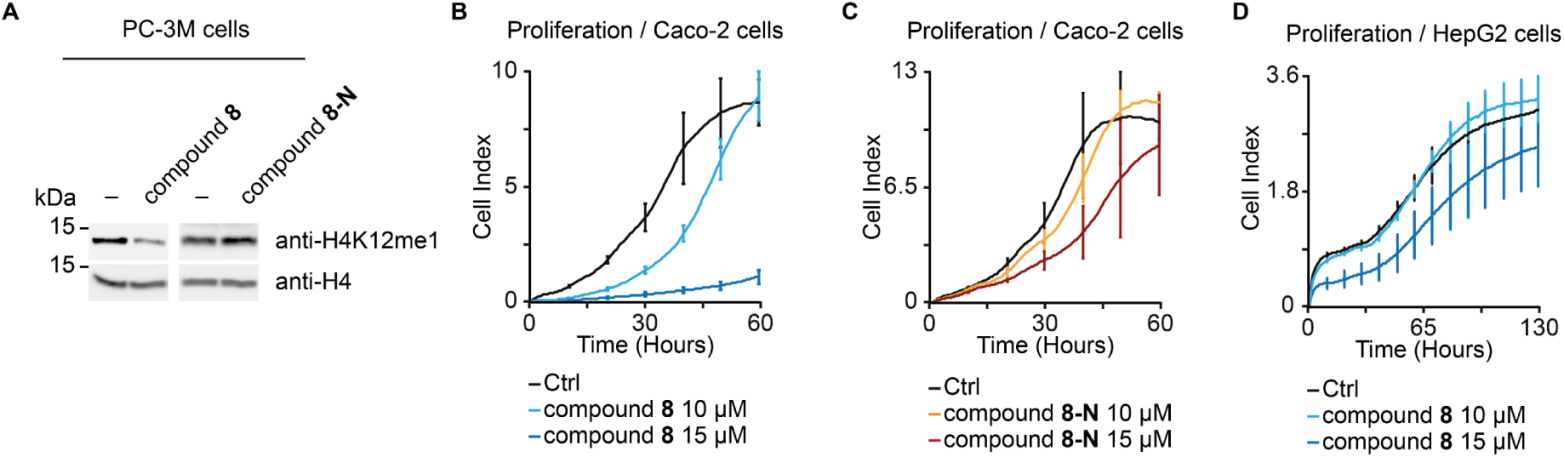
Cellular activity of prodrug compound **8**. (A) Levels of H4K12me1 in PC-3 M prostate tumor cells cultured in the presence of DMSO (−), 10 μM compound **8** or compound **8-N** for 3 days were analyzed by Western blot using the indicated antibodies. Histone H4 was used as control. (B, C) Proliferation of Caco-2 cells treated with DMSO (Ctrl), compound **8**, or compound **8-N** were measured by Xcelligence. Data represent means ± s.d (*n* = 3). (D) Proliferation of HepG2 cells upon treatment of DMSO (Ctrl) and compound **8**.

Next, we examined whether KMT9 inhibition reduced proliferation of colorectal carcinoma cell lines, in which KMT9 was reported to control stemness and growth of colon cancer.^24^ Compared to DMSO or the control compound **8-N**, treatment with compound **8** strongly suppressed proliferation of Caco-2 cells (Figure 7B,C), which was comparable to that observed upon KMT9 knockdown (Figure S5A). Furthermore, antiproliferative effects mediated by compound **8** were not restricted to Caco-2 cells but also found in SW480 tumor cell lines (Figure S5B,C).

In addition, compound **8** was tested in human liver hepatocellular carcinoma (HepG2) cells, which was previously observed to be insensitive to KMT9 knockdown or inhibition.^8,20^ CETSA assays using HepG2 cells showed a strongly increased Δ*T*_m_ for KMT9 upon treatment with compound **8** compared to the DMSO control (Δ*T*_m_ = 8 K), whereas no Δ*T*_m_ was observed for KMT5A (Δ*T*_m_ = 0.1 K), which indicated that prodrug compound **8** can target endogenous KMT9 after releasing the ethyl group by the esterase in HepG2 cells (Figure S5D–G). Consistent with the previous knockdown and inhibition results, treatment of HepG2 cells with compound **8** did not affect cell proliferation (Figure 7D).

### Chemistry

The synthesis of the ribose derivatives, namely compounds **1, 2a-d, 3a-d, 4a-g and 5a-c**, started from adenosine (see Scheme 1), which was first selectively protected on the diol as an acetonide (**si1**). The terminal unprotected alcohol was then converted to an azide (**si2**) using DPPA (diphenyl phosphorazidate), DBU (1,8-diazabicyclo[5.4.0]­undec-7-ene) and NaN_3_. After Boc-protection of the aromatic amine of the adenine (**si3**), the azide was reduced with H_2_ and Pd/C to give the terminal free amine compound and key intermediate **si4**. Boc-protection of the adenine was not necessary in all cases, as described in the Supporting Information.

**Scheme 1:**
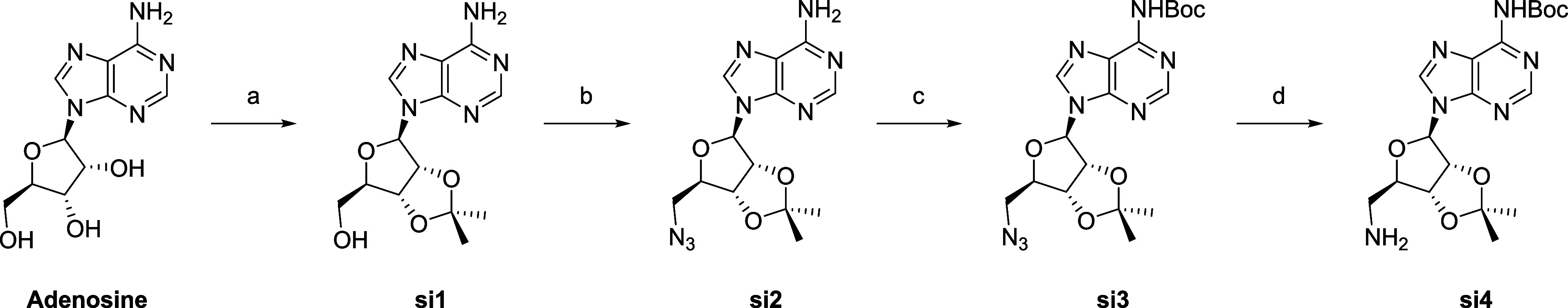
Synthesis of the Key Intermediate si4^a^

a Reagents and conditions: (a) CH­(OEt)_3_, *p*TsOH, acetone, rt, 16 h, 92%; (b) DPPA, DBU, 1,4-dioxane, rt, 16 h then NaN_3_, 15-crown-5, reflux, 6 h, 80%; (c) Boc_2_O, NaH, THF, 0 °C to rt, 90 min, 80%; (d) H_2_, Pd/C, EtOAc/MeOH, rt, 16 h, quant.

The preparation of the “methionine-like” amino acid side chain of the inhibitors is shown in Scheme 2 and started from *N*-Boc-d-aspartic acid 1-(*tert*-butyl) ester, which was reduced to the alcohol **si5** and then again oxidized to the corresponding aldehyde **si6**. The subsequent reductive amination in DCE with AcOH and STAB (NaBH­(OAc)_3_) allowed the synthesis of the secondary amine of intermediate **si7** (Scheme 3), which was then used in a second reductive amination step with different aldehydes to give the desired inhibitor derivatives after a final deprotection step. Most of the aldehydes used were commercially available and the detailed synthesis of the corresponding aldehydes for the preparation of compounds **5a**–**5c** is shown in Scheme S2. Their synthesis started from benzaldehyde, 2-phenylacetaldehyde and 3-phenylpropanal, which were first reacted with 3-amino-1-propanol and then Boc-protected. Finally, the terminal alcohol was oxidized by Swern oxidation using oxalyl chloride and DMSO in DCM. The synthesis of the final compound **1** is shown in Scheme S1 and results from a double reductive amination of the free amino group of **si4** with **si6** using 4 equiv of **si6**.

**Scheme 2:**
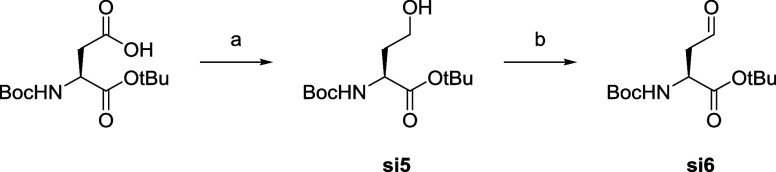
Synthesis of the “Methionine-Like” Amino Acid Side Chain^a^

a Reagents and conditions: (a) isobutyl chloroformate, Et_3_N, THF, −5 °C, 30 min then NaBH_4_, MeOH, 0 °C to rt, 2 h, 45%; (b) (COCl)_2_, Et_3_N, DMSO, CH_2_Cl_2_, −78 °C to rt, 60 Min, 78%.

**Scheme 3:**
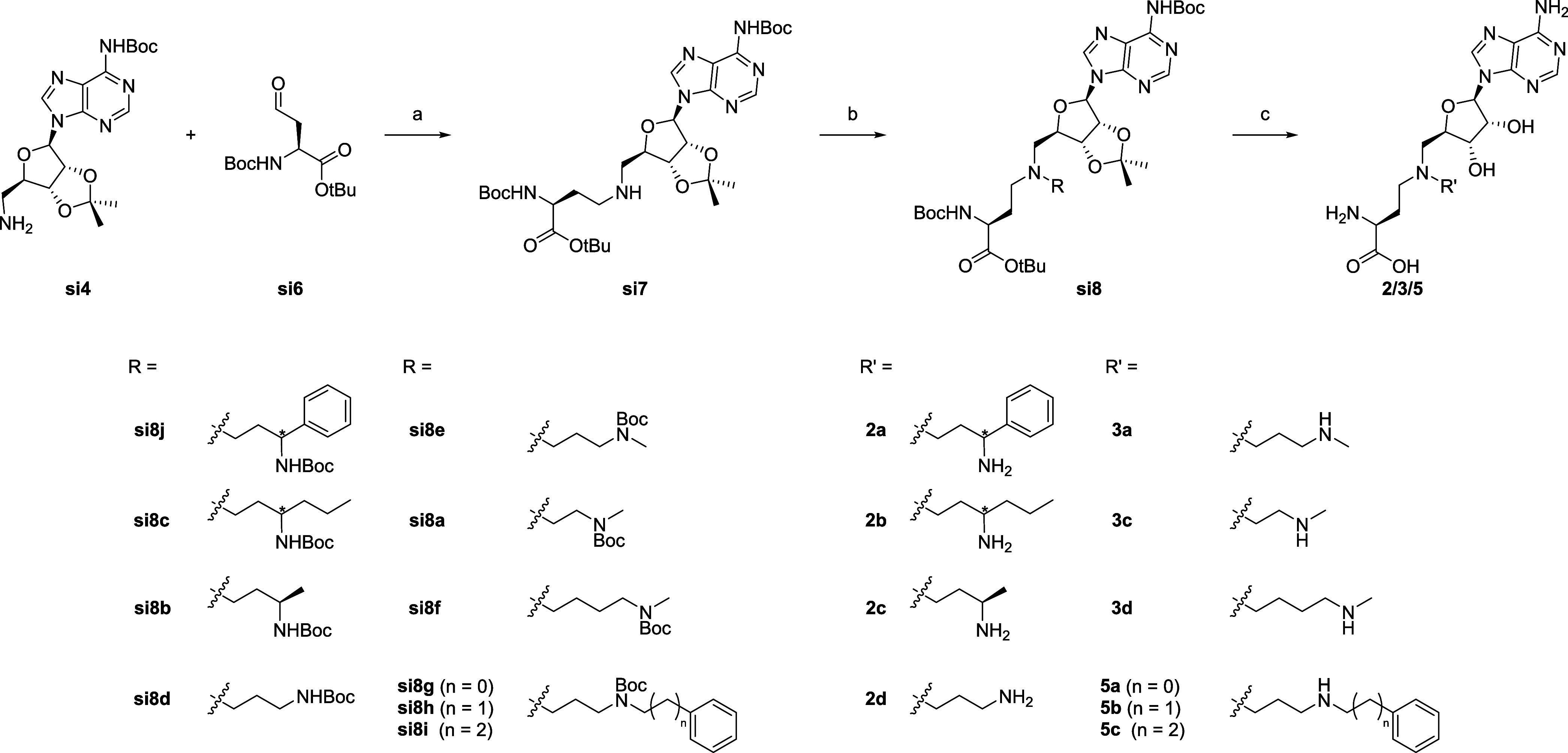
Synthesis of the Final Compounds 2/3/5 Derivatives^a^

a Reagents and conditions: (a) AcOH, DCE, rt, 4 h, then NaBH­(OAc)_3_, rt, 4 h; (b) aldehyde 13a-d, 66a-f, AcOH, DCE, rt, 4 h, then NaBH­(OAc)_3_, rt, 4 h, then 70 °C, 12 h; (c) TFA/H_2_O (4:1), rt, 6-16 h.

The synthesis of the compound **4** derivatives with modified side chains to optimize the physicochemical properties of the inhibitors followed a similar route as for the previously presented compounds, starting from the intermediate **si4**, followed by two reductive amination steps and a final deprotection step. The synthesis of compounds **4a**–**g** is described in detail in Schemes S3–S6.

For the further optimization of the inhibitors the adenine was sought to be replaced by a 7-deazapurine and the ribose was replaced by a carbocyclic sugar mimic. The synthesis started from (1*R*,2*S*,3*R*,5*R*)-3-amino-5-(hydroxymethyl)­cyclopentane-1,2-diol hydrochloride and 4,6-dichloropyrimidine-5-acetaldehyde. Ring closure under reflux overnight allowed the synthesis of the compound **si42**, which was subsequently protected as acetonide and a nucleophilic aromatic substitution with methylamine gave the intermediate **si44**. In a similar manner as described before compound **si47** was obtained after azide synthesis and reduction and Boc-protection of the *N*-6 amine (see Scheme 4).

**Scheme 4:**
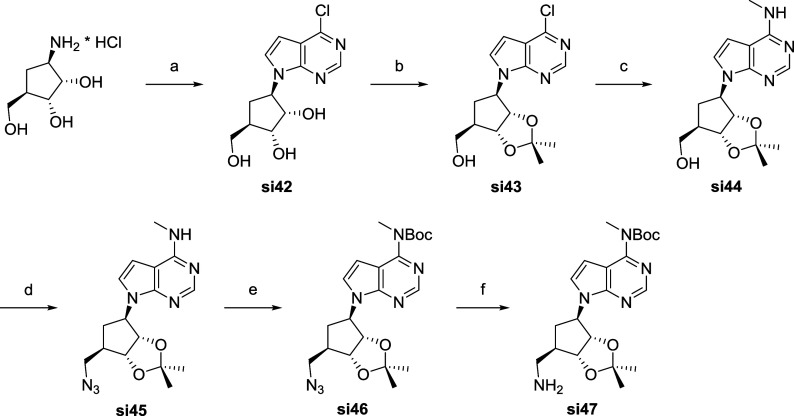
Synthesis of the Carbasugar Derivative si47^a^

a Reagents and conditions: (a) 4,6-dichloropyrimidine-5-acetaldehyde, Et_3_N, abs. EtOH, reflux, 16 h, 97%; (b) CH­(OEt)_3_, pTsOH, acetone, rt, 16 h, 58%; (c) CH_3_NH_2_ (33% in EtOH), *n*-BuOH, μ-waves, 120 °C (100 W), 25 min, 79%; (d) DPPA, DBU, 1,4-dioxane, rt, 16 h; then NaN_3_, 15-crown-5, reflux, 6 h, 44%; (e) Boc_2_O, NaH, THF/DMF, 0 °C to rt, 5 days, 74%; (f) H_2_, Pd/C, EtOAc/MeOH (1:1), rt, 6 h, 48%.

Subsequently, the intermediate **si47** was subjected to a first reductive amination with **si6**. As with the other compounds, the amino acid side chain was introduced. A second reductive amination was then employed for the synthesis of the diamine side chain of the compounds **6, 7a** and **7b** (see Scheme 5). The detailed synthetic route for the phenoxybenzene side chains is depicted in Scheme S7 and followed a sequence of nucleophilic substitutions and final oxidation of a terminal alcohol to obtain the corresponding aldehyde.

**Scheme 5:**
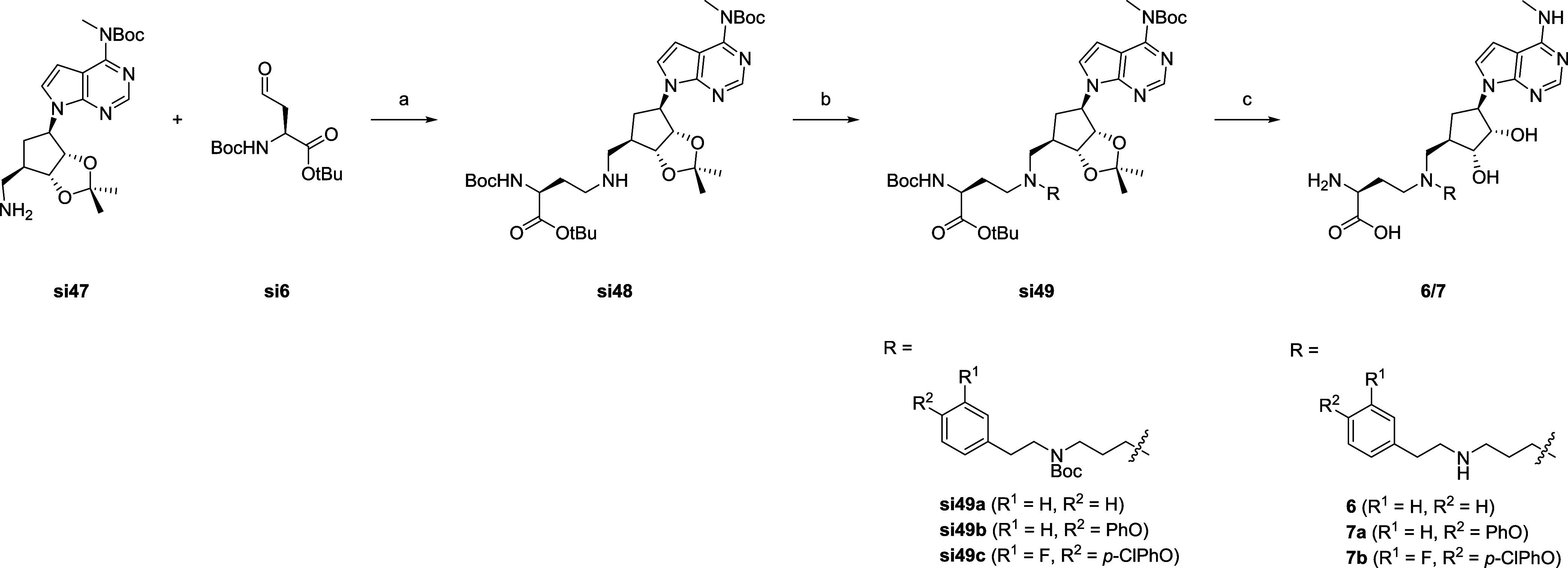
Synthesis of the Final Compounds **6**, **7a** Aand **7b** ^a^

a Reagents and conditions: (a) AcOH, DCE, rt, 3 h; then NaBH­(OAC)_3_, 4 h, 31%; (b) AcOH, **si13a** or **si13d** or **si61,** DCE, rt, (4 h for **si49a-b**), (3.5 h for **si49c**); then NaBH­(OAC)_3_, (rt, 4 h, then 70 °C, 12 h for **si49a-b**), (rt, 3 h **for si49c**); (c) TFA/water (4:1), rt, (6-12 h for **6/7a**), (4 h for **7b**).

In the synthesis of the ethyl ester prodrug **8** and the negative control compounds (**7b-N, 8-N**), the order of the reductive amination was changed. Initially, the diamine side chain was introduced, followed by the attachment of the amino acid side chain, and culminating in the deprotection of the desired final compounds. The detailed synthesis of these compounds, including the variation of the side chain to an ether in the case of the negative control compounds, is outlined in the Schemes S8 and S9. 2-(4-(4-chlorophenoxy)-3-fluorophenyl)­ethan-1-ol was treated with ethyl acrylate and Cs_2_CO_3_ in acetonitrile resulting in the ether analogue in the side chain of compound **7** *b* **/8**. The ethyl ester was then reduced to the corresponding alcohol with LiAlH_4_ and the resulting alcohol was reoxidized with Dess-Martin periodinane to give the aldehyde (see Scheme S8). Reductive amination and subsequent deprotection allowed access to compound **7b-N**, which was again converted to the ester using TMS-Cl and ethanol to obtain **8-N** (see Scheme S9).

## Discussion and Conclusions

Here, we reported the strategies in the structure guided inhibitor development of a micromolar hit (compound **1**) to a prodrug (compound **8**) with cellular activity. The parental compound **7b** binds KMT9 with nanomolar affinity and the prodrug compound **8** blocks proliferation of colon cancer cells at micromolar concentrations. We verified the selective target activity of compound **8** in cells by using a structural analog, compound **8-N**, as well as cell lines that are insensitive to KMT9 depletion and, as anticipated, do not respond to compound **8**. Our results provide a proof-of-concept for druggability and suitability of KMT9 as a potential therapeutic target.

Designing bisubstrate inhibitors is an established strategy which has been successfully applied to several other PMTs,^25–37^ such as TC-5115 for inhibiting histone methyltransferase MLL1^34^, prodrug inhibitor SKI-73 of CARM1^32^, a 5,5-bicyclic nucleoside-derived PRMT5 inhibitor,^36^ and YD1130 as a highly selective PRMT4 inhibitor.^37^ Following a structure-guided optimization, we achieved high potency and selectivity for compound **7b**. Distinct from the conserved SAM binding pocket of PMTs, the specificity and plasticity of the substrate-binding pocket accounts for the high degree substrate selectivity, providing opportunity to develop selective inhibitor by targeting the pocket.^38,39^ Consequently, in the substrate channel, we extended the “amine anchor” with a 1-(4-chlorophenoxy)-2-fluorobenzene that accommodates to and fills the hydrophobic pocket of the KMT9 substrate channel and potentially forms π-π interactions with W142. In addition, in the SAM binding pocket, we used a distinctive feature between SET domain-containing and Rossmann-fold PMTs. In SET domain-containing PMTs, the N6 of the adenosine moiety is in close contact with the protein main chain, which makes even small extensions intolerable. Thus, the N6-methyl extension of compound **8** may importantly contribute to compound selectivity. Together, these modifications could account for the high potency and specificity of compound **8**. The structure determinants for the high potency as well as the SAR information provided here may facilitate the future design of KMT9 inhibitors with better biophysical properties. In addition, to overcome the problem of the cellular permeability of the bisubstrate compound, a prodrug strategy was used to mask the highly polar carboxylate group, which was proven to show specific on-target and antiproliferation effect in colon cancer cells. Thus, our prodrug inhibitor together with the control compound may also help to delineate the biological function of KMT9 in physiological and pathological conditions.

## Experimental Section

### General Remarks

All final compounds are ≥ 95% pure by HPLC analysis besides compound **2a**, **2c**, **5b** and **5c** which are >93% purity and compound **6** and **7b** that are >94% purity. Purity was determined by HPLC and additional structural characterization was performed by NMR and mass spectrometry as described below. All reactions were carried out in glassware under inert (nitrogen) atmosphere. All used chemicals and reagents were purchased from commercial sources and were used without further purification. Solvents were freshly purified by distillation/drying over molecular sieves following the instructions from the Purification Book. Particularly mentioned anhydrous/dry solvents were purchased from Acros organics. Reactions were monitored by thin-layer chromatography (TLC) performed with Merck alumina plates coated with silica gel 60 F254, silica gel 60 RP-18 F254s or silica gel 60 NH2 F254S (layer thickness: 0.2 mm) and analyzed under UV light (254 and 365 nm) or revealed using KMnO_4_, Bromocresol green, ninhydrin, phosphomolybdic acid or 2,4-dinitrophenylhydrazine (2,4-DNPH) as staining agent. The composition of the mobile phase was adjusted to the compound properties. Yields were not optimized. Flash column chromatography was performed on a Biotage Isolera Prime/One purification system using 40–60 μm prepacked silica gel columns from Biotage, HP-spherical 50 μm prepacked silica gel columns from Interchim (Jumbo Pack), or SNAP Ultra 25 μm prepacked silica gel columns from Biotage. NMR spectroscopy and mass spectrometry were used for product identification. NMR spectra were acquired on a BRUKER Avance 400 spectrometer (400 and 100.6 MHz for 1H and 13C respectively), at a temperature of 303 K unless specified using DMSO-d6 as solvent, or on a Bruker Fourier 300 MHz, where TMS was used as an internal standard. Chemical shifts (δ) are reported in ppm, multiplicity abbreviations are as follows: bs = broad singlet, s = singlet, d = doublet, dd = doublet of doublets, dt = doublet of triplets, *t* = triplet, td = triplet of doublets, q = quartet, *m* = multiplet, coupling constant (J) are expressed in Hz. The ^1^H assignment resulted from COSY experiments. Mass spectra were recorded on an Advion expression CMS using an ASAP (Atmospheric Solids Analysis Probe; aka APCI: Atmospheric Pressure Chemical Ionization) as ion source, on a Thermo Scientific Exactive mass spectrometer using electrospray ionization (ESI) as ion source or HR-MS were obtained on a THERMO SCIENTIFIC Advantage. HPLC analysis was performed to determine the purity of final compounds on an Agilent Technologies 1260 Infinity II system using diode array detector (DAD) UV detection at either 210, 230, 248, 254, 260, and 280 nm. Alternatively, the purity and identity were determined on an Agilent LC/MSD 1200 Series (Column: ODS 2000 (50 × 4.6 mm, 5 μm) operating in ES (+) or (−) ionization mode (quadrupole mass spectrometer); *T* = 30 °C; flow rate = 1.5 mL/min; and UV-detection at the wavelength 214 nm. Preparative HPLC was performed for final compounds on an Agilent 1260 Infinity II using UV detection at 210 or 220 nm. As columns were applied either XBridge Prep Shield RP18 5 μm, Phenomenex Kinetex 5 μm XB-C18 100 Å or Welchrom C18, 150 × 20 mm. The flow rate varies between the different methods listed below. For chiral HPLC a Chiralpak IC, 250 mm × 4.6 mm, 5 μm was applied, *T* = 30 °C, detection at 230 nm.

### General Procedures

#### General Procedure for the First Reductive Amination

To a stirred solution of **si4** (1.1 equiv) and aldehyde (1 equiv) in dry DCE (0.12 M based on the aldehyde) was added AcOH (1.1 equiv). The solution was stirred for 4 h at rt, then NaBH­(OAc)_3_ (2.6 equiv) was added and the mixture was stirred for 4 h at rt. After completion, the reaction was quenched by the addition of a 5% aq. NaHCO_3_ solution and the phases were separated. The aqueous phase was then extracted 3 times with CH_2_Cl_2_ and the combined organic phases once with brine. Drying over Na_2_SO_4_, filtration and evaporation afforded the crude product that was subjected to silica gel column chromatography eluting with CH_2_Cl_2_/MeOH (99.5:0.5–90.5:9.5) to afford the secondary amine.

#### General Procedure for the Second Reductive Amination

To a stirred solution of amine **7** (1.1 equiv) and corresponding aldehydes **13a-c, 66a-g** (1 equiv) in dry DCE (0.12 M based on **7**) was added AcOH (1.1 equiv). The solution was stirred for 4 h at rt, then NaBH­(OAc)_3_ (2.6 equiv) was added and the mixture was stirred for 4 h at rt and 12 h at 70 °C. After completion, the reaction was quenched by the addition of a 5% aq. NaHCO_3_ solution and the phases were separated. The aqueous phase was then extracted 3 times with CH_2_Cl_2_ and the combined organic phases were washed once with brine. Drying over Na_2_SO_4_, filtration and evaporation afforded the crude product that was subjected to silica gel column chromatography eluting with CH_2_Cl_2_/MeOH (mostly 99.5:0.5–94:6) to afford the tertiary amines as yellow oils.

#### General Procedure for the Final Deprotection

The protected tertiary amines were dissolved (0.02 M) in freshly prepared TFA/H_2_O (4:1) solution and stirred at rt for 6 h–16 h, then evaporated to give the desired product as foam (2 or 3 TFA salts).

#### General Procedure for the Preparation of Extended *N*-Boc Protected Amino-Alcohols

To a mixture of aldehyde (1 equiv) and 3-aminopropan-1-ol (1 equiv) in anhydrous CH_2_Cl_2_ (0.2 M) was added Et_3_N (2.5 equiv) at rt, and the resulting solution was stirred vigorously for 1 h. Then, Boc_2_O (1.2–3 equiv) followed by NaBH­(OAc)_3_ (2 equiv). The reaction was stirred for an additional 16 h at rt, quenched with saturated NaHCO_3_ solution, and extracted with CH_2_Cl_2_. The combined organics were dried over Na_2_SO_4_, and concentrated under reduced pressure. The crude residue was purified by flash chromatography (CH_2_Cl_2_/MeOH 99:1–95:5).

#### General Procedure for the Boc Protection of Extended Amino-Alcohols

To a solution of amino-alcohol (1 equiv) was added Et_3_N (1.5 equiv) and the mixture was cooled to 0 °C. After few minutes stirring at that temperature, Boc_2_O (1.1 equiv) was added dropwise and the reaction mixture was stirred for 1–2 h at rt. After completion according to TLC, the reaction mixture was filtered through a silica pad, the pad was washed with EtOAc and the filtrate was evaporated under vacuum. The corresponding crude was subjected to flash column chromatography eluting with CH_2_Cl_2_/MeOH (99.7:0.3–98:2).

#### General Procedure for Swern Oxidation

To a solution of oxalyl chloride (1.5 equiv) in dry CH_2_Cl_2_ (0.3 M) at −78 °C was added DMSO (2 equiv) in CH_2_Cl_2_. After 15 min, *N*-Boc protected amino-alcohol (1 equiv) in CH_2_Cl_2_ was added. After 30 min, Et_3_N was added (5 equiv) and the mixture was allowed to warm up to rt. The organic phase was washed with a freshly prepared citric acid solution (5% w/w), then an aq. sat. NaHCO_3_ solution and brine, before being dried over Na_2_SO_4_, filtered and evaporated. The corresponding residue was pure enough to be used without further purification.

#### General Procedure for the Second Reductive Amination on Carbasugar Derivatives

To a stirred solution of secondary amine **si** **48** (1.1 equiv) and the corresponding aldehyde (1 equiv) in dry DCE (0.12 M based on aldehyde) was added AcOH (1.1 equiv). The solution was stirred for 4 h at rt, then NaBH­(OAc)_3_ (2.6 equiv) was added and the mixture was stirred for 4 h at rt and 12 h at 70 °C. After completion, the reaction was quenched by the addition of a 5% aq. NaHCO_3_ solution and the phases were separated. The aqueous phase was then extracted three times with CH_2_Cl_2_ and the combined organic phases once with brine. Drying over Na_2_SO_4_, filtration and evaporation afforded the crude product that was subjected to silica gel column chromatography eluting with CH_2_Cl_2_/MeOH (mostly 99.5:0.5–94:6) to afford the tertiary amines as yellow oils.

### Analytical HPLC Methods

**Method A:** Isera Sphere-Image 5 SCX 250 × 4.6 mm column and eluent A was a mixture of 10 mM NH_4_^+^HCO_2_^–^ and 15% CH_3_CN at pH 2.6 and eluent B was a mixture of 1 M NH_4_^+^HCO_2_^–^ and 15% CH_3_CN at pH 4.3. Linear gradient conditions were as follows: 0–5 min: 100:0→70:30 (A/B); 5–6 min: 70:30→0:100 (A/B); 6–10 min: 0:100→100:0 (A/B); 10–20 min: 100:0 (A/B) with a flow rate of 1.5 mL·min^–1^. **Method B:** Phenomenex Kinetex 5u XB-C18 100 Å 250 × 4.6 mm column and eluent A was H_2_O containing 0.05% formic acid (FA) and eluent B was CH_3_CN containing 0.05% FA. Linear gradient conditions were as follows: 0–1 min: 100:0 (A/B); 1–9 min: 100:0→60:40 (A/B); 9–11 min: 60:40→5:95 (A/B); 11–13 min: 5:95 (A/B); 13–14 min: 5:95→100:0 (A/B); 14–16 min: 100:0 (A/B) with a flow rate of 0.95 mL·min^–1^. **Method C:** Phenomenex Kinetex 5u XB-C18 100 Å 250 × 4.6 mm column and eluent A was H_2_O containing 0.05% trifluoroacetic acid (TFA) and eluent B was CH_3_CN containing 0.05% TFA. Linear gradient conditions were as follows: 0–1 min: 100:0 (A/B); 1–9 min: 100:0→60:40 (A/B); 9–11 min: 60:40→5:95 (A/B); 11–13 min: 5:95 (A/B); 13–14 min: 5:95→100:0 (A/B); 14–16 min: 100:0 (A/B) with a flow rate of 0.95 mL·min^–1^. **Method D**: XBridge Shield RP18 5 μm XB-C18 100 Å 150 × 4.6 mm column and eluent A was H_2_O containing 0.05% trifluoracetic acid (TFA) and eluent B was CH_3_CN containing 0.05% TFA. Linear gradient conditions were as follows: 0–9 min: 100:0 (A/B); 9–11 min: 100:0→0:100 (A/B); 11–13 min: 0:100; (A/B); 13–14 min: 0:100→100:0 (A/B); 14–16 min: 100:0 (A/B) with a flow rate of 1.00 mL·min^–1^. **Method E:** XBridge Shield RP18 5 μm XB-C18 100 Å 150 × 4.6 mm column and eluent A was H_2_O containing 0.05% trifluoroacetic acid (TFA) and eluent B was CH_3_CN containing 0.05% TFA. Linear gradient conditions were as follows: 0–4 min: 90:10 (A/B); 4–19 min: 90:0→0:100 (A/B); 19–21 min: 0:100 (A/B); 21–21.5 min: 0:100→90:10 (A/B); 21.5–25 min: 90:10 (A/B) with a flow rate of 1.00 mL·min^–1^. **Method F:** XBridge Shield RP18 5 μm XB-C18 100 Å 150 × 4.6 mm column and eluent A was H_2_O containing 0.01% ammonia (NH_3_) and eluent B was CH_3_CN containing 0.05% trifluoroacetic acid (TFA). Linear gradient conditions were as follows: 0–4 min: 90:10 (A/B); 4–19 min: 90:0→0:100 (A/B); 19–21 min: 0:100 (A/B); 21–21.5 min: 0:100→90:10 (A/B); 21.5–25 min: 90:10 (A/B) with a flow rate of 1.00 mL·min^–1^. **Method G:** Phenomenex Kinetex 5 μm XB-C18 100 Å 250 × 4.6 mm column and eluent A was H_2_O containing 0.05% trifluoracetic acid (TFA) and eluent B was CH_3_CN containing 0.05% TFA. Linear gradient conditions were as follows: 0–4 min: 85:15 (A/B); 4–29 min: 85:15→0:100 (A/B); 29–31 min: 0:100; (A/B); 31–31.5 min: 0:100→10:90 (A/B); 31.5–40 min: 10:90 (A/B) with a flow rate of 1.00 mL·min^–1^. Method S1: Agilent LC/MSD 1200 Series (Column: ODS 2000 (50 × 4.6 mm, 5 μm), *T* = 40 °C, eluent A was H_2_O containing 0.1% trifluoracetic acid (TFA) and eluent B was CH_3_CN. Linear gradient conditions were as follows: 0–0.5 min: 100:0 (A/B); 0.5–4.5 min: 100:0→95:5 (A/B); 4.5–5 min: 95:5→5:95; (A/B); 5–6 min: 5:95 (A/B); 6–6.1 min: 5:95→100:0 (A/B); 6.1–6.5 min: 100:0 (A/B) with a flow rate of 1.50 mL·min^–1^. **Method S2:** Agilent LC/MSD 1200 Series (Column: ODS 2000 (50 × 4.6 mm, 5 μm), *T* = 40 °C, eluent A was H_2_O containing 0.1% trifluoracetic acid (TFA) and eluent B was CH_3_CN. Linear gradient conditions were as follows: 0–0.5 min: 100:0 (A/B); 0.5–4.5 min: 100:0→70:30 (A/B); 4.5–5 min: 70:30→5:95; (A/B); 5–6 min: 5:95 (A/B); 6–6.1 min: 5:95→100:0 (A/B); 6.1–6.5 min: 100:0 (A/B) with a flow rate of 1.50 mL·min^–1^

### Preparative HPLC Methods

**Method H:** XBridge Prep Shield RP 18 5 μm OBDTM 19 × 150 mm column and eluent A was H_2_O containing 0.05% trifluoracetic acid (TFA) and eluent B was CH_3_CN. Linear gradient conditions were as follows: 0–10 min: 100:0 (A/B); 10–14 min: 100:0→60:40 (A/B); 14–15 min: 60:40→5:95; (A/B); 15–17 min: 5:95 (A/B); 17–20 min: 5:95→100:0 (A/B) with a flow rate of 20.20 mL·min^–1^. **Method I:** XBridge Prep Shield RP 18 5 μm OBDTM 19 × 150 mm column and eluent A was H_2_O containing 0.05% trifluoracetic acid (TFA) and eluent B was CH_3_CN. Linear gradient conditions were as follows: 0–9 min: 100:0 (A/B); 9–11 min: 100:0→5:95 (A/B); 11–13 min: 5:95; (A/B); 13–14 min: 5:95→100:0 (A/B); 14–20 min: 100:0 (A/B) with a flow rate of 17.00 mL·min^–1^. **Method J:** Phenomenex Kinetex 5u XB-C18 100 Å 250 × 21.2 mm column and eluent A was H_2_O containing 0.05% trifluoracetic acid (TFA) and eluent B was CH_3_CN. Linear gradient conditions were as follows: 0–10 min: 100:0 (A/B); 10–14 min: 100:0→60:40 (A/B); 14–15 min: 60:40→5:95 (A/B); 15–17 min: 5:95 (A/B); 17–20 min: 5:95→100:0 (A/B) with a flow rate of 17.00 mL·min^–1^. **Method K:** Phenomenex Kinetex 5u XB-C18 100 Å 250 × 21.2 mm column and eluent A was H_2_O containing 0.05% trifluoracetic acid (TFA) and eluent B was CH_3_CN. Linear gradient conditions were as follows: 0–1 min: 100:0 (A/B); 1–9 min: 100:0→60:40 (A/B); 9–11 min: 60:40→5:95 (A/B); 11–13 min: 5:95 (A/B); 13–14 min: 5:95→100:0 (A/B); 14–20 min: 100:0 (A/B) with a flow rate of 20.20 mL·min^–1^. **Method L:** XBridge Prep Shield RP 18 5 μm OBDTM 19 × 150 mm column and eluent A was H_2_O containing 0.01% ammonia (NH_3_) and eluent B was CH_3_CN. Linear gradient conditions were as follows: 0–4 min: 90:10 (A/B); 4–7 min: 90:10→75:25 (A/B); 7–9 min: 75:25→0:100; (A/B); 9–10 min: 0:100 (A/B); 10–11 min: 100:0→90:10 (A/B); 11–12 min: 90:10 (A/B) with a flow rate of 17.10 mL·min^–1^. **Method M:** XBridge Prep Shield RP 18 5 μm OBDTM 19 × 150 mm column and eluent A was H_2_O containing 0.01% ammonia (NH_3_) and eluent B was CH_3_CN. Linear gradient conditions were as follows: 0–4 min: 90:10 (A/B); 4–7 min: 90:10→70:30 (A/B); 7–8 min: 70:30→0:100; (A/B); 8–9 min: 0:100 (A/B); 9–10 min: 100:0→90:10 (A/B); 10–11 min: 90:10 (A/B) with a flow rate of 17.10 mL·min^–1^.

#### ((3aR,4R,6R,6aR)-6-(6-Amino-9*H*-purin-9-yl)-2,2-dimethyltetrahydrofuro­[3,4-*d*]­[1,3]­dioxol-4-yl)­methanol (**si1**)

To a solution of adenosine (10.0 g, 37.4 mmol, 1 equiv) in acetone (1 L), was added CH­(OEt)_3_ (31.1 mL, 187 mmol, 5 equiv) and *p*TsOH (35.6 g, 187 mmol, 5 equiv). The mixture was stirred at rt for 16 h, then quenched with aqueous saturated NaHCO_3_, concentrated until a precipitate was obtained. The precipitate was dissolved in MeOH/CH_2_Cl_2_, filtered, and evaporated to give the product (10.5 g, 34.4 mmol, 92%) as a white solid. ^1^H NMR (500 MHz, DMSO-*d*_6_) δ 8.33 (s, 1H), 8.15 (s, 1H), 7.33 (s, 2H), 6.11 (d, *J* = 3.1 Hz, 1H), 5.33 (dd, *J* = 6.2, 3.1 Hz, 1H), 5.29 (d, *J* = 21.9 Hz, 1H), 4.96 (dd, *J* = 6.2, 2.5 Hz, 1H), 4.20 (td, *J* = 4.8, 2.5 Hz, 1H), 3.59 – 3.48 (m, 2H), 1.53 (d, *J* = 0.7 Hz, 3H), 1.31 (d, *J* = 0.8 Hz, 3H). ^13^C NMR (101 MHz, DMSO) δ 156.1, 152.5, 148.8, 139.6, 119.0, 113.0, 89.5, 86.3, 83.2, 81.3, 61.5, 27.0, 25.1. R_f_: 0.31 (4% MeOH in CH_2_Cl_2;_ + 2% NH_3_ 7 M in MeOH). HR-MS (ESI): calcd. for C_13_H_17_N_5_O_4_ [M + H]^+^: 308.13 found: 308.13.

#### 9-((3aR,4R,6R,6aR)-6-(Azidomethyl)-2,2-dimethyltetrahydrofuro­[3,4-*d*]­[1,3]­dioxol-4-yl)-9*H*-purin-6-amine (**si2**)

To a solution of ((3a*R*,4*R*,6*R*,6a*R*)-6-(6-amino-9*H*-purin-9-yl)-2,2-dimethyltetrahydro-furo­[3,4-*d*]­[1,3]­dioxol-4-yl)­methanol (6.50 g, 21.2 mmol, 1 equiv) in dioxane (70 mL) at 0 °C were added DPPA (9.12 mL, 42.3 mmol, 2 equiv) and DBU (9.50 mL, 63.5 mmol, 3 equiv). The mixture was stirred at rt for 16 h. NaN_3_ (6.88 g, 106 mmol, 5 equiv) and 15-crown-5 (4.66 g, 21.2 mmol, 1 equiv) were added and the mixture was stirred at 110 °C for 6 h. The organic phase was evaporated, water was added and the aqueous phase was extracted three times with EtOAc. The combined organics were washed with brine, dried over Na_2_SO_4_ and evaporated. The residue was purified on silica gel column eluting with 30–100% EtOAc in PE to afford the desired product (6.43 g, 19.3 mmol, 91%) as a light yellow solid. ^1^H NMR (400 MHz, CDCl_3_) δ 8.35 (s, 1H), 7.91 (s, 1H), 6.11 (d, *J* = 2.4 Hz, 1H), 6.06–5.91 (m, 2H), 5.46 (ddd, *J* = 6.4, 2.3, 0.4 Hz, 1H), 5.06 (ddd, *J* = 6.4, 3.5, 0.5 Hz, 1H), 4.45–4.31 (m, 1H), 3.66–3.45 (m, 2H), 1.61 (d, *J* = 0.7 Hz, 3H), 1.39 (d, *J* = 0.7 Hz, 3H). ^13^C NMR (101 MHz, CDCl_3_) δ 155.8, 153.2, 149.3, 140.0, 120.4, 114.8, 90.8, 85.7, 84.1, 82.2, 52.4, 27.2, 25.4. R_f_: 0.41 (95:5 CH_2_Cl_2_/MeOH). HR-MS (ESI): *m*/*z* [M + H]^+^ calcd. for C_13_H_17_N_8_O_3_: 333.13 found: 333.13.

#### *tert*-Butyl (9-((3aR,4R,6R,6aR)-6-(Azidomethyl)-2,2-dimethyltetrahydrofuro­[3,4-*d*]­[1,3]­dioxol-4-yl)-9*H*-purin-6-yl)­carbamate (**si3**)

To a solution of **si2** (2.47 g, 7.4 mmol, 1 equiv) in dry THF (C = 0.3 M, V = 25 mL) at 0 °C under argon was added portionwise 60% suspended NaH in oil (744 mg, 18 mmol, 2.5 equiv). After 45 min at room temperature, Boc_2_O was added (1.776 g, 8.14 mmol, 1.1 equiv) in 3 mL dry THF at 0 °C. After 1 h 30 min at room temperature, Boc_2_O (807 mg, 3.7 mmol, 0.5 equiv) was added at 0 °C and the reaction was stirred at room temperature for 1 h 30 min. After that, reaction was quenched with NaCl at 0 °C, extracted with EtOAc. The combined organics were washed with brine, dried over Na_2_SO_4_ and evaporated. The residue was purified on silica gel column eluting with 10–100% EtOAc in cyclohexane to afford the desired product (661 mg, 1.53 mmol, 80%) as a light yellow solid. ^1^H NMR (400 MHz, CDCl_3_) δ 8.77 (s, 2H), 8.09 (s, 2H), 6.15 (d, *J* = 2.4 Hz, 1H), 5.46 (dd, *J* = 6.4, 2.4 Hz, 1H), 5.05 (dd, *J* = 6.5, 3.6 Hz, 2H), 3.58 (d, *J* = 5.5 Hz, 2H), 1.63 (d, *J* = 0.7 Hz, 3H), 1.54 (s, 9H), 1.39 (d, *J* = 0.7 Hz, 3H). R_f_: 0.76 (Ethyl Acetate). HR-MS (ESI): *m*/*z* [M + H]^+^ calcd. for C_18_H_25_N_8_O_5_: 433.19 found: 433.19.

#### *tert*-Butyl (9-((3aR,4R,6R,6aR)-6-(Aminomethyl)-2,2-dimethyltetrahydrofuro­[3,4-*d*]­[1,3]­dioxol-4-yl)-9H-purin-6-yl)­carbamate (**si4**)

To a solution of **si3** (2.56 g, 5.9 mmol, 1 eq) in EtOAc/MeOH (1:1, 19 mL) was added Pd/C (256 mg, 10% w/w). The suspension was put under H_2_ and stirred for 16 h at room temperature. The solution was then filtrated on Celite and evaporated to afford the desired product as a gray foam (2.39 g, quant). ^1^H NMR (300 MHz, CDCl_3_) δ 8.75 (s, 1H), 8.06 (s, 1H), 7.96 (d, *J* = 12.3 Hz, 1H), 6.07 (d, *J* = 3.0 Hz, 1H), 5.47 (dd, *J* = 6.5, 3.1 Hz, 1H), 5.02 (dd, *J* = 6.5, 3.5 Hz, 1H), 4.27 (ddd, *J* = 6.0, 4.5, 3.5 Hz, 1H), 3.10 – 2.88 (m, 2H), 1.63 (d, *J* = 0.7 Hz, 3H), 1.57 (s, 9H), 1.39 (d, *J* = 0.7 Hz, 3H). R_f_: 0.42 (95:5 CH_2_Cl_2_/MeOH + 1% Et_3_N). HR-MS (ESI): *m*/*z* [M + H]^+^ calcd. for C_18_H_27_N_6_O_5_: 407.20 found: 407.20.

#### *tert*-Butyl (*tert*-Butoxycarbonyl)-l-homoserinate (**si5**)

To a solution of (*S*)-4-(*tert*-butoxy)-3-((*tert*-butoxycarbonyl)­amino)-4-oxobutanoic acid (500 mg, 1.73 mmol, 1 equiv) and Et_3_N (265 mL, 1.90 mmol, 1.1 equiv) in dry THF (6.9 mL) was added isobutyl chloroformate (247 mL, 1.90 mmol, 1.1 equiv) at −5 °C. The mixture was stirred for 30 min, then filtered. The solution containing (*S*)-(*S*)-4-(*tert*-butoxy)-3-((*tert*-butoxycarbonyl)­amino)-4-oxobutanoic (ethyl carbonic) anhydride was recovered, cooled at 0 °C, MeOH (6.9 mL) and NaBH_4_ (131 mg, 3.46 mmol, 2 equiv) were added portion wise. The solution was then stirred at rt for 2 h. The reaction was quenched with diluted ammonium chloride, extract three times with EtOAc. The combined organics were washed with brine, dried over Na_2_SO_4_ and evaporated. The residue was purified on silica gel column eluting with 20–100% EtOAc in cyclohexane to afford the desired product (345 mg, 0.77 mmol, 45%) as a colorless oil. ^1^H NMR (400 MHz, CDCl_3_) δ 5.33 (d, *J* = 7.8 Hz, 1H), 4.42–4.28 (m, 1H), 3.80–3.57 (m, 2H), 3.44 (br s, 1H), 2.12 (ddt, *J* = 17.7, 12.8, 7.0 Hz, 1H), 1.69–1.49 (m, 1H), 1.47 (s, 9H), 1.45 (s, 9H). ^13^C NMR (101 MHz, CDCl_3_) δ 172.1, 156.7, 82.4, 80.4, 58.4, 51.1, 36.7, 28.4, 28.1. R_f_: 0.26 (67:33 cyclohexane/EtOAc). HR-MS (ESI): *m*/*z* [M + H]^+^ calcd. for C_13_H_26_NO_5_: 276.17 found: 276.18.

#### *tert*-Butyl (s)-2-((*tert*-butoxycarbonyl)­amino)-4-oxobutanoate (**si6**)

To a solution of oxalyl chloride (1.17 mL, 13.6 mmol, 1.5 equiv) in dry CH_2_Cl_2_ (30 mL) at −78 °C was added DMSO (2.15 mL, 18.2 mmol, 2 equiv) in CH_2_Cl_2_ (5 mL). After 15 min, *tert*-butyl (*tert*-butoxycarbonyl)-*L*-homoserinate (2.50 g, 9.08 mmol, 1 equiv) in CH_2_Cl_2_ (10 mL) was added. After 30 min, Et_3_N was added (6.30 mL, 45.4 mmol, 5 equiv) and the mixture was allowed to return at rt. The organic phase was washed with a solution of citric acid (5% w/w), then aqueous saturated NaHCO_3_, brine, dried over Na_2_SO_4_, evaporated. The residue was purified on silica gel column (10–100% EtOAc in cyclohexane) to afford the desired product (1.96 g, 79%) as a colorless oil. ^1^H NMR (400 MHz, CDCl_3_) 9.75 (s, 1H), 5.35 (m, 1H), 4.42 (m, 1H), 2.84–3.06 (m, 2H), 1.4 (s, 18H). ^13^C NMR (101 MHz, CDCl_3_) δ 199.3, 170.0, 155.4, 82.7, 80.1, 49.5, 46.4, 28.4, 27.9. R_f_: 0.44 (76:33 cyclohexane/EtOAc). HR-MS (ESI): *m*/*z* [M + H]^+^ calc. for C_13_H_24_NO_5_: 274.16 found: 274.16.

#### *tert*-Butyl (s)-2-((*tert*-butoxycarbonyl)­amino)-4-((((3aR,4R,6R,6aR)-6-(6-((*tert*-butoxycarbonyl)­amino)-9*H*-purin-9-yl)-2,2-dimethyltetrahydrofuro­[3,4-*d*]­[1,3]­dioxol-4-yl)­methyl)­amino)­butanoate (**si7**)

The synthesis followed the general procedure for the first reductive amination. Yield: 2.4 g, 3.6 mmol, 43%. C_31_H_49_N_7_O_9_ (663.77 g/mol). ^1^H NMR (400 MHz; DMSO-*d*_6_): δ 10.14 (bs, 1H, N*H* carbamate nucleobase), 8.62 (s, 1H, H2), 8.61 (s, 1H, H8), 7.15 (d, ^3^ *J* = 8.0 Hz, 1H, N*H* carbamate), 6.17 (d, ^3^ *J* = 2.7 Hz, 1H, H1’), 5.47 (dd, ^3^ *J* = 6.0 Hz, 2.7 Hz, 1H, H2’), 4.99 (dd, ^3^ *J* = 6.0 Hz and ^3^ *J* = 2.7 Hz, 1H, H3′), 4.22 (td, ^3^ *J* = 5.6 Hz and ^3^ *J* = 2.8 Hz, 1H, H4’), 3.94–3.87 (m, 1H, Hα), 2.73 (dd, ^2^ *J* = 12.4 Hz and ^3^ *J* = 5.8 Hz, 1H, H5_A_’), 2.61 (dd, ^2^ *J* = 12.4 Hz and ^3^ *J* = 5.8 Hz, 1H, H5_B_’), 2.56–2.45 (m, 2H, Hγ), 1.75–1.71 (m, 1H, Hβ_A_), 1.66–1.60 (m, 1H, Hβ_B_), 1.55 (s, 3H, C*H*_3_), 1.47 (s, 9H, C*H*_3_ *t*Bu), 1.41–1.29 (m, 22H, C*H*_3_, 2 × C*H*_3_ *t*Bu); HR-MS (ESI): calcd. for C_31_H_50_N_7_O_9_ [M + H]^+^: 664.3665, found: 664.3661; R*_f_*: 0.58 (CH_2_Cl_2_/MeOH 9:1).

#### *tert*-Butyl (9-((3aR,4R,6R,6aR)-6-(((3-((*tert*-butoxycarbonyl)­amino)-4,4-difluorobutyl) amino)­methyl)-2,2-dimethyltetrahydrofuro­[3,4-*d*]­[1,3]­dioxol-4-yl)-9*H*-Purin-6-yl)­carbamate (**si15a**)

The synthesis followed the general procedure for the first reductive amination reductive amination. Yield: 293.5 mg, 0.478 mmol, 43%. C_27_H_41_N_7_O_7_F_2_ (613.7 g/mol). ^1^H NMR (400 MHz; DMSO-*d*_6_): δ 10.16 (ds, 1H, N*H* carbamate nucleobase), 8.63 (s, 1H, H2), 8.60 (s, 1H, H8), 7.08 (d, ^3^ *J* = 9.2 Hz, 1H, N*H* carbamate), 6.18 (t, ^3^ *J* = 2.8 Hz, 1H, H1’), 5.88 (tt, ^3^ *J* = 56.0 Hz and ^3^ *J* = 3.6 Hz, 1H, C*H*F_2_), 5.51–5.44 (m, 1H, H2’), 4.99 (dd, ^3^ *J* = 6.4 Hz and ^3^ *J* = 2.8 Hz, 1H, H3′), 4.26–4.18 (m, 1H, H4’), 3.84–3.82 (m, 1H, Hα), 2.75–2.62 (m, 2H, H5′), 2.55–2.43 (m, 2H, Hγ), 1.63–1.59 (m, 1H, Hβ_A_), 1.54–1.33 (m, 25H, Hβ_B_, 2 × C*H*_3_, 2 × C*H*_3_ *t*Bu); ^13^C NMR (101 MHz, DMSO-*d*_6_) δ 155.5, 151.6, 151.1, 151.0, 150.1, 142.8, 123.9, 113.2, 113.1, 89.5, 89.4, 85.3, 85.2, 82.7, 82.7, 82.2, 82.1, 80.1, 78.1, 50.9, 45.2, 28.0, 27.8, 27.0, 26.9, 25.2, 25.1; HR-MS (ESI): calcd. for C_27_H_42_N_7_O_7_F_3_ [M + H]^+^: 614.3108, found: 614.3109; R*_f_*: 0.27 (CH_2_Cl_2_/MeOH 9:1).

#### *tert*-Butyl (9-((3aR,4R,6R,6aR)-6-(((3-((*tert*-butoxycarbonyl)­amino)-4,4,4-trifluorobutyl) amino)­methyl)-2,2-dimethyltetrahydrofuro­[3,4-*d*]­[1,3]­dioxol-4-yl)-9*H*-purin-6-yl) carbamate (**si15b**)

The synthesis followed the general procedure for the first reductive amination. Yield: 311.8 mg, 0.494 mmol, 45%. C_27_H_40_N_7_O_7_F_3_ (631.7 g/mol). ^1^H NMR (400 MHz; DMSO-*d*_6_): δ 10.16 (bs, 1H, N*H* carbamate nucleobase), 8.62 (s, 1H, H2), 8.59 (s, 1H, H8), 7.45 (d, ^3^ *J* = 9.2 Hz, 1H, N*H* carbamate), 6.20–6.16 (m, 1H, H1’), 5.52–5.42 (m, 1H, H2’), 5.00–4.98 (m, 1H, H3′), 4.30–4.19 (m, 2H, H4’, Hα), 2.78–2.61 (m, 2H, H5′), 2.57–2.43 (m, 2H, Hγ), 1.70–1.66 (m, 1H, Hβ_A_), 1.63–1.60 (m, 1H, Hβ_B_), 1.54 (s, 3H, C*H*_3_), 1.47 (s, 9H, C*H*_3_ *t*Bu), 1.41–1.27 (m, 12H, C*H*_3_, C*H*_3_ *t*Bu); ^13^C NMR (101 MHz, DMSO-*d*_6_) δ 155.4, 151.5, 151.1, 151.0, 150.2, 142.8, 123.9, 113.2, 113.1 (dia), 89.5, 89.4 (dia), 85.1, 82.7, 82.6 (dia), 82.2, 82.0 (dia), 80.1, 78.6, 50.9, 50.8 (dia), 45.0, 44.6 (dia), 28.0, 27.8, 27.0, 26.9 (dia), 25.2, 25.1 (dia); HR-MS (ESI): calcd. for C_27_H_41_N_7_O_7_F_3_ [M + H]^+^: 632.3014, found: 632.3010; R*_f_*: 0.64 (CH_2_Cl_2_/MeOH 9:1).

#### *tert*-Butyl (s)-4-((2-((*tert*-butoxycarbonyl)­(methyl)­amino)­ethyl)­(((3aR,4R,6R,6aR)-6-(6-((tert-butoxycarbonyl)­amino)-9*H*-purin-9-yl)-2,2-dimethyltetrahydrofuro­[3,4-*d*]­[1,3]­dioxol-4-yl)­methyl)­amino)-2-((tert-butoxycarbonyl)­amino)­butanoate (**si8a**)

Reductive amination: for **si8a** started from the aldehyde (*tert*-butyl methyl­(2-oxoethyl)­carbamate) and followed the general procedure for the second reductive amination. Yield: 49.7 mg, 0.061 mmol, 22%. HR-MS (ESI): calcd. for C_39_H_65_N_8_O_11_ [M + H]^+^: 821.4767, found: 821.4764; R*_f_*: 0.83 (CH_2_Cl_2_/MeOH 9:1).

#### (S)-2-Amino-4-((((2R,3S,4R,5R)-5-(6-amino-9*H*-purin-9-yl)-3,4-dihydroxytetrahydro-furan-2-yl)­methyl)­(2-(methylamino)­ethyl)­amino)­butanoic acid (**3d**)

Deprotection of **si8a** followed the general procedure for deprotection. Yield: 56.7 mg, 0.074 mmol, 100% (3 TFA salt). ^1^H NMR (400 MHz; DMSO-*d*_6_): δ 8.88 (bs, 3H, N*H*_3_^+^), 8.61 (s, 1H, H2), 8.54–8.22 (m, 6H, H8, N*H*_3_^+^, N*H*_2_^+^), 5.96 (d, ^3^ *J* = 4.9 Hz, 1H, H1’), 4.57 (t, ^3^ *J* = 4.9 Hz, 1H, H2’), 4.19 (q, ^3^ *J* = 5.5 Hz, 1H, H4’), 4.11 (t, ^3^ *J* = 4.9 Hz, 1H, H3′), 3.99 (t, ^3^ *J* = 5.8 Hz, 1H, Hα), 3.12 (s, 2H, H5′), 3.06 (s, 2H, H2’’), 2.95 (s, 2H, H1’’), 2.90 (s, 2H, Hγ), 2.50 (s, 3H, C*H*_3_), 2.13–2.01 (m, 1H, Hβ_A_), 2.00–1.88 (m, 1H, Hβ_B_); HR-MS (ESI): calcd. for C_17_H_29_N_8_O_5_ [M + H]^+^: 425.2255, found: 425.2255; HPLC: *t*_R_ = 8.810 min (Method A), UV-purity at 254 nm: 95.7%.

#### *tert*-Butyl (S)-2-((*tert*-butoxycarbonyl)­amino)-4-((((3aR,4R,6R,6aR)-6-(6-((*tert*-butoxy-carbonyl)­amino)-9*H*-purin-9-yl)-2,2-dimethyltetrahydrofuro­[3,4-*d*]­[1,3]­dioxol-4-yl)­methyl)­((R)-3-((*tert*-butoxycarbonyl)­amino)­butyl)­amino)­butanoate (**si8b**)

Reductive amination for **si8b** started from aldehyde **(** *tert*-butyl (*R*)-(4-oxobutan-2-yl)­carbamate) and followed the general procedure for the second reductive amination. Yield: 79 mg, 0.095 mmol, 35%. HR-MS (ESI): calcd. for C_40_H_67_N_8_O_11_ [M + H]^+^: 835.4924, found: 835.4920; R*_f_*: 0.72 (CH_2_Cl_2_/MeOH 9:1).

#### (S)-2-Amino-4-((((2R,3S,4R,5R)-5-(6-amino-9*H*-purin-9-yl)-3,4-dihydroxytetrahydro-furan-2-yl)­methyl)­((R)­3-aminobutyl)­amino)­butanoic acid (**2c**)

Deprotection of **si8b** followed the general procedure for deprotection. Yield: 80 mg, 0.102 mmol, 100% (3 TFA salt). ^1^H NMR (400 MHz; DMSO-*d*_6_): δ 8.62–8.49 (m, 7H, H2, 2 × N*H*_3_^+^), 8.40 (s, 1H, H8), 8.02 (m, 3H, N*H*_3_^+^), 6.02 (d, ^3^ *J* = 4.8 Hz, 1H, H1’), 4.64 (t, ^3^ *J* = 4.8 Hz, 1H, H2’), 4.43–4.32 (m, 1H, H4’), 4.22 (t, ^3^ *J* = 4.8 Hz, 1H, H3′), 4.04–4.01 (m, 1H, Hα), 3.68–3.62 (m, 1H, H5′_A_), 3.55–3.51 (m, 1H, H5′_B_), 3.34–3.19 (m, 5H, H1’’, H3′’, Hγ), 2.31–2.18 (m, 1H, Hβ_A_), 2.18–2.05 (m, 1H, Hβ_B_), 1.98–1.80 (m, 2H, H2’’_A and B_), 1.14 (d, ^3^ *J* = 6.4 Hz, 3H, C*H*_3_); HR-MS (ESI): calcd. for C_18_H_31_N_8_O_5_ [M + H]^+^: 439.2412, found: 439.2413; HPLC: *t*_R_ = 2.237 min (Method B), UV-purity at 254 nm: 93.2%.

#### *tert*-Butyl (2S)-2-((*tert*-butoxycarbonyl)­amino)-4-((((3aR,4R,6R,6aR)-6-(6-((*tert*-butoxycarbonyl)­amino)-9*H*-purin-9-yl)-2,2-dimethyltetrahydrofuro­[3,4-*d*]­[1,3]­dioxol-4-yl)­methyl)­(3-((*tert*-butoxycarbonyl)­amino)­hexyl)­amino)­butanoate (**si8c**)

Reductive amination for **si8c** started from the aldehyde (*tert*-butyl (1-oxohexan-3-yl)­carbamate) and followed the general procedure for the second reductive amination. Yield: 61.2 mg, 0.071 mmol, 35%. HR-MS (ESI): calcd. for C_42_H_71_N_8_O_11_ [M + H]^+^: 863.5237, found: 863.5237.

#### (2-S)-2-Amino-4-((((2R,3S,4R,5R)-5-(6-amino-9*H*-purin-9-yl)-3,4-dihydroxytetrahydrofuran-2-yl)­methyl)­(3-aminohexyl)­amino)­butanoic acid (**2a**)

Deprotection (**9c**): yield: 60.5 mg, 0.075 mmol, 100% (3 TFA salt). ^1^H NMR (400 MHz; DMSO-*d*_6_): δ 8.78–8.30 (m, 8H, H2, H8, 2 x N*H*_3_^+^), 7.95 (bs, 3H, N*H*_3_^+^), 6.01 (d, *J* = 4.8 Hz, 1H, H1’), 4.66–4.60 (m, 1H, H2’), 4.37–4.34 (m, 1H, H4’), 4.26–4.20 (m, 1H, H3′), 4.02 (t, *J* = 5.4 Hz, 1H, Hα), 3.70–3.61 (m, 1H, H5′_A_), 3.54–3.50 (m, 1H, H5′_B_), 3.32–3.08 (m, 5H, Hγ, H1’’, H3′’), 2.30–2.19 (m, 1H, Hβ_A_), 2.15–2.06 (m, 1H, Hβ_B_), 1.93–1.80 (m, 2H, H2’’), 1.43–1.36 (m, 2H, H4’’), 1.24–1.16 (m, 2H, H5′’), 0.87–0.72 (m, 3H, H6’’); HR-MS (ESI): calcd. for C_20_H_35_N_8_O_5_ [M + H]^+^: 467.2725, found: 467.2725; HPLC: *t*_R_ = 9.237 min (Method A), UV-purity at 254 nm: 93.4%.

#### *tert*-Butyl (S)-2-((*tert*-butoxycarbonyl)­amino)-4-((((3aR,4R,6R,6aR)-6-(6-((*tert*-butoxycarbonyl)­amino)-9*H*-purin-9-yl)-2,2-dimethyltetrahydrofuro­[3,4-*d*]­[1,3]­dioxol-4-yl)­methyl)­(3-((*tert*-butoxycarbonyl)­amino)­propyl)­amino)­butanoate (**si8d**)

Reductive amination for **si8e** started from aldehyde (*tert*-butyl (3-oxopropyl)­carbamate) and followed the general procedure for the second reductive amination. Yield: 89.6 mg, 0.109 mmol, 40%. HR-MS (ESI): calcd. for C_39_H_65_N_8_O_11_ [M + H]^+^: 821.4767, found: 821.4766; R*_f_*: 0.72 (CH_2_Cl_2_/MeOH 9:1).

#### (S)-2-Amino-4-((((2R,3S,4R,5R)-5-(6-amino-9*H*-purin-9-yl)-3,4-dihydroxytetrahydrofuran-2-yl)­methyl)­(3-aminopropyl)­amino)­butanoic acid (**2d**)

Deprotection (**9d**) of **si8d** followed the general procedure for deprotection. Yield: 75 mg, 0.098 mmol, 100% (3 TFA salt). ^1^H NMR (400 MHz; DMSO-*d*_6_): δ 8.52 (s, 1H, H2), 8.49–8.27 (m, 4H, H8, N*H*_3_^+^), 7.93 (bs, 3H, N*H*_3_^+^), 6.01 (d, ^3^ *J* = 4.8 Hz, 1H, H1’), 4.64 (t, ^3^ *J* = 4.8 Hz, 1H, H2’), 4.41–4.31 (m, 1H, H4’), 4.23 (t, ^3^ *J* = 4.8 Hz, 1H, H3′), 4.01 (t, ^3^ *J* = 6.0 Hz, 1H, Hα), 3.70–3.58 (m, 1H, H5′_A_), 3.53–3.47 (m, 1H, H5′_B_), 3.32–3.24 (m, 2H, Hγ), 3.23–3.12 (m, 2H, H1’’), 2.91–2.78 (m, 2H, H3′’), 2.30–2.17 (m, 1H, Hβ_A_), 2.15–2.04 (m, 1H, Hβ_B_), 1.97–1.84 (m, 2H, H2’’); HR-MS (ESI): calc. for C_17_H_29_N_8_O_5_ [M + H]^+^: 425.2255, found: 425.2255; Purified by preparative HPLC (Method I). HPLC: *t*_R_ = 1.684 min (Method D), UV-purity at 254 nm: 95.6%.

#### *tert*-Butyl (S)-4-((3-((*tert*-butoxycarbonyl)­(methyl)­amino)­propyl)­(((3aR,4R,6R,6aR)-6-(6-((*tert*-butoxycarbonyl)­amino)-9*H*-purin-9-yl)-2,2-dimethyltetrahydrofuro­[3,4-*d*]­[1,3]­dioxol-4-yl)­methyl)­amino)-2-((*tert*-butoxycarbonyl)­amino)­butanoate (**si8e**)

Reductive amination for **8e** started from aldehyde (*tert*-butyl methyl­(3-oxopropyl) carbamate) and followed the general procedure for the second reductive amination. Yield: 88 mg, 0.105 mmol, 39%. HR-MS (ESI): calcd. for C_40_H_67_N_8_O_11_ [M + H]^+^: 835.4924, found: 835.4921; R*_f_*: 0.75 (CH_2_Cl_2_/MeOH 9:1).

#### (S)-2-Amino-4-((((2R,3S,4R,5R)-5-(6-amino-9*H*-purin-9-yl)-3,4-dihydroxytetrahydro furan-2-yl)­methyl)­(3-(methylamino)­propyl)­amino)­butanoic acid (**3a**)

Deprotection of **si8e** followed the general procedure for deprotection. Yield: 82 mg, 0.105 mmol, 100% (3 TFA salt). ^1^H NMR (400 MHz; DMSO-*d*_6_): δ 8.67 (bs, 3H, N*H*_3_^+^), 8.54 (s, 1H, H2), 8.53–8.35 (m, 4H, H8, N*H*_3_^+^), 6.01 (d, ^3^ *J* = 4.8 Hz, 1H, H1’), 4.63 (t, ^3^ *J* = 4.8 Hz, 1H, H2’), 4.40–4.31 (m, 1H, H4’), 4.22 (t, ^3^ *J* = 4.8 Hz, 1H, H3′), 4.02 (t, ^3^ *J* = 6.0 Hz, 1H, Hα), 3.69–3.59 (m, 1H, H5′_A_), 3.56–3.47 (m, 1H, H5′_B_), 3.39–3.22 (m, 2H, Hγ), 3.22–3.13 (m, 2H, H1’’), 3.02–2.84 (m, 2H, H3′’), 2.57–2.52 (m, 3H, C*H*_3_), 2.27–2.19 (m, 1H, Hβ_A_), 2.13–2.06 (m, 1H, Hβ_B_), 2.01–1.87 (m, 2H, H2’’); HR-MS (ESI): calcd. for C_18_H_31_N_8_O_5_ [M + H]^+^: 439.2412, found: 439.2416; HPLC: *t*_R_ = 11.016 min (Method A), UV-purity at 254 nm: 96.8%.

#### *tert*-Butyl (S)-4-((4-((*tert*-butoxycarbonyl)­(methyl)­amino)­butyl)­(((3aR,4R,6R,6aR)-6-(6-((*tert*-butoxycarbonyl)­amino)-9*H*-purin-9-yl)-2,2-dimethyltetrahydrofuro­[3,4-*d*]­[1,3]­dioxol-4-yl)­methyl)­amino)-2-((*tert*-butoxycarbonyl)­amino)­butanoate (**si8f**)

Reductive amination for the compound **8f** started from aldehyde (*tert*-butyl methyl­(4-oxobutyl)­carbamate) and followed the general procedure for second reductive amination. Yield: 88.9 mg, 0.105 mmol, 39%. HR-MS (ESI): calcd. for C_41_H_69_N_8_O_11_ [M + H]^+^: 849.5080, found: 849.5077; R*_f_*: 0.82 (CH_2_Cl_2_/MeOH 9:1).

#### (S)-2-Amino-4-((((2R,3S,4R,5R)-5-(6-amino-9H-purin-9-yl)-3,4-dihydroxytetrahydro-furan-2-yl)­methyl)­(4-(methylamino)­butyl)­amino)­butanoic acid (**3e**)

Deprotection of **si8f** followed the general procedure for deprotection. Yield: 73.1 mg, 0.092 mmol, 100% (3 TFA salt). ^1^H NMR (400 MHz; DMSO-*d*_6_): δ 8.63 (bs, 3H, N*H*_2_^+^), 8.56 (s, 1H, H2), 8.48 (bs, 3H, N*H*_3_^+^), 8.38 (s, 1H, H8), 6.02 (dd, ^3^ *J* = 4.8, 2.0 Hz, 1H, H1’), 4.64 (t, ^3^ *J* = 4.8 Hz, 1H, H2’), 4.42–4.31 (m, 1H, H4’), 4.22 (t, ^3^ *J* = 4.8 Hz, 1H, H3′), 4.07–3.98 (m, 1H, Hα), 3.72–3.60 (m, 1H, H5′_A_), 3.59–3.49 (m, 1H, H5′_B_), 3.39–3.30 (m, 1H, Hγ_A_), 3.30–3.19 (m, 1H, Hγ_B_), 3.19–3.06 (m, 2H, H1’’), 2.86–2.75 (m, 2H, H4’’), 2.55–2.50 (m, 3H, C*H*_3_), 2.29–2.17 (m, 1H, Hβ_A_), 2.17–2.04 (m, 1H, Hβ_B_), 1.64–1.57 (m, 2H, H2’’), 1.57–1.51 (m, 2H, H3′’); HR-MS (ESI): calcd. for C_19_H_33_N_8_O_5_ [M + H]^+^: 453.2568, found: 453.2569. Purified by preparative HPLC (Method L). HPLC: *t*_R_ = 9.307 min (Method F), UV purity at 254 nnm: 95.9%.

#### *tert*-Butyl (S)-4-((3-(Benzyl­(*tert*-butoxycarbonyl)­amino)­propyl)­(((3aR,4R,6R,6aR)-6-(6-((*tert*-butoxycarbonyl)­amino)-9*H*-purin-9-yl)-2,2-dimethyltetrahydrofuro­[3,4-*d*]­[1,3]­dioxol-4-yl)­methyl)­amino)-2-((*tert*-butoxycarbonyl)­amino)­butanoate (**si8g**)

Reductive amination for **si8g** started from aldehyde **si13c** and followed the general procedure for the second reductive amination. Yield: 27 mg, 0.030 mmol, 21%. HR-MS (ESI): calcd. for C_46_H_71_N_8_O_11_ [M + H]^+^: 911.5237, found: 911.5222; R*_f_*: 0.8 (CH_2_Cl_2_/MeOH 9:1).

#### (S)-2-Amino-4-((((2R,3S,4R,5R)-5-(6-amino-9H-purin-9-yl)-3,4-dihydroxytetrahydro furan-2-yl)­methyl)­(3-(benzylamino)­propyl)­amino)­butanoic acid (**5a**)

Deprotection of si8g followed the general procedure for the second reductive amination. Yield: 18 mg, 0.024 mmol, 100% (2 TFA salt). ^1^H NMR (400 MHz; DMSO-*d*_6_): δ 9.06 (s, 2H, N*H*_2_), 8.52 (s, 1H, H2), 8.45–8.25 (m, 4H, H8, N*H*_3_^+^), 7.50–7.36 (m, 5H, *H*-Ph), 6.01 (d, ^3^ *J* = 4.8 Hz, 1H, H1’), 4.63 (t, ^3^ *J* = 4.8 Hz, 1H, H2’), 4.39–4.31 (m, 1H, H4’), 4.23 (t, ^3^ *J* = 4.8 Hz, 1H, H3′), 4.11 (s, 2H, C*H*_2_), 4.01 (t, ^3^ *J* = 6.2 Hz, 1H, Hα), 3.63–3.60 (m, 1H, H5′_A_), 3.55–3.46 (m, 1H, H5′_B_), 3.32–3.18 (m, 4H, Hγ, H1’’), 3.02–2.89 (m, 2H, H3′’), 2.26–2.20 (m, 1H, Hβ_A_), 2.11–2.05 (m, 1H, Hβ_B_), 2.06–1.96 (m, 2H, H2’’); HR-MS (ESI): calcd. for C_24_H_35_N_8_O_5_ [M + H]^+^: 515.2725, found: 515.2718. Purified by preparative HPLC (Method M). HPLC: *t*_R_ = 10.190 min (Method F), UV purity at 254 nm: 96.3%.

#### *tert*-Butyl (S)-4-((3-((*tert*-Butoxycarbonyl)­(phenethyl)­amino)­propyl)­(((3aR,4R,6R,6aR)-6-(6-((*tert*-butoxycarbonyl)­amino)-9*H*-purin-9-yl)-2,2-dimethyltetrahydrofuro­[3,4-*d*]­[1,3]­dioxol-4-yl)­methyl)­amino)-2-((*tert*-butoxycarbonyl)­amino)­butanoate (**si8h**)

Reductive amination for compound **si8h** started from aldehyde **si13a** and followed the general procedure for the second reductive amination. Yield: 66 mg, 0.071 mmol, 24%. HR-MS (ESI): calcd. for C_47_H_73_N_8_O_11_ [M + H]^+^: 925.5393, found: 925.5391; R*_f_*: 0.71 (CH_2_Cl_2_/MeOH 9:1).

#### (S)-2-Amino-4-((((2R,3S,4R,5R)-5-(6-amino-9H-purin-9-yl)-3,4-dihydroxytetrahydro furan-2-Yyl)­methyl)­(3-(phenethylamino)­propyl)­amino)­butanoic acid (**5b**)

Deprotection of compound **si9h** followed the general procedure for deprotection. Yield: 62 mg, 0.071 mmol, 100% (3 TFA salt). ^1^H NMR (400 MHz; DMSO-*d*_6_): δ 8.86 (bs, 2H, N*H*_2_^+^), 8.73–8.23 (m, 5H, H2, H8, N*H*_3_^+^), 8.05 (bs, 3H, N*H*_3_^+^), 7.33 (t, ^3^ *J* = 7.2 Hz, 2H, *m*-H), 7.29–7.20 (m, 3H, *o*-H, *p*-H), 5.98 (d, ^3^ *J* = 4.8 Hz, 1H, H1’), 4.63 (t, ^3^ *J* = 4.8 Hz, 1H, H2’), 4.38–4.28 (m, 1H, H4’), 4.21 (t, ^3^ *J* = 4.8 Hz, 1H, H3′), 3.98 (t, ^3^ *J* = 5.8 Hz, 1H, Hα), 3.64–3.52 (m, 1H, H5′_A_), 3.52–3.41 (m, 1H, H5′_B_), 3.25–3.20 (m, 2H, Hγ), 3.20–3.05 (m, 4H, H1’’, C*H*_2_–N), 3.03–2.92 (m, 2H, H3′’), 2.92–2.77 (m, 2H, C*H*_2_–Ph), 2.27–2.13 (m, ^3^ *J* = 6.0 Hz, 1H, Hβ_A_), 2.13–2.01 (m, 1H, Hβ_B_), 2.00–1.94 (m, 2H, H2’’); HR-MS (ESI): calcd. for C_25_H_37_N_8_O_5_ [M + H]^+^: 529.2881, found: 529.2881; HPLC: *t*_R_ = 6.011 min (Method B), UV-purity at 254 nm: 93.5%.

#### *tert*-Butyl (S)-4-((3-((*tert*-butoxycarbonyl)­(3-phenylpropyl)­amino)­propyl) (((3aR,4R,6R,6aR)-6-(6-((*tert*-butoxycarbonyl)­amino)-9H-purin-9-yl)-2,2-dimethyltetra hydrofuro­[3,4-*d*]­[1,3]­dioxol-4-yl)­methyl)­amino)-2-((*tert*-butoxycarbonyl)­amino) Butanoate (**si8i**)

Reductive amination for **si8i** started from aldehyde **13b** and followed the general procedure for deprotection. Yield: 104.6 mg, 0.111 mmol, 41%. HR-MS (ESI): calcd. for C_48_H_75_N_8_O_11_ [M + H]^+^: 939.5550, found: 939.5550; R_f_: 0.82 (CH_2_Cl_2_/MeOH 9:1).

#### (S)-2-Amino-4-((((2R,3S,4R,5R)-5-(6-amino-9H-purin-9-yl)-3,4-dihydroxytetrahydro-furan-2-yl)­methyl)­(3-((3-phenylpropyl)­amino)­propyl)­amino)­butanoic acid (**5c**)

Deprotection of **si9i** followed the general procedure for deprotection. Yield: 101.8 mg, 0.115 mmol, 100% (3 TFA salt). ^1^H NMR (400 MHz; DMSO-*d*_6_): δ 8.68 (bs, 2H, N*H*_2_^+^), 8.49 (s, 1H, H2), 8.33 (s, 1H, H8), 8.18 (bs, 3H, N*H*_3_^+^), 7.33–7.29 (m, 2H, *m*-H), 7.22–7.19 (m, 3H, *o*-H, *p*-H), 6.00 (d, ^3^ *J* = 4.9 Hz, 1H, H1’), 4.64 (t, ^3^ *J* = 4.9 Hz, 1H, H2’), 4.38–4.31 (m, 1H, H4’), 4.23 (t, ^3^ *J* = 4.9 Hz, 1H, H3′), 4.00 (t, ^3^ *J* = 6.4 Hz, 1H, Hα), 3.64–3.58 (m, 1H, H5′_A_), 3.55–3.44 (m, 1H, H5′_B_), 3.31–3.13 (m, 4H, Hγ, H1’’), 3.00–2.90 (m, 2H, H3′’), 2.90–2.78 (m, 2H, C*H*_2_–N), 2.63 (t, ^3^ *J* = 7.6 Hz, 2H, C*H*_2_–Ph), 2.27–2.18 (m, 1H, Hβ_A_), 2.11–2.06 (m, 1H, Hβ_B_), 1.94–1.90 (m, 2H, H2’’), 1.90–1.80 (m, 2H, C–C*H*_2_–C); HR-MS (ESI): calcd. for C_26_H_37_N_8_O_5_ [M–H]^−^: 541.2892, found: 541.2894; HPLC: *t*_R_ = 6.510 min (Method B), UV-purity at 254 nm: 93.1%

#### Di-*tert*-Butyl 4,4’-((((3aR,4R,6R,6aR)-6-(6-(bis­(*tert*-butoxycarbonyl)­amino)-9*H*-purin-9-yl)-2,2-dimethyltetrahydrofuro­[3,4-*d*]­[1,3]­dioxol-4-yl)­methyl)­azanediyl)­(2S,2’S)-bis­(2-((*tert*-butoxycarbonyl)­amino)­butanoate) (**8k**)

*tert-*Butyl (*S*)-2-((*tert-*butoxycarbonyl)­amino)-4-oxobutanoate **si6** (357 mg, 1.31 mmol, 4.0 equiv) and MgSO_4_ (118 mg, 0.978 mmol, 3.0 equiv) were added to a solution of 5′-amino-*N* ^6^,*N* ^6^-bis­(*tert-*butoxycarbonyl)-5′-deoxy-2’,3′*-O-*isopropylidene-adenosine **si4a** (165 mg, 0.326 mmol, 1.0 equiv) in dry MeOH (3.3 mL) at 0 °C and stirred for 30 min before NaBH_3_CN (82 mg, 1.31 mmol, 4.0 equiv) was added. The reaction mixture was stirred overnight at rt. The solvent was removed under *vacuo*, water was added and the aqueous phase was extracted three times with EA. The combined organic layers were washed with brine, dried over Na_2_SO_4_ and concentrated *in vacuo*. The residue was purified by column chromatography (PE/EA, 1:1). The *bis*-Boc protected title compound **si8k** was obtained as a colorless oil (110 mg, 0.108 mmol, 33%) and the *mono*-Boc protected compound was obtained as a colorless foam (173 mg, 0.188 mmol, 58%). ^1^H NMR (500 MHz, CDCl_3_): δ = 1.44 (s, 30H), 1.46 (s, 30H), 2.14 (ddt, *J* = 14.5 Hz, *J* = 9.6 Hz, *J* = 3.2 Hz, 4H), 2.39–2.69 (m, 3H), 2.79 (m_c_, 1H), 3.31–3.50 (m, 3H), 3.58–3.76 (m, 7H), 4.35 (ddd, *J* = 11.0 Hz, *J* = 8.1 Hz, *J* = 3.6 Hz, 4H), 4.97 (m_c_, 1H), 5.33 (d, *J* = 7.6 Hz, 4H), 6.11 (d, *J* = 2.3 Hz, 1H), 8.19 (d, *J* = 4.4 Hz, 1H), 8.86 (s, 1H). HR-MS (ESI): *m*/*z* [M + H]^+^ calc. for C_49_H_81_O_15_N_8_: 1021.5816 found: 1021.5829.

#### (2S,2’S)-4,4’-((((2R,3S,4R,5R)-5-(6-Amino-9*H*-purin-9-yl)-3,4-dihydroxytetrahydrofuran-2-yl)­methyl)­azanediyl)­bis­(2-aminobutanoic Acid) (**1**)

The tertiary amine **si8k** was dissolved (0.02 M) in 2.2 mL freshly prepared TFA/H_2_O (4:1) solution and stirred at rt for 16 h, then evaporated to remove the TFA and dried using freeze-dryer to give the desired products **1** (71 mg, quant) as TFA salts as a colorless foam. ^1^H NMR (400 MHz, D_2_O): δ = 2.04–2.14 (m, 2H), 2.15–2.24 (m, 2H), 2.40 (m_c_, 1H), 2.75 (m_c_, 1H), 3.40–3.68 (m, 2H), 3.72–3.79 (m, 5H), 4.10 (dd, *J* = 8.0 Hz, *J* = 5.0 Hz, 1H), 4.17 (dd, *J* = 7.1 Hz, *J* = 5.4 Hz, 2H), 4.31–4.53 (m, 2H), 4.56 (td, *J* = 9.3 Hz, *J* = 1.2 Hz, 1H), 6.12 (dd, *J* = 10.7 Hz, *J* = 4.3 Hz, 1H), 8.40 (s, 1H), 8.41 (s, 1H). HR-MS (ESI): *m*/*z* [M + H]^+^ calc. for C_18_H_29_N_8_O_7_: 469.2154 found: 469.2156. Purified by preparative HPLC (Method H). HPLC: *t*_R_ = 2.935 min (Method C), UV purity at 254 nm: 97.4%

#### *tert*-Butyl (9-((3aR,4R,6R,6aR)-6-(((3-((*tert*-butoxycarbonyl)­amino)-4,4-difluorobutyl)­(3-((*tert*-butoxycarbonyl)­amino)­propyl)­amino)­methyl)-2,2-dimethyltetrahydrofuro­[3,4-*d*]­[1,3]­dioxol-4-yl)-9*H*-purin-6-yl)­carbamate (**si16a**)

Reductive amination for compound **16a** started from the aldehyde **si6** and the secondary amine **si15a** and followed the general procedure for the second reductive amination. Yield: 52.8 mg, 0.068 mmol, 32%. HR-MS (ESI): calcd. for C_35_H_57_N_8_O_9_F_2_ [M + H]^+^: 771.4211, found: 771.4221; R*_f_*: 0.79 (CH_2_Cl_2_/MeOH 9:1).

#### (2R,3S,4R,5R)-2-(((3-Amino-4,4-difluorobutyl)­(3-aminopropyl)­amino)­methyl)-5-(6-amino-9H-purin-9-yl)­tetrahydrofuran-3,4-diol (**4b**)

Deprotection of **si16a** followed the general procedure for deprotection. Yield: 68.3 mg, 0.088 mmol, 100% (3 TFA salt). ^1^H NMR (400 MHz; DMSO-*d*_6_): δ 8.69 (bs, 3H, N*H*_3_^+^), 8.51 (s, 1H, H2), 8.35 (s, 1H, H8), 8.26 (bs, 3H, N*H*_3_^+^), 7.93 (bs, 3H, N*H*_3_^+^), 6.42–6.10 (m, 1H, C*H*F_2_), 6.00 (d, *J* = 4.3 Hz, 1H, H1’), 4.64 (t, *J* = 4.3 Hz, 1H, H2’), 4.35–4.34 (m, 1H, H4’), 4.22 (s, 1H, H3′), 3.83–3.68 (m, 1H, Hα), 3.65–3.61 (m, 1H, H5′_A_), 3.50–3.48 (m, 1H, H5′_B_), 3.33–3.18 (m, 4H, Hγ, H1’’), 2.91–2.73 (m, 2H, H3′’), 2.12–2.10 (m, 1H, Hβ_A_), 1.96–1.90 (m, 3H, Hβ_B_, H2’’); HR-MS (ESI): calcd. for C_17_H_29_N_8_O_3_F_2_ [M + H]^+^: 431.2325, found: 431.2333. Purified by preparative HPLC (Method J). HPLC: *t*_R_ = 4.341 min (Method C), UV purity at 254 nm: 100% (mixture of diastereomers).

#### *tert*-Butyl (9-((3aR,4R,6R,6aR)-6-(((3-((*tert*-butoxycarbonyl)­amino)-4,4,4-trifluorobutyl) (3-((*tert*-butoxycarbonyl)­amino)­propyl)­amino)­methyl)-2,2-dimethyltetrahydrofuro­[3,4-*d*]­[1,3]­dioxol-4-yl)-9*H*-purin-6-yl)­carbamate (**si16b**)

Reductive amination for compound **si16b** started from the aldehyde **si6** and the secondary amine **si15b** and followed the general procedure for the second reductive amination. Yield: 45.8 mg, 0.058 mmol, 27%. HR-MS (ESI): calcd. for C_35_H_56_N_8_O_9_F_3_ [M + H]^+^: 789.4117, found: 789.4109; R*_f_*: 0.78 (CH_2_Cl_2_/MeOH 9:1).

#### (2R,3S,4R,5R)-2-(((3-Amino-4,4,4-trifluorobutyl)­(3-aminopropyl)­amino)­methyl)-5-(6-amino-9*H*-purin-9-yl)­tetrahydrofuran-3,4-diol (**4c**)

Deprotection of **si16b** followed the general procedure for deprotection. Yield: 41.4 mg, 0.052 mmol, 100% (3 TFA salt). ^1^H NMR (400 MHz; DMSO-*d*_6_): δ 8.48 (s, 1H, H2), 8.31 (s, 1H, H8), 8.10 (bs, 3H, N*H*_3_^+^), 7.89 (bs, 3H, N*H*_3_^+^), 6.00 (d, ^3^ *J* = 4.8 Hz, 1H, H1’), 4.66 (d, ^3^ *J* = 4.8 Hz, 1H, H2’), 4.36–4.33 (m, 1H, H4’), 4.25–4.20 (m, 1H, H3′), 4.11–4.07 (m, 1H, Hα), 3.67–3.59 (m, 1H, H5′_A_), 3.53–3.42 (m, 1H, H5′_B_), 3.35–3.28 (m, 1H, Hγ_A_), 3.25–3.18 (m, 3H, H1’’, Hγ_B_), 2.85–2.84 (m, 2H, H3′’), 2.14–2.09 (m, 1H, Hβ_A_), 2.02–1.85 (m, 3H, H2’’, Hβ_B_); HR-MS (ESI): calcd. for C_17_H_28_N_8_O_3_F_3_ [M + H]^+^: 449.2231, found: 449.2231. Purified by preparative HPLC (Method K). HPLC: *t*_R_ = 6.158 min (Method C), UV purity at 254 nm: 99.4% (mixture of diastereomers).

#### *tert*-Butyl (3-hydroxypropyl)­(phenethyl)­carbamate **(si12a**)

**si12a** was prepared according to the general procedure for the preparation of extended *N*-Boc protected amino-alcohols, starting from 2-phenylacetaldehyde. Yield: 111 mg, 0.39 mmol, 8%. C_16_H_25_NO_3_ (279.4 g/mol). ^1^H NMR (400 MHz; DMSO-*d*_6_): δ 7.33–7.25 (m, 2H, *m*-H), 7.23–7.16 (m, 3H, *o*-H, *p*-H), 4.41 (t, ^3^ *J* = 5.4 Hz, 1H, O*H*), 3.37 (q, ^3^ *J* = 5.9 Hz, 2H, C*H*_2_–O), 3.34–3.29 (m, 2H, Ph–C–C*H*_2_–N), 3.21–3.08 (m, 2H, N–C*H*_2_), 2.75 (t, ^3^ *J* = 7.4 Hz, 2H, C*H*_2_–Ph), 1.59 (quint, ^3^ *J* = 6.8 Hz, 2H, C–C*H*_2_–C), 1.43–1.28 (m, 9H, C*H*_3_ *t*Bu); APCI-MS­(+): *m*/*z* 180.1 [M–Boc+2H]^+^; R*_f_*: 0.68 (CH_2_Cl_2_/MeOH 9:1).

#### *tert*-Butyl (3-hydroxypropyl)­(3-phenylpropyl)­carbamate (**si12b**)

**si12b** was prepared according to the general procedure for the preparation of extended *N*-Boc protected amino-alcohols, starting from 2-phenylacetaldehyde. Yield: 302 mg, 1.69 mmol, 23%. C_17_H_27_NO_3_ (293.4 g/mol). ^1^H NMR (400 MHz; DMSO-*d*_6_): δ 7.29–7.25 (m, 2H, *m*-H), 7.21–7.14 (m, 3H, *o*-H, *p*-H), 4.43–4.41 (m, 1H, O*H*), 3.38 (q, ^3^ *J* = 5.9 Hz, C*H*_2_–O, 2H), 3.18–3.10 (m, 4H, 2 × C*H*_2_–N), 2.58–2.49 (m, 2H, Ph–C*H*_2_), 1.75–1.70 (m, 2H, Ph–C–C*H*_2_–N), 1.61–1.58 (m, 2H, O–C–C*H*_2_–C–N), 1.41–1.36 (m, 9H, C*H*_3_ *t*Bu); APCI-MS­(+): *m*/*z* 194.2 [M–Boc+2H]^+^; R*_f_*: 0.68 (CH_2_Cl_2_/MeOH 9:1).

#### 3-((4-Phenoxyphenethyl)­amino)­propan-1-ol (**si11b**)

A mixture of 2-(4-phenoxyphenyl)­acetaldehyde (1 equiv) and 3-aminopropan-1-ol (1 equiv) in anhydrous MeOH (0.5 M) was stirred at rt for 48 h. After the formation of the imine was confirmed according to TLC and MS, the mixture was cooled down to 0 °C and NaBH_4_ (1.5 equiv) was added portionwise and the reaction was stirred at rt overnight. The volatiles were evacuated *in vacuo* and the crude was extracted with H_2_O/EtOAc (3 times). The combined organics were dried over Na_2_SO_4_ and concentrated under reduced pressure. The crude residue was purified by flash chromatography (CH_2_Cl_2_/MeOH 99.6:0.4–92:8) to yield the title product. Yield: 589 mg, 2.171 mmol, 51%. C_17_H_21_NO_2_ (271.36 g/mol). ^1^H NMR (400 MHz; DMSO-*d*_6_): δ 7.39–7.35 (m, 2H, *m*’-H), 7.23–7.21 (m, 2H, *o*-H), 7.13–7.09 (m, 1H, *p*’-H), 6.98–6.96 (m, 2H, *o*’-H), 6.93–6.91 (m, 2H, *m*-H), 3.44 (t, ^3^ *J* = 6.5 Hz, 2H, C*H*_2_–O), 2.74–2.63 (m, 4H, C*H*_2_–N, C*H*_2_–Ph), 2.58 (t, ^3^ *J* = 6.5 Hz, 2H, C*H*_2_–N), 1.54 (quintet, ^3^ *J* = 6.5 Hz, 2H, CH_2_–C*H*_2_–CH_2_). R*_f_*: 0.16 (CH_2_Cl_2_/MeOH 9:1).

#### *tert*-Butyl benzyl­(3-hydroxypropyl)­carbamate (**si12c**)

According to the general procedure for the Boc protection of extended amino-alcohols 3-(benzylamino)­propan-1-ol was Boc-protected. Yield: 1116.7 mg, 4.208 mmol, 70%. C_15_H_23_NO_3_ (265.35 g/mol). ^1^H NMR (400 MHz; DMSO-*d*_6_): 7.34 (t, ^3^ *J* = 7.4 Hz, 2H, *m*-H), 7.28–7.23 (m, 1H, *p*-H), 7.22–7.20 (m, 2H, *o*-H), 4.41 (t, ^3^ *J* = 5.2 Hz, 1H, O*H*), 4.37 (s, 2H, Ph–C*H*_2_–N), 3.36 (q, ^3^ *J* = 6.0 Hz, 2H, C*H*_2_–O), 3.18–3.13 (m, 2H, C*H*_2_–N), 1.63–1.56 (m, 2H, C–C*H*_2_–C), 1.42–1.36 (m, 9H, C*H*_3_ *t*Bu). R*_f_*: 0.41 (CH_2_Cl_2_/MeOH 9:1).

#### *tert*-Butyl (3-hydroxypropyl)­(4-phenoxyphenethyl)­carbamate (**si12d**)

According to the general procedure for the Boc protection of extended amino-alcohols 3-((4-phenoxyphenethyl)­amino)­propan-1-ol was Boc-protected. Yield: 824 mg, 2.22 mmol, 100%. C_22_H_29_NO_4_ (371.48 g/mol). ^1^H NMR (400 MHz; DMSO-*d*_6_): δ 7.38–7.34 (m, 2H, *m*’-H), 7.21–7.19 (m, 2H, *o*-H), 7.13–7.09 (m, 1H, *p*’-H), 6.98–6.93 (m, 4H, *m*-H, *o*’-H), 4.44–4.42 (m, 1H, O*H*), 3.41–3.37 (m, 2H, C*H*_2_–O), 3.35–3.30 (m, 2H, C*H*_2_-N), 3.16–3.14 (m, 2H, C*H*_2_–N), 2.74 (t, *J* = 7.4 Hz, 2H, C*H*_2_–Ph), 1.62–1.56 (m, 2H, C–C*H*_2_–C), 1.44–1.26 (m, 9H, C*H*_3_ *t*Bu). R*_f_*: 0.75 (CH_2_Cl_2_/MeOH 9:1).

#### *tert*-Butyl (3-oxopropyl)­(phenethyl)­carbamate (**si13a**)

According to the general procedure for Swern oxidation **si13a** was prepared starting from **si12a**. Yield: 84.2 mg, 0.3 mmol, 77%. C_16_H_23_NO_3_ (277.36 g/mol). ^1^H NMR (400 MHz; DMSO-*d*_6_): δ 9.64 (t, ^3^ *J* = 1.6 Hz, 1H, C*H*O), 7.33–7.25 (m, 2H, *m*-H), 7.22–7.18 (m, 3H, *o*-H, *p*-H), 3.39–3.38 (m, 2H, N–C*H*_2_), 3.36–3.28 (m, 2H, C*H*_2_–N), 2.74 (t, ^3^ *J* = 7.6 Hz, 2H, C*H*_2_–Ph), 2.60 (td, ^3^ *J* = 6.7 Hz and ^3^ *J* = 1.6 Hz, 1H, C*H*_2_-CHO), 1.36–1.32 (m, 9H, C*H*_3_ *t*Bu); R*_f_*: 0.71 (CH_2_Cl_2_/MeOH 9:1).

#### *tert*-Butyl (3-oxopropyl)­(3-phenylpropyl)­carbamate (**si13b**)

According to the general procedure for Swern oxidation **si13b** was prepared starting from **si12b**. Yield: 235 mg, 0.806 mmol, 80%. C_17_H_25_NO_3_ (291.4 g/mol). ^1^H NMR (400 MHz; DMSO-*d*_6_): δ 9.65 (s, 1H, C*H*O), 7.29–7.26 (m, 2H, *m*-H), 7.21–7.14 (m, 3H, *o*-H, *p*-H), 3.43 (t, ^3^ *J* = 6.6 Hz, 2H, CHO–C–C*H*_2_–N), 3.23–3.06 (m, 2H, C*H*_2_–N), 2.61 (t, ^3^ *J* = 6 Hz, 2H, C*H*_2_–CHO), 2.57–2.49 (m, 2H, C*H*_2_–Ph), 1.77–1.73 (m, 2H, C–C*H*_2_–C), 1.41–1.36 (m, 9H, C*H*_3_ *t*Bu); APCI-MS­(+): *m*/*z* 192.2 [M–Boc+2H]^+^; R*_f_*: 0.71 (CH_2_Cl_2_/MeOH 9:1).

#### *tert*-Butyl benzyl­(3-oxopropyl)­carbamate (**si13c**)

According to the general procedure for Swern oxidation **si13c** was prepared starting from **si12c**. Yield: 322 mg, 0.906 mmol, 93%. C_21_H_25_NO_4_ (355.43 g/mol). ^1^H NMR (400 MHz; DMSO-*d*_6_): δ 9.63 (t, ^3^ *J* = 1.7 Hz, 1H, C*H*O), 7.38–7.30 (m, 2H, *m*-H), 7.28–7.24 (m, 1H, *p*-H), 7.24–7.21 (m, 2H, *o*-H), 4.38 (s, 2H, N–C*H*_2_-Ph), 3.41–3.38 (m, 2H, C*H*_2_-N), 2.60 (td, ^3^ *J* = 6.6 Hz and ^3^ *J* = 1.7 Hz, 2H, C*H*_2_-CHO), 1.41–1.35 (m, 9H, C*H*_3_ *t*Bu). R*_f_*: 0.75 (CH_2_Cl_2_/MeOH 9:1).

#### *tert*-Butyl (3-oxopropyl)­(4-phenoxyphenethyl)­carbamate (**si13d**)

According to the general procedure for Swern oxidation **si13d** was prepared starting from **si12d**. Yield: 591 mg, 1.60 mmol, 83%. C_22_H_27_NO_4_ (369.46 g/mol). ^1^H NMR (400 MHz; DMSO-*d*_6_): δ 9.65 (s, 1H, C*H*O), 7.39–7.35 (m, 2H, *m*’’-H), 7.22–7.20 (m, 2H, *o*-H), 7.14–7.08 (m, 1H, *p*’’-H), 6.97–6.93 (m, 4H, *m*-H, *o*’’-H), 3.42–3.38 (m, 2H, C*H*_2_-N), 3.34 (t, *J* = 7.4 Hz, 2H, N–C*H*_2_), 2.74 (t, *J* = 7.4 Hz, 2H, C*H*_2_-Ph), 2.61 (td, *J* = 6.8, 2.0 Hz, 2H, C*H*_2_-CHO), 1.39–1.30 (m, 9H, C*H*_3_ *t*Bu). R*_f_*: 0.69 (CH_2_Cl_2_/MeOH 9:1).

#### *tert*-Butyl (3-((((3aR,4R,6R,6aR)-6-(6-amino-9*H*-purin-9-yl)-2,2-dimethyltetrahydrofuro [3,4-*d*]­[1,3]­dioxol-4-yl)­methyl)­amino)­propyl)­carbamate (**si19**)

A solution of compound **si18** (1.5 g, 4.9 mmol), *tert*-butyl (3-oxopropyl)­carbamate (850 mg, 4.9 mmol) and NaBH_3_CN (925 mg, 14.6 mmol) in EtOH (100 mL) was stirred at 30 °C overnight. The reaction mixture was concentrated and purified by column chromatography (DCM:MeOH = 10:1) to give compound **si19** (1.7 g, 76% yield) as colorless oil. MS Calcd: 464.3; MS Found: 464.3 [M + H]^+^.

#### *tert*-Butyl (R)-2-(((((3aR,4R,6R,6aR)-6-(6-amino-9*H*-purin-9-yl)-2,2-dimethyltetrahydro furo­[3,4-*d*]­[1,3]­dioxol-4-yl)­methyl)­(3-((*tert*-butoxycarbonyl)­amino)­propyl)­amino) methyl)-2,5-dihydro-1*H*-pyrrole-1-carboxylate (**si20**)

A solution of compound **si19** (352 mg, 0.76 mmol) and compound **si31** (150 mg, 0.76 mmol) in EtOH (4 mL) was stirred at rt for 20 min. Then AcOH (2 drops) and NaBH_3_CN (96 mg, 1.52 mmol) were added. The reaction mixture was stirred at rt for 24 h. The reaction mixture was concentrated and purified by *prep*-HPLC to give compound **si20** (60 mg, 12% yield) as a white solid. MS Calcd: 645.4; MS Found: 645.4 [M + H]^+^.

#### (2R,3R,4S,5R)-2-(6-Amino-9*H*-purin-9-yl)-5-(((3-aminopropyl)­(((R)-2,5-dihydro-1*H*-pyrrol-2-yl)­methyl)­amino)­methyl)­tetrahydrofuran-3,4-diol (**4f**)

A solution of compound **si20** (30 mg, 0.046 mmol) and TFA (2 mL) in DCM (2 mL) was stirred at rt for 2 h. Then H_2_O (1 mL) was added. The reaction mixture was stirred at rt for another 3 h. The reaction mixture was concentrated and purified by *prep*- HPLC to give compound **4f** (14.9 mg, 79% yield) as yellow oil. ^1^H NMR (400 MHz, CD_3_OD) δ: 8.32 (s, 1H), 8.28 (s, 1H), 5.95 (d, *J =* 3.6 Hz, 1H), 5.81–5.73 (m, 2H), 4.56–4.52 (m, 2H), 4.21–4.12 (m, 2H), 3.94–3.84 (m, 2H), 2.98–2.59 (m, 8H), 1.79–1.73 (m, 2H). MS: calcd. for C_18_H_29_N_8_O_3_ [M + H] ^+^: 405.2; found: 405.2. HPLC: *t*_R_ = 3.497 min (Method S2), UV-purity at 254 nm: 99.1%.

#### *tert*-Butyl (3-((((3aR,4R,6R,6aR)-6-(6-amino-9*H*-purin-9-yl)-2,2-dimethyltetrahydrofuro [3,4-*d*]­[1,3]­dioxol-4-yl)­methyl)­(2-cyanoethyl)­amino)­propyl)­carbamate (**si22**)

To a solution of compound **si19** (1.67 g, 3.6 mmol) in MeOH (30 mL) were added Et_3_N (400 g, 3.96 mmol) and acrylonitrile (210 mg, 3.96 mmol). The reaction mixture was stirred at 60 °C overnight and concentrated to dryness. The crude was purified by silica gel column (DCM/MeOH = 50/1) to give compound **si22** (1.7 g, yield: 91%) as a yellow oil. MS Calcd: 517.3; MS Found: 517.2 [M + H]^+^.

#### *tert*-Butyl (3-((2-(2*H*-tetrazol-5-yl)­ethyl)­(((3aR,4R,6R,6aR)-6-(6-amino-9*H*-purin-9-yl)-2,2-dimethyltetrahydrofuro­[3,4-d]­[1,3]­dioxol-4-yl)­methyl)­amino)­propyl)­carbamate (**si23**)

To a solution of compound **si22** (1.7 g, 3.3 mmol) in DMF (20 mL) were added NH_4_Cl (1.78 g, 33 mmol) and NaN_3_ (1.05 g, 16.5 mmol). The reaction mixture was stirred at 130 °C overnight. The mixture was quenched with H_2_O (50 mL) and extracted with EtOAc (50 mL x 3). The organic layer was washed with NaCl (50 mL), concentrated to dryness. The residue was purified by silica gel column (DCM/MeOH = 10/1) to give compound **si23** (400 mg, yield: 22%) as a white solid. MS Calcd: 560.3; MS Found: 560.3 [M + H]^+^.

#### (2R,3S,4R,5R)-2-(((2-(2*H*-tetrazol-5-yl)­ethyl)­(3-aminopropyl)­amino)­methyl)-5-(6-amino-9*H*-purin-9-yl)­tetrahydrofuran-3,4-diol (**4g**)

To a solution of compound **si23** (400 mg, 0.71 mmol) in DCM (15 mL) were added TFA (5 mL) and H_2_O (5 mL). The reaction mixture was stirred at room temperature overnight. The reaction mixture was concentrated and purified by *prep*-HPLC to give **4g** (97.5 mg, yield: 33%) as a white solid. ^1^H NMR (400 MHz, CD_3_OD): δ = 8.30 (d, *J* = 11.2 Hz, 2H), 5.99 (d, *J* = 4.0 Hz, 1H), 4.66–4.63 (m, 1H), 4.39–4.31 (m, 2H), 3.67–3.52 (m, 4H), 3.32–3.26 (m, 4H), 2.95–2.91 (m, 2H), 2.06–1.99 (m, 2H). MS: calcd. for C_16_H_26_N_11_O_3_ [M + H]^+^: 420.2; found: 420.2. HPLC: *t*_R_ = 2.421 min (Method S2), UV-purity at 254 nm: 99.7%

#### *tert*-Butyl (3-((S)-*N*-(((3aR,4R,6R,6aR)-6-(6-amino-9*H*-purin-9-yl)-2,2-dimethyltetra hydrofuro­[3,4-*d*]­[1,3]­dioxol-4-yl)­methyl)-2-(4-((*tert*-butyldiphenylsilyl)­oxy)­piperidin-1-yl)­propanamido)­propyl)­carbamate (**si25**)

To a solution of compound **si33** (48.8 mg, 0.12 mmol) and compound **si19** (50 mg, 0.11 mmol) in DMF (5 mL) was added PyBop (61.4 mg, 0.12 mmol) and DIPEA (70 mg, 0.54 mmol). The reaction mixture was stirred at rt overnight. The reaction mixture was quenched with saturated NH_4_Cl (10 mL) and extracted with DCM. The combined organic layer was washed with brine, dried over Na_2_SO_4_, filtered and concentrated. The residue was purified by column chromatography (DCM/MeOH) to give compound **si25** (79 mg, 85.8% yield) as a white solid. MS Calcd: 857.5; MS Found: 857.5 [M + H]^+^.

#### *tert*-Butyl (3-((((3aR,4R,6R,6aR)-6-(6-amino-9*H*-purin-9-yl)-2,2-dimethyltetrahydrofuro [3,4-*d*]­[1,3]­dioxol-4-yl)­methyl)­((s)-2-(4-((*tert*-butyldiphenylsilyl)­oxy)­piperidin-1-yl) propyl)­amino)­propyl)­carbamate (**si26**)

A solution of LiAlH_4_ (2.41 mL, 1 M in THF, 2.41 mmol) and AlCl_3_ (321 mg, 2.41 mmol) in THF (20 mL) was stirred at 40 °C for 30 min. Then the reaction mixture was cooled to 0 °C. Then a solution of compound **si25** (69 mg, 0.08 mmol) in THF (2 mL) was added slowly. The reaction mixture was stirred at 0 °C for 30 min. The reaction mixture was quenched with saturated Na_2_CO_3_ (5 mL) and extracted with DCM. The combined organic layer was washed with brine, died over Na_2_SO_4_, filtered and concentrated. The residue was purified by *prep*-HPLC to give compound **si26** (15 mg, 22% yield) as colorless oil. MS Calcd: 843.5; MS Found: 843.5 [M + H]^+^.

#### (2R,3R,4S,5R)-2-(6-Amino-9*H*-purin-9-yl)-5-(((3-aminopropyl)­((s)-2-(4-hydroxypiperidin-1-yl)­propyl)­amino)­methyl)­tetrahydrofuran-3,4-diol (**4e**)

A solution of compound **si26** (15 mg, 0.018 mmol) and TFA (3 mL) in DCM (2 mL) was stirred at rt for 4 h. Then H_2_O (0.5 mL) was added and stirred at rt for another 2 h. The reaction mixture was concentrated and purified by *prep*-HPLC to give compound **4e** (2.4 mg, 29% yield) as yellow oil. ^1^H NMR (400 MHz, CD_3_OD) δ: 8.29 (s, 1H), 8.23 (s, 1H), 5.98 (d, *J =* 3.6 Hz, 1H), 4.55 (dd, *J =* 5.6, 4.0 Hz, 1H), 4.23 (t, *J =* 6.0 Hz, 1H), 4.08–4.76 (m, 1H), 3.72–3.75 (m, 1H), 3.21–3.23 (m, 1H), 3.08–3.15 (m, 1H), 2.64–2.97 (m, 11H), 1.67–1.88 (m, 5H) 1.35–1.45, (m, 1H), 1.01 (d, *J =* 6.8 Hz, 3H). MS: calcd. for C_21_H_37_N_8_O_4_ [M + H]^+^: 465.3; found: 465.3 [M + H]^+^. HPLC: *t*_R_ = 3.249 min (Method S2), UV-purity at 254 nm: 99.3%

#### 1-(*tert*-Butyl)­2-methyl (2*R*,4*S*)-4-bromopyrrolidine-1,2-dicarboxylate (**si28**)

To a solution of 1-(*tert*-butyl)-2-methyl-(2*R*,4*R*)-4-hydroxypyrrolidine-1,2-dicarboxylate (5.0 g, 20.4 mmol) and CBr_4_ (8.1 g, 24.5 mmol) in DCM (100 mL) was added PPh_3_ (12.4 g, 40.8 mmol). The reaction mixture was stirred at rt overnight. The reaction mixture was concentrated and the residue was purified by column (PE/EtOAc 5:1) to give compound **si28** (4.5 g, 72% yield) as colorless liquid.

#### 1-(*tert*-Butyl) 2-methyl (R)-2,5-dihydro-1*H*-pyrrole-1,2-dicarboxylate (**si29**)

A solution of compound **si28** (2.0 g, 6.5 mmol) and TBAF (7.2 mL, 1 M in THF, 7.2 mmol) in DMF (30 mL) was stirred at 50 °C for 20 min. The reaction mixture was cooled, quenched with H_2_O (20 mL) and extracted with EtOAc. The combined organic layer was concentrated. The residue was purified by column (PE:EtOAc = 10:1) to give compound **si29** (1.4 g, 94% yield) as colorless liquid. MS Calcd: 228.1; MS Found: 228.1 [M + H]^+^.

#### *tert*-Butyl (R)-2-(hydroxymethyl)-2,5-dihydro-1*H*-pyrrole-1carboxylate (**si30**)

To a solution of compound LiAlH_4_ (469 mg, 12.34 mmol) in THF (50 mL) was added a solution of compound **si29** (1.4 g, 6.17 mmol) in THF (10 mL) slowly under ice–water bath cooling over 10 min. The reaction mixture was stirred at rt for 30 min. The reaction mixture was quenched with saturated NH_4_Cl (20 mL) and extracted with DCM. The combined organic layer was washed with brine, dried over Na_2_SO_4_, filtered and concentrated. The residue was purified by column to give compound **si30** (340 mg, 27%) as colorless liquid. MS Calcd: 200.1; MS Found: 200.1 [M + H]^+^.

#### *tert*-Butyl (R)-2-formyl-2,5-dihydro-1*H*-pyrrole-1-carboxylate (**si31**)

A solution of compound **si30** (340 mg, 1.71 mmol) and Dess-Martin (869 mg, 3.42 mmol) in DCM (20 mL) was stirred at 0 °C for 3 h. The reaction mixture was concentrated. The residue was purified by column to give compound **si31** (240 mg, 71% yield) as yellow liquid. MS Calcd: 198.1; MS Found: 198.1 [M + H]^+^.

#### 4-((*tert*-Butyldiphenylsilyl)­oxy)­piperidine (**si32**)

A solution of piperidin-4-ol (4 g, 39.55 mmol), Et_3_N (12 g, 118.59 mmol) and *tert*-butyl­(chloro)­diphenylsilane (16 g, 58.21 mmol) in THF (100 mL) was stirred at rt for 16 h. The reaction mixture was filtered out. The filtrate was dried over anhydrous sodium sulfate, filtered, and concentrated under vacuum. The residue was purified by column (PE/EtOAc = 1:2) to afford compound **si32** (1.1 g) as colorless oil. MS Calcd: 340.2; MS Found: 340.2 [M + H]^+^.

#### (S)-2-(4-((*tert*-Butyldiphenylsilyl)­oxy)­piperidin-1-yl)­propanoic Acid (**si33**)

A solution of compound **si32** (500 mg, 1.47 mmol), (*R*)-2-bromopropanoic acid (271 mg, 1.77 mmol) and Et_3_N (289 mg, 2.95 mmol) in THF (20 mL) was stirred at rt for 5h. The reaction mixture was quenched with H_2_O (20 mL) and extracted with EA. The combined organic layer was concentrated. The residue was purified by *prep*-HPLC to give compound **si33** (100 mg, 16.5% yield) as colorless oil. MS Calcd: 412.2; MS Found: 412.2 [M + H]^+^.

#### (S)-3-(((benzyloxy)­carbonyl)­amino)-4-(*tert*-butoxy)-4-oxobutanoic Acid (**si34**)

To a solution of (*S*)-3-amino-4-(*tert*-butoxy)-4-oxobutanoic acid (7.0 g, 37 mmol) in 70 mL/70 mL of dioxane/H_2_O was added NaHCO_3_ (9.3 g, 111 mmol) at room temperature. After stirring for 2 h, Cbz-Cl (68 mL, 48 mmol) was added under ice–water bath cooling. The reaction mixture was stirred at room temperature overnight. The reaction was diluted with H_2_O (50 mL × 2) and extracted with DCM (50 mL × 3). The combined organic phase was washed with brine (50 mL × 2), dried over anhydrous Na_2_SO_4_, filtered and concentrated to give compound **si34** (2.7 g, 22.5% yield) as yellow oil which was used to the next step without further purification. MS Calcd: 324; MS Found: 324 [M + H]^+^.

#### *tert*-Butyl ((Benzyloxy)­carbonyl)-l-homoserinate (**si35**)

To a solution of compound **si34** (1.7 g, 5.26 mmol) in THF (20 mL) was added BH_3_.DMS (10 M, 1.2 mL, 2.3 mmol) at 0 °C. The mixture was stirred at room temperature for 5 h. The reaction mixture was quenched with MeOH (10 mL) and concentrated to get the crude product. The residue was purified by flash chromatography on silica gel (PE/EtOAc = 2/1) to give compound **si35** (780 mg, 48% yield) as yellow oil. MS Calcd: 310; MS Found: 310 [M + H]^+^.

#### *tert*-Butyl (S)-2-(((benzyloxy)­carbonyl)­amino)-4-oxobutanoate (**si36**)

A solution of compound **si35** (780 mg, 2.52 mmol) in DCM (10 mL) was added PCC (1.63 g, 7.56 mmol). The reaction was stirred at room temperature for 4 h. The reaction mixture was diluted with silica gel (2.0 g), filtered and the residue was concentrated to give compound **si36** (580 mg, 75% yield) as yellow oil which was used to the next step without further purification. MS Calcd: 308; MS Found: 308 [M + H]^+^.

#### *tert*-Butyl (3-((((3aR,4R,6R,6aR)-6-(6-amino-9*H*-purin-9-yl)-2,2-dimethyltetrahydrofuro [3,4-*d*]­[1,3]­dioxol-4-yl)­methyl)­amino)-1-phenylpropyl)­carbamate (**si37**)

To a mixture of compound **si18** (500 mg, 1.6 mmol) in 10 mL of MeOH was added *tert*-butyl (3-oxo-1-phenylpropyl)­carbamate (597 mg, 2.4 mmol). After stirring for 5 min, NaBH_3_CN (302 mg, 4.8 mmol) was added under ice–water bath cooling. The reaction mixture was stirred at 45 °C overnight. The reaction mixture was concentrated, and the residue was purified by flash chromatography on reverse phase silica gel (ACN/H_2_O = 5%–95%, 254 nm, 30 min) to give compound **si37** (200 mg, 23% yield) as a yellow solid. MS Calcd: 540; MS Found: 540 [M + H]^+^.

#### *tert*-Butyl (2S)-4-((((3aR,4R,6R,6aR)-6-(6-amino-9*H*-purin-9-yl)-2,2-dimethyltetrahydro furo­[3,4-*d*]­[1,3]­dioxol-4-yl)­methyl)­(3-((*tert*-butoxycarbonyl)­amino)-3-phenylpropyl) amino)-2-(((benzyloxy)­carbonyl)­amino)­butanoate (**si38**)

To a solution of compound **si37** (100 mg, 0.19 mmol) and **si36** (74 mg, 0.24 mmol) in MeOH (10 mL) and AcOH (0.1 mL) was added NaBH_3_CN (36 mg, 0.57 mmol). The reaction mixture was stirred at 35 °C overnight. The reaction mixture was concentrated and the residue was purified by flash chromatography on silica gel (DCM/MeOH = 10/1) to give compound **si38** (100 mg, 65% yield) as yellow oil. MS Calcd: 831; MS Found: 831 [M + H]^+^

#### (2S)-2-Amino-4-((3-amino-3-phenylpropyl)­(((2R,3S,4R,5R)-5-(6-amino-9*H*-purin-9-yl)-3,4-dihydroxytetrahydrofuran-2-yl)­methyl)­amino)­butanoic Acid (**2b**)

To a solution of compound **si38** (100 mg, 0.12 mmol) in AcOH (2 mL) was added HBr (30% in AcOH, 2 mL). The reaction mixture was stirred at room temperature for 3 h. The reaction mixture was concentrated. The residue was dissolved in 2 mL of MeOH and adjusted pH = 7 with Na_2_CO_3_ solution. The residue was concentrated and purified by *prep*-HPLC (acidic conditions) to give compound **2b** (12 mg, 20% yield) as a white solid. ^1^H NMR (400 MHz, CD_3_OD) δ: 8.23–818 (m, 2H), 728–7.24 (m, 5H), 5.59 (d, *J* = 3.6 Hz, 1H), 4.55–4.50 (m, 1H), 4.31–4.25 (m, 3H), 3.86–3.81 (m, 1H), 3.55–3.32 (m, 3H), 2.89–2.79 (m, 1H), 2.46–2.26 (m, 2H), 2.23–2.08 (m, 1H), 2.02–1.92 (m, 1H), 1.21–1.19 (m, 2H). MS: calcd. for C_23_H_33_N_8_O_5_ [M + H]^+^: 501.2; found: 501.2. HPLC: *t*_R_ = 2.034 min (diastereomer 1) and *t*_R_ = 2.104 min (diastereomer 2) (Method S2). UV-purity at 254 nm: 99.7%.

#### *tert*-Butyl 4-((((3aR,4R,6R,6aR)-6-(6-amino-9*H*-purin-9-yl)-2,2-dimethyltetrahydrofuro [3,4-*d*]­[1,3]­dioxol-4-yl)­methyl)­(3-((*tert*-butoxycarbonyl)­amino)­propyl)­amino)­piperidine-1-carboxylate (**si40**)

A solution of compound **si18** (150 mg, 0.49 mmol) in 10 mL of MeOH was added *tert*-butyl 4-oxopiperidine-1-carboxylate (297 mg, 1.47 mmol). After stirring for 5 min, NaBH_3_CN (228 mg, 1.47 mmol) was added. The reaction mixture was stirred at room temperature overnight. Compound *tert*-butyl (3-oxopropyl)­carbamate (173 mg, 0.98 mmol) was added. The reaction mixture was stirred at room temperature overnight. The reaction mixture was concentrated and the residue was purified by flash chromatography on reverse phase silica gel (H_2_O/ACN) to give compound **si40** (20 mg, 6% yield) as a white solid. MS Calcd: 647; MS Found: 647 [M + H]^+^.

#### (2R,3R,4S,5R)-2-(6-Amino-9*H*-purin-9-yl)-5-(((3-aminopropyl)­(piperidin-4-yl)­amino) methyl)­tetrahydrofuran-3,4-diol (**4d**)

To a solution of compound **si40** (20 mg, 0.31 mmol) in DCM (2.5 mL) was added TFA (1.5 mL) and water (0.5 mL). The reaction mixture was stirred at room temperature for 2 h. The reaction mixture was concentrated to give compound **4d** after purification via *prep*-HPLC (acidic conditions) (19.5 mg, 97% yield) as a yellow solid. ^1^H NMR (400 MHz, CD_3_OD) δ: 8.43 (s, 1H), 8.41 (s, 1H), 6.10 (d, *J* = 3.2 Hz, 1H), 4.69 (t, *J* = 4.0 Hz, 1H), 4.45–4.38 (m, 2H), 3.58–3.52 (m, 5H), 3.24–3.21 (m, 2H), 3.10–2.85 (m, 4H), 2.30–2.19 (m, 2H), 2.07–2.00 (m, 4H). MS calcd. for C_18_H_31_N_8_O_3_ [M + H]^+^: 407.2; found: 407.2 [M + H]^+^. HPLC: *t*_R_ = 2.059 min (Method S1), UV-purity at 254 nm: 98.6%.

#### (1R,2S,3R,5R)-3-(4-Chloro-7*H*-pyrrolo­[2,3-*d*]­pyrimidin-7-yl)-5-(hydroxymethyl) cyclopentane-1,2-diol (**si42**)

To a solution of 3-amino-5-(hydroxymethyl)­cyclopentane-1,2-diol (5.0 g, 27 mmol, 1 equiv) in abs. grade EtOH (234 mL) was added 4,6-dichloropyrimidin-5-acetaldehyde (5.15 g, 27 mmol, 1 equiv) and Et_3_N (12.5 mL, 53.9 mmol, 2 equiv). The reaction mixture was refluxed (90 °C) for 16 h. Volatiles were then evaporated *in vacuo* and the corresponding residue was purified by silica gel flash column chromatography (CH_2_Cl_2_/MeOH 99:1–90:10) to afford compound **si42** as a yellow solid (7.39 g, 26 mmol, 97%). C_12_H_14_N_3_O_3_Cl (283.7 g/mol). ^1^H NMR (400 MHz; DMSO-*d*_6_): δ 8.61 (s, 1H, H2), 7.91 (d, ^3^ *J* = 3.6 Hz, 1H, H8), 6.68 (d, ^3^ *J* = 3.6 Hz, 1H, H7), 5.04 (dt, ^3^ *J* = 10.2 Hz and ^3^ *J* = 8.9 Hz, 1H, H1’), 4.89–4.75 (m, 3H, 3 × O*H*), 4.23 (dd, ^3^ *J* = 8.9 Hz and ^3^ *J* = 5.2 Hz, 1H, H2’), 3.83 (dd, ^3^ *J* = 5.2 Hz and ^3^ *J* = 2.8 Hz, 1H, H3′), 3.52–3.43 (m, 2H, H5′), 2.22 (dt, ^3^ *J* = 12.8 Hz ^3^ *J* = 8.9 Hz, 1H, H6’_A_), 2.09–2.02 (m, 1H, H4’), 1.61 (ddd, ^3^ *J* = 12.8 Hz, ^3^ *J* = 10.2 Hz and ^3^ *J* = 7.6 Hz, 1H, H6’_B_); ^13^C NMR (101 MHz, DMSO-*d*_6_) δ 151.0, 150.4, 149.9, 129.2, 116.9, 98.6, 75.2, 71.7, 62.8, 59.3, 45.1 29.5; APCI-MS­(+): *m*/*z* 284.0 [M + H]^+^; R*_f_*: 0.29 (CH_2_Cl_2_/MeOH 9:1).

#### ((3aR,4R,6R,6aS)-6-(4-Chloro-7*H*-pyrrolo­[2,3-*d*]­pyrimidin-7-yl)-2,2-dimethyltetrahydro-4*H*-cyclopenta­[d]­[1,3]­dioxol-4-yl)­methanol (**si43**)

To a solution of **si42** (7.39 g, 25.8 mmol, 1 equiv) in acetone (685 mL) was added CH­(OEt)_3_ (21.6 mL, 128.9 mmol, 5 equiv) followed by *p*TsOH (24.7 g, 128.9 mmol, 5 equiv) and the mixture was stirred at rt for 16. After quenching with 5% NaHCO_3_, most of acetone was evaporated and the remaining aqueous solution was extracted 3 times with CH_2_Cl_2_. The combined organic layer were dried over Na_2_SO_4_, filtered and evaporated to afford the crude product subjected to flash column chromatography (CH_2_Cl_2_/MeOH 99.6:0.4–97.5:2.5). The pure compound **si43** was obtained as a beige foam (4.81 g, 14.8 mmol, 58%). C_15_H_18_N_3_O_3_Cl (323.8 g/mol). ^1^H NMR (400 MHz; DMSO-*d*_6_): δ 8.65 (s, 1H, H2), 7.96 (d, ^3^ *J* = 3.8 Hz, 1H, H8), 6.72 (d, ^3^ *J* = 3.8 Hz, 1H, H7), 5.12–5.06 (m, 1H, H1’), 4.91 (t, ^3^ *J* = 7.0 Hz, 1H, H2’), 4.81 (t, ^3^ *J* = 5.4 Hz, 1H, O*H*-5′), 4.55 (dd, ^3^ *J* = 7.0 Hz and ^3^ *J* = 4.4 Hz, 1H, H3′), 3.52 (td, ^3^ *J* = 5.2 Hz and ^4^ *J* = 0.8 Hz, 2H, H5′), 2.28–2.21 (m, 2H, H4’, H6’_A_), 2.13–2.08 (m, 1H, H6’_B_), 1.48 (s, 3H, C*H*_3_), 1.22 (s, 3H, C*H*_3_); ^13^C NMR (101 MHz, DMSO-*d*_6_) δ 150.7, 150.6, 150.2, 129.2, 116.9, 112.4, 99.1, 83.4, 80.6, 61.9, 60.7, 45.2, 33.8, 27.4, 25.1; APCI-MS­(+): 324.1 [M + H]^+^; R*_f_*: 0.57 (CH_2_Cl_2_/MeOH 9:1).

#### ((3aR,4R,6R,6aS)-2,2-Dimethyl-6-(4-(methylamino)-7*H*-pyrrolo­[2,3-*d*]­pyrimidin-7-yl)­tetrahydro-4*H*-cyclopenta­[d]­[1,3]­dioxol-4-yl)­methanol (**si44**)

Compound **si43** (2.93 g, 8.96 mmol) was dissolved in *n*-BuOH (22.40 mL). Then, a 33% CH_3_NH_2_ solution in EtOH (22.40 mL) was added. The mixture was stirred under microwave irradiation at 120 °C (100 W, 100 psi) for 25 min. The volatiles were removed *in vacuo* and the resulting residue was purified over silica (CH_2_Cl_2_/MeOH; 0–10%) to afford the title product as grayish foam (2.25 g, 79%). ^1^H NMR (400 MHz, DMSO-*d*_6_) δ 8.14 (s, 1H, H2), 7.46 (q, *J* = 4.3 Hz, 1H, NH), 7.30 (d, *J* = 3.5 Hz, 1H, H6), 6.56 (d, *J* = 3.5 Hz, 1H, H5), 4.93 (dt, *J* = 11.7, 6.3 Hz, 1H, H1́), 4.90–4.85 (m, 1H, H2́), 4.77 (t, *J* = 5.4 Hz, 1H, 5́OH), 4.52 (dd, *J* = 7.0, 4.5 Hz, 1H, H3́), 3.50 (td, *J* = 5.4, 2.0 Hz, 2H, CH_2_, H5́), 2.96 (d, *J* = 4.7 Hz, 3H, CH_3_, NMe), 2.23–2.13 (m, 2H, H4́, CH_2_, cyclopentane), 2.08–1.99 (m, 1H, CH_2_, cyclopentane), 1.47 (s, 3H, CH_3_, acetonide), 1.22 (s, 3H, CH_3_, acetonide). APCI-MS­(+): *m*/*z* 319.4 [M + H]^+^; R*_f_*: 0.32 (CH_2_Cl_2_/MeOH 9:1).

#### 7-((3As,4R,6R,6aR)-6-(Azidomethyl)-2,2-dimethyltetrahydro-4*H*-cyclopenta­[d]­[1,3]­dioxol-4-yl)-*N*-methyl-7*H*-pyrrolo­[2,3-*d*]­pyrimidin-4-amine (**si45**)

Compound **si44** (1.30 g, 4.04 mmol) was dissolved in dry 1,4-dioxane (16.20 mL). The solution was cooled down to 0 °C. Then, DPPA (1.76 mL, 8.09 mmol) and DBU (1.83 mL, 12.13 mmol) were added to the cooled solution. The reaction mixture was stirred magnetically overnight at rt. TLC monitored full consumption of the SM. Then, NaN_3_ (1.33 g, 20.21 mmol) and 15-crown-5 (0.84 mL, 4.04 mmol) were added, and the mixture was heated to 110 °C. At this temperature, the mixture was stirred for 6 h. After this time, the mixture was allowed to cool down to rt and the organic solvent was evaporated under vacuum. Afterward, water was added, and the aqueous phase was extracted with EtOAc. The combined organic layers were washed with brine, dried over sodium sulfate, and concentrated under reduced pressure. The obtained residue was purified over silica (cyclohexane/EtOAc; 20–80%) afford the product as yellowish foam (0.61 g, 44%). ^1^H NMR (400 MHz, DMSO-*d*_6_): δ 8.14 (s, 1H, H2), 7.47 (d, ^3^ *J* = 4.6 Hz, 1H, N*H*), 7.29 (d, ^3^ *J* = 3.6 Hz, 1H, H8), 6.56 (d, ^3^ *J* = 3.6 Hz, 1H, H7), 4.95 (dt, ^3^ *J* = 12.4 Hz and ^3^ *J* = 6.4 Hz, 1H, H1’), 4.89 (t, ^3^ *J* = 6.4 Hz, 1H, H2’), 4.51 (dd, ^3^ *J* = 7.2 Hz and ^3^ *J* = 5.2 Hz, 1H, H3′), 3.57–3.44 (m, 2H, H5′), 2.95 (d, ^3^ *J* = 4.6 Hz, 3H, N–C*H*_3_), 2.35–2.20 (m, 2H, H4’, H6’_A_), 2.12–1.97 (m, 1H, H6’_B_), 1.47 (s, 3H, C*H*_3_), 1.22 (s, 3H, C*H*_3_); ^13^C NMR (101 MHz, DMSO-*d*_6_) δ 151.4, 129.8, 121.8, 120.1, 120.0, 112.8, 83.7, 81.4, 59.4, 52.6, 43.1, 35.1, 27.3, 25.1 APCI-MS­(+): *m*/*z* 344.4 [M + H]^+^; R*_f_*: 0.65 (CH_2_Cl_2_/MeOH 9:1).

#### *tert*-Butyl (7-((3aS,4R,6R,6aR)-6-(Azidomethyl)-2,2-dimethyltetrahydro-4*H*-cyclopenta­[d] [1,3]­dioxol-4-yl)-7*H*-pyrrolo­[2,3-*d*]­pyrimidin-4-yl)­(methyl)­carbamate (**si46**)

The azido-nucleoside **si45** (0.60 g, 1.73 mmol) was dissolved in dry THF (7.50 mL) and cooled down in an ice-bath. To this cooled solution, NaH (60% in mineral oil, 0.14 g, 3.46 mmol) was added portion wise. The mixture was allowed to warm up rt and stirred for 30 min before the mixture was cooled down again to 0 °C. Then, di*tert*-butyl decarbonate (0.40 mL, 1.73 mmol) was added portion wise at 0 °C. The mixture was stirred magnetically at rt for 6 h. TLC indicated slow conversion. At this point, the mixture was cooled down and 8 eq. NaH were added. The reaction mixture was stirred for 30 min at rt before the mixture was again cooled down to 0 °C. Then, 3 eq. di*tert*-butyl dicarbonate were added and the mixture was stirred for 72 h at rt. At this point, TLC monitored still SM. Therefore, the mixture was cooled down and 5 eq. NaH were added and the mixture was stirred at rt for 30 min. Then, the mixture was cooled down again and 2 eq. di*tert*-butyl decarbonate were added. The reaction mixture was stirred for further 48 h at ambient temperature. After this time almost all SM was converted. Then, the mixture was cooled down and quenched carefully with water (highly exothermic reaction). Then, the aqueous phase was extracted with CH_2_Cl_2_. The combined organic layers were washed with brine, dried over sodium sulfate, and concentrated under vacuum. The crude product was purified by flash chromatography (CH_2_Cl_2_/MeOH, 0–10%) to afford the product as grayish foam (0.57 g, 74%). ^1^H NMR (400 MHz, DMSO-*d*_6_) δ 8.62 (s, 1H, H2), 7.72 (d, *J* = 3.7 Hz, 1H, H6, 6.45 (d, *J* = 3.7 Hz, 1H, H5), 5.12 (dt, *J* = 12.1, 6.4 Hz, 1H, H1́), 4.92 (dd, *J* = 7.2, 6.2 Hz, 1H, H2́), 4.53 (dd, *J* = 7.3, 5.2 Hz, 1H, H3́), 3.53 (ddt, *J* = 19.4, 12.4, 6.6 Hz, 2H, CH_2_, H5́), 3.34 (s, 3H, CH_3_, NMe), 2.40–2.26 (m, 2H, H4́, CH_2_, cyclopentane), 2.16–2.06 (m, 1H, CH_2_, cyclopentane), 1.49 (s, 3H, CH_3_, acetonide), 1.44 (s, 9H, CH_3_, *t*-butyl), 1.23 (s, 3H, CH_3_, acetonide). APCI-MS­(+): *m*/*z* 388.5 [M–*t*Bu+2H]^+^, 444.6 [M + H]^+^; R*_f_*: 0.78 (CH_2_Cl_2_/MeOH 9:1).

#### *tert*-Butyl (7-((3aS,4R,6R,6aR)-6-(aminomethyl)-2,2-dimethyltetrahydro-4*H*-cyclopenta [d]­[1,3]­dioxol-4-yl)-7*H*-pyrrolo­[2,3-*d*]­pyrimidin-4-yl)­(methyl)­carbamate (**si47**)

Compound **si46** (0.56 g, 1.25 mmol) was dissolved in a mixture of EtOAc/MeOH (4.20 mL, 1:1) under nitrogen atmosphere. Then, the solution was degassed before palladium on activated charcoal moistened with water (0.10 g, 0.13 mmol) was added. The suspension was degassed and purged with H_2_ from a storage vessel. The reaction mixture was stirred for 17 h at ambient temperature. Then, the mixture was purged with nitrogen and filtered over Celite. The filter cake was rinsed with MeOH and the filtrate was concentrated under reduced pressure. The obtained residue was purified over silica (CH_2_Cl_2_/MeOH, 0–20%) to afford the desired product as grayish foam (0.25 g, 48%). ^1^H NMR (400 MHz, DMSO-*d*_6_) δ 8.63 (s, 1H, H2), 7.76 (d, *J* = 3.7 Hz, 1H, H6), 6.45 (d, *J* = 3.7 Hz, 1H, H5), 5.09 (dt, *J* = 12.1, 6.7 Hz, 1H, H1́), 4.91–4.86 (m, 1H, H2́), 4.51 (dd, *J* = 7.2, 4.9 Hz, 1H, H3́), 3.35 (s, 3H, CH_3_, NMe), 2.78–2.62 (m, 2H, CH_2_, H5́), 2.31–2.23 (m, 1H, CH_2_, cyclopentane), 2.19–2.08 (m, 1H, H4́), 2.08–1.99 (m, 1H, CH_2_, cyclopentane), 1.48 (s, 3H, CH_3_, acetonide), 1.45 (s, 9H, CH_3_, *t*-butyl), 1.23 (s, 3H, CH_3_, acetonide). APCI-MS­(+): *m*/*z* 318.5 [M–Boc+2H]^+^, 362.5 [M–*t*Bu+H]^+^, 418.7 [M + H]^+^; R*_f_*: 0.22 (CH_2_Cl_2_/MeOH 9:1).

#### *tert*-Butyl (S)-4-((((3aR,4R,6R,6aS)-6-(4-((*tert*-butoxycarbonyl)­(methyl)­amino)-7*H*-pyrrolo­[2,3-*d*]­pyrimidin-7-yl)-2,2-dimethyltetrahydro-4*H*-cyclopenta­[d]­[1,3]­dioxol-4-yl)­methyl)­amino)-2-((*tert*-butoxycarbonyl)­amino)­butanoate (**si48**)

To a stirred solution of **si47** (400 mg, 0.96 mmol, 1.1 equiv) and **si6** (240 mg, 0.87 mmol, 1 equiv) in dry DCE (7.5 mL) was added AcOH (55 μL, 0.96 mmol, 1.1 equiv). The solution was stirred for 3 h at rt, then NaBH­(OAc)_3_ (484 mg, 2.26 mmol, 2.6 equiv) was added and the mixture was stirred for 4 h at rt. After completion, the reaction was quenched by the addition of a 5% aq. NaHCO_3_ solution and the phases were separated. The aqueous phase was then extracted three times with CH_2_Cl_2_ and the combined organic phases once with brine. Drying over Na_2_SO_4_, filtration and evaporation afforded the subjected crude product to silica gel column chromatography eluting with CH_2_Cl_2_/MeOH (99.4:0.6–95:5) to afford the secondary amine **si48** (180.1 mg, 0.27 mmol, 31%) as a beige foam. C_34_H_54_N_6_O_8_ (674.84 g/mol). ^1^H NMR (400 MHz; DMSO-*d*_6_): δ 8.61 (s, 1H, H2), 7.74 (d, *J* = 3.8 Hz, 1H, H8), 7.34 (d, *J* = 7.2 Hz, 1H, N*H* Carbamate), 6.44 (d, *J* = 3.8 Hz, 1H, H7), 5.08 (dt, *J* = 12.4 Hz and *J* = 6.2 Hz, 1H, H1’), 4.90 (t, *J* = 6.6 Hz, 1H, H2’), 4.48–4.45 (m, 1H, H3′), 3.95–3.86 (m, 1H, Hα), 2.76–2.65 (m, 1H, H5′_A_), 2.64–2.58 (m, 1H, Hγ_A_), 2.56–2.50 (m, 2H, H5′_B_, Hγ_B_), 2.33–2.29 (m, 1H, H6’_A_), 2.25–2.19 (m, 1H, H4’), 2.07–1.98 (m, 1H, H6’_B_), 1.77–1.72 (m, 1H, Hβ_A_), 1.71–1.64 (m, 1H, Hβ_B_), 1.47 (s, 3H, C*H*_3_), 1.44 (s, 9H, C*H*_3_ *t*Bu), 1.38 (s, 9H, C*H*_3_ *t*Bu), 1.28 (s, 9H, C*H*_3_ *t*Bu), 1.22 (s, 3H, C*H*_3_); ^13^C NMR (101 MHz, DMSO-*d*_6_) δ 171.9, 155.4, 154.1, 152.8, 151.6, 149.8, 126.1, 112.4, 111.6, 101.2, 83.5, 82.1, 81.1, 80.0, 77.8, 59.9, 53.0, 52.0, 46.0, 43.3, 35.7, 34.7, 30.5, 28.0, 27.8, 27.7, 27.6, 27.4, 25.1; R*_f_*: 0.46 (CH_2_Cl_2_/MeOH 9:1).

#### *tert*-Butyl (S)-4-((((3aR,4R,6R,6aS)-6-(4-((*tert*-butoxycarbonyl)­(methyl)­amino)-7*H*-pyrrolo­[2,3-*d*]­pyrimidin-7-yl)-2,2-dimethyltetrahydro-4*H*-cyclopenta­[d]­[1,3]­dioxol-4-yl)­methyl)­(3-((*tert*-butoxycarbonyl)­(phenethyl)­amino)­propyl)­amino)-2-((*tert*-butoxycarbonyl)­amino)­butanoate (**si49a**)

The reductive amination for compound **si49a** followed the general procedure for the second reductive amination on carbasugar derivatives. As aldehyde was used **si13a**. Yield: 101.5 mg, 0.108 mmol, 54%. HR-MS (ESI): calcd. for C_50_H_78_N_7_O_10_ [M + H]^+^: 936.5805, found: 936.5796; R*_f_*: 0.85 (CH_2_Cl_2_/MeOH 9:1).

#### (S)-2-Amino-4-((((1R,2R,3S,4R)-2,3-dihydroxy-4-(4-(methylamino)-7*H*-pyrrolo­[2,3-*d*] pyrimidin-7-yl)­cyclopentyl)­methyl)­(3-(phenethylamino)­propyl)­amino)­butanoic acid (**6**)

Deprotection of **si49a** followed the general procedure for deprotection. Yield: 64 mg, 0.097 mmol, 100% (1 TFA salt). ^1^H NMR (400 MHz; DMSO-*d*_6_): δ 9.44 (bs, 1H, CH_3_–N*H*), 8.91 (bs, 2H, N*H*_2_^+^), 8.35 (s, 1H, H2), 7.58 (s, 1H, H8), 7.39–7.32 (m, 2H, *m*-H), 7.29–7.25 (m, 3H, *o*-H, *p*-H), 6.91 (s, 1H, H7), 5.00–4.89 (m, 1H, H1’), 4.15 (t, ^3^ *J* = 6.4 Hz, 1H, H2’), 4.02 (t, ^3^ *J* = 6.4 Hz, 1H, Hα), 3.87 (t, ^3^ *J* = 5.8 Hz, 1H, H3′), 3.37–3.18 (m, 9H, H5′, Hγ, H1’’, C*H*_2_–N), 3.14–2.99 (m, 5H, H3′’, C*H*_3_), 2.97–2.86 (m, 2H, C*H*_2_–Ph), 2.40–2.35 (m, 2H, H4’, H6’_A_), 2.28–2.21 (m, 1H, Hβ_A_), 2.18–2.09 (m, 1H, Hβ_B_), 2.09–1.96 (m, 2H, H2’’), 1.68–1.61 (m, 1H, H6’_B_); HR-MS (ESI): calcd. for C_28_H_42_N_7_O_4_ [M + H]^+^: 540.3293, found: 540.3293; HPLC: *t*_R_ = 8.270 min (Method C), UV-purity at 254 nm: 94.5%.

#### *tert*-Butyl (S)-4-((3-((*tert*-butoxycarbonyl)­(4-phenoxyphenethyl)­amino)­propyl) (((3aR,4R,6R,6aS)-6-(4-((*tert*-Butoxycarbonyl)­(methyl)­amino)-7*H*-pyrrolo­[2,3-*d*] pyrimidin-7-yl)-2,2-dimethyltetrahydro-4*H*-cyclopenta­[d]­[1,3]­dioxol-4-yl)­methyl) amino)-2-((*tert*-butoxycarbonyl)­amino)­butanoate (**si49b**)

The reductive amination for compound **si49b** followed the general procedure for the second reductive amination on carbasugar derivatives. As aldehyde was used **si13b**. Yield: 30.2 mg, 0.029 mmol, 37%. HR-MS (ESI): calcd. for C_56_H_82_N_7_O_11_ [M + H]^+^: 1028.6067, found: 1028.6046; R*_f_*: 0.83 (CH_2_Cl_2_/MeOH 9:1).

#### (S)-2-Amino-4-((((1R,2R,3S,4R)-2,3-dihydroxy-4-(4-(methylamino)-7*H*-pyrrolo­[2,3-*d*] pyrimidin-7-yl)­cyclopentyl)­methyl)­(3-((4-phenoxyphenethyl)­amino)­propyl)­amino) Butanoic acid (**7a**)

Deprotection of the compound **si49b** followed the general procedure for deprotection. Yield: 21 mg, 0.024 mmol, 100% (2 TFA salts). ^1^H NMR (400 MHz; DMSO-*d*_6_): δ 9.57 (bs, 3H, N*H*_3_^+^), 8.91 (bs, 2H, N*H*_2_^+^), 8.36 (s, 1H, H2), 7.60 (s, 1H, H8), 7.39 (t, ^3^ *J* = 7.6 Hz, 2H, *m*-H’), 7.28 (d, ^3^ *J* = 8.4 Hz, 2H, *m*-H), 7.14 (t, ^3^ *J* = 7.2 Hz, 1H, *p*-H’), 7.00–6.97 (m, 4H, *o*-H, *o*-H’), 6.92 (s, 1H, H7), 5.01–4.89 (m, 1H, H1’), 4.14 (t, ^3^ *J* = 6.2 Hz, 1H, H2’), 4.04 (t, ^3^ *J* = 6.2 Hz, 1H, Hα), 3.94–3.85 (m, 1H, H3′), 3.47–3.29 (m, 4H, H5′, Hγ), 3.23–3.17 (m, 4H, H1’’, C*H*_2_–N), 3.14–3.00 (m, 5H, H3′’, C*H*_3_), 2.96–2.84 (m, 2H, C*H*_2_–Ph), 2.43–2.35 (m, 2H, H4’, H6’_A_), 2.30–2.18 (m, 1H, Hβ_A_), 2.16–2.03 (m, 3H, H2’’, Hβ_B_), 1.74–1.57 (m, 2H, H6’_B_); HR-MS (ESI): calcd. for C_34_H_46_N_7_O_5_ [M + H]^+^: 632.3555, found: 632.3553. Purified by preparative HPLC (Method K with adjusted flow rate of 16.25 mL/min). HPLC: *t*_R_ = 10.804 min (Method C), UV-purity at 254 nm: 97.8%.

#### *tert*-Butyl (S)-4-((3-((*tert*-butoxycarbonyl)­(4-(4-chlorophenoxy)-3-fluorophenethyl) amino)­propyl)­(((3aR,4R,6R,6aS)-6-(4-((*tert*-butoxycarbonyl)­(methyl)­amino)-7*H*-pyrrolo­[2,3-*d*]­pyrimidin-7-yl)-2,2-dimethyltetrahydro-4*H*-cyclopenta­[d]­[1,3]­dioxol-4-yl)­methyl)­amino)-2-((*tert*-butoxycarbonyl)­amino)­butanoate (**si49c**)

Aldehyde **si61** was dissolved in dry DCE (0.90 mL) under nitrogen atmosphere. Then, AcOH (7.00 mL, 0.13 mmol) was added and then a solution of the 2° amine **si48** (0.09 g, 0.13 mmol) was slowly added. The resulted mixture was stirred at ambient temperature for 3.5 h. TLC (CH_2_Cl_2_/MeOH, 3%) monitored almost complete consumption of the aldehyde. After 3.5 h, NaBH­(OAc)_3_ (0.06 g, 0.30 mmol) was added portion wise and stirred for additional 3 h. Then, the mixture was diluted with aqueous 5% bicarbonate solution and the mixture was extracted with CH_2_Cl_2_. The combined organic layers were washed with brine, dried over sodium sulfate, and concentrated under rotatory evaporation. The obtained residue was deprotected in the next step.

#### (s)-2-Amino-4-((3-((4-(4-chlorophenoxy)-3-fluorophenethyl)­amino)­propyl) (((1R,2R,3S,4R)-2,3-dihydroxy-4-(4-(methylamino)-7*H*-pyrrolo­[2,3-*d*]­pyrimidin-7-yl)­cyclopentyl)­methyl)­amino)­butanoic Acid (**7b**)

Compound **si49c** was suspended in a mixture of TFA and water (4:1). The mixture was stirred for 4 h, at ambient temperature. After 4 h, the mixture was concentrated under reduced pressure. Purified by semipreparative HPLC (H_2_O/MeCN; 0.05% TFA) to afford the product as white solid (TFA-salt, 0.02 g, 59%). Analytical data: ^1^H NMR (400 MHz, DMSO-*d*_6_) δ 8.90 (s, 3H, NH_3_^+^), 8.34 (s, 1H, H2), 7.60–7.53 (m, 1H, H6), 7.42 (d, *J* = 9.1 Hz, 2H, ḿ́), 7.36 (dd, *J* = 11.9, 1.9 Hz, 1H, ḿ), 7.22 (t, *J* = 8.4 Hz, 1H, ó-2), 7.17–7.12 (m, 1H, o-6), 6.97 (d, *J* = 9.0 Hz, 2H, ó́), 6.92–6.85 (m, 1H, H5), 4.98–4.89 (m, 1H, H1́), 4.15 (t, *J* = 6.4 Hz, 1H, H2́), 4.04–3.96 (m, 1H, Hα), 3.87 (t, *J* = 5.6 Hz, 1H, H3́), 3.37–3.11 (m, 8H, CH_2_, H5́, Hγ, CH_2_–NH_2_^+^, H3́́), 3.11–3.00 (m, 5H, CH3, H1́́), 2.95 (t, *J* = 8.0 Hz, 2H, CH_2_–Ph), 2.40–2.30 (m, 2H, H4́, H6́-axial), 2.27–2.10 (m, 2H, CH_2_, Hβ), 2.06–1.96 (m, 2H, CH_2_, H2́́), 1.69–1.59 (m, 1H, H6́-equatorial). Purified by semipreparative HPLC. HPLC: *t*_R_ = 14.333 min (Method G). UV-purity at 210 nm: 94.5%

#### 2-Fluoro-4-(2-hydroxyethyl)­phenol (**si55**)

To a heat dried three-necked round-bottom flask equipped with a thermometer and a dropping funnel, NaBH_4_ (2.65 g, 70.00 mmol)) was added in dry THF (60.00 mL). To this mixture, Me_2_SO_4_ (6.64 mL, 70.00 mmol) was slowly added at 0 °C and stirred for 1 h at 0 °C. Then, the mixture was allowed to warm up to rt and stirred for 3 h. Afterward, a solution of 2-(3-fluoro-4-hydroxyphenyl) acetic acid (5.95 g, 35.00 mmol) and B­(OMe)_3_ (7.91 mL, 70.00 mmol) in dry THF (10 mL) were added slowly added at rt while cooling the flask with an ice-bath. The mixture was stirred overnight at rt. TLC (100% EtOAc) monitored the formation of a new spot. The mixture was cooled down in an ice-bath and stirred vigorously while water was carefully added. Then, the organic solvent was evaporated under reduced pressure. The aqueous phase was extracted three times with EtOAc. The combined organic layers were washed three times with saturated sodium bicarbonate solution and three times with brine. The separated organic layer was dried over sodium sulfate and concentrated under reduced pressure. ^1^H NMR (400 MHz, DMSO-d6) δ 9.53 (s, Ar–OH 1H), 6.98 (dd, *J* = 12.7, 1.8 Hz, 1H, Ar–H), 6.89–6.77 (m, 2H- Ar–H), 4.61 (t, *J* = 5.1 Hz, 1H, OH), 3.55 (td, *J* = 7.0, 5.1 Hz, 2H, CH_2_), 2.61 (t, *J* = 7.0 Hz, 2H, CH_2_). APCI-MS­(+): *m*/*z* 157.0 [M + H]^+^.

#### 2-(4-(4-Chlorophenoxy)-3-fluorophenyl)­ethan-1-ol (**si56**)

In a heat-dried three-necked round-bottom flask, 4-chlorophenylboronic acid (3.05 g, 19.50 mmol) and **si55** (1.0 g, 6.50 mmol) were added in dry C_2_H_2_Cl_2_ (40.60 mL). Afterward, dry pyridine (1.51 mL, 19.50 mmol), anhydrous copper acetate (1.77 g, 9.75 mmol), and 4 Å molecular sieves (1.12 g) were added to the mixture. The reaction mixture was stirred for 48 h at rt. The mixture was filtered over Celite and the cake was rinsed several times with CH_2_Cl_2_. The filtrate was concentrated under vacuum to complete dryness. The obtained residue was purified over silica (cyclohexane/EtOAc; 10–80%) to afford the pure product (0.93 g, 54%). ^1^H NMR (400 MHz, DMSO-*d*_6_) δ 7.46–7.37 (m, 2H, H 2,6), 7.28 (dd, *J* = 12.1, 1.9 Hz, 1H, 6́H), 7.15 (t, *J* = 8.3 Hz, 1H, 2́H), 7.10 (dd, *J* = 8.5, 1.9 Hz, 1H, 5́H), 6.99–6.94 (m, 2H, 2,6 H), 4.69 (t, *J* = 5.2 Hz, 1H, OH), 3.64 (td, *J* = 6.8, 5.2 Hz, 2H, CH_2_), 2.75 (t, *J* = 6.8 Hz, 2H, CH_2_). APCI-MS­(+): *m*/*z* 265.1/268.1 [M + H]^+^.

#### 4-(4-Chlorophenoxy)-3-fluorophenethyl 4-methylbenzenesulfonate (**si57**)

The compound **si56** (1.28 g, 4.80 mmol) was dissolved in dry CH_2_Cl_2_ (24.00 mL). Then, TsCl (0.92 g, 4.80 mmol) was added. Afterward, dry pyridine (1.16 mL, 14.34 mmol) and DMAP (0.06 g, 0.48 mmol) were added 0 °C. The solution was stirred at rt overnight. TLC (cyclohexane/EtOAc; 20%) monitored no full conversion. In addition, dry pyridine (0.58 mL, 7.20 mmol) was added and stirred at rt until complete conversion was observed via TLC. After 6 h, the mixture was diluted with saturated bicarbonate solution and extracted three times with CH_2_Cl_2_. The combined organic layers were washed with brine, dried over sodium sulfate, and concentrated under reduced pressure. The crude was purified by flash chromatography (cyclohexane/EtOAc; 0–80%) to afford the product as a colorless oil (1.33 g, 75%). ^1^H NMR (400 MHz, DMSO-*d*_6_) δ 7.73–7.68 (m, 2H, *o*-tosyl), 7.48–7.38 (m, 4H, *m*-tosyl, H3/5), 7.19 (dd, *J* = 12.0, 2.0 Hz, 1H, H5́), 7.10 (t, *J* = 8.4 Hz, 1H, H2́), 7.02 (dd, *J* = 8.3, 1.3 Hz, 1H, H6́), 7.02–6.93 (m, 2H, H2/6), 4.28 (t, *J* = 6.3 Hz, 2H, CH_2_), 2.92 (t, *J* = 6.3 Hz, 2H, CH_2_), 2.41 (s, 3H, CH_3_). APCI-MS­(+): *m*/*z* 420.0/422.1 [M + H]^+^.

#### 3-((*tert*-Butyldiphenylsilyl)­oxy)-*N*-(4-(4-chlorophenoxy)-3-fluorophenethyl)­propan-1-amine (**si58**)

A solution of **si57** (1.05 g, 2.51 mmol) in dry DMF (6.25 mL) was added to a solution of **si65** (1.18 g, 3.76 mmol) in dry DMF (6.25 mL) at rt. Then, CsCO_3_ (1.77 g, 5.01 mmol) was added and the suspension was heated to 80 °C and stirred at this temperature for 5 h. TLC (cyclohexane/EtOAc; 50%) monitored full consumption of **si57**. Then, the reaction was allowed to cool down to rt and was diluted with saturated bicarbonate solution. The aqueous phase was extracted three times with EtOAc. Afterward, the combined organic layers were washed with brine, dried over sodium sulfate, and concentrated under reduced pressure. The obtained residue was purified by flash chromatography (cyclohexane/EtOAc; 0–60%) to afford the pure product. ^1^H NMR (400 MHz, DMSO-*d*_6_) δ 7.64–7.60 (m, 2H, Ar–H), 7.51–7.35 (m, 8H, Ar–H, H3/5), 7.26 (dd, *J* = 12.0, 1.9 Hz, 1H, H5́), 7.15–7.08 (m, 1H, H2́), 7.06 (dd, *J* = 8.4, 1.8 Hz, 1H, H6́), 7.00–6.91 (m, 2H, H2/6), 3.71 (t, *J* = 6.3 Hz, 2H, CH_2_, H5́́), 2.77–2.66 (m, 4H, CH_2_, H1́́/H2́́), 2.63 (t, *J* = 6.8 Hz, 2H, CH_2_, H3́́), 1.67 (p, *J* = 6.5 Hz, 2H, CH_2_, H4́́), 1.00 (s, 9H, CH_3_, t-butyl). APCI-MS­(+): *m*/*z* 561.8/563.8 [M + H]^+^.

#### 3-((4-(4-Chlorophenoxy)-3-fluorophenethyl)­amino)­propan-1-ol (**si59**)

To a solution of **si58** (0.67 g, 1.19 mmol) in dry THF (5.90 mL), TBAF (0.65 mL, 2.38 mmol) was added dropwise. Then, the solution was stirred magnetically at rt for 5 h. TLC (CH_2_Cl_2_/MeOH; 10%) indicated full conversion. After 5h, the organic solvent was evaporated under vacuum and the obtained residue was purified over silica (CH_2_Cl_2_/MeOH; 0–10%) to afford the desired product (0.23 g, 60%). ^1^H NMR (400 MHz, DMSO-*d*_6_) δ 7.44–7.39 (m, 2H, H2/6), 7.29 (dd, *J* = 12.1, 1.9 Hz, 1H, H5́), 7.15 (t, *J* = 8.3 Hz, 1H, H2́), 7.09 (dd, *J* = 8.2, 1.8 Hz, 1H, H6́), 6.99–6.95 (m, 2H, H3/5), 3.45 (t, *J* = 6.3 Hz, 2H, CH_2_, H5́́), 2.82–2.68 (m, 4H, CH_2_, H1́́/2́́), 2.61 (t, *J* = 6.9 Hz, 2H, CH_2_, H3́́), 1.56 (p, *J* = 6.6 Hz, 2H, CH_2_, H4́́). APCI-MS­(+): *m*/*z* 324.0/326.0 [M + H]^+^.

#### *tert*-Butyl (4-(4-chlorophenoxy)-3-fluorophenethyl)­(3-hydroxypropyl)­carbamate (**si60**)

Triethylamine (0.14 mL, 1.02 mmol) was added to a solution of **si59** (0.22 g, 0.68 mmol) in dry CH_2_Cl_2_ (3.40 mL). The solution was cooled down to 0 °C in an ice-bath. Afterward, di-*tert*-butyl dicarbonate (0.17 mL, 0.75 mmol) was added portion wise. Then, the reaction mixture was allowed to warm up to rt and stirred for 5 h until complete conversion was monitored by TLC (cyclohexane/EtOAc; 50%). The mixture was diluted with saturated sodium bicarbonate solution and the organic phase was separated. The aqueous phase was extracted three times with CH_2_Cl_2_. The combined organic layers were washed with brine, dried over sodium sulfate, and concentrated over vacuum. The crude was purified by flash chromatography (cyclohexane/EtOAc; 10–80%) to afford a white solid (0.26 g, 89%). ^1^H NMR (400 MHz, DMSO-*d*_6_) δ 7.44–7.38 (m, 2H, H2/6), 7.26 (dd, *J* = 11.9, 2.0 Hz, 1H, H5́), 7.17 (t, *J* = 8.5 Hz, 1H, H2́), 7.06 (d, *J* = 8.3 Hz, 2H, H6́), 6.99–6.94 (m, 2H, H3/5), 4.43 (t, *J* = 5.0 Hz, 1H, OH), 3.42–3.35 (m, 4H, CH_2_, H2́́/5́́), 3.21 – 3.10 (m, 2H, CH_2_, H3́́), 2.79 (t, *J* = 7.2 Hz, 2H, CH_2_, H1́́), 1.60 (p, *J* = 6.5 Hz, 2H, CH_2_, H4́́), 1.35 (s, 9H, CH_3_, t-butyl). APCI-MS­(+): *m*/*z* 324.2/326.2 [M + H]^+^ without Boc-group.

#### *tert*-Butyl (4-(4-chlorophenoxy)-3-fluorophenethyl)­(3-oxopropyl)­carbamate (**si61**)

To a solution of **si60** (0.06 g, 0.14 mmol) in dry DMSO (0.70 mL), triethylamine (0.04 mL, 0.28 mmol) was added at rt. Then, a solution of sulfur trioxide pyridine complex (0.05 g, 0.28 mmol) in dry DMSO (0.70 mL) was added while vigorously stirring. TLC (cyclohexane/EtOAc; 50%) monitored conversion. After 1 h, the solution was quenched with water at 0 °C and the aqueous phase was extracted with CH_2_Cl_2_. The combined organic layers were washed with brine, dried over sodium sulfate, and concentrated under reduced pressure. The crude was purified over silica (cyclohexane/EtOAc; 10–60%) to afford the product (0.06 g, 100%) as a yellowish resin. ^1^H NMR (400 MHz, DMSO-*d*_6_) δ 9.66 (s, 1H, R-CHO), 7.45–7.37 (m, 2H, H2/6), 7.27 (dd, *J* = 12.0, 1.8 Hz, 1H, H5), 7.17 (t, *J* = 8.5 Hz, 1H, H2), 7.07 (dd, *J* = 8.0, 1.3 Hz, 1H, H6), 3.45–3.36 (m, 4H, CH_2_, H2́́/3́́), 2.78 (t, *J* = 7.2 Hz, 2H, CH_2_, H1́́), 2.63 (td, *J* = 6.6, 1.7 Hz, 2H, CH_2_, H4́́), 1.33 (s, 9H, CH_3_, t-butyl). APCI-MS­(+): *m*/*z* 322.1/324.1 [M + H]^+^ without Boc-group.

#### Ethyl 3-(4-(4-chlorophenoxy)-3-fluorophenethoxy)­propanoate (**si62**)

To a solution of 2-(4-(4-chlorophenoxy)-3-fluorophenyl)­ethanol **si56** (300 mg, 1.1 mmol) in acetonitrile was added ethyl acrylate (564 mg, 5.6 mmol) and Cs_2_CO_3_ (715 mg, 2.2 mmol), then the solution was stirred at 35 ^o^ C overnight. The mixture was concentrated and the residue was purified by silica gel flash chromatography (PE:EA = 25:1) to give compound **si62** (400 mg, yield: 97%) as colorless oil. ^1^H NMR (300 MHz, CD_3_Cl): δ = 7.32–7.29 (m, 2H), 7.13–6.92 (m, 5H), 4.19 (q, *J* = 6.9 Hz, 2H), 3.80–3.70 (m, 4H), 2.90 (t, *J* = 6.6 Hz, 2H), 2.62 (t, *J* = 6.3 Hz, 2H), 1.30 (t, *J* = 6.9 Hz, 3H).

#### 3-(4-(4-Chlorophenoxy)-3-fluorophenethoxy)­propan-1-ol (**si63**)

To a solution of **si62** (400 mg, 1.1 mmol) in THF (10 mL) was added LiAlH_4_ (124 mg, 3.3 mmol) and the solution was stirred at room temperature for 1 h. The reaction solution was quenched with aq. NH_4_Cl (20 mL) and the reaction mixture was diluted with EtOAc (20 mL). The organic phase was washed with H_2_O (10 mL ×3), dried over Na_2_SO_4_ and concentrated to dryness. The residue was purified by silica gel flash chromatography (PE:EtOAc = 2:1) to give compound **si63** (330 mg, yield: 93%) as colorless oil.

#### 3-(4-(4-Chlorophenoxy)-3-fluorophenethoxy)­propanal (**si64**)

To a solution of **si63** (330 mg, 1.0 mmol) in DCM (5 mL) was added Dess-Martin periodinane (636 mg, 1.5 mmol) and the solution was stirred at room temperature overnight. The reaction solution was concentrated to dryness. The residue was purified by silica gel flash chromatography (PE:EtOAc = 6:1) to give compound **si64** (270 mg, yield: 82%) as colorless oil. ^1^H NMR (300 MHz, CD_3_Cl): δ = 9.82 (t, *J* = 1.5 Hz, 1H), 7.32–7.29 (m, 2H), 7.11–6.92 (m, 5H), 3.84 (t, *J* = 6.0 Hz, 2H), 3.72 (t, *J* = 6.9 Hz, 2H), 2.90 (t, *J* = 6.6 Hz, 2H), 2.75–2.70 (m, 2H).

#### 3-((*tert*-Butyldiphenylsilyl)­oxy)­propan-1-amine (**si65**)

3-Amino-1-propanol (2.07 mL, 27.29 mmol) was dissolved in dry CH_2_Cl_2_ (91.00 mL) and cooled down in an ice-bath. Then, TBDPS-Cl (4.73 mL, 18.19 mmol) was added dropwise at 0 °C. Afterward, triethylamine (3.78 mL, 27.29 mmol) was added. The solution was allowed to warm up to rt and the reaction was stirred magnetically at rt overnight. The reaction mixture was diluted with saturated bicarbonate solution and extracted with CH_2_Cl_2_. The combined organic layers were washed with brine, dried over sodium sulfate, and concentrated over vacuum. The obtained crude was purified over silica (CH_2_Cl_2_/MeOH; 0–20%) to afford the pure product as a colorless oil (3.42 g, 60%). ^1^H NMR (400 MHz, DMSO-*d*_6_) δ 7.67–7.58 (m, 4H, *o*–Ar-H), 7.52–7.39 (m, 6H, *m,p*–Ar-H), 3.71 (t, *J* = 6.3 Hz, 2H, CH_2_, H3), 2.64 (t, *J* = 6.8 Hz, 2H, CH_2_, H1), 1.61 (p, *J* = 6.5 Hz, 2H, CH_2_, H2), 1.00 (s, 9H, CH_3_, t-butyl). APCI-MS­(+): *m*/*z* 314.0 [M + H]^+^.

#### *tert*-Butyl (4-(4-Chlorophenoxy)-3-fluorophenethyl)­(3-((((3aR,4R,6R,6aS)-2,2-dimethyl-6-(4-(methylamino)-7*H*-pyrrolo­[2,3-*d*]­pyrimidin-7-yl)­tetrahydro-4*H*-cyclopenta­[d]­[1,3] dioxol-4-yl)­methyl)­amino)­propyl)­carbamate (**si52a**)

A mixture of compound **si61** (100 mg, 0.23 mmol) and **si51** (60 mg, 0.19 mmol) in EtOH (10 mL) was stirred for 1 h at rt. Then NaBH_3_CN (29 mg, 0.46 mmol) was added. The resulting mixture was stirred for additional 12 h at rt. Solvent was removed and the residue was purified by flash chromatography (5% MeOH in DCM) to give compound **si52a** (45 mg, 27% yield) as a white gum. MS Calcd: 723; MS Found: 723 [M + H]^+^

#### 7-((3as,4R,6R,6aR)-6-(((3-(4-(4-Chlorophenoxy)-3-fluorophenethoxy)­propyl)­amino) methyl)-2,2-dimethyltetrahydro-4*H*-cyclopenta­[d]­[1,3]­dioxol-4-yl)-*N*-methyl-7*H*-pyrrolo­[2,3-*d*]­pyrimidin-4-amine (**si52b**)

A solution of **si64** (50 mg, 0.16 mmol), **si51** (50 mg, 0.16 mmol) in MeOH (3 mL) was stirred at room temperature for 30 min. Then to the solution was added NaBH_3_CN (30 mg, 0.48 mmol). The solution was stirred at room temperature for 16 h. The solution was quenched with water (2 mL) and concentrated. The crude was purified by reverse phase flash (MeCN/H_2_O) to afford **si52b** (20 mg, yield: 14%) as a yellow oil. MS Calcd: 624.3; MS Found: 624.0 [M+H^+^].

#### Ethyl (S)-4-((3-((*tert*-butoxycarbonyl)­(4-(4-chlorophenoxy)-3-fluorophenethyl)­amino) propyl)­(((3aR,4R,6R,6aS)-2,2-dimethyl-6-(4-(methylamino)-7*H*-pyrrolo­[2,3-*d*] pyrimidin-7-yl)­tetrahydro-4*H*-cyclopenta­[d]­[1,3]­dioxol-4-yl)­methyl)­amino)-2-((*tert*-butoxycarbonyl)­amino)­butanoate (**si53a**)

A mixture of compound **si52a** (45 mg, 0.062 mmol) and ethyl (*S*)-2-((*tert*-butoxycarbonyl)­amino)-4-oxobutanoate (23 mg, 0.093 mmol) in EtOH (5 mL) was stirred for 1 h at rt. Then NaBH_3_CN (8 mg, 0.124 mmol) was added. The resulting mixture was stirred for additional 12 h at 30 °C. Solvent was removed and the residue was purified by flash chromatography (5% EtOH in DCM) to give compound **si53a** (30 mg, 50.8% yield) as a white gum. MS Calcd: 953; MS Found: 953 [M + H]^+^.

#### *tert*-Butyl (S)-2-((*tert*-butoxycarbonyl)­amino)-4-((3-(4-(4-chlorophenoxy)-3-fluorophenethoxy)­propyl)­(((3aR,4R,6R,6aS)-2,2-dimethyl-6-(4-(methylamino)-7*H*-pyrrolo­[2,3-*d*]­pyrimidin-7-yl)­tetrahydro-4*H*-cyclopenta­[d]­[1,3]­dioxol-4-yl)­methyl)­amino)­butanoate (**si53b**)

A solution of **si52b** (20 mg, 0.03 mmol), **si6** (9 mg, 0.3 mmol) in EtOH (3 mL) was stirred at room temperature for 30 min. Then NaBH_3_CN (4 mg, 0.06 mmol) was added to the solution. The solution was stirred at room temperature for 16 h. The reaction solution was quenched with water (2 mL) and concentrated. The crude was purified by reverse phase flash (MeCN/H_2_O) to afford **si53b** (15 mg, yield: 53%) as a colorless oil. MS Calcd: 881.4; MS Found: 881.3 [M + H]^+^.

#### Ethyl (S)-2-amino-4-((3-((4-(4-chlorophenoxy)-3-fluorophenethyl)­amino)­propyl) (((1R,2R,3S,4R)-2,3-dihydroxy-4-(4-(methylamino)-7*H*-pyrrolo­[2,3-*d*]­pyrimidin-7-yl) cyclopentyl)­methyl)­amino)­butanoate (**8**)

To a mixture of compound **si53a** (30 mg, 0.032 mmol) in DCM (5 mL) was added TFA (2 mL). The resulting mixture was stirred for 2 h at rt. The mixture was concentrated and the residue was purified by *prep*-HPLC (acidic conditions) to give compound **si54a** (17 mg, 83% yield) as a white solid. ^1^H NMR (400 MHz, D_2_O) δ: 8.16 (s, 1H), 7.37 (d, *J* = 3.2 Hz, 1H), 7.27 (d, *J* = 8.8 Hz, 2H), 7.20 (d, *J* = 11.2 Hz, 1H), 7.07 (s, 2H), 6.89 (d, *J* = 8.4 Hz, 2H), 6.77 (s, 1H), 4.96–4.93 (m, 1H), 4.32–4.14 (m, 4H), 4.09–4.06 (t, *J* = 6.0 Hz, 1H), 3.51–3.43 (m, 4H), 3.35–3.32 (m, 4H), 3.23–3.11 (m, 4H), 3.02–2.96 (m, 2H), 2.45–2.31 (m, 4H), 2.15–2.10 (m, 2H), 1.81–1.74 (m, 1H), 1.26–1.21 (m, 3H). MS: calcd. for C_36_H_48_ClFN_7_O_5_ [M + H]^+^: 712.3; found: 712.3/713.3/715.4. HPLC: *t*_R_ = 9.233 min (Method D, without TFA in eluent B), UV-purity at 254 nm: 100%.

#### (S)-2-Amino-4-((3-(4-(4-chlorophenoxy)-3-fluorophenethoxy)­propyl)­(((1R,2R,3S,4R)-2,3-dihydroxy-4-(4-(methylamino)-7*H*-pyrrolo­[2,3-*d*]­pyrimidin-7-yl)­cyclopentyl)­methyl) amino)­butanoic Acid (**7b-N**)

To a solution of **si53b** (15 mg, 0.02 mmol) in DCM (3 mL) was added TFA (3 mL). The solution was stirred at room temperature for 3 h. The mixture was concentrated to dryness and the residue was purified by *prep*-HPLC (acidic conditions) to give **si54b** (10.0 mg, yield: 86%) as a white solid. ^1^H NMR (400 MHz, CD_3_OD): δ = 8.22 (s, 1H), 7.51 (d, *J* = 3.6 Hz, 1H), 7.31–7.28 (m, 2H), 7.17 (d, *J* = 12.4 Hz, 1H), 7.09–7.08 (m, 2H), 6.91–6.88 (m, 3H), 5.09–5.03 (m, 1H), 4.34 (t, *J* = 6.4 Hz, 1H), 4.10 (t, *J* = 6.0 Hz, 1H), 3.93–3.90 (m, 1H), 3.74–3.71 (m, 2H), 3.63–3.60 (m, 2H), 3.55–3.20 (m, 9H), 2.90 (t, *J* = 6.4 Hz, 2H), 2.57–2.45 (m, 2H), 2.41–2.32 (m, 1H), 2.16–2.05 (m, 3H), 1.88–1.80 (m, 1H). MS calcd. for C_34_H_43_ClFN_6_O_6_ [M+H^+^]: 685.3; found: 685.5/688.5. HPLC: *t*_R_ = 3.873 min (Method S1), UV-purity at 254 nm: 97.5%.

#### Ethyl (S)-2-amino-4-((3-(4-(4-chlorophenoxy)-3-fluorophenethoxy)­propyl) (((1R,2R,3S,4R)-2,3-dihydroxy-4-(4-(methylamino)-7*H*-pyrrolo­[2,3-*d*]­pyrimidin-7-yl)­cyclopentyl)­methyl)­amino)­butanoate (**8-N**)

To a solution of **si54b** (15 mg, 0.02 mmol) in EtOH (3 mL) was added TMSCl (9 mg, 0.08 mmol) and the mixture was stirred at 68 °C for 5 h. The mixture was concentrated, and the residue was purified by *prep*-HPLC (acidic conditions) to give **8-N** (6.0 mg, yield: 38%) as a white solid. ^1^H NMR (400 MHz, CD_3_OD): δ = 8.22 (s, 1H), 7.48 (d, *J* = 3.6 Hz, 1H), 7.31–7.28 (m, 2H, 7.17 (d, *J* = 12.8 Hz, 1H), 7.09–7.07 (m, 2H), 6.90–6.87 (m, 3H), 5.05–5.01 (m, 1H), 4.39–4.31 (m, 3H), 4.22 (t, *J* = 6.0 Hz, 1H), 4.11 (t, *J* = 6.8 Hz, 1H), 3.73 (t, *J* = 8.0 Hz, 2H), 3.63 (t, *J* = 5.6 Hz, 2H), 3.26–3.15 (m, 9H), 3.91 (t, *J* = 6.8 Hz, 2H), 2.50–2.38 (m, 3H), 2.31–2.25 (m, 1H), 2.03–2.01 (m, 2H), 1.86–1.80 (m, 1H), 1.36 (t, *J* = 8.0 Hz, 3H). MS: calcd. for C_36_H_47_ClFN_6_O_6_ [M+H^+^]: 713.3; found: 713.4. HPLC: *t*_R_ = 2.998 min (Method S2) UV-purity at 254 nm: 99.8%

### Protein Expression and Purification

For the functional assays, plasmid construction, protein expression and purification of heterodimer KMT9, ETF1 were performed as described previously.^8^ For crystallization, plasmid of pET-Duet1–6*His-TEV-KMT9α (13–214)-KMT9β was constructed. Protein expression and purification of truncated KMT9 were performed in the same way as described before.^8^

### Microscale Thermophoresis (MST) Assays

To determine the binding affinity of the compound and KMT9, microscale thermophoresis (MST) analysis was performed with a NanoTemper Monolith NT.115 instrument (NanoTemper Technologies GmbH). KMT9 was labeled with a RED-Tris-NTA labeling kit (NanoTemper Technologies GmbH) based on the manufacturer’s instructions. Buffer including 25 mM HEPES (pH 7.5), 100 mM NaCl, 1 mM DTT and 0.05% Tween was used for the reaction buffer. Varying concentrations of compounds were titrated against His-tag labeled KMT9 proteins (20 nM). Samples were loaded into standard Capillaries (NanoTemper Technologies GmbH) and MST measurements were performed using 40% MST power and 100% LED power. For each set of binding experiments, MST measurement was carried out with Binding Affinity module in MO. Control program under Nano-RED excitation. Data sets were processed with the MO.Affinity Analysis software (NanoTemper Technologies GmbH).

### KMT9 Methyltransferase Inhibition Assays

The assay was carried out in 0.5 mL Eppendorf tubes in duplicates. Assay buffer consisted of 50 mM BTP, 1 mM MgCl_2_, 1 mM DTT and 0,01% Triton-X100 at a pH of 8.5 (adjusted using 1 M HCl and 1 M NaOH). 20X inhibitor stock (in DMSO), 2× enzyme stock and 4× substrate mix (both in assay buffer) were prepared. The substrate mix consisted of Tritium-labeled S-adenosylmethionine (3H-SAM), S-adenosylmethionine (SAM) and eukaryotic peptide chain release factor subunit1 (, residues 140–275) (ETF1). Final assay concentrations were set to 25 nM KMT9, 0.3 μM 3H-SAM, 0.7 μM SAM and 5 μM ETF1. A 1:1 dilution series of 20× stocks of compound **8** (10 concentrations) was prepared in an optimized range of 250–0.488 μM. 1 μL of the appropriate 20× stock of compound **8**, 4 μL of assay buffer and 10 μL of 2× KMT9 stock were pipetted and incubated at 25 °C for 15 min. Five μL of 4× substrate mix was added to initiate the reaction. The final reaction volume was 20 μL. For the positive control, the compound was replaced by DMSO; for the negative control, the compound was replaced by DMSO and KMT9 was replaced by assay buffer. The reaction mix was incubated in an Eppendorf thermomixer comfort at 30 °C for 2 h, shaking at 300 rpm. The reaction was quenched using 5 μL 50% trichloroacetic acid (TCA) and incubated at 25 °C for 5 min. Twenty-two μL of the mixture was transferred into 96-well MultiScreenHTS FB filter plates (Merck KGaA, Darmstadt, Germany) and washed four times with 10% TCA and two times with 100% ethanol. The plate was dried overnight, the filters were transferred into individual 6 mL Pony Vials (PerkinElmer Inc., Waltham, MA, USA) and incubated in 3 mL of Ultima Gold scintillation cocktail (PerkinElmer Inc., Waltham, MA, USA) for 30 min. The scintillation signal was measured for 3× 1 min using TriCarb 2910 TR (PerkinElmer) scintillation counter in 3H CPM mode (LL: 0, UL: 18.6).

Inhibition was calculated using the following formula:

$$Inhibition\;\left[\%\right]=\left(1-\frac{x_{c}-x_{pos}}{x_{pos}-x_{neg}}\right)\times 100$$

With x_c_: signal of compound, x_pos_: mean signal of positive control, x_neg_: mean signal of negative control. Data fitting was carried out by GraphPad 7.0 using nonlinear fit ([Inhibitor] vs response – variable slope (four parameters)).

### Thermal Shift Assays

The assay was carried out in 96-well Hard-Shell PCR plates (Bio-Rad Laboratories Inc.). Assay buffer consisted of 50 mM BTP, 1 mM MgCl_2_ and1 mM DTT at a pH of 8.5 (adjusted using 1 M HCl and 1 M NaOH). 20× inhibitor stock (in DMSO), 2× enzyme stock and 4× SyproOrange (SO) stock (both in assay buffer) were prepared. Final assay concentrations were set to 500 μM of compound **8**, 2 μM KMT9 and 5× SO. One μL of 20× compound, 4 μL of assay buffer, 10 μL of 2× KMT9 and 5 μL of 4× SO were pipetted. For the no-compound control, 20× inhibitor stock was replaced by DMSO. The final volume was 20 μL. The plate was spun down at 700 rpm for 1 min using a Hettich UNIVERSAL 320 centrifuge and incubated at 25 °C, shaking at 600 rpm. After incubation, the plate was spun down for 1 min at 700 rpm and measurement was conducted using a CFX96 Touch Real-Time PCR Detection System (Bio-Rad Laboratories Inc.). The plate was equilibrated at 20 °C for 4 min and then heated stepwise at a rate of 1 °C per 15 s until 95 °C. After every step, fluorescence was measured in FRET mode. Calculation of melting points was conducted by a Boltzman sigmoidal model using GraphPad Prism 7.0.

### Crystallization, Data Collection and Structure Determination

To obtain the cocrystals of inhibitor bound KMT9 and SAH bound KMT9, purified KMT9 protein was mixed with excess of compound (1:10) and grown in different reservoir conditions at 20 °C: KMT9/compound **1** mixture was grown in 1.1 M Na3 Citrate, 0.1 M Tris (pH 8.5); KMT9/SAH mixture was grown in 2 M (NH4)­2SO4, 0.1 M Bis-Tris (pH 6.0); KMT9/compound **2a**, KMT9/compound **3a**, and KMT9/compound **3b** mixture were grown in 1.2 M Na3 Citrate, 0.1 M Tris (pH 7.5); KMT9/compound **2b** mixture was grown in 1.8 M (NH4)­2SO4, 0.1 M Bis-Tris (pH 6.75); KMT9/compound **2c** mixture was grown in 52% Tacsimate (pH 7.7); KMT9/compound **5b** mixture was grown in 1.3 M Na3 Citrate, 0.1 M HEPES (pH 7.75). Crystals appeared after 1 day and reached full size within 1 week. The cocrystals with compound **5b** were obtained by soaking the KMT9-SAH crystal in the reservoir condition with 10 mM compound for 1 day before the harvesting. Crystals were cryoprotected before being flash frozen in liquid nitrogen. The data were collected at the Swiss Light Source beamline PX3 using a wavelength of 1.0000 Å at 100 K. Data were processed and analyzed with XDS^40^ and Aimless.^41^ The crystal belongs to the space group P61 and contains one copy of KMT9 in one asymmetric unit. The structure was solved via molecular replacement with Phaser^42^ using the published KMT9 structure (PDB: 6H1D) as a search model. Manual building and refinement were performed using Coot^43^ and Refmac^44^ in the CCP4 package.^45^ The final models were validated using MolProbity^46^ in the Phenix package^47^ and RCSB Validation server. Tables S1–S3 summarize the statistics of data collection and refinement. Crystallographic data have been deposited in the Protein Data Bank under the accession codes (9FIM, 9FKE, 9FKG, 9FKM, 9FKV, 9FKW, 9FL5, and 9FL4).

### Structure Based Docking and Modeling

Schrodinger suite 2019-1 (Schrodinger, NY) was used for modeling. The cocrystal structure of KMT9-compound **5b** (PDB code:9FL4) was used as the receptor and prepared by Protein Preparation Wizard. The ligands (compound **7a** and **7b**) were prepared and minimized by Ligprep Module. Glide XP^48^ was used for the docking with default settings. Core constraints were implemented with the SMART pattern of compound **5b**. Prime^49^ MM-GBSA was performed by using the docking poses from Glide XP.

### Multiple Structural Alignment

PyMod 3 ^50^ package is used for the alignment. Structures Corresponding of PMTs were downloaded from PDB database and prepared in Pymol. SALIGN module of Modeler^51^ and SCR-FIND^52^ algorithm in the package is used for structural alignment and structural conservation analysis with default setting.

### Selectivity Assays

Selectivity profile of compound **6** was evaluated by testing the IC_50_ in a panel of methyltransferases. The IC_50_ of a panel of SET-domain containing and Rossmann-fold methyltransferases was assessed in a radiometric assay measuring substrate methylation using ^3^H-labeled SAM by RBC Corp by compound **6**. Data was process and plotted using KNIME Analytic Platform from KNIME AG.

### Cell Lines

SW480, Caco-2 and HepG2 cells were obtained from ATCC. PC-3 M cells were obtained from Caliper Life Sciences. HEK 293 KMT9α^–/–^ have been previously described.^8^ Cell lines were not further authentified. They were tested for mycoplasma and found to be uncontaminated.

### Cell Culture

PC-3 M were cultured in RPMI 1640. HEK 293 KMT9α^–/–^, Caco-2, and HepG2 cells were cultured in DMEM. All media were supplemented with 10% fetal calf serum, penicillin/streptomycin, and glutamine. The culture medium for HepG2 was supplemented with nonessential amino acids.

### Western Blot Analysis

Experiments were performed as previously described.^53^ Histones used for Western blot analysis were extracted from cells as following: Cells were harvested, washed with PBS, resuspended in Triton Extraction Buffer (TEB; PBS, Triton ×100, 2 mM PMSF, 0.02% NaN3), stored 10 min on ice, centrifuged, and supernatant was discarded. After washing cells, a second time with TEB, pellets were resuspended in 0.2 N HCl and histones were acid-extracted overnight at 4 °C. Upon centrifugation for 10 min at 2000 rpm, supernatant was collected and protein concentration was determined. Three days before harvesting, cells were transfected with siRNA as indicated. The following antibodies were used: anti-KMT9α (#27630, lot 20062017, Schüle Lab), antihistone H4 (#ab10158, lot GR322677–1, Abcam), anti-H4K12me1 (#27429, lot 27062017, Schüle Lab).

### Cell Proliferation Assay

Cell proliferation was determined using the X-Celligence RTCA system (Roche). For real-time recording of SW480, Caco-2 cell proliferation, 5000 cells/well were seeded in 16 well E-plates (Roche). Cells were seeded in the presence of DMSO or compound **8**/**8-N** or transfected with the indicated siRNAs in the presence of Dharmafect (Life Technologies) 24 h before seeding in E-plates. Cell indices were automatically recorded every 15 min. Relative velocities represent the change of the cell index over time. The sequences of the siRNAs (Stealth RNAi siRNAs; Life Technologies) used in the experiments have been previously described.^8^

### Cellular Thermal Shift Assay (CETSA)

For whole cell CETSA, the cells were incubated with 10/15 μM of compound **7b**/**8/8-N** for 18 h. Then, cells were washed with cell culture medium, detached from the surface using trypsin, washed with PBS and the number of cells was determined. Cell concentration was adjusted to 3.6 × 10^7^ cells/mL using in PBS with Complete (w/o EDTA, Roche) protease inhibitor. Then, cells were frozen in liquid nitrogen and thawed at 25 °C three times, before being divided in 16 aliquots. Each aliquot was heated to a defined temperature between 40 and 70 °C in a PCR cycler (16 well gradient, 55 °C ± 15). After 3 min incubation, aliquots were snap frozen in liquid nitrogen and thawed at 25 °C. Cell lysates were then centrifuged at 20,000 × g , 4 °C for 20 min. Supernatant were mixed with 5× loading buffer, heated to 70 °C for 5 min and analyzed by Western blotting essentially as previously described.^53^ For the CETSA performed with cell lysates, cells were directly harvested and lysed as described above. The lysates were then split and incubated either with DMSO or 10 μM compound **7b** for 2 h. Then, lysates were aliquoted again into 16 samples each and treated as described for the whole cell CETSA.

### Statistics

Data are represented as mean + standard deviation (s.d.). Significance was calculated by two-tailed Student’s *t* test as indicated in the figure legends. Statistical significance was set to *p* < 0.05 and is represented as following: ***p < 0.001, ** *p* < 0.01, * *p* < 0.05. Sample sizes are indicated where appropriate.

## Supplementary Material









## Notes

Molecular formula strings (CSV)
Validation Report of PDB structures (PDB)
Coordinates for the modeling structures of KMT9 in complex with compound **7a**/**7b** (PDB)
Supplementary Figures: Figure S1. Identification of a hit compound of KMT9. Figure S2. Structure activity relationship of compound **1** derivatives. Figure S3. Lead optimization of compound **2d**. Figure S4. Cellular target engagement of the lead compounds. Figure S5. Cellular activity of prodrug compound **8**. Supplementary Synthetic Schemes: Scheme 1–9. Synthetic schemes of the final or intermediate compounds. Characterization of synthesized compounds: HPLC purity, Mass spectrum and ^1^H NMR spectra of all compounds. Supplementary Tables : Table S1–S3. Crystallography data and refinement statistics (PDF)

## References

[1] Kaniskan H. U., Martini M. L., Jin J. (2018). Inhibitors of Protein Methyltransferases
and Demethylases. Chem. Rev..

[2] Luo M. (2018). Chemical and
Biochemical Perspectives of Protein Lysine Methylation. Chem. Rev..

[3] Shen H., Laird P. W. (2013). Interplay between
the cancer genome and epigenome. Cell.

[4] Polak P., Karlic R., Koren A., Thurman R., Sandstrom R., Lawrence M., Reynolds A., Rynes E., Vlahovicek K., Stamatoyannopoulos J. A., Sunyaev S. R. (2015). Cell-of-origin chromatin
organization shapes the mutational landscape of cancer. Nature.

[5] Chi P., Allis C. D., Wang G. G. (2010). Covalent
histone modifications--miswritten,
misinterpreted and mis-erased in human cancers. Nat. Rev. Cancer.

[6] Schneider R., Bannister A. J., Kouzarides T. (2002). Unsafe SETs: histone lysine methyltransferases
and cancer. Trends Biochem. Sci..

[7] Yang Y., Bedford M. T. (2013). Protein arginine
methyltransferases and cancer. Nat. Rev. Cancer.

[8] Metzger E., Wang S., Urban S., Willmann D., Schmidt A., Offermann A., Allen A., Sum M., Obier N., Cottard F., Ulferts S., Preca B. T., Hermann B., Maurer J., Greschik H., Hornung V., Einsle O., Perner S., Imhof A., Jung M., Schule R. (2019). KMT9 monomethylates
histone H4 lysine 12 and controls proliferation of prostate cancer
cells. Nat. Struct. Mol. Biol..

[9] Heurgue-Hamard V., Champ S., Engstrom A., Ehrenberg M., Buckingham R. H. (2002). The hemK gene in Escherichia coli
encodes the N(5)-glutamine
methyltransferase that modifies peptide release factors. EMBO J.

[10] Nakahigashi K., Kubo N., Narita S., Shimaoka T., Goto S., Oshima T., Mori H., Maeda M., Wada C., Inokuchi H. (2002). HemK, a class of protein methyl transferase with similarity
to DNA methyl transferases, methylates polypeptide chain release factors,
and hemK knockout induces defects in translational termination. Proc. Natl. Acad. Sci. U. S. A..

[11] Kusevic D., Kudithipudi S., Jeltsch A. (2016). Substrate Specificity of the HEMK2
Protein Glutamine Methyltransferase and Identification of Novel Substrates. J. Biol. Chem..

[12] Cheng X. (1995). Structure
and function of DNA methyltransferases. Annu.
Rev. Biophys. Biomol. Struct..

[13] Yang Z., Shipman L., Zhang M., Anton B. P., Roberts R. J., Cheng X. (2004). Structural characterization
and comparative phylogenetic analysis
of Escherichia coli HemK, a protein (N5)-glutamine methyltransferase. J. Mol. Biol..

[14] Xiao C.L., Zhu S., He M., Chen D., Zhang Q., Chen Y., Yu G., Liu J., Xie S.Q., Luo F., Liang Z. (2018). N6-Methyladenine
DNA Modification in the Human Genome. Mol. Cell..

[15] Li X., Zhao Q., Wei W., Lin Q., Magnan C., Emami M. R., Wearick-Silva L. E., Viola T. W., Marshall P. R., Yin J., Madugalle S. U., Wang Z., Nainar S., Vagbo C. B., Leighton L. J., Zajaczkowski E. L., Ke K., Grassi-Oliveira R., Bjoras M., Baldi P. F., Spitale R. C., Bredy T. W. (2019). The DNA
modification N6-methyl-2’-deoxyadenosine (m6dA) drives activity-induced
gene expression and is required for fear extinction. Nat. Neurosci..

[16] Baumert H. M., Metzger E., Fahrner M., George J., Thomas R. K., Schilling O., Schule R. (2020). Depletion of histone methyltransferase
KMT9 inhibits lung cancer cell proliferation by inducing non-apoptotic
cell death. Cancer Cell. Int..

[17] Berlin C. C. F., Cottard F., Willmann D., Urban S., Tirier S. M., Marx L., Rippe K., Schmitt M., Petrocelli V., Greten F. R. (2022). KMT9 controls stemness
and growth of colorectal
cancer. Cancer Res..

[18] Koll F. J., Metzger E., Hamann J., Ramos-Triguero A., Bankov K., Köllermann J., Döring C., Chun F. K. H., Schüle R., Wild P. J., Reis H. (2023). Overexpression
of KMT9α Is Associated with Aggressive Basal-like Muscle-Invasive
Bladder Cancer. Cells.

[19] Chen D., Meng Y., Yu D., Noinaj N., Cheng X., Huang R. (2021). Chemoproteomic Study
Uncovers HemK2/KMT9 As a New Target for NTMT1
Bisubstrate Inhibitors. ACS Chem. Biol..

[20] Wang S., Klein S. O., Urban S., Staudt M., Barthes N. P. F., Willmann D., Bacher J., Sum M., Bauer H., Peng L. (2024). Structure-guided design of a selective inhibitor of
the methyltransferase KMT9 with cellular activity. Nat. Commun..

[21] Campagna-Slater V., Mok M. W., Nguyen K. T., Feher M., Najmanovich R., Schapira M. (2011). Structural chemistry
of the histone methyltransferases
cofactor binding site. J. Chem. Inf. Model..

[22] Jafari R., Almqvist H., Axelsson H., Ignatushchenko M., Lundbäck T., Nordlund P., Molina D. M. (2014). The cellular
thermal
shift assay for evaluating drug target interactions in cells. Nat. Protoc..

[23] Beaumont K., Webster R., Gardner I., Dack K. (2005). Design of Ester Prodrugs
to Enhance Oral Absorption of Poorly Permeable Compounds: Challenges
to the Discovery Scientist. Curr. Drug Metab..

[24] Berlin C., Cottard F., Willmann D., Urban S., Tirier S. M., Marx L., Rippe K., Schmitt M., Petrocelli V., Greten F. R., Fichtner-Feigl S., Kesselring R., Metzger E., Schule R. (2022). KMT9 Controls Stemness
and Growth
of Colorectal Cancer. Cancer Res..

[25] Mori S., Iwase K., Iwanami N., Tanaka Y., Kagechika H., Hirano T. (2010). Development of novel
bisubstrate-type inhibitors of
histone methyltransferase SET7/9. Bioorg. Med.
Chem..

[26] Dowden J., Hong W., Parry R. V., Pike R. A., Ward S. G. (2010). Toward
the development of potent and selective bisubstrate inhibitors of
protein arginine methyltransferases. Bioorg.
Med. Chem. Lett..

[27] van
Haren M., van Ufford L. Q., Moret E. E., Martin N. I. (2015). Synthesis
and evaluation of protein arginine N-methyltransferase inhibitors
designed to simultaneously occupy both substrate binding sites. Org. Biomol. Chem..

[28] van
Haren M. J., Marechal N., Troffer-Charlier N., Cianciulli A., Sbardella G., Cavarelli J., Martin N. I. (2017). Transition state mimics are valuable mechanistic probes
for structural studies with the arginine methyltransferase CARM1. Proc. Natl. Acad. Sci. U. S. A..

[29] Chen D., Dong C., Dong G., Srinivasan K., Min J., Noinaj N., Huang R. (2020). Probing the
Plasticity in the Active
Site of Protein N-terminal Methyltransferase 1 Using Bisubstrate Analogues. J. Med. Chem..

[30] Gunnell E. A., Al-Noori A., Muhsen U., Davies C. C., Dowden J., Dreveny I. (2020). Structural and biochemical
evaluation of bisubstrate
inhibitors of protein arginine N-methyltransferases PRMT1 and CARM1
(PRMT4). Biochem. J..

[31] Chen D., Dong G., Noinaj N., Huang R. (2019). Discovery of Bisubstrate
Inhibitors for Protein N-Terminal Methyltransferase 1. J. Med. Chem..

[32] Cai X. C., Zhang T., Kim E. J., Jiang M., Wang K., Wang J., Chen S., Zhang N., Wu H., Li F. (2019). A chemical
probe of CARM1 alters epigenetic plasticity
against breast cancer cell invasion. Elife.

[33] Pande V., Sun W., Beke L., Berthelot D., Brehmer D., Brown D., Corbera J., Irving S., Meerpoel L., Nys T., Parade M., Robinson C., Sommen C., Viellevoye M., Wu T., Thuring J. W. (2020). A Chemical
Probe for the Methyl Transferase PRMT5 with
a Novel Binding Mode. ACS Med. Chem. Lett..

[34] Chern T. R., Liu L., Petrunak E., Stuckey J. A., Wang M., Bernard D., Zhou H., Lee S., Dou Y., Wang S. (2020). Discovery
of Potent Small-Molecule Inhibitors of MLL Methyltransferase. ACS Med. Chem. Lett..

[35] Van
Aller G. S., Graves A. P., Elkins P. A., Bonnette W. G., McDevitt P. J., Zappacosta F., Annan R. S., Dean T. W., Su D. S., Carpenter C. L., Mohammad H. P., Kruger R. G. (2016). Structure-Based
Design of a Novel SMYD3 Inhibitor that Bridges the SAM-and MEKK2-Binding
Pockets. Structure.

[36] Quiroz R. V., Reutershan M. H., Schneider S. E., Sloman D., Lacey B. M., Swalm B. M., Yeung C. S., Gibeau C., Spellman D. S., Rankic D. A., Chen D., Witter D., Linn D., Munsell E., Feng G., Xu H., Hughes J. M. E., Lim J., Sauri J., Geddes K., Wan M., Mansueto M. S., Follmer N. E., Fier P. S., Siliphaivanh P., Daublain P., Palte R. L., Hayes R. P., Lee S., Kawamura S., Silverman S., Sanyal S., Henderson T. J., Ye Y., Gao Y., Nicholson B., Machacek M. R. (2021). The Discovery of
Two Novel Classes of 5,5-Bicyclic Nucleoside-Derived PRMT5 Inhibitors
for the Treatment of Cancer. J. Med. Chem..

[37] Deng Y., Kim E. J., Song X., Kulkarni A. S., Zhu R. X., Wang Y., Bush M., Dong A., Noinaj N., Min J., Xu W., Huang R. (2024). An Adenosine Analogue Library Reveals
Insights into Active Sites of Protein Arginine Methyltransferases
and Enables the Discovery of a Selective PRMT4 Inhibitor. J. Med. Chem..

[38] Schapira M. (2016). Chemical Inhibition
of Protein Methyltransferases. Cell Chem. Biol..

[39] Zhang J., Zheng Y. G. (2016). SAM/SAH Analogs as Versatile Tools for SAM-Dependent
Methyltransferases. ACS Chem. Biol..

[40] Kabsch W. (2010). Xds. Acta Crystallogr.,
Sect. D: Biol. Crystallogr..

[41] Evans P. R., Murshudov G. N. (2013). How good are my data and what is
the resolution?. Acta Crystallogr., Sect. D:
Biol. Crystallogr..

[42] McCoy A. J., Grosse-Kunstleve R. W., Adams P. D., Winn M. D., Storoni L. C., Read R. J. (2007). Phaser crystallographic software. J. Appl. Crystallogr..

[43] Emsley P., Lohkamp B., Scott W. G., Cowtan K. (2010). Features and
development
of Coot. Acta Crystallogr., Sect. D: Biol. Crystallogr..

[44] Vagin A. A., Steiner R. A., Lebedev A. A., Potterton L., McNicholas S., Long F., Murshudov G. N. (2004). REFMAC5
dictionary: organization of prior chemical knowledge and guidelines
for its use. Acta Crystallogr., Sect. D: Biol.
Crystallogr..

[45] Winn M. D., Ballard C. C., Cowtan K. D., Dodson E. J., Emsley P., Evans P. R., Keegan R. M., Krissinel E. B., Leslie A. G., McCoy A., McNicholas S. J. (2011). Overview of the CCP4 suite and current developments. Acta Crystallogr., Sect. D: Biol. Crystallogr..

[46] Chen V. B., Arendall W. B., Headd J. J., Keedy D. A., Immormino R. M., Kapral G. J., Murray L. W., Richardson J. S., Richardson D. C. (2010). MolProbity: all-atom structure validation for macromolecular
crystallography. Acta Crystallogr., Sect. D:
Biol. Crystallogr..

[47] Adams P. D., Afonine P. V., Bunkoczi G., Chen V. B., Davis I. W., Echols N., Headd J. J., Hung L. W., Kapral G. J., Grosse-Kunstleve R. W. (2010). PHENIX: a comprehensive Python-based system
for macromolecular structure solution. Acta
Crystallogr., Sect. D: Biol. Crystallogr..

[48] Friesner R. A., Murphy R. B., Repasky M. P., Frye L. L., Greenwood J. R., Halgren T. A., Sanschagrin P. C., Mainz D. T. (2006). Extra precision
glide: docking and scoring incorporating a model of hydrophobic enclosure
for protein-ligand complexes. J. Med. Chem..

[49] Jacobson M.
P., Pincus D. L., Rapp C. S., Day T. J., Honig B., Shaw D. E., Friesner R. A. (2004). A hierarchical approach to all-atom
protein loop prediction. Proteins.

[50] Janson G., Paiardini A., Arne E. (2021). PyMod 3: A complete suite for structural
bioinformatics in PyMOL. Bioinformatics.

[51] Webb B., Sali A. (2016). Comparative Protein
Structure Modeling Using MODELLER. Curr. Protoc.
Protein Sci..

[52] Paiardini A., Bossa F., Pascarella S. (2005). CAMPO, SCR_FIND
and CHC_FIND: a suite
of web tools for computational structural biology. Nucleic Acids Res..

[53] Metzger E., Wissmann M., Yin N., Muller J. M., Schneider R., Peters A. H., Gunther T., Buettner R., Schule R. (2005). LSD1 demethylates
repressive histone marks to promote androgen-receptor-dependent transcription. Nature.

