# Supplementary material for: Structure-Guided Design of a KMT9 Inhibitor Prodrug with Cellular Activity
Source: J Med Chem. 2025 Jun 17;68(13):13295–320. doi: 10.1021/acs.jmedchem.4c02953 (PMC12257510; doi:10.1021/acs.jmedchem.4c02953)
Supplement: Supplementary file 4 [file jm4c02953_si_004.pdf]

Supporting Information

Structure-guided design of a KMT9 inhibitor prodrug with cellular activity

Sheng Wang[1#], Nicolas P. F. Barthes[2#], Sylvia Urban[1], Viktor I. Hazai[2], Sebastian O. Klein[2], Tabea Pappert[2], Paul Kümmel[2], Nicolas Heller[2], Johannes Bacher[2], Maximilian Staudt[2], Jan Ruprecht[2], Ling Peng[1], Manuela Sum[1], Christopher Berlin[1], Daad Sarraf[3], Pierre Regenass[3], Robin Warstat[3], Johannes Walz[3], Pankaj Mishra[4], Lin Zhang[5], Oliver Einsle[5], Stefan Günther[4], Bernhard Breit[3], Eric Metzger[1,6], Manfred Jung[2,6,7*] and Roland Schüle[1,6,7*]

[1] Klinik für Urologie und Zentrale Klinische Forschung, Klinikum der Albert-Ludwigs-Universität Freiburg, Freiburg 79106, Germany.

[2] Institute of Pharmaceutical Sciences, Albert-Ludwigs-Universität Freiburg, Freiburg 79104, Germany.

[3] Institute of Organic Chemistry, Albert-Ludwigs-Universität Freiburg, Freiburg 79104, Germany.

[4] Institute of Pharmaceutical Sciences, Albert-Ludwigs-Universität Freiburg, Freiburg 79104, Germany.

[5] Institut für Biochemie, Albert-Ludwigs-Universität Freiburg, Freiburg 79104, Germany.

[6] German Cancer Consortium (DKTK), Partner site Freiburg, a partnership between DKFZ and Medical Center - University of Freiburg. Freiburg 79106, Germany.

[7] CIBSS Centre of Biological Signalling Studies, University of Freiburg, Freiburg 79106, Germany.

[*] Email: manfred.jung@pharmazie.uni-freiburg.de and roland.schuele@uniklinik-freiburg.de

[#] S.W. and N.P.F.B. contributed equally to this work

Table of Contents

Supplementary Figures

Fig S1. Identification of hit compound of KMT9 .... S3

Fig S2. Structure activity relationship of compound **1** derivatives S4

Fig S3. Lead optimisation of compound **2d** S5

Fig S4. Cellular target engagement of the lead compounds S6

Fig S5. Cellular activity of pro-drug compound **8** S7

Supplementary Synthetic Schemes

Scheme 1. Synthesis of the final compound 1 .... S8

Scheme 2. Synthesis of aldehyde intermediates si13a-d S8

Scheme 3. Synthesis of the fluorinated compounds 4b and 4c S9

Scheme 4. Synthesis of the Adenosine analogues 4e-g S10

Scheme 5. Synthesis of the final compound 4d S10

Scheme 6. Synthesis of the side chain derivatives si31 and si33 .... S10

Scheme 7. Synthesis of the side chain si61 .... S11

Scheme 8. Synthesis of ether linker si64 .... S11

Scheme 9. Synthesis of the final compounds 8, 7b-N and 8-N S12

Characterization of synthesized compounds

HPLC purity, Mass spectrum and ^{1}H-NMR spectra of final compounds S13

Supplementary Tables

Table S1. Crystallographic data for co-crystal structures of KMT9 in complex with SAH, compound **1** and **2b** S48

Table S2. Crystallographic data for co-crystal structures of KMT9 in complex with compound **2c, 3a** and **3b** S49

Table S3. Crystallographic data for co-crystal structures of KMT9 in complex with compound **5b** S50

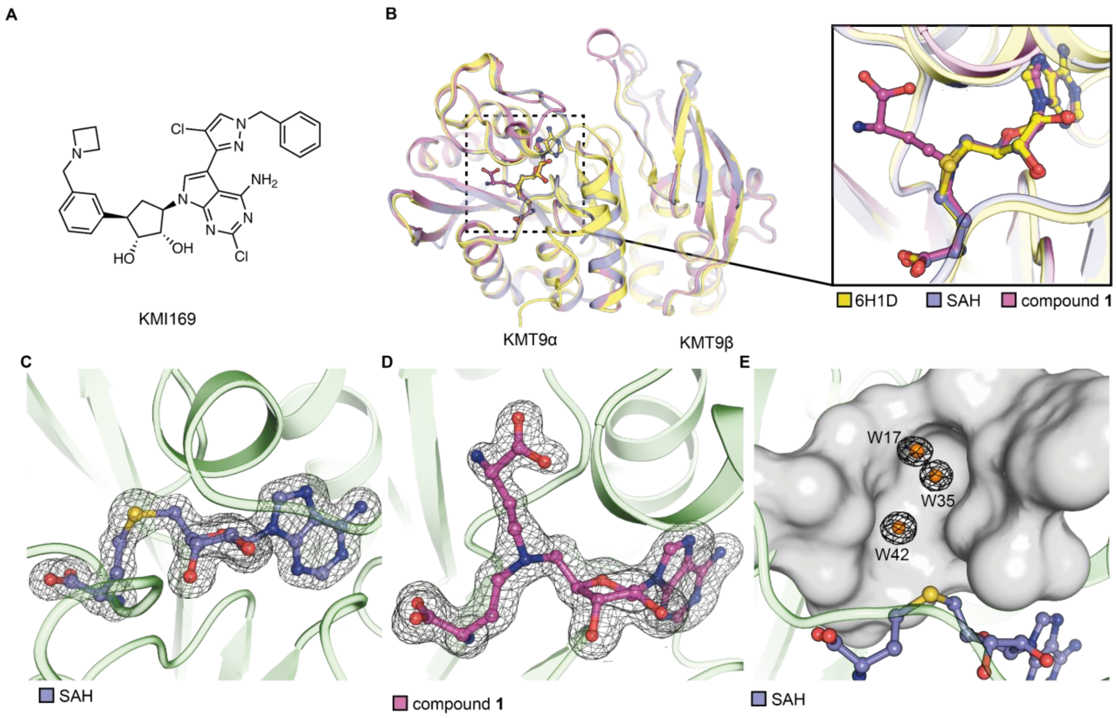

Figure S1. Identification of hit compound of KMT9. (A) Chemical structure of KMI169. (B) Superimposition of SAH-/compound **1**- bound KMT9. (C-E) 2Fo-Fc electron density map contoured at 1σ for SAH (C), compound **1** (D), and water molecules (E). SAH (blue) and compound **1** (magenta) are shown as sticks. Water molecules are shown as orange spheres. Protein is shown as ribbon. Substrate pocket of KMT9α is shown as surface (green).

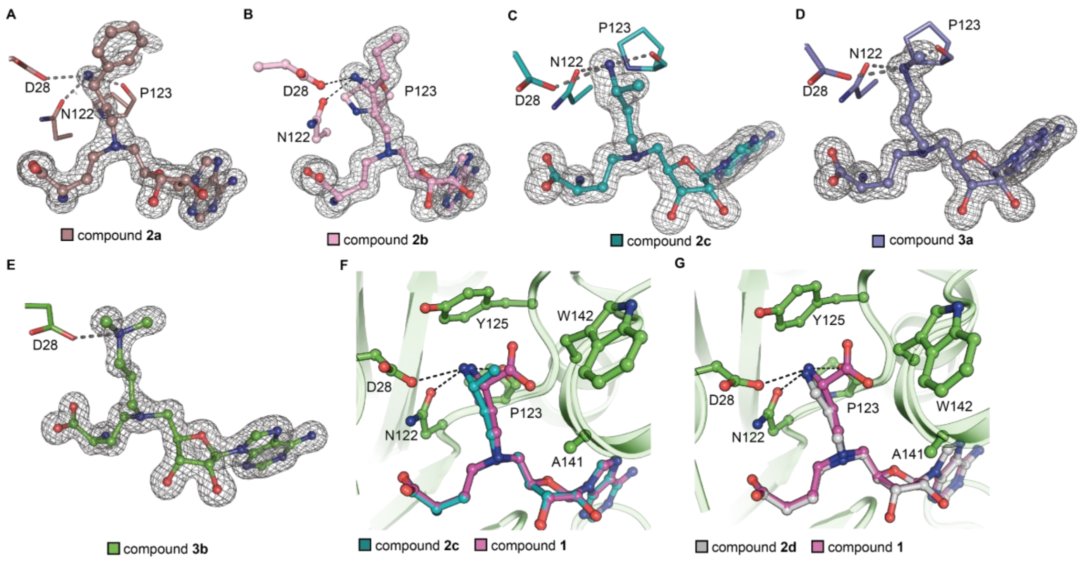

Figure S2. Structure activity relationship of compound 1 derivatives. (A-E) 2Fo-Fc electron density map contoured at 1σ for compound **2a** (brown), **2b** (pink), **2c** (cyan), **3a** (blue), and **3b** (green). (F) Superimposition of compound **2c-** (cyan) and compound **1-** (magenta) bound KMT9α. (G) Superimposition of compound **2d-** (white) and compound **1-** (magenta) bound KMT9α. Ligands and key residues are represented by sticks. KMT9α is shown as ribbon. Hydrogen-bonds are shown as grey dashed lines.

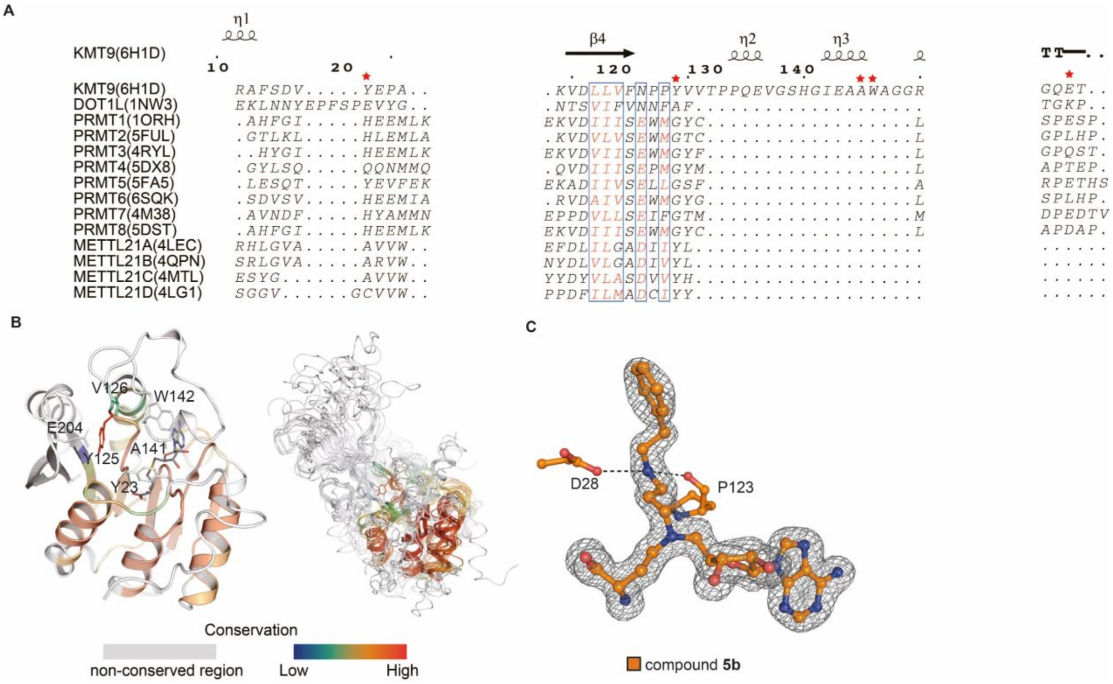

Figure S3. Lead optimisation of compound 2d. (A) Multiple structural alignment of KMT9 across Rossmann fold MTs. Key residues forming the substrate channel of KMT9 are marked by red stars. (B) Structural conservation of KMT9a and Superimposition of structural aligned Rossmann fold MTs including KMT9. Residue conservation is shown with a color scheme ranging from blue (low conservation) to red (high conservation); the residues which are not found in structural conserved region are colored in white; protein is displayed by cartoon; key residues are shown by stick. (C) 2Fo-Fc electron density map contoured at 1σ for compound **5b**.

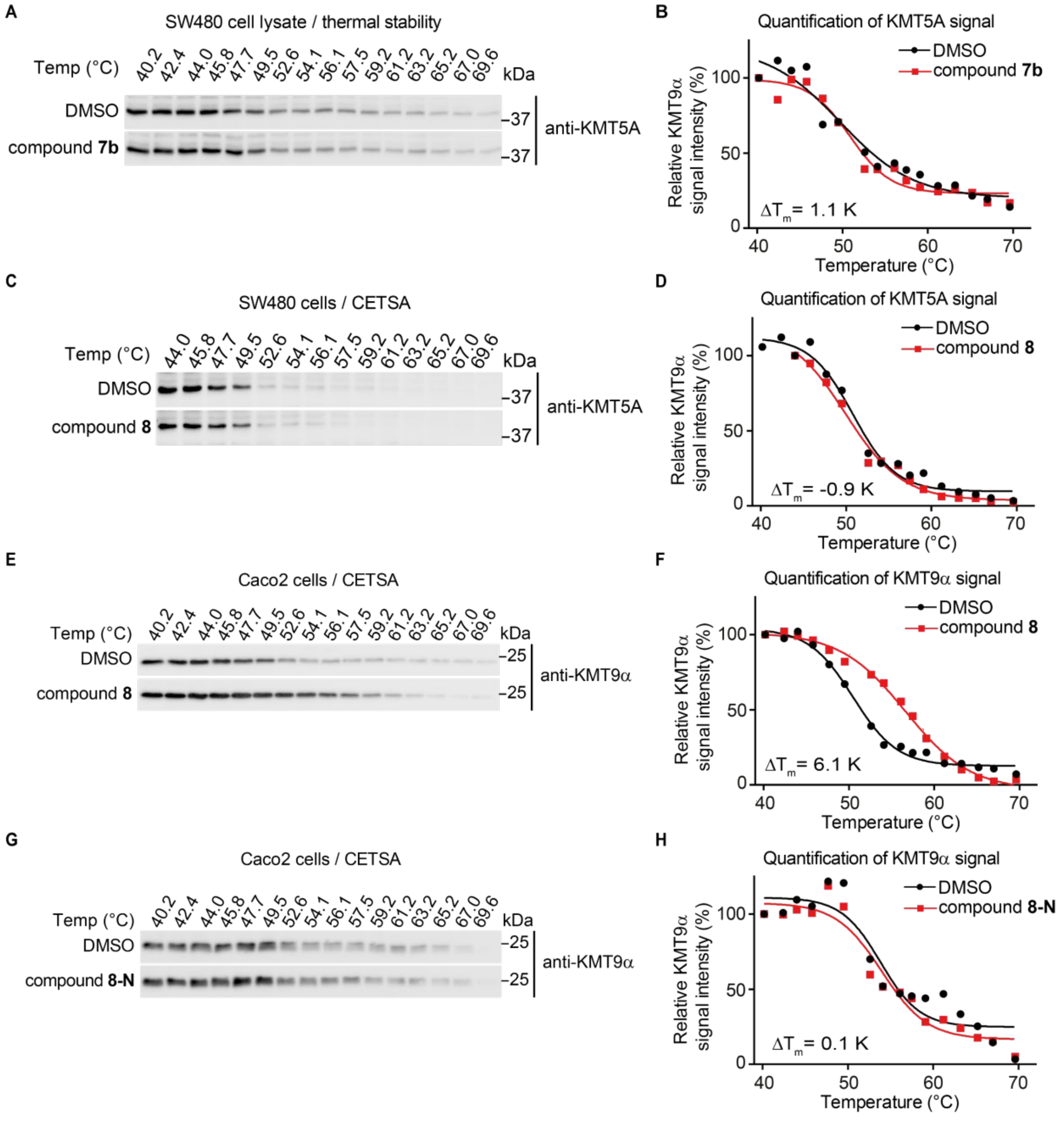

Figure S4. Cellular target engagement of the lead compounds. (A, B) CETSA for KMT5A in SW480 cell lysate treated with vehicle (DMSO), 10µM compound **7b**. Representative Western blots (A) and quantification (B) showing no obvious shifting melting temperatures (ΔT_m) of endogenous KMT5A upon treatment with compound **7b** compared to DMSO. (C, D) CETSA for KMT5A in SW480 cells treated with vehicle (DMSO), 10µM compound **8**. Representative Western blots (C) and quantification (D) showing no shifting melting temperatures (ΔT_m) of endogenous KMT5A upon treatment with compound **8** compared to DMSO. (E-H) CETSA for KMT9a in Caco2 cells treated with vehicle (DMSO), 15µM compound **8** or compound **8-N**. Representative Western blots (E, G) and quantification (F, H) showing increased melting temperatures (ΔT_m) of endogenous KMT9 upon treatment with compound **8** compared to compound **8-N** or DMSO.

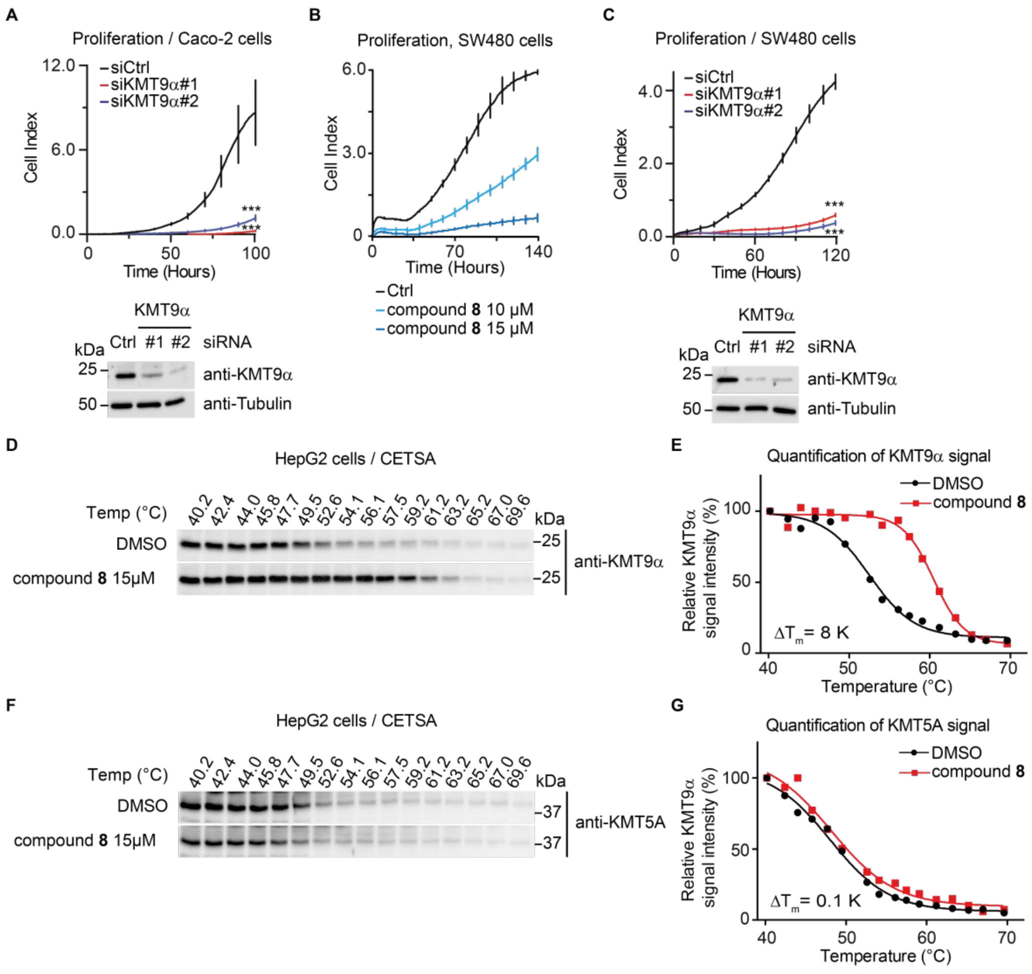

Figure S5. Cellular activity of pro-drug compound 8. (A) Proliferation of Caco-2 cells transfected with siCtrl or siKMT9a as indicated. Western blot analyses were performed with the indicated antibodies to verify knockdown of KMT9a. (B) Proliferation of SW480 cells treated with compound 8 or DMSO (Ctrl). (C) Proliferation of SW480 cells transfected with siCtrl or siKMT9a as indicated. Western blot analyses were performed with the indicated antibodies to verify knockdown of KMT9a. Data represent means +s.d (n=3), * p<0.05, ** p<0.005, *** p<0.001 by two-tailed Student's test. (D, E) CETSA for KMT9a in HepG2 cells treated with vehicle (DMSO), 15µM compound **8**. Representative Western blots (D) and quantification (E) showing increased melting temperatures (ΔT_m) of endogenous KMT9a upon treatment with compound **8** compared to DMSO. (F, G) CETSA for KMT5A in HepG2 cells treated with vehicle (DMSO), 15µM compound **8**. Representative Western blots (F) and quantification (G) showing no obvious shifting melting temperatures (ΔT_m) of endogenous KMT5A upon treatment with compound **8** compared to DMSO.

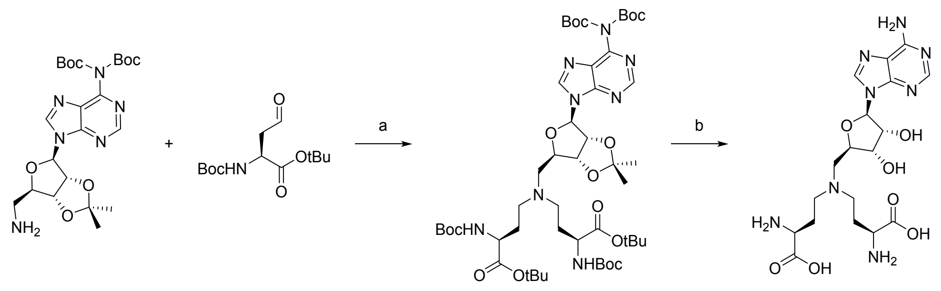

si4-a **si6** **si8k** **1**

Supplementary Scheme 1. Synthesis of the final compound 1. Reagents and conditions: (a) $MgSO_4$, MeOH, 0 °C, 30 min, then $NaBH_3CN$, rt, overnight; (b) TFA/H_2O (4:1), rt, 16 h.

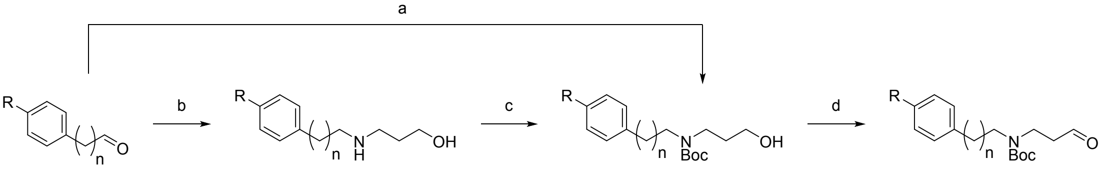

si10a (n = 1, R = H)
si10b (n = 2, R = H)
si10c (n = 1, R = OPh)

si11a (n = 0, R = H)
si11b (n = 1, R = OPh)

si12a (n = 1, R = H)
si12b (n = 2, R = H)
si12c (n = 0, R = H)
si12d (n = 1, R = OPh)

si13a (n = 1, R = H)
si13b n = 2, R = H)
si13c (n = 0, R = H)
si13d (n = 1, R = OPh)

Supplementary Scheme 2. Synthesis of aldehyde intermediates si13a-d. Reagents and conditions: (a) i) 3-amino-1-propanol, Et_3N, DCM, rt, 1 h; ii) Boc_2O, $NaBH(OAc)_3$ rt, 16 h, 8% (**si12a**), 23% (**si12b**); (b) i) 3-aminopropan-1-ol, MeOH, rt, 48 h; ii) $NaBH_4$, 0 °C to rt, overnight, 51% (**si11b**); (c) Et_3N, Boc_2O, DCM, 0 °C to rt, 1-2 h, 100% (**si12d**), 70% (**si12c**); (d) i) $(COCl)_2$, DMSO, DCM, -78 °C, 15 min; ii) compound **si12a-d** in DCM, -78 °C, 30 min, 77% (**si13a**), 80% (**si13b**), 92% (**si13c**) 83% (**si13d**).

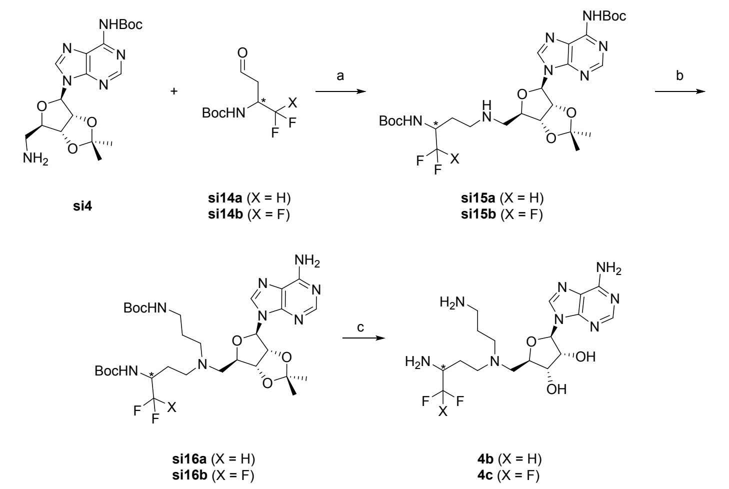

Supplementary Scheme 3. Synthesis of the fluorinated compounds 4b and 4c. Reagents and conditions: (a) AcOH, DCE, rt, 4 h, then $NaBH(OAc)_3$, rt, 4 h, 43% (**si15a**), 45% (**si15b**); (b) aldehyde **si66d**, AcOH, DCE, rt, 4 h, then $NaBH(OAc)_3$, rt, 4 h, then 70 °C, 12 h, 32% (**si16a**), 27% (**si16b**); (c) TFA/H_2O (4:1), rt, 6-16 h, quant. (**si17a**), quant. (**si17b**).

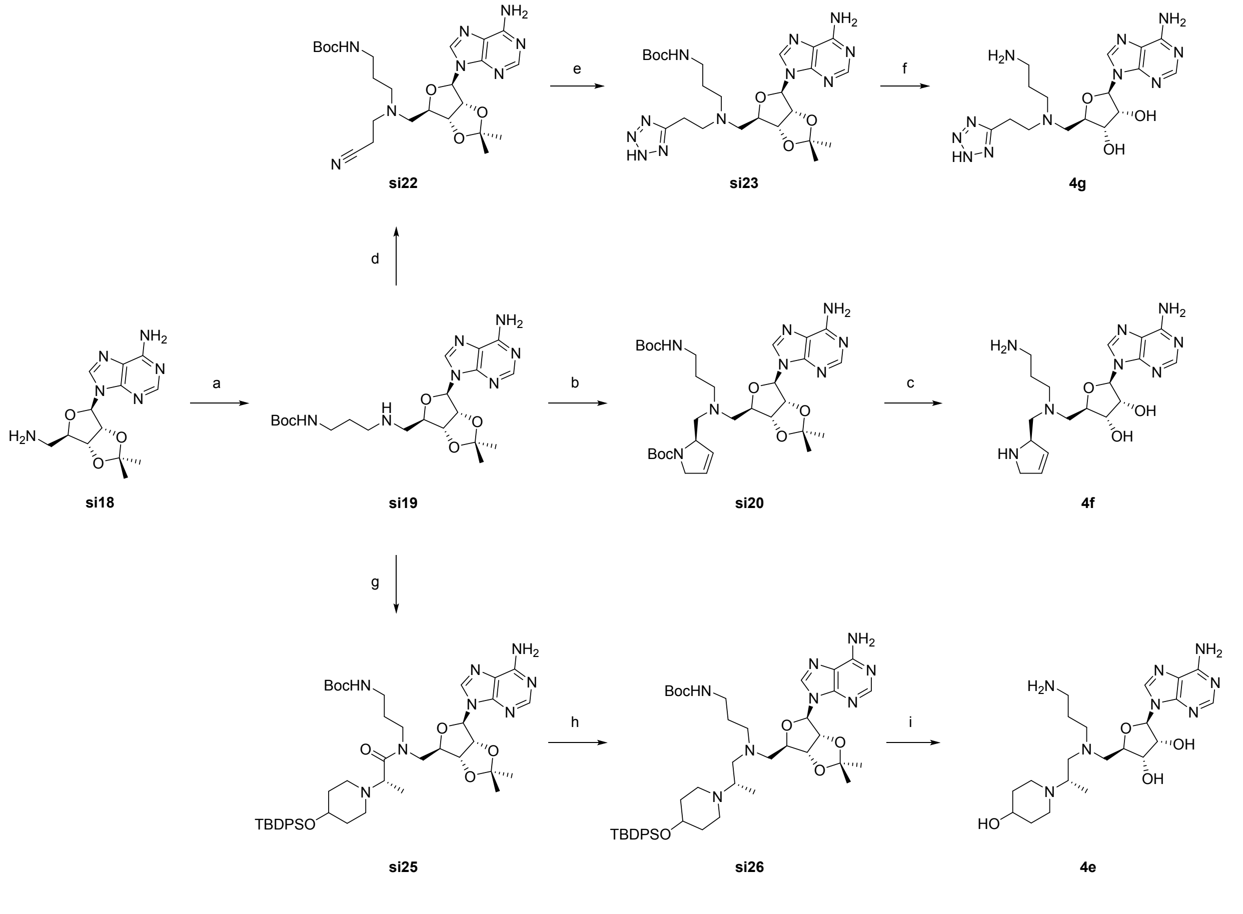

Supplementary Scheme 4. Synthesis of the Adenosine analogues 4e-g. Reagents and conditions: (a) compound **si66d**, $NaBH_3CN$, EtOH, 30 °C, overnight, 76%; (b) compound **si31**, $NaBH_3CN$, AcOH, EtOH, rt, 24 h, 12%; (c) TFA, DCM, 1 h, then H_2O, rt, 3 h, 79%; (d) acrylonitrile, Et_3N, MeOH, 60 °C, overnight, 91%; (e) NaN_3, NH_4Cl, DMF, 130 °C, overnight, 22%; (f) TFA/H_2O (1:1), DCM, rt, overnight, 33%; (g) compound **si33**, PyBop, DIPEA, DMF, rt, overnight, 85%; (h) i) $LiAlH_4$, $AlCl_3$, THF, 40 °C, 30 min, ii) substrate **si26**, THF, 0 °C, 30 min, 22%; (i) TFA, DCM, rt, 4 h, then H_2O, rt, 2 h, 29%.

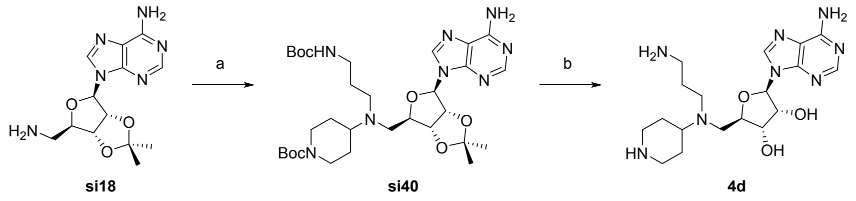

Supplementary Scheme 5. Synthesis of the final compound 4d. Reagents and conditions: (a) 4-oxopiperidine-1-carboxylate, $NaBH_3CN$, MeOH, rt, overnight, then compound **si66d**, rt, overnight, 6%; (b) TFA, DCM, H_2O, rt, 2 h, 97%

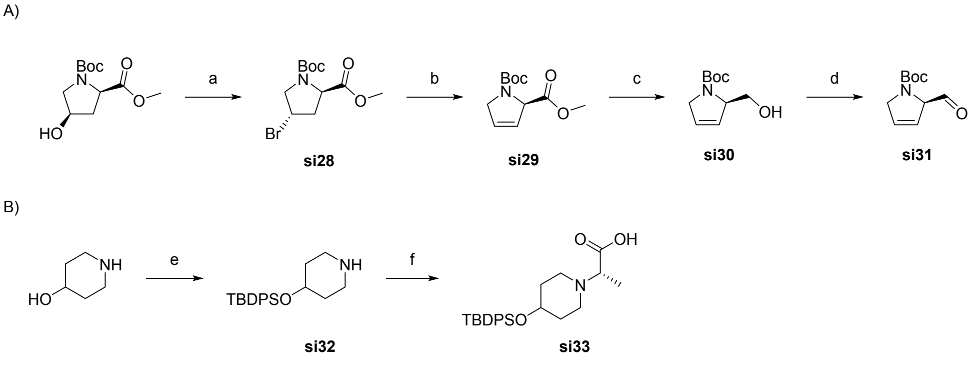

Supplementary scheme 6. Synthesis of the side chain derivatives si31 and si33. Reagents and conditions: (a) CBr_3, PPh_3, DCM, rt, overnight, 72%; (b) TBAF, DMF, 50 °C, 20 min, 94%; (c) $LiAlH_4$, THF, 0 °C to rt, 30 min, 27%; (d) Dess-Martin periodinane, DCM, 0 °C, 3 h, 71%; (e) TBDPSCl, Et_3N, THF, rt, 16 h, 8%; (f) (*R*)-2-bromopropanoic acid, Et_3N, THF, rt, 5 h, 16%.

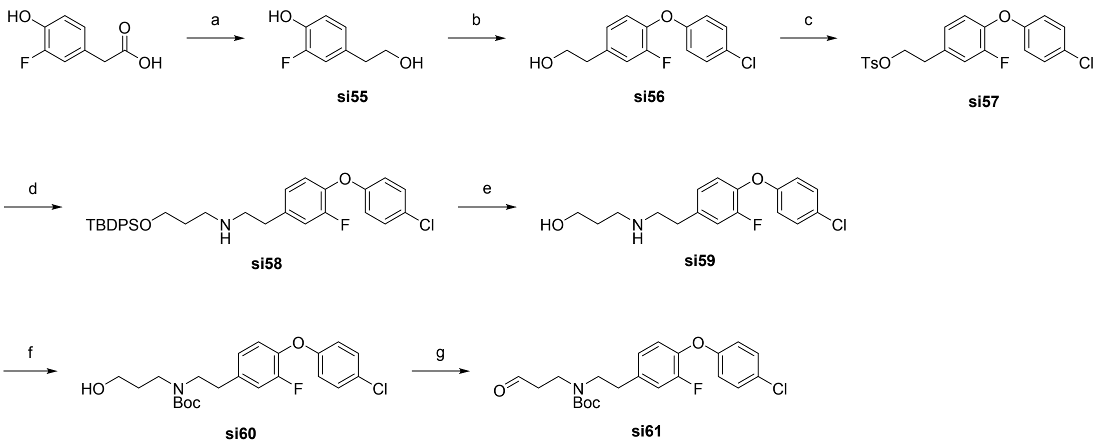

Supplementary Scheme 7. Synthesis of the side chain si61. Reagents and Conditions: (a) $NaBH_4$, Me_2SO_4, dry THF, 0 °C, 1 h, then 2-(3-fluoro-4-hydroxyphenyl) acetic acid, $B(OMe)_3$, rt, overnight; (b) 4-Chlorophenylboronic acid, dry pyridine, anhydr. $Cu(OAc)_2$, 4 Å molecular sieves, dry $C_2H_2Cl_2$, rt, 48 h, 54%; (c) TsCl, DMAP, dry pyridine, dry CH_2Cl_2, 0 °C to rt, overnight, 75%; (d) compound **si65**, $CsCO_3$, dry DMF, 80 °C, 5 h; (e) TBAF, dry THF, rt, 5 h, 60%; (f) Et_3N, Boc_2O, dry CH_2Cl_2, 0 °C to rt, 5 h, 89%; (g) Et_3N, SO_3*pyr, dry DMSO, rt, 1 h, 100%.

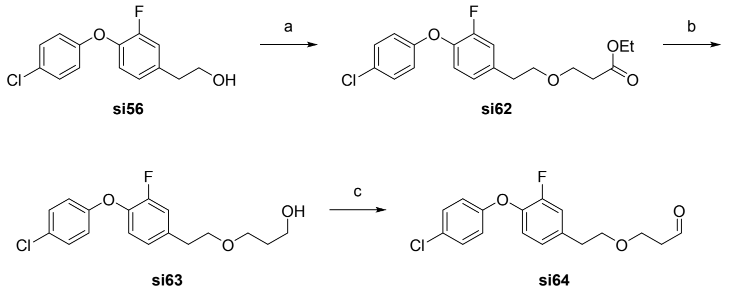

Supplementary Scheme 8. Synthesis of ether linker si64. Reagents and Conditions: (a) ethyl acrylate, Cs_2CO_3, ACN, 35 °C, overnight, 97%; (b) $LiAlH_4$, THF, rt, 1 h, 93%; (c) Dess-Martin periodinane, DCM, rt, overnight, 82%.

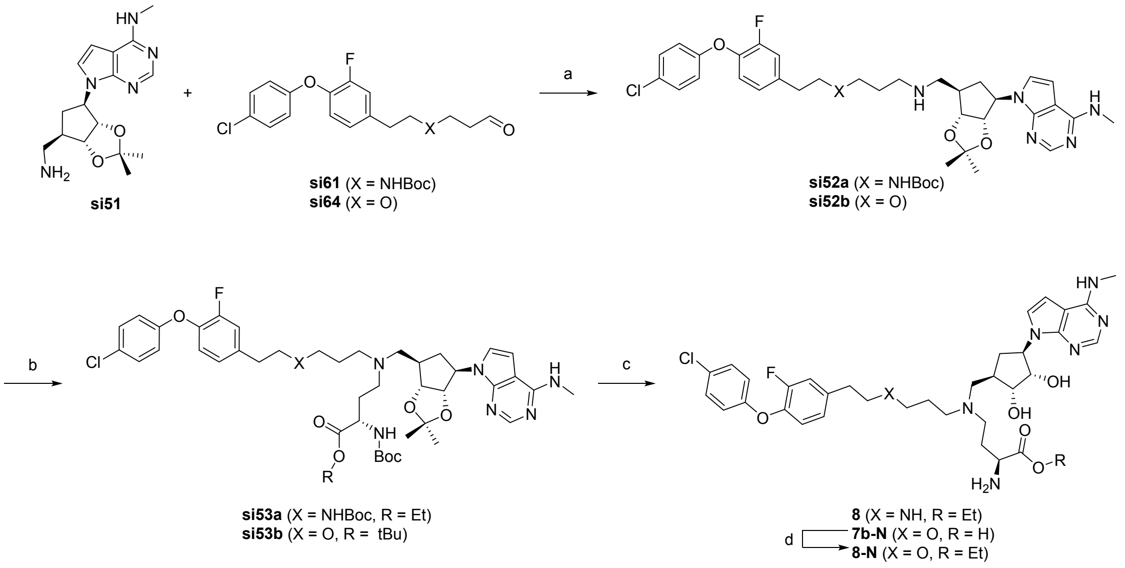

Supplementary Scheme 9. Synthesis of the final compounds 8, 7b-N and 8-N. Reagents and Conditions: (a) EtOH, rt, 1 h, then $NaBH_3CN$, rt, 12 h, 27% (for **si52a**); or MeOH, rt, 30 min, then $NaBH_3CN$, rt, 16 h, 14% (for **si52b**); (b) ethyl (*S*)-2-((*tert*-butoxycarbonyl)amino)-4-oxobutanoate, EtOH, rt, 1 h, then $NaBH_3CN$, 30 °C, 12 h, 51% (for **si53a**); or compound **si6**, EtOH, rt, 30 min, then $NaBH_3CN$, rt, 16 h, 53% (for **si53b**); (c) TFA, DCM, rt, 2 h, 83% (for **si54a**); or 3 h, 86% (**for si54b**); (d) TMSCl, EtOH, 68 °C, 5 h, 38% (**si54c**)

HPLC purity, Mass spectrum and ^{1}H-NMR spectra of all compounds

Compound **1** = (Synthesis: **9j**)

HPLC purity

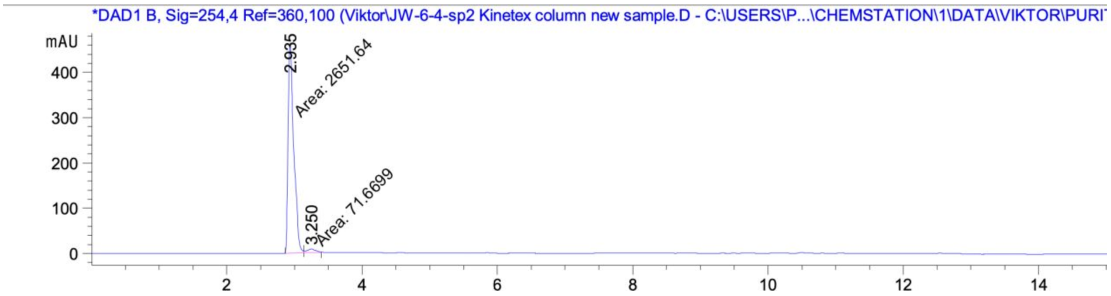

Signal 2: DAD1 B, Sig=254,4 Ref=360,100
Signal has been modified after loading from rawdata file!

Peak #	RetTime [min]	Type	Width [min]	Area [mAU*s]	Height [mAU]	Area %
1	2.935	PM	0.0952	2651.63599	464.29471	97.3683
2	3.250	MM	0.1555	71.66988	7.67990	2.6317

Totals : 2723.30587 471.97461

Mass spectrum

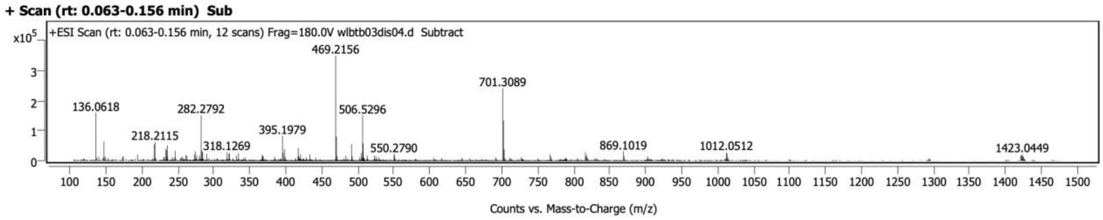

^{1}H-NMR

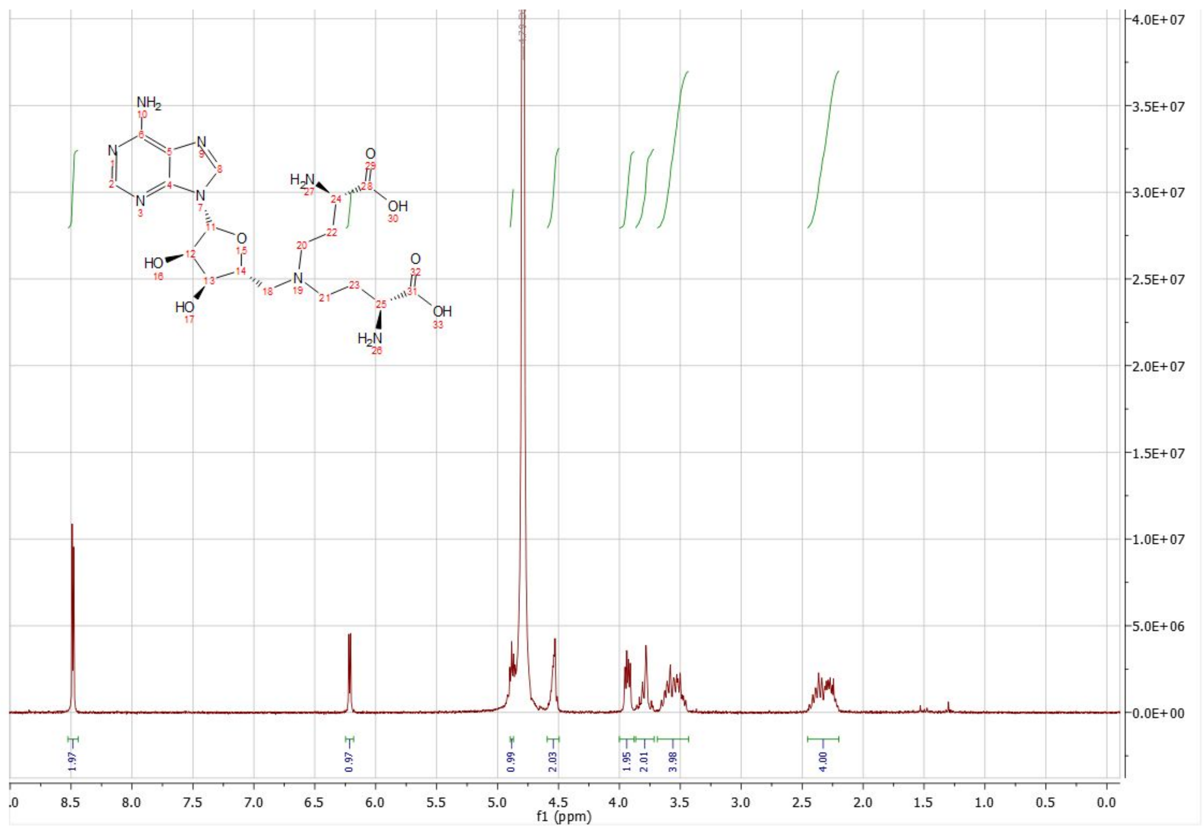

Compound **2a** = (Synthesis: **9c**)

HPLC purity

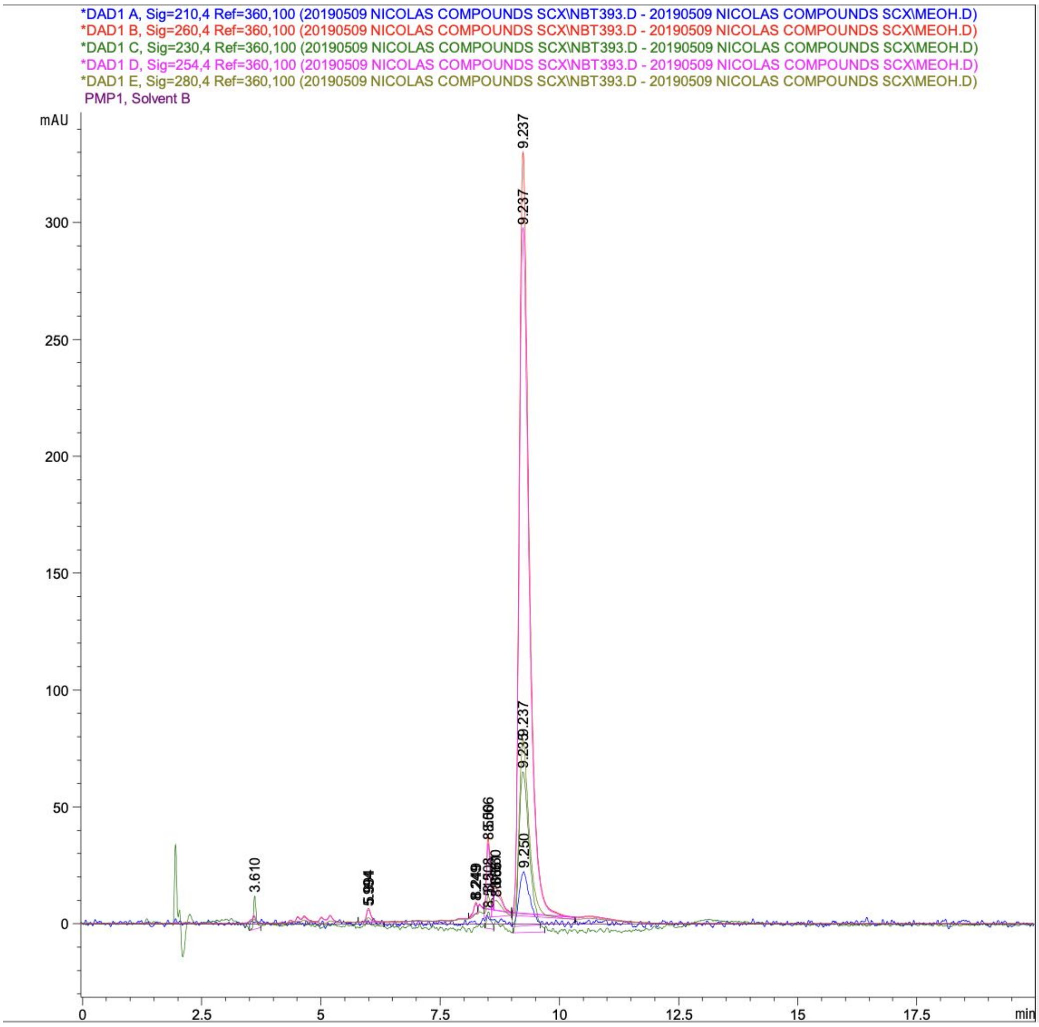

Signal 4: DAD1 D, Sig=254,4 Ref=360,100
Signal has been modified after loading from rawdata file!

Peak #	RetTime [min]	Type	Width [min]	Area [mAU*s]	Height [mAU]	Area %
1	5.994	BB	0.1048	36.11717	5.21532	0.7989
2	8.249	BV	0.0793	21.53014	4.09481	0.4763
3	8.506	BV	0.0844	160.64333	28.20704	3.5536
4	8.661	VB	0.1477	81.50098	7.68797	1.8029
5	9.237	BB	0.2178	4220.81250	293.39557	93.3683

Totals : 4520.60412 338.60071

Mass spectrum

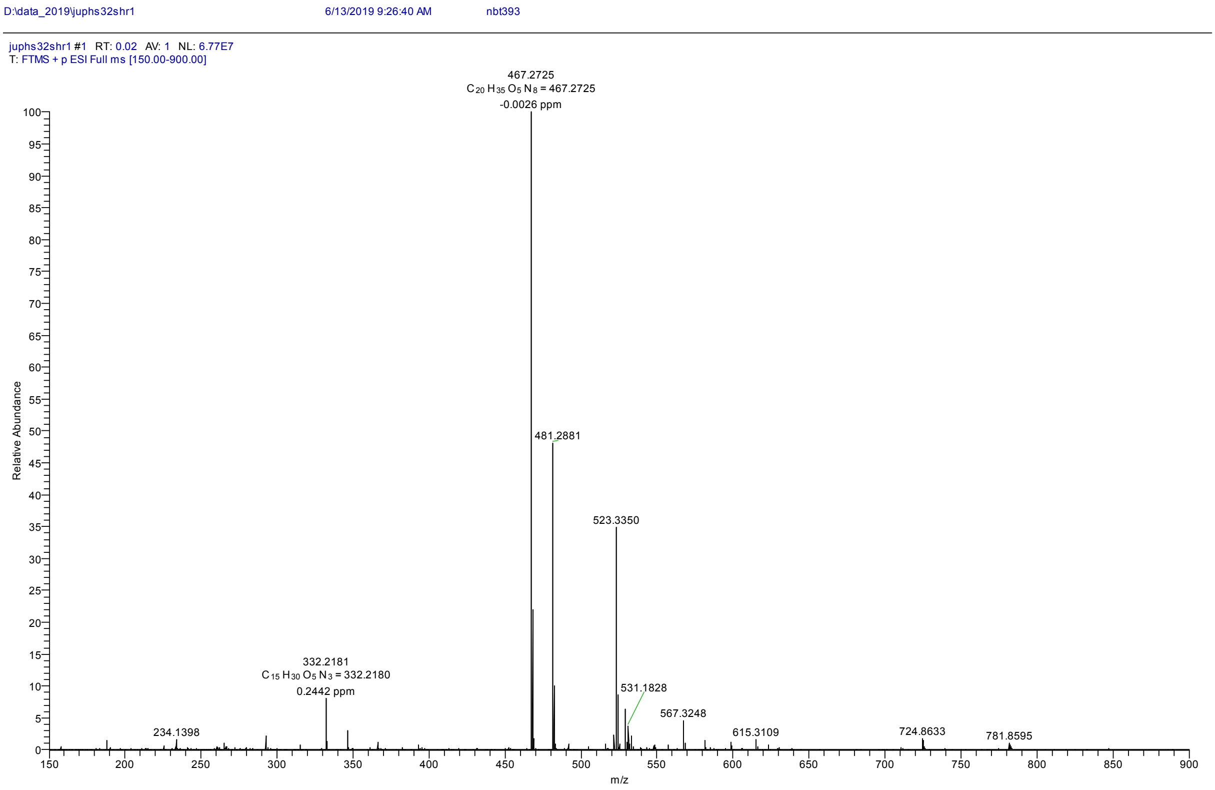

^{1}H-NMR

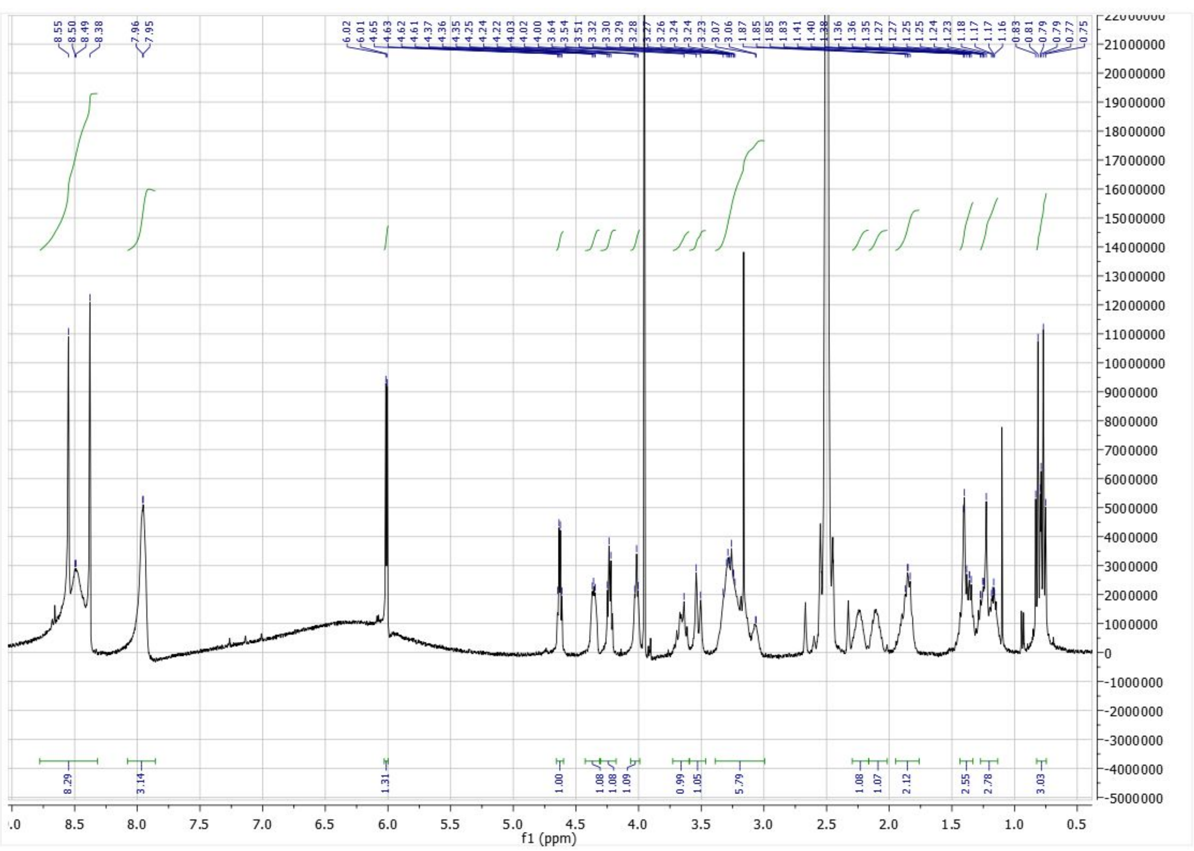

Compound **2b** = (Synthesis: **39**)

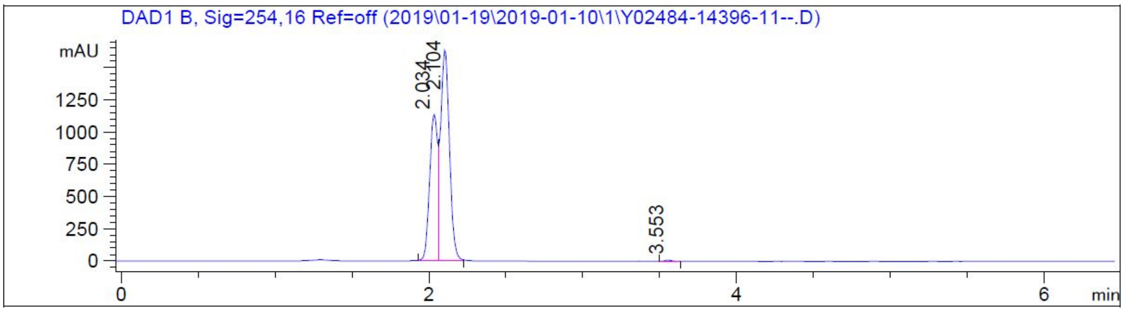

Signal 2: DAD1 B, Sig=254,16 Ref=off

#	R.T.	Type	Height	Height%	Width	Area	Area %
1	2.034	BV	1135.392	40.785	0.055	4025.411	38.487
2	2.104	MF	1640.957	58.945	0.065	6409.782	61.284
3	3.553	BB	7.524	0.270	0.049	23.879	0.228

Mass spectrum

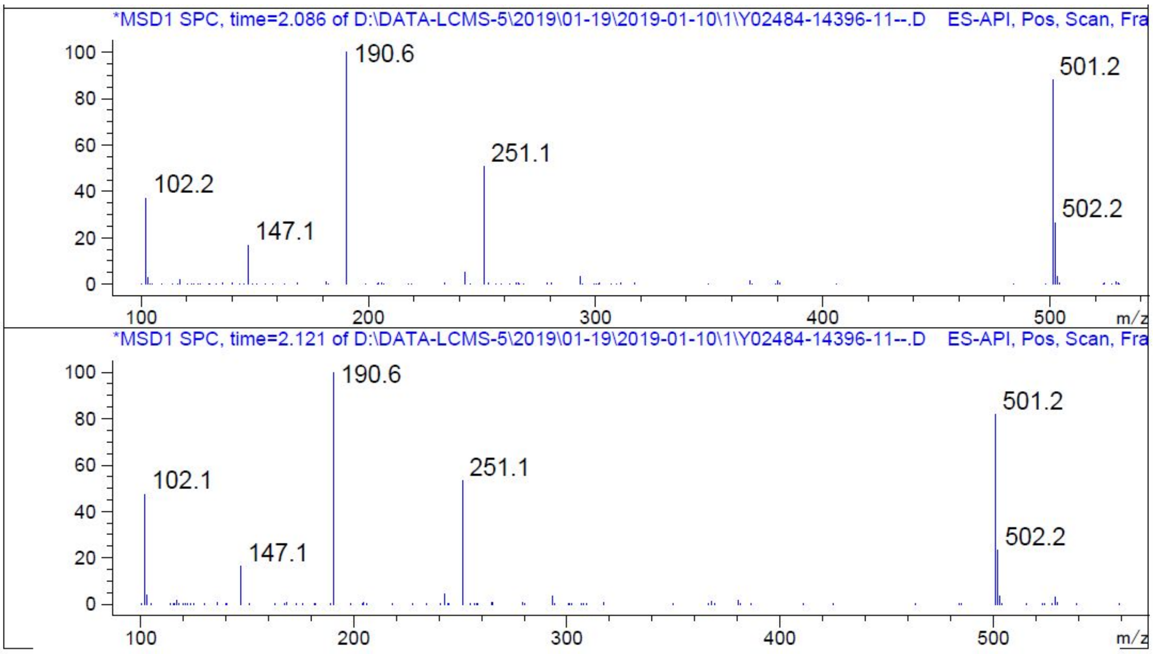

^{1}H-NMR

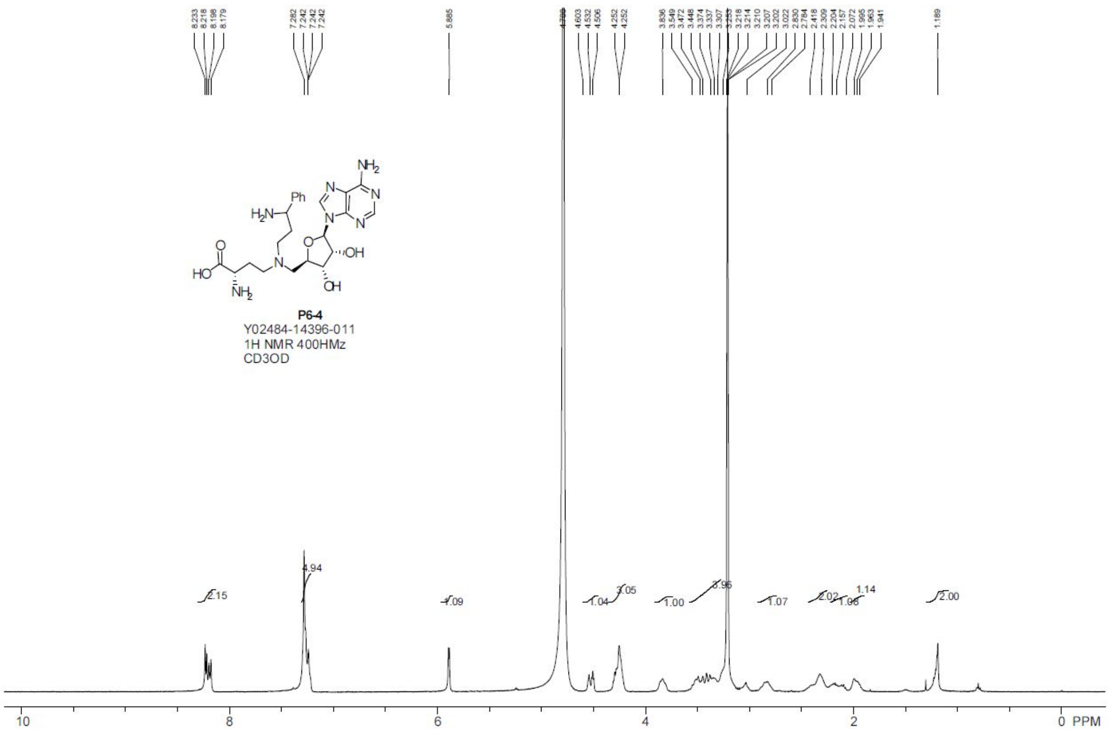

Compound **2c** = (Synthesis: **9b**)

HPLC purity

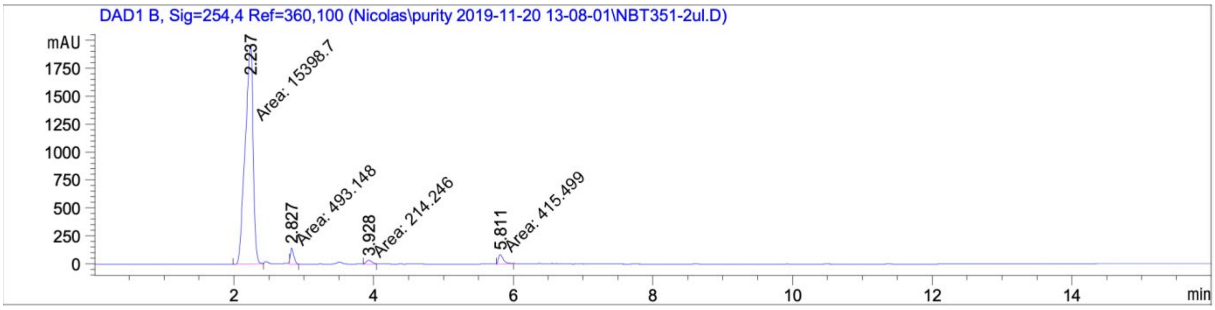

Signal 2: DAD1 B, Sig=254,4 Ref=360,100

Peak #	RetTime [min]	Type	Width [min]	Area [mAU*s]	Height [mAU]	Area %
1	2.237	PM	0.1304	1.53987e4	1968.24084	93.2035
2	2.827	MM	0.0564	493.14819	145.79680	2.9849
3	3.928	MM	0.1022	214.24625	34.95581	1.2968
4	5.811	MM	0.0815	415.49860	85.00331	2.5149

Totals : 1.65216e4 2233.99677

Mass spectrum:

D:\data_2019\juphr99shr2 2/14/2019 9:39:41 AM nbt351

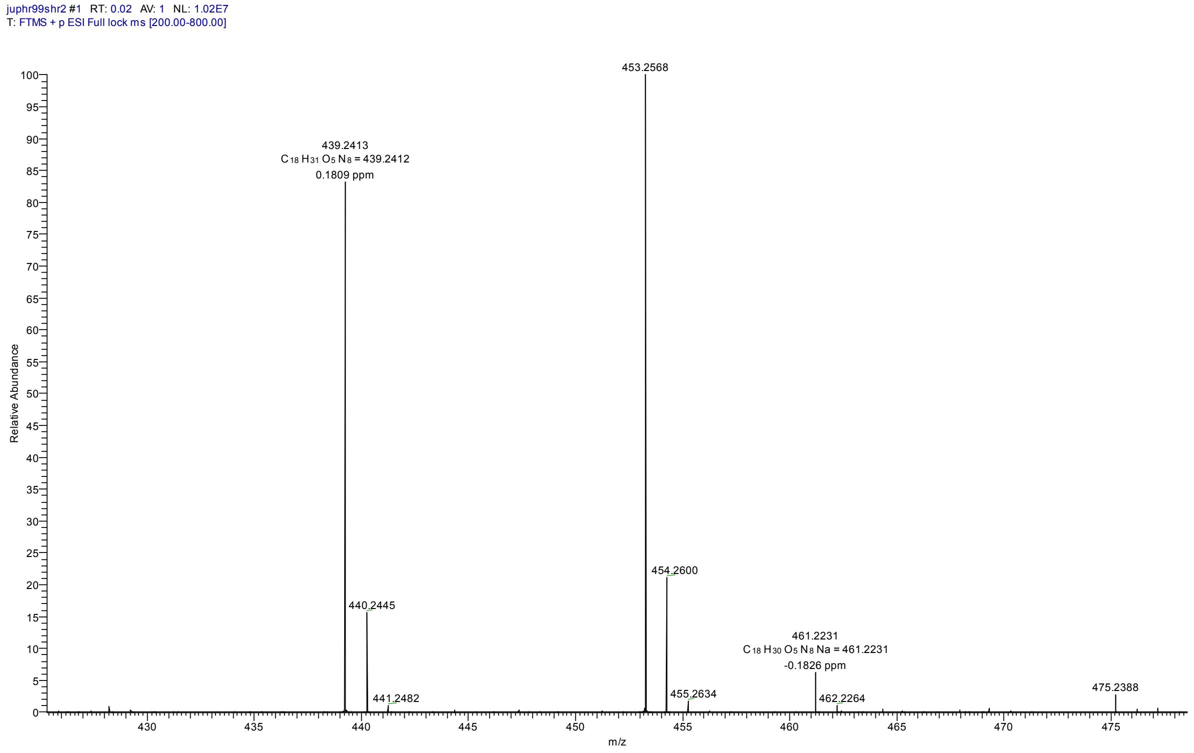

^{1}H-NMR

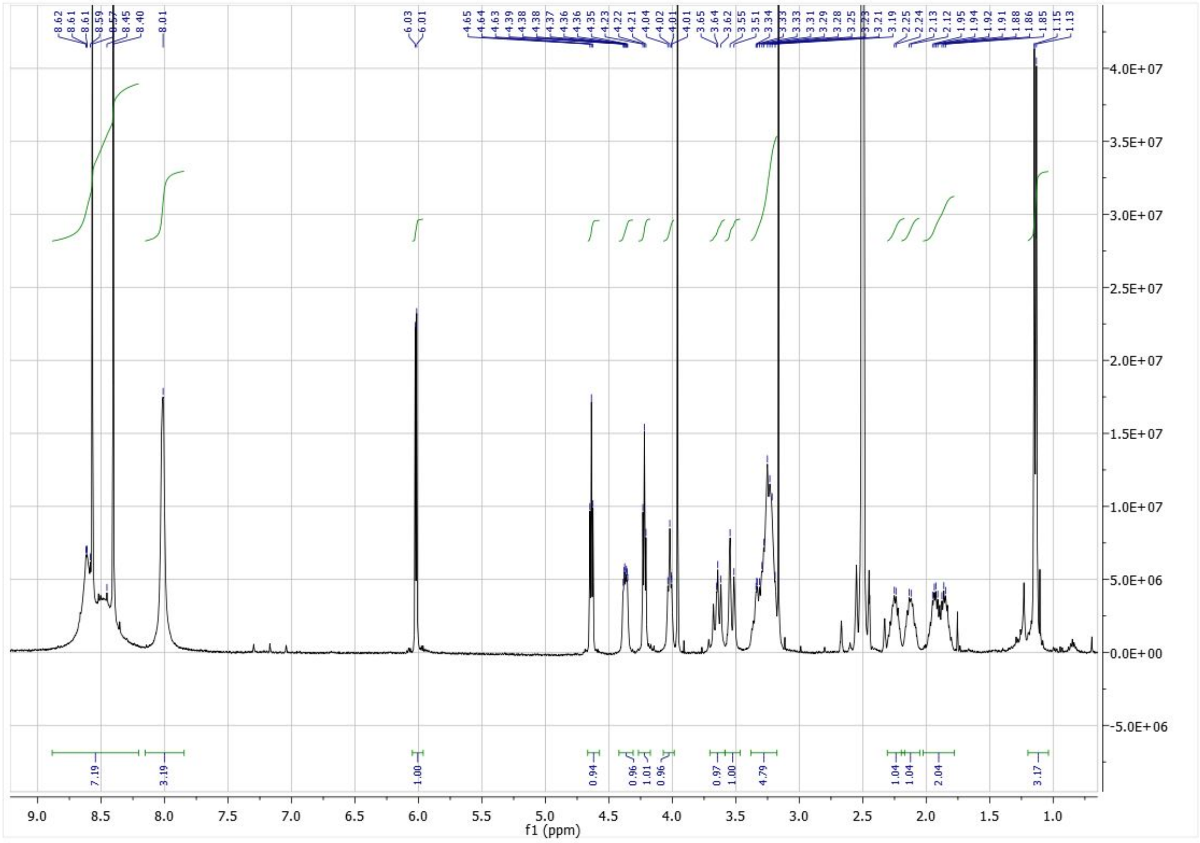

Compound **2d** = (Synthesis: **9d**)

HPLC purity

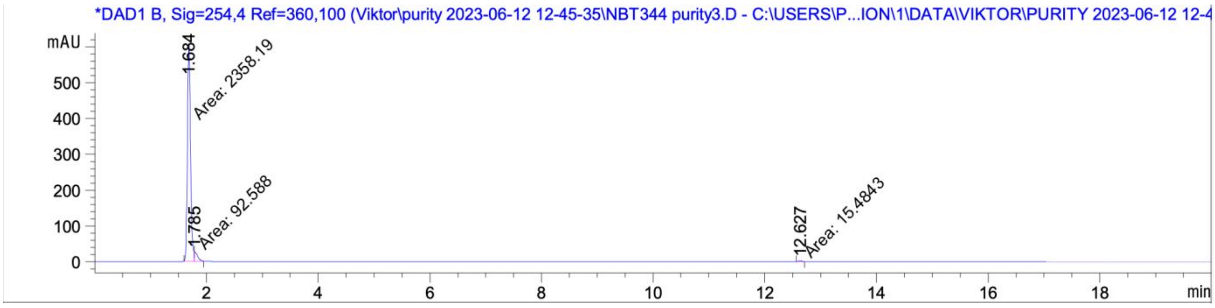

Signal 2: DAD1 B, Sig=254,4 Ref=360,100
Signal has been modified after loading from rawdata file!

Peak #	RetTime [min]	Type	Width [min]	Area [mAU*s]	Height [mAU]	Area %
1	1.684	PM	0.0645	2358.18896	609.33386	95.6180
2	1.785	MM	0.0619	92.58804	24.93105	3.7542
3	12.627	MM	0.0715	15.48429	3.60964	0.6278
Totals :				2466.26130	637.87455	

Mass spectrum

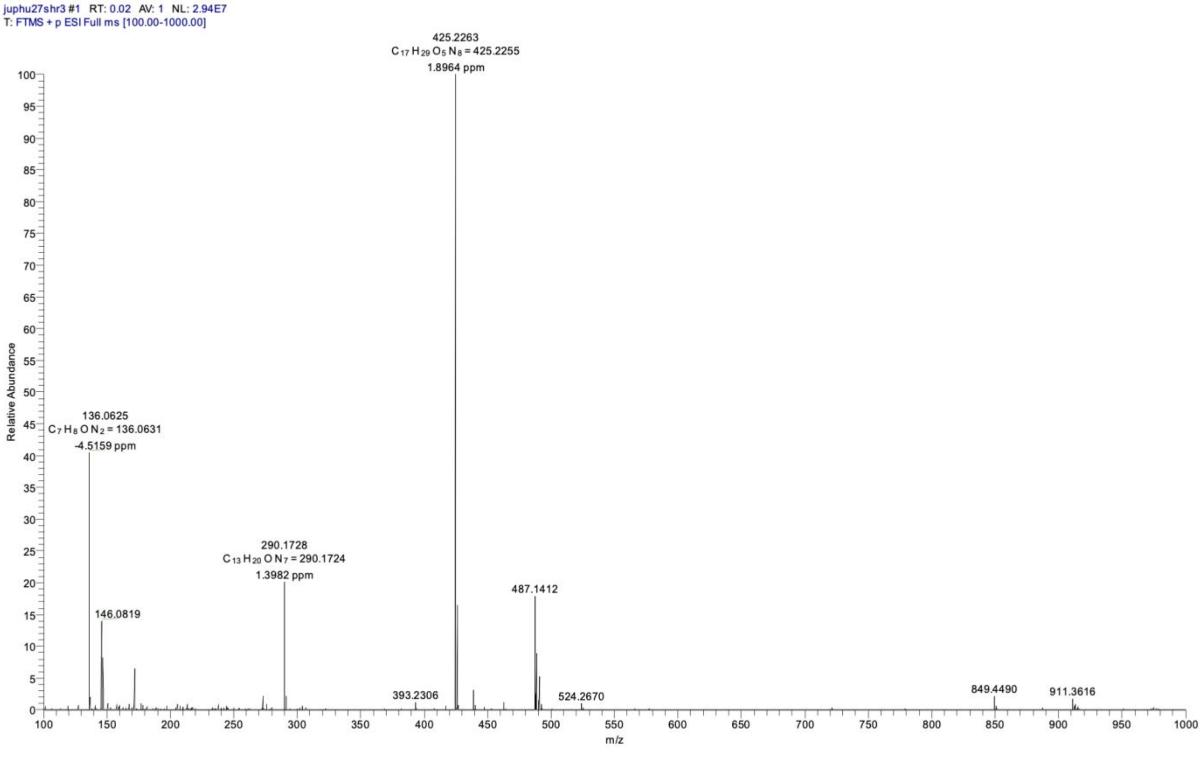

^{1}H-NMR

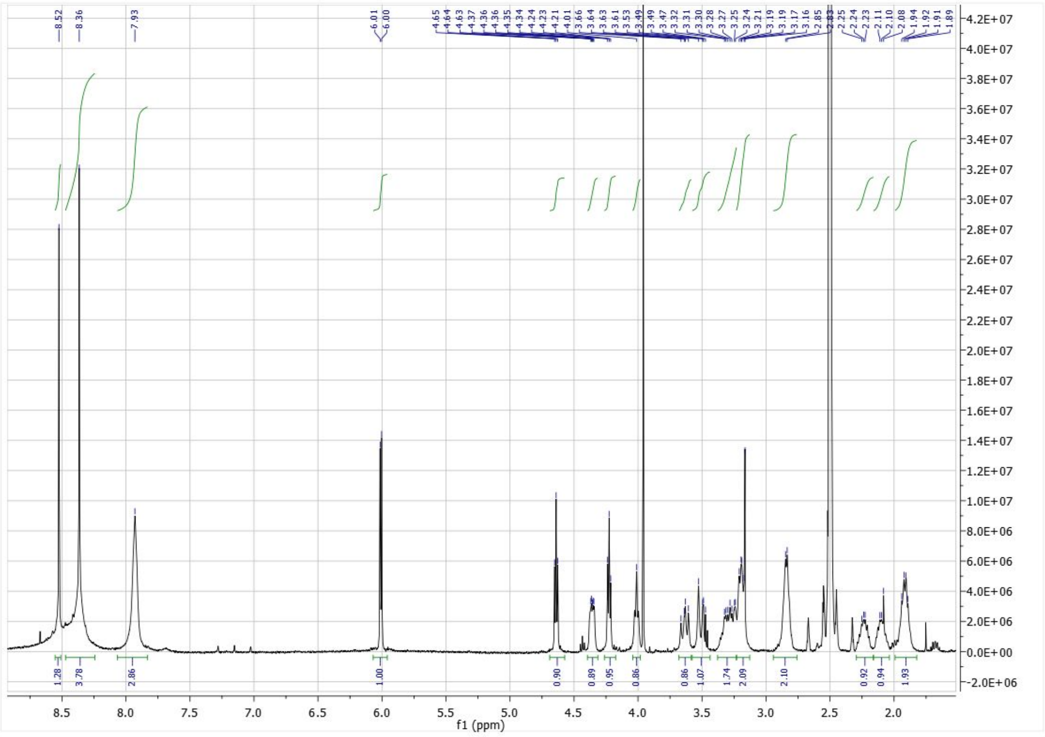

Compound **3a** = (Synthesis: **9e**)

HPLC purity

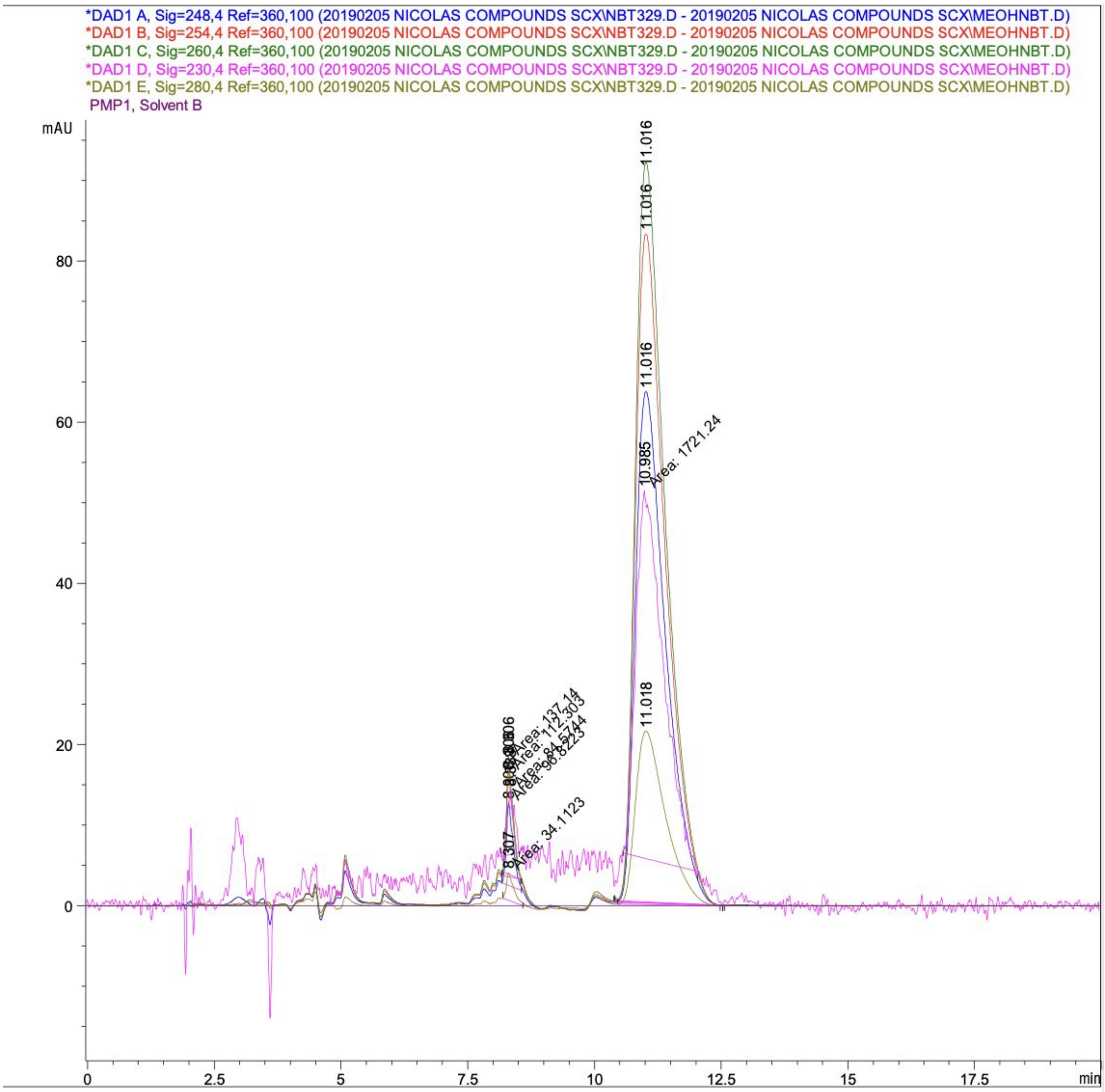


```
Signal 2: DAD1 B, Sig=254,4 Ref=360,100
 Signal has been modified after loading from rawdata file!

Peak RetTime Type  Width     Area      Height     Area
  #   [min]        [min]   [mAU*s]     [mAU]        %
----|-------|----|-------|----------|----------|--------|
   1   8.306 MM    0.1474  112.30278   12.69404   3.1506
   2  11.016 BB    0.6155 3452.15259   82.96428  96.8494

Totals :                  3564.45537   95.65832
```

Mass spectrum

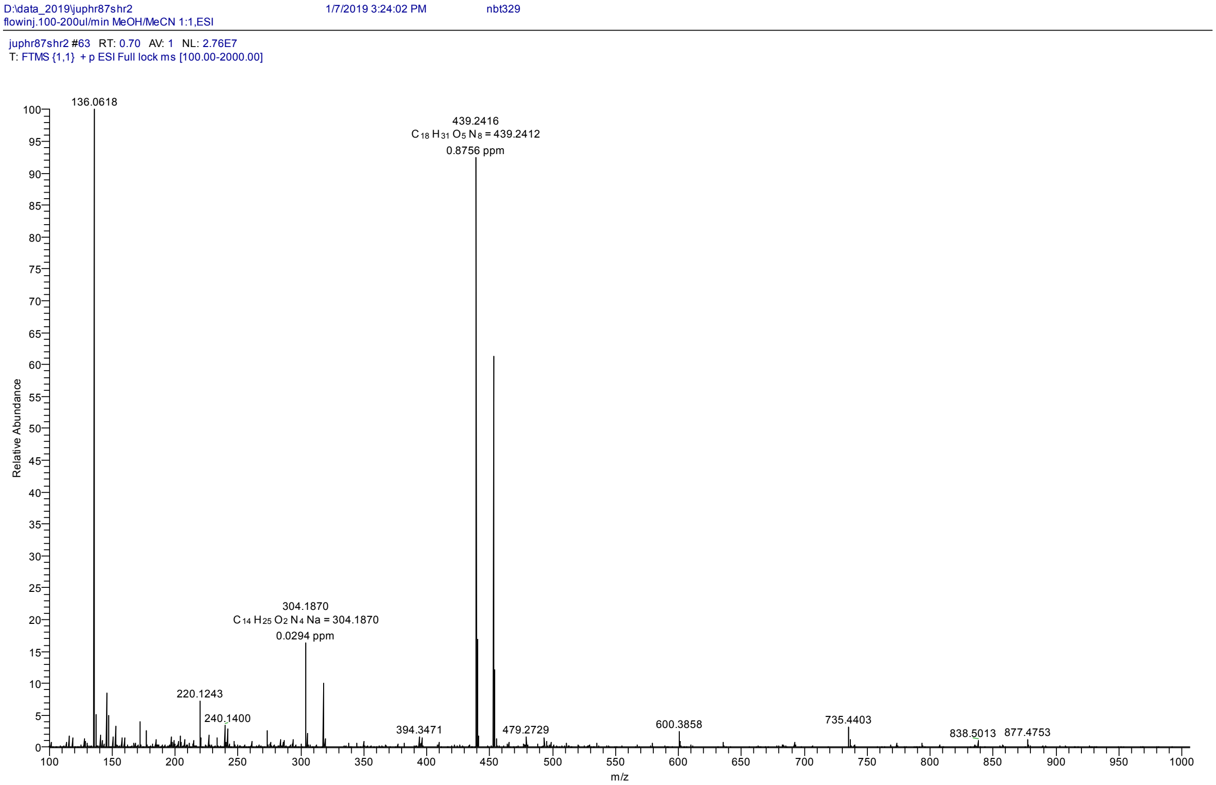
D:\data_2019\juphr87shr2
flowinj.100-200ul/min MeOH/MeCN 1:1,ESI
1/7/2019 3:24:02 PM
nbt329
juphr87shr2 #63 RT: 0.70 AV: 1 NL: 2.76E7
T: FTMS {1,1} + p ESI Full lock ms [100.00-2000.00]
136.0618
439.2416
C 18 H 31 O 5 N 8 = 439.2412
0.8756 ppm
304.1870
C 14 H 25 O 2 N 4 Na = 304.1870
0.0294 ppm
220.1243
240.1400
394.3471
479.2729
600.3858
735.4403
838.5013
877.4753
Relative Abundance
m/z

^{1}H-NMR

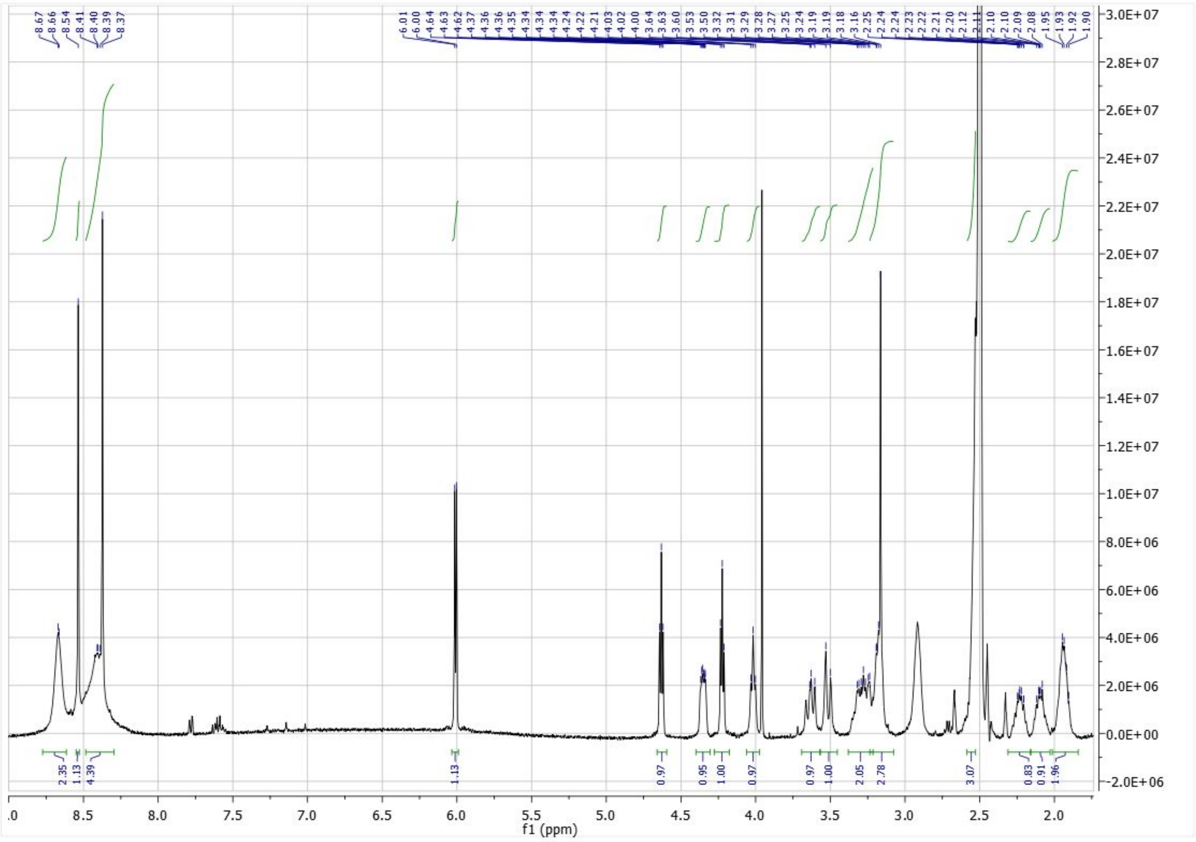
f1 (ppm)

Compound **3c** = (Synthesis: **9f**)

HPLC purity

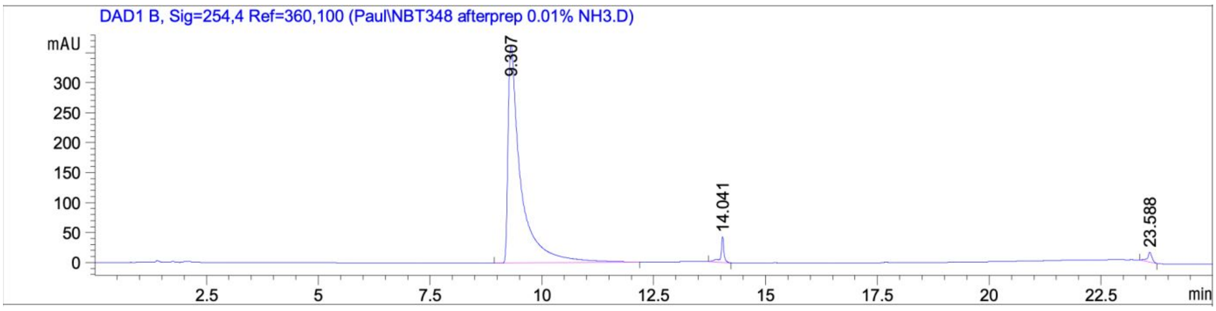

Signal 2: DAD1 B, Sig=254,4 Ref=360,100

Peak #	RetTime [min]	Type	Width [min]	Area [mAU*s]	Height [mAU]	Area %
1	9.307	BB	0.2765	7230.14844	362.85773	95.9235
2	14.041	VB R	0.0565	198.63673	42.68462	2.6353
3	23.588	BB	0.0975	108.62453	16.35922	1.4411
Totals :				7537.40971	421.90157	

Mass spectrum

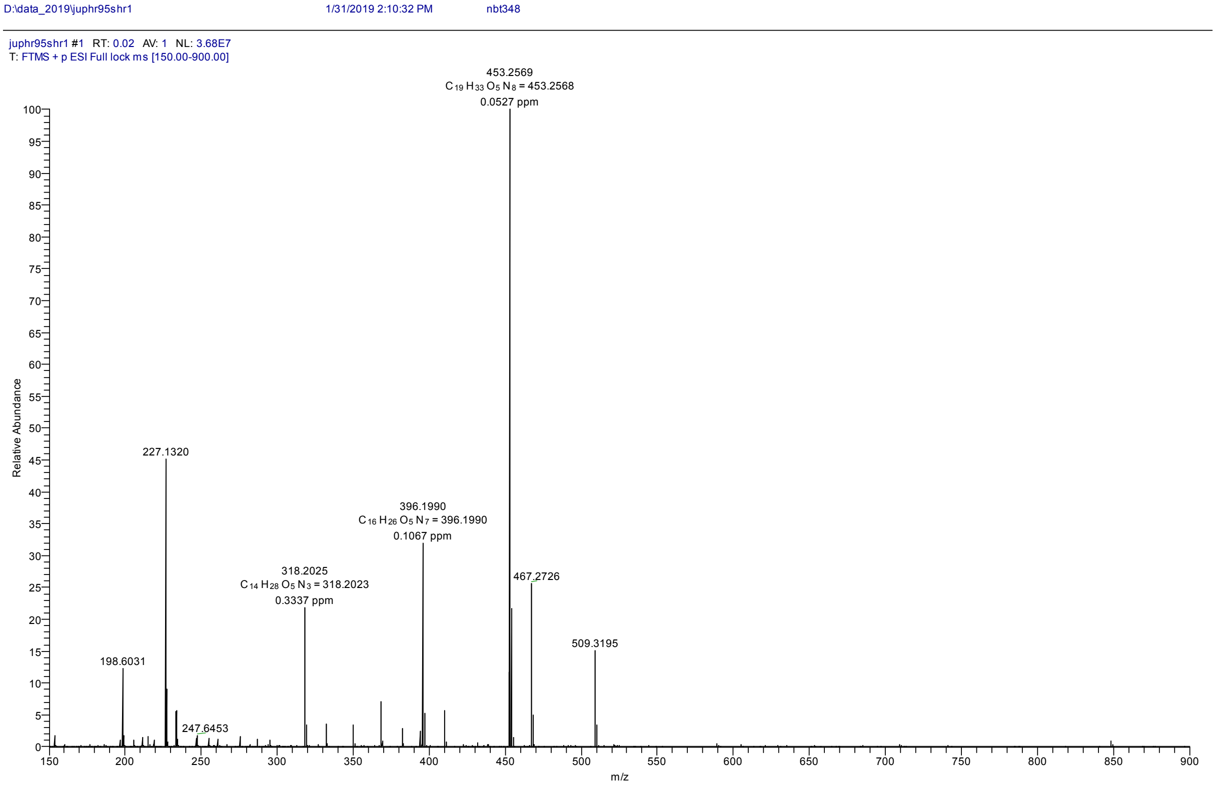

^{1}H-NMR

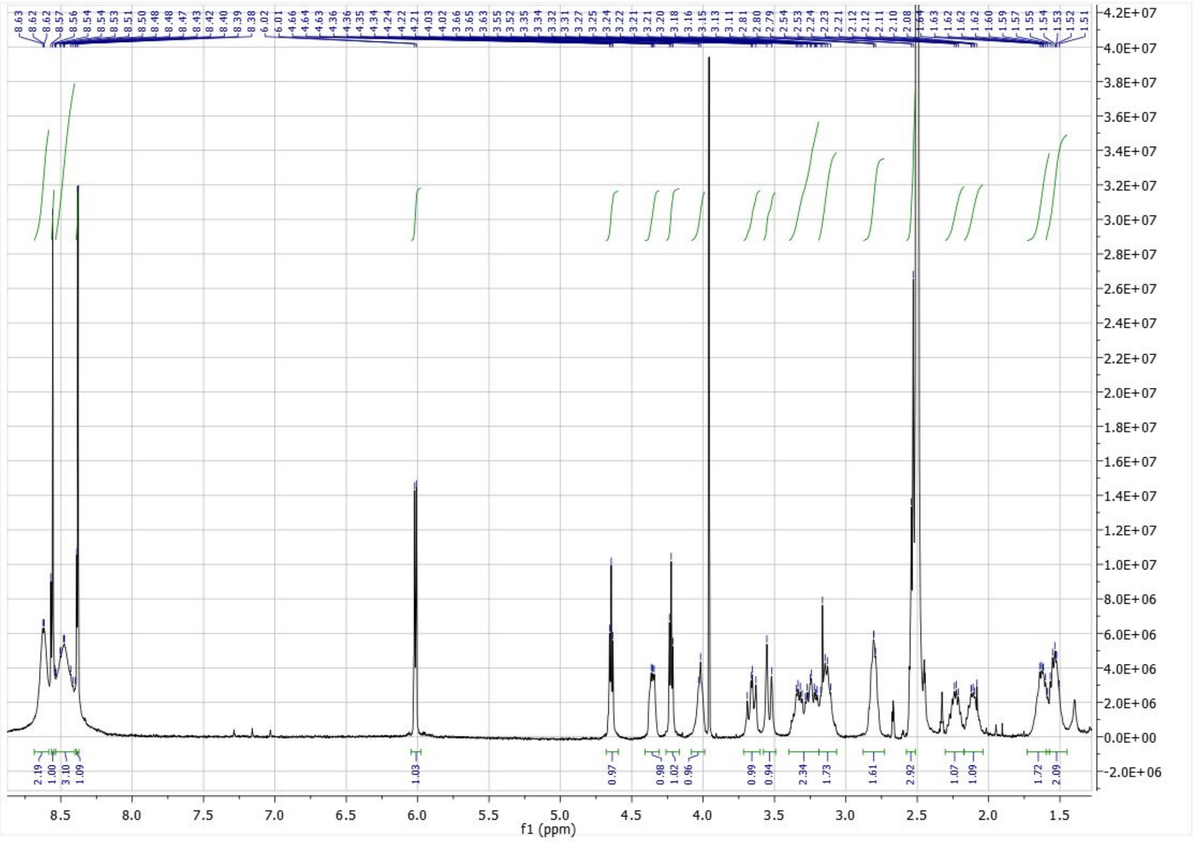

Compound **3d**: (Synthesis: **9a**)

HPLC purity

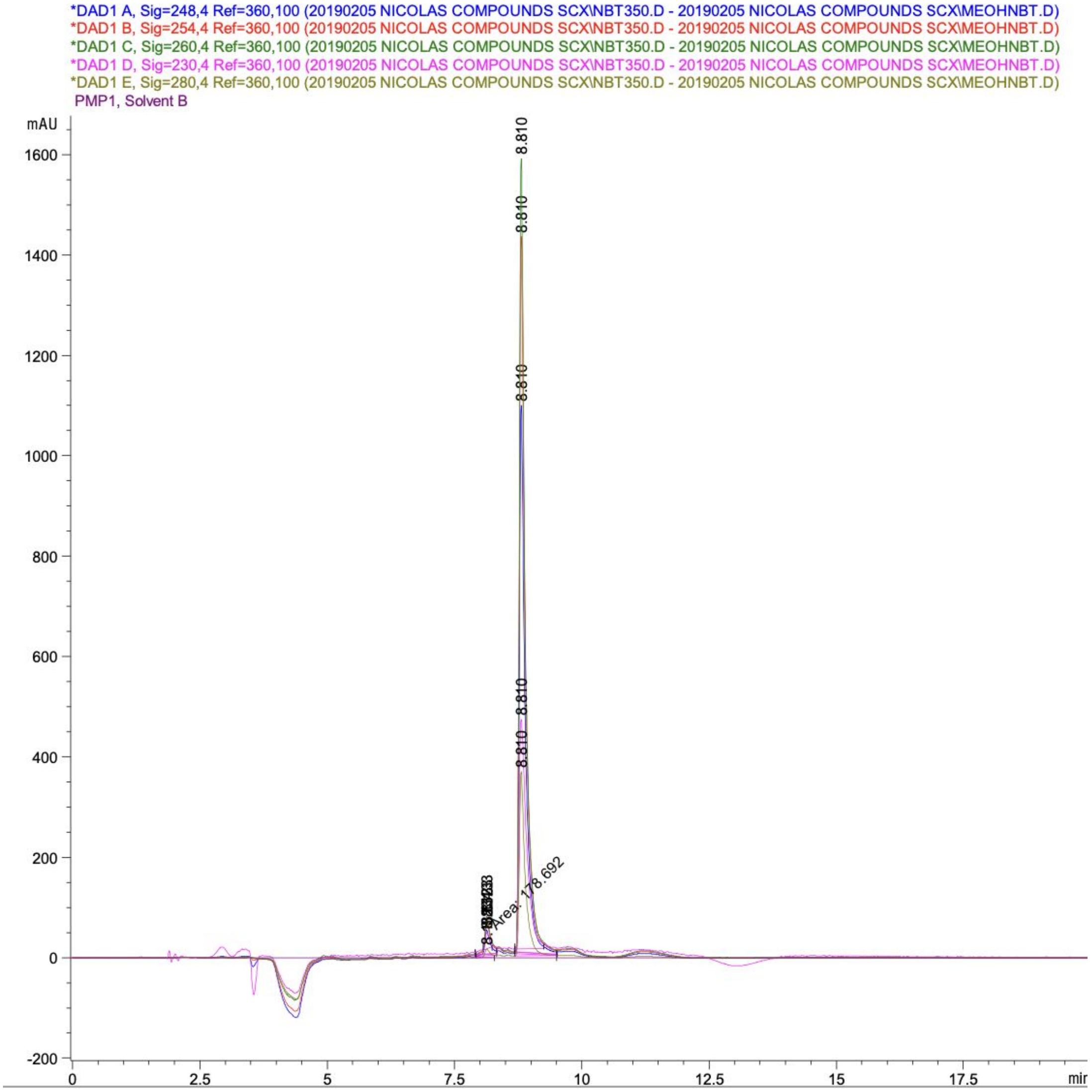


```
Signal 2: DAD1 B, Sig=254,4 Ref=360,100
 Signal has been modified after loading from rawdata file!

Peak RetTime Type  Width     Area       Height      Area
  #   [min]        [min]   [mAU*s]      [mAU]        %
----|-------|----|-------|----------|----------|--------|
   1   8.133 BV    0.1098  531.63647   67.60066    4.3214
   2   8.810 BV    0.1162 1.17706e4  1427.60486   95.6786

Totals :                  1.23023e4  1495.20552
```

Mass spectrum

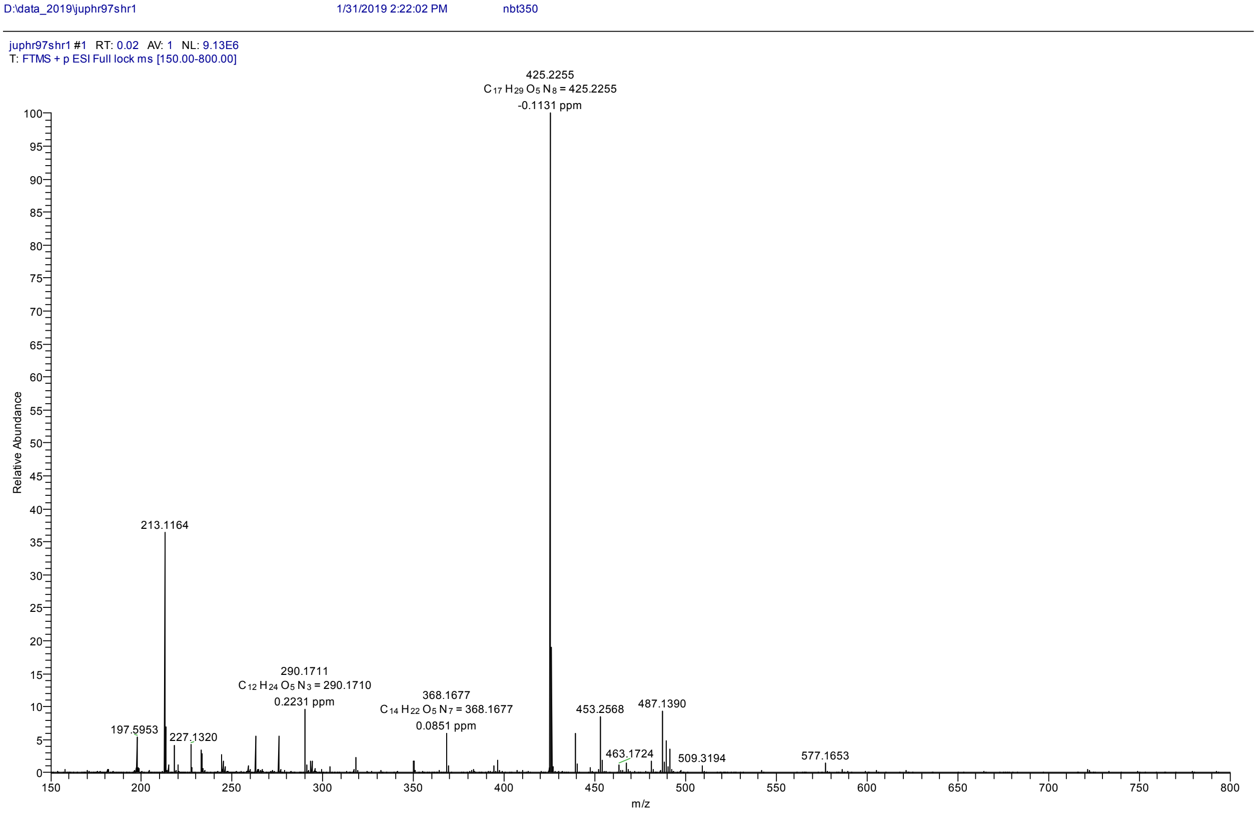

^{1}H-NMR

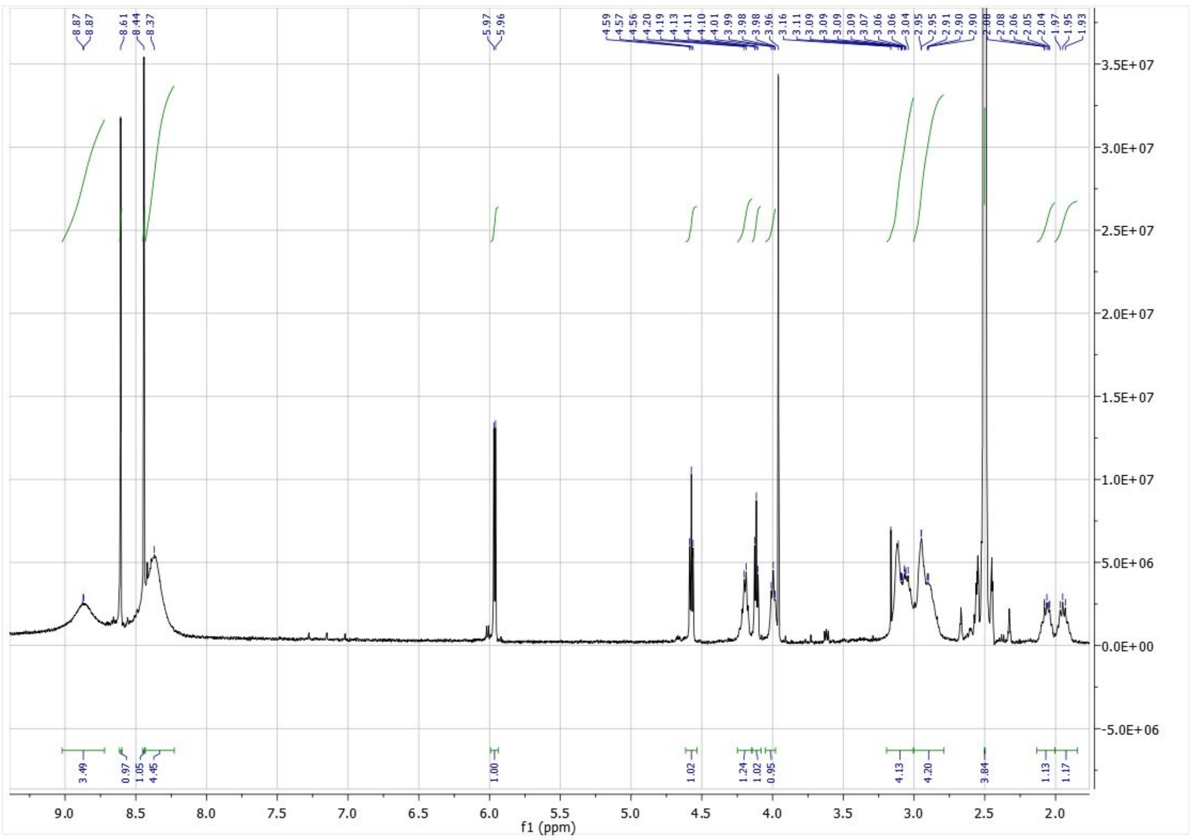

Compound **4a**

HPLC purity

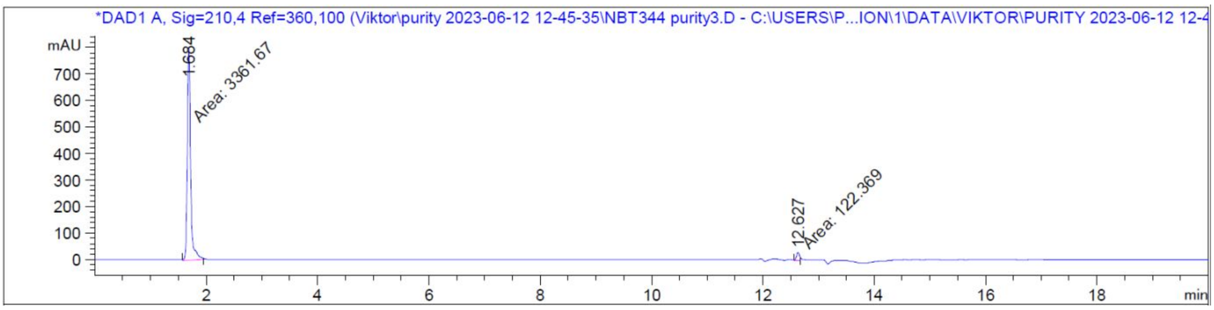


```
Signal 1: DAD1 A, Sig=210,4 Ref=360,100
 Signal has been modified after loading from rawdata file!

Peak RetTime Type  Width     Area      Height     Area
  #   [min]        [min]   [mAU*s]     [mAU]        %
----|-------|----|-------|----------|----------|--------|
   1   1.684 MM    0.0694 3361.66553  807.75366  96.4877
   2  12.627 MM    0.0668  122.36874   30.52597   3.5123

Totals :                  3484.03427  838.27964
```

Mass spectrum:

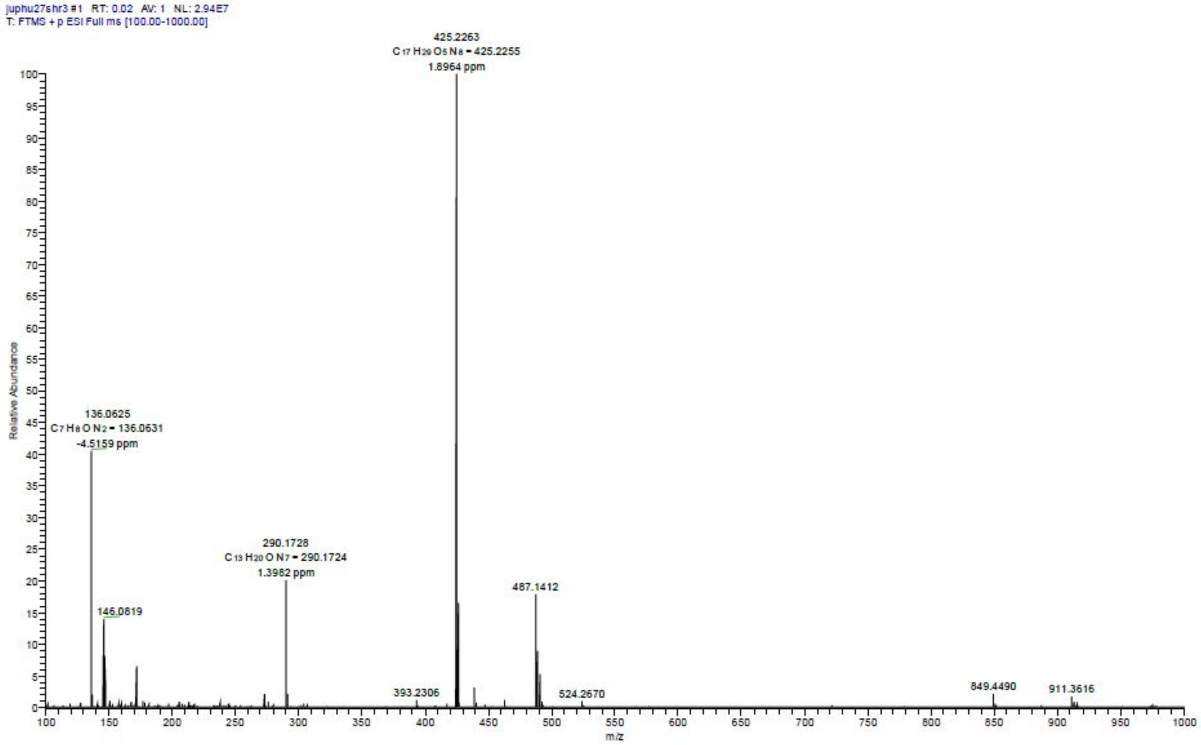

^{1}H-NMR

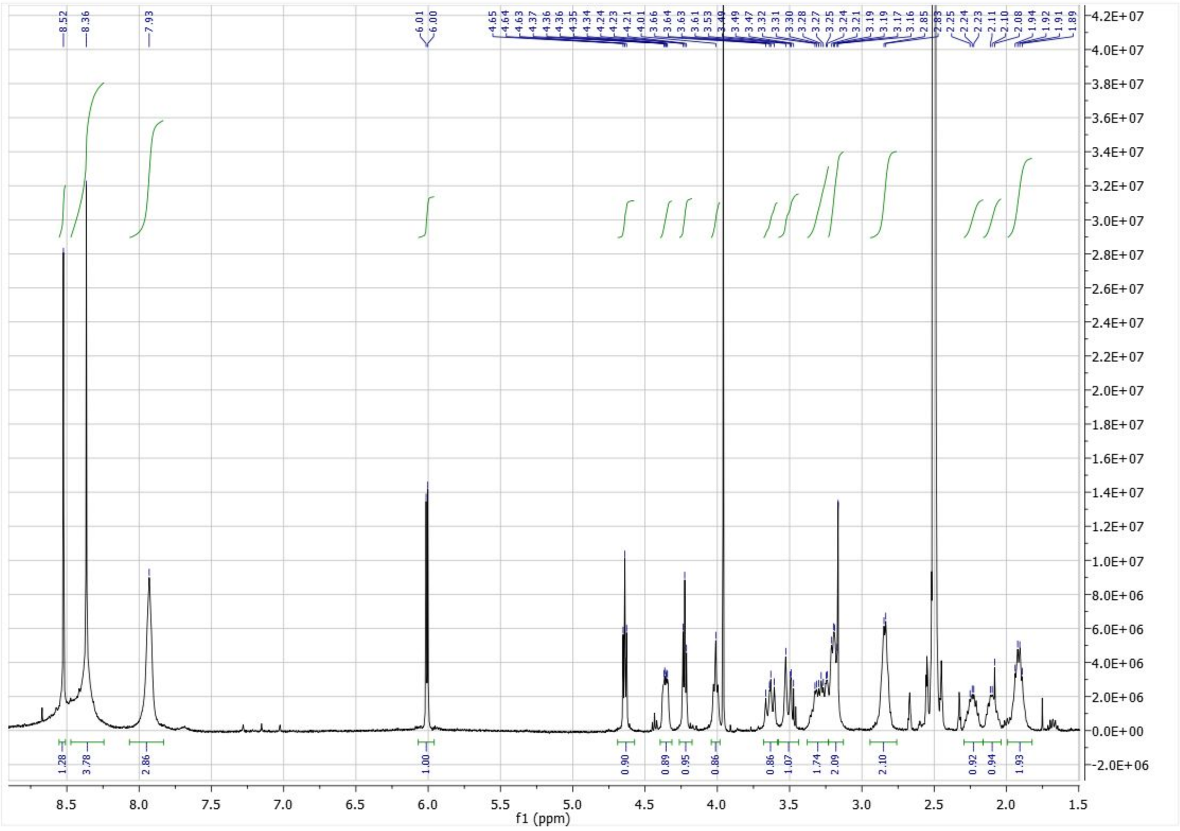

Compound **4b** = (Synthesis: **17a**)

HPLC purity (UV-Vis; 254 nm). 100 % (mixture of two diastereomers)

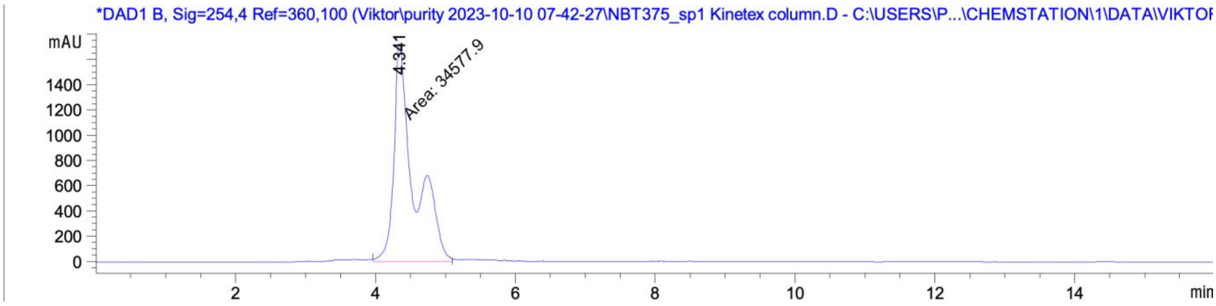

Mass spectrum; positive ESI

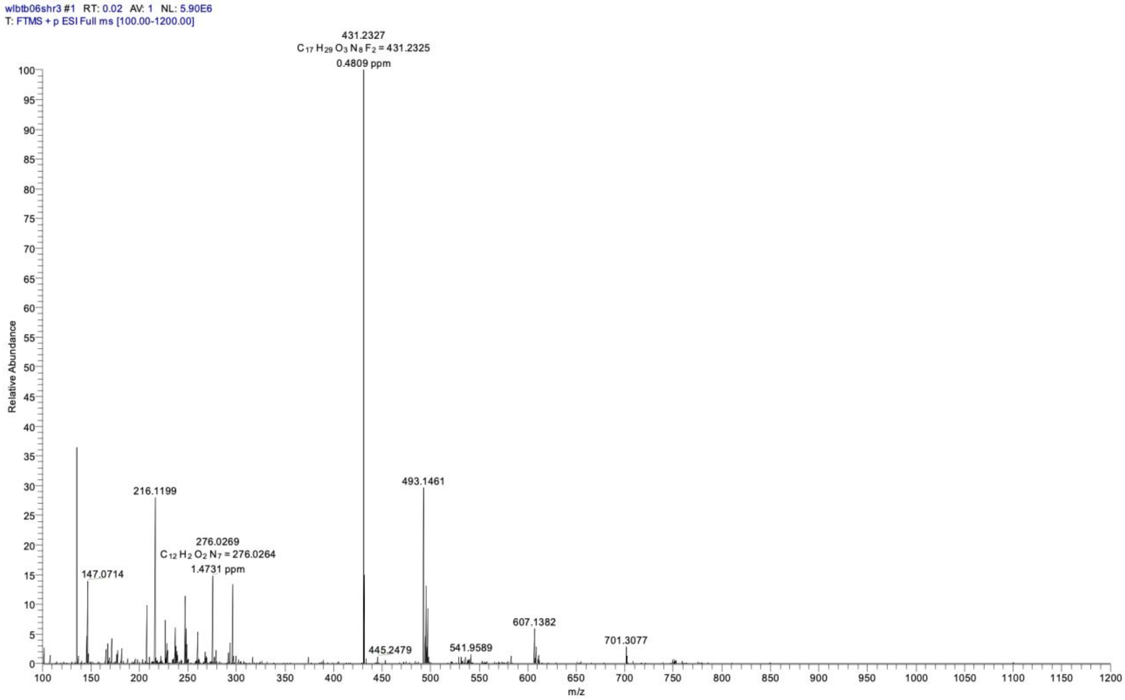
wlbtb06shr3 #1 RT: 0.02 AV: 1 NL: 5.90E6
T: FTMS + p ESI Full ms [100.00-1200.00]
431.2327
$C_{17}H_{29}O_3N_8F_2$ = 431.2325
0.4809 ppm
216.1199
276.0269
$C_{12}H_2O_2N_7$ = 276.0264
1.4731 ppm
147.0714
493.1461
445.2479
541.9589
607.1382
701.3077
Relative Abundance
m/z

^{1}H-NMR

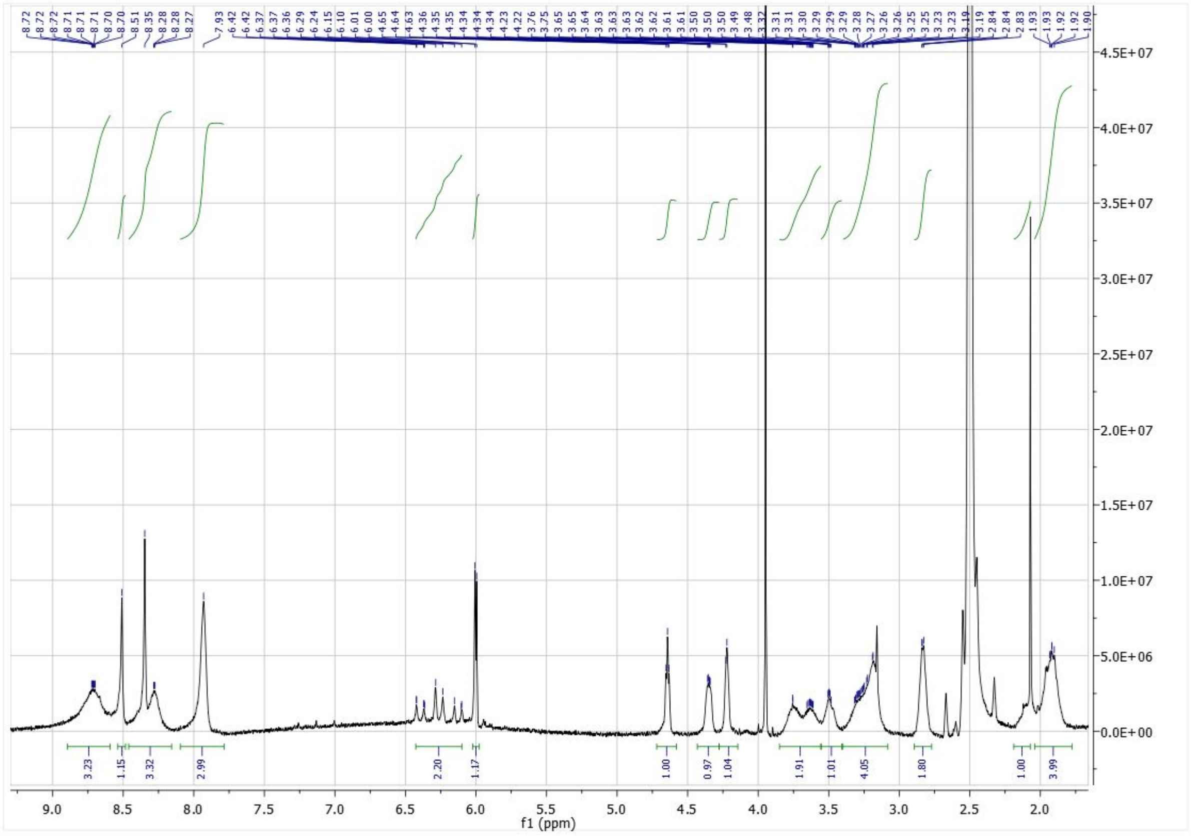
3.23
1.15
3.32
2.99
2.20
1.17
1.00
0.97
1.04
1.91
1.01
4.05
1.80
1.00
3.99
f1 (ppm)

Compound **4c** = (Synthesis: **17b**)

HPLC purity

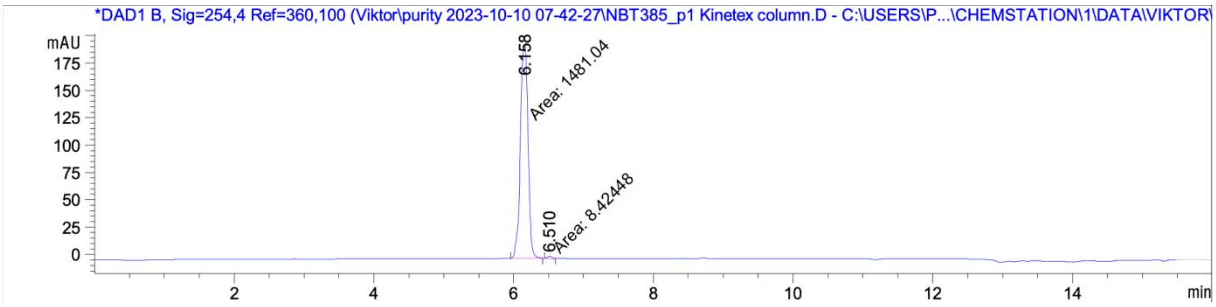

Signal 2: DAD1 B, Sig=254,4 Ref=360,100
Signal has been modified after loading from rawdata file!

Peak #	RetTime [min]	Type	Width [min]	Area [mAU*s]	Height [mAU]	Area %
1	6.158	MM	0.1262	1481.04395	195.64230	99.4344
2	6.510	MM	0.0712	8.42448	1.97204	0.5656
Totals :				1489.46843	197.61435	

Mass spectrum

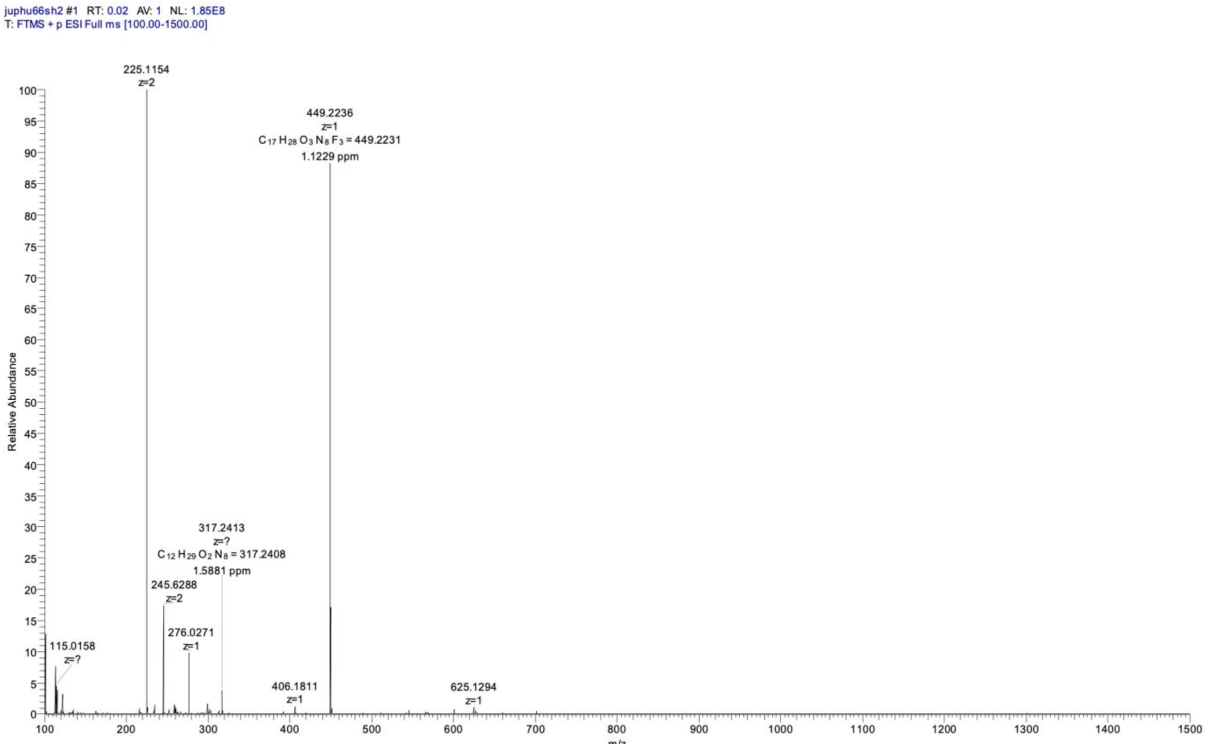

^{1}H-NMR

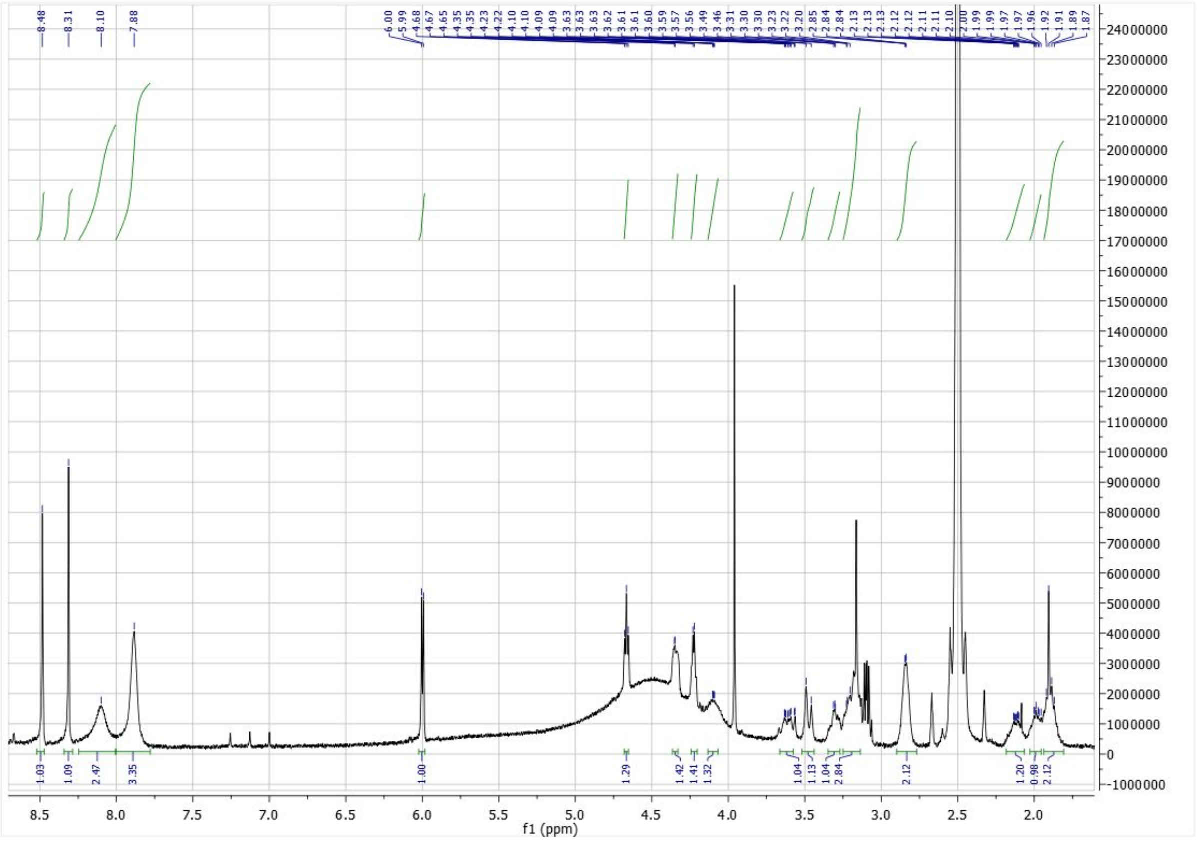

Compound **4d** = (Synthesis: **41**)

HPLC purity

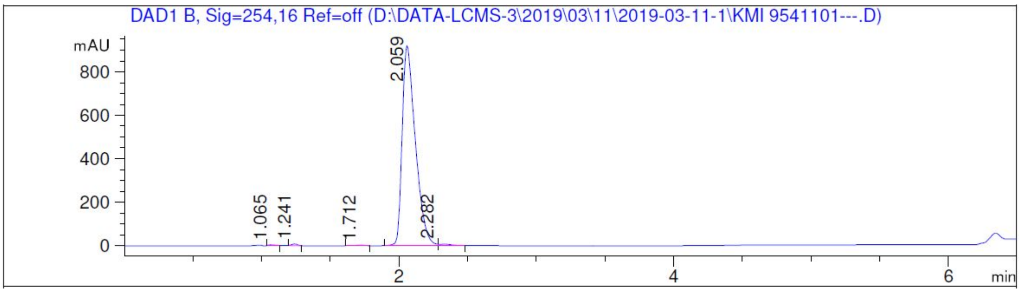

Signal 2: DAD1 B, Sig=254,16 Ref=off

#	R.T.	Type	Height	Height%	Width	Area	Area %
1	1.065	MM	3.306	0.352	0.049	9.728	0.157
2	1.241	MM	7.693	0.818	0.047	21.678	0.349
3	1.712	MM	2.065	0.220	0.080	9.939	0.160
4	2.059	MF	920.186	97.893	0.111	6122.098	98.642
5	2.282	FM	6.744	0.717	0.094	42.918	0.692

Mass spectrum

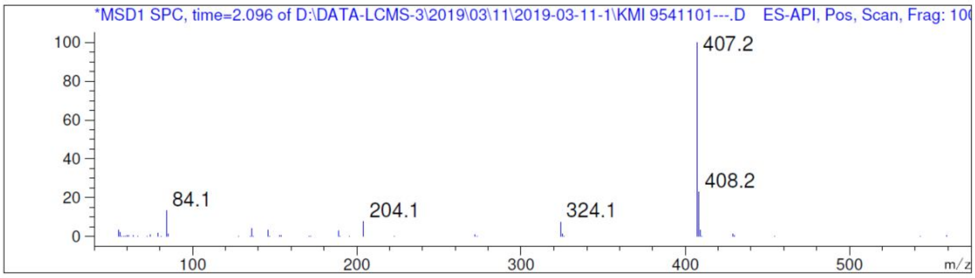

^{1}H-NMR

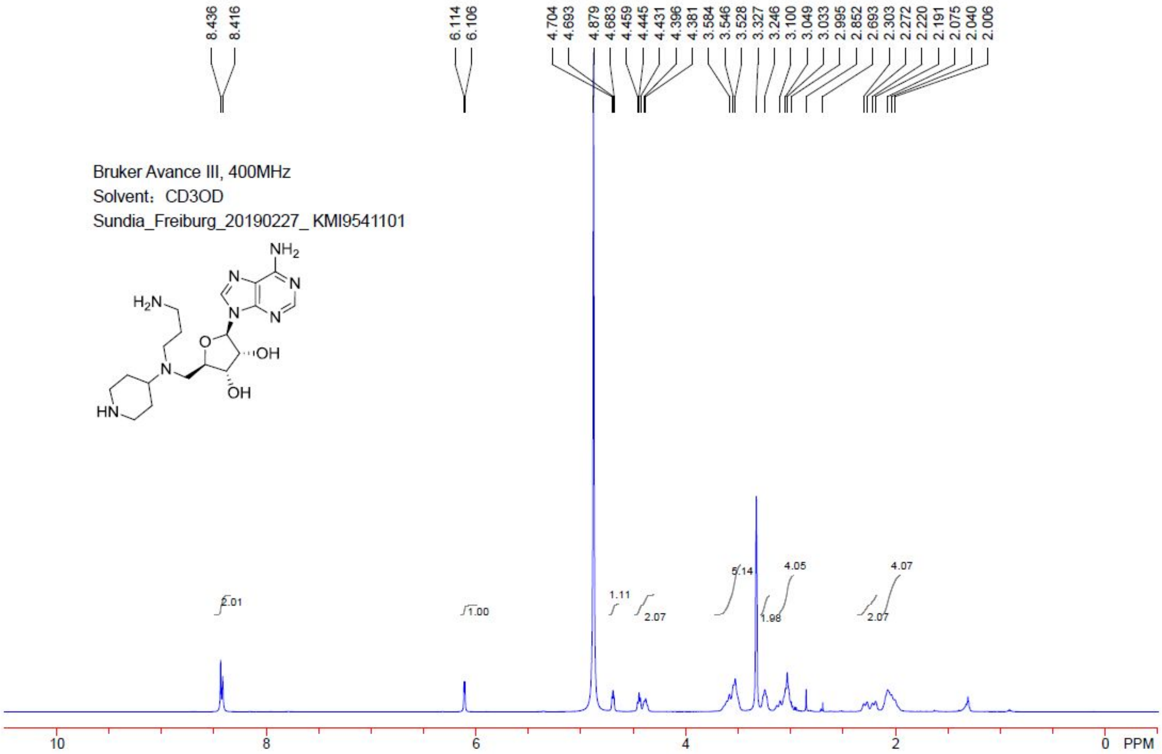

Compound **4e** = (Synthesis: **27**)

HPLC purity

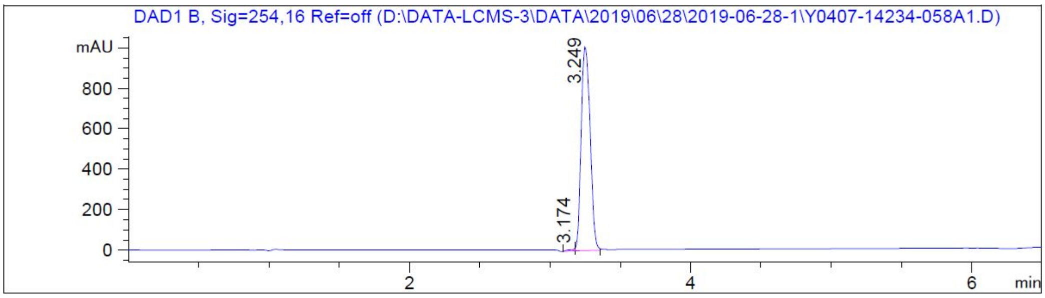

#	R.T.	Type	Height	Height%	Width	Area	Area %
1	3.174	MF	10.470	1.024	0.047	29.612	0.655
2	3.249	MF	1011.831	98.976	0.074	4493.961	99.345

Mass spectrum

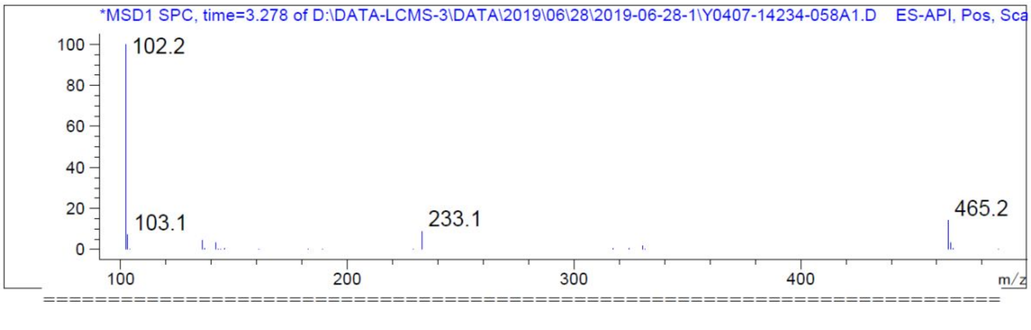

^{1}H-NMR

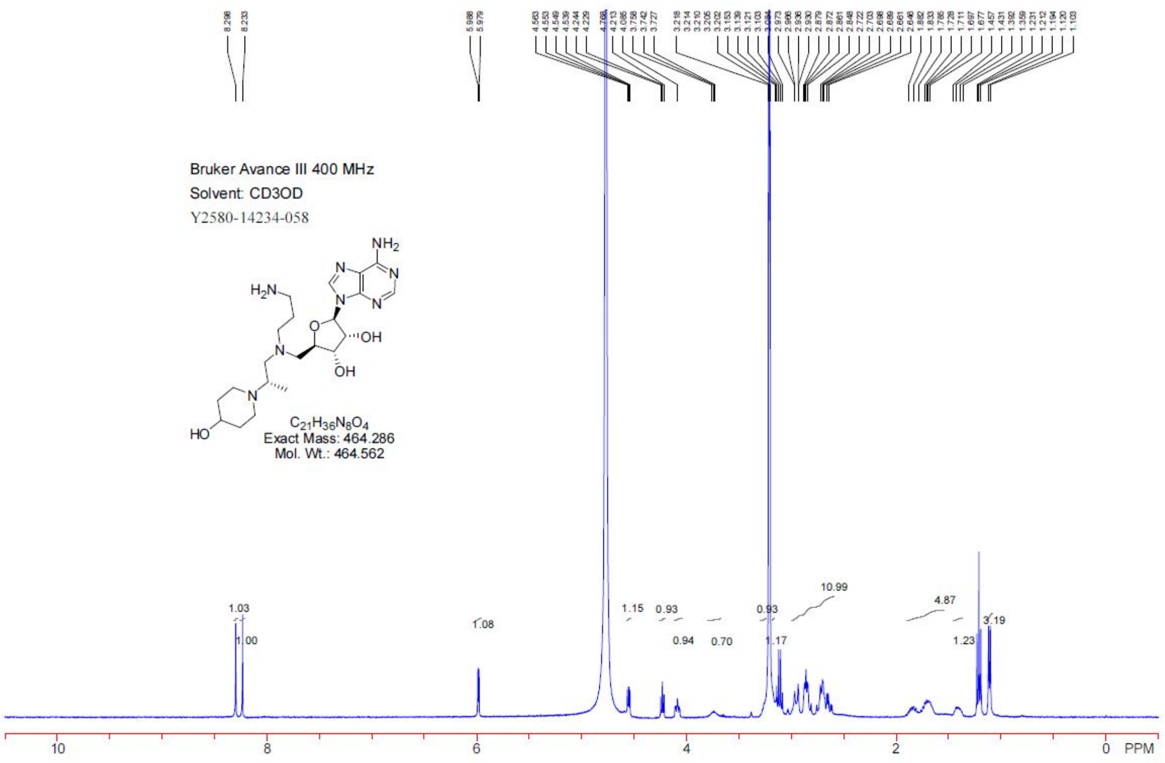

Compound **4f** = (Synthesis: **21**)

HPLC purity

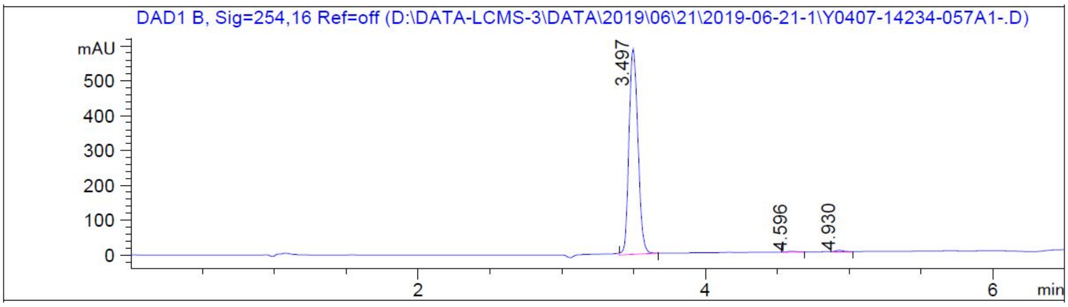

#	R.T.	Type	Height	Height%	Width	Area	Area %
1	3.497	FM	591.748	98.921	0.072	2572.451	99.124
2	4.596	FM	2.273	0.380	0.062	8.421	0.324
3	4.930	FM	4.179	0.699	0.057	14.324	0.552

Mass spectrum

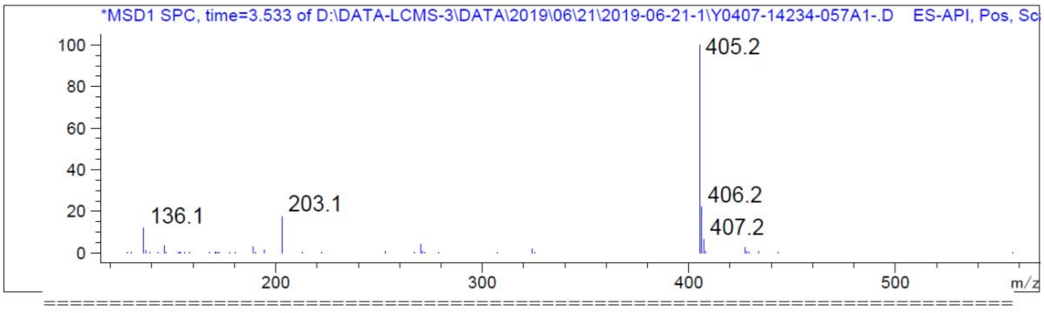

===

^{1}H-NMR

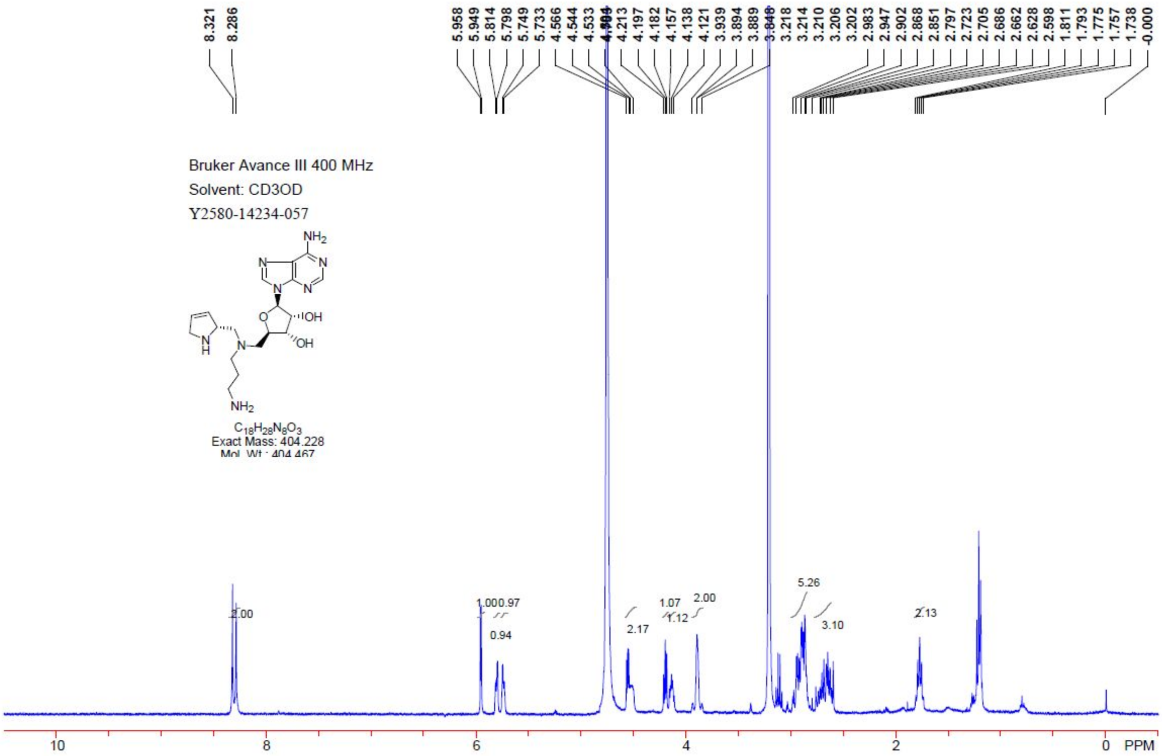

Compound **4g** = (Synthesis: **24**)

HPLC purity

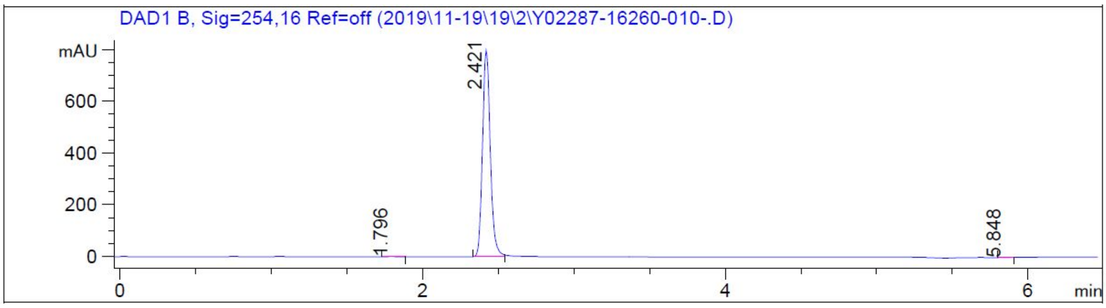

#	R.T.	Type	Height	Height%	Width	Area	Area %
1	1.796	MM	0.726	0.091	0.084	3.670	0.128
2	2.421	MF	800.067	99.770	0.060	2859.577	99.725
3	5.848	MM	1.119	0.140	0.063	4.207	0.147

Mass spectrum

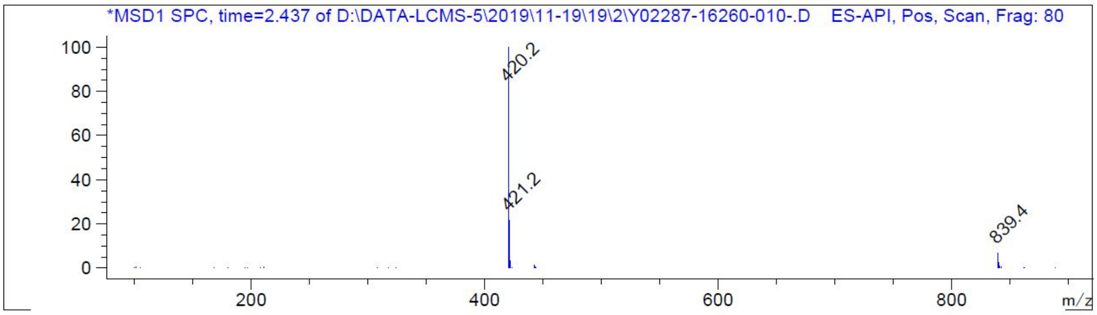

1H-NMR

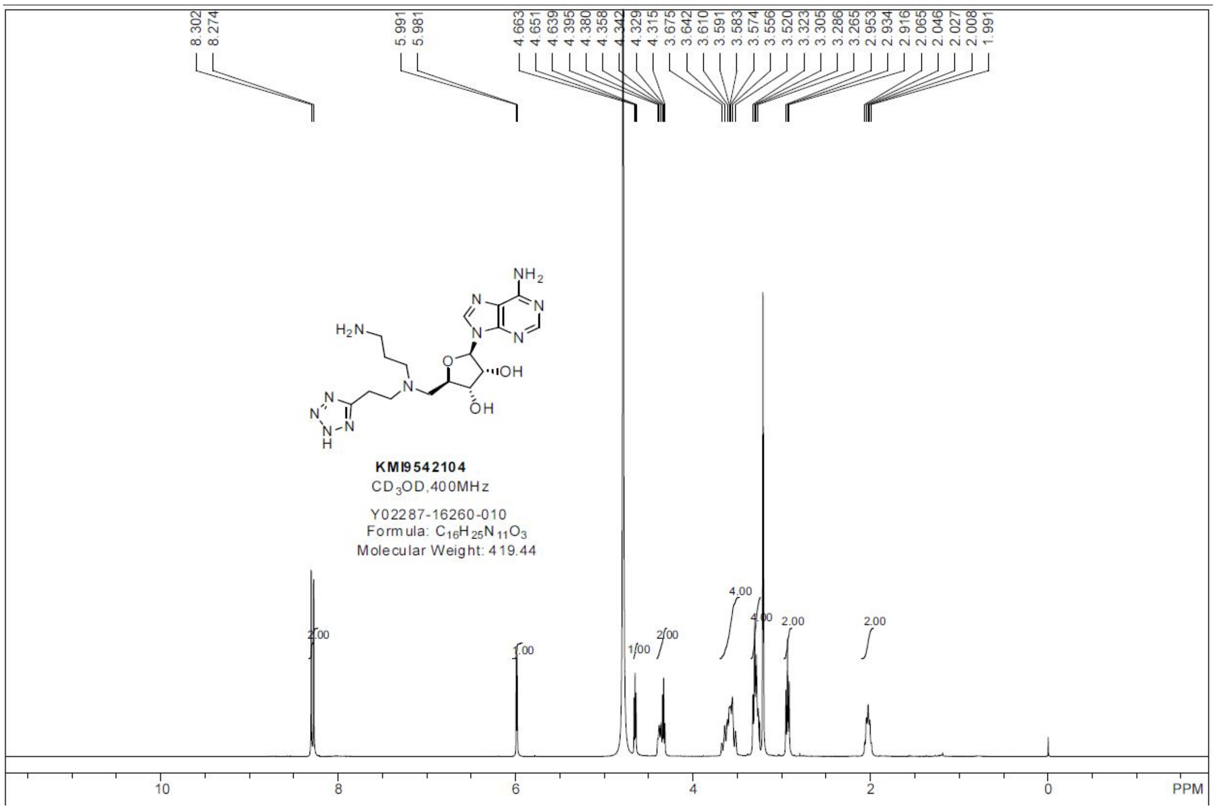

Compound **5a** = (Synthesis: **9g**)

HPLC purity

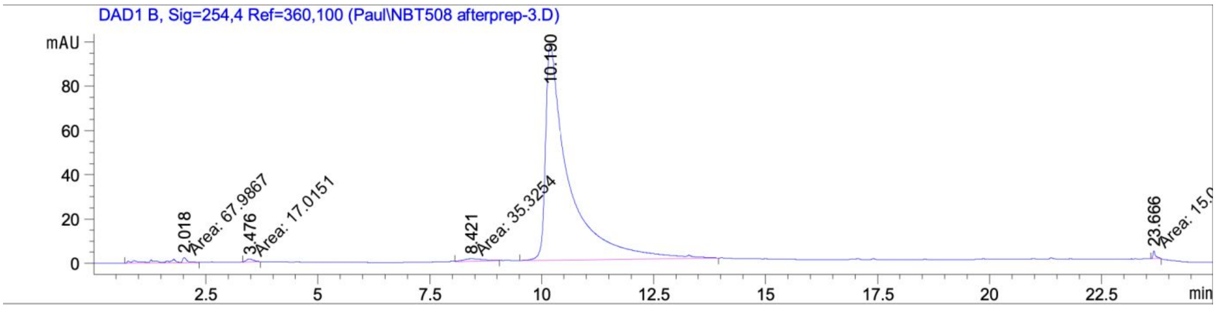

Signal 2: DAD1 B, Sig=254,4 Ref=360,100

Peak #	RetTime [min]	Type	Width [min]	Area [mAU*s]	Height [mAU]	Area %
1	2.018	MM	0.4852	67.98672	2.33546	1.8480
2	3.476	MM	0.1965	17.01510	1.44297	0.4625
3	8.421	MM	0.5266	35.32535	1.11805	0.9602
4	10.190	BV R	0.4860	3543.55322	97.49412	96.3198
5	23.666	MP	0.0693	15.06627	3.62598	0.4095

Totals : 3678.94667 106.01658

MS

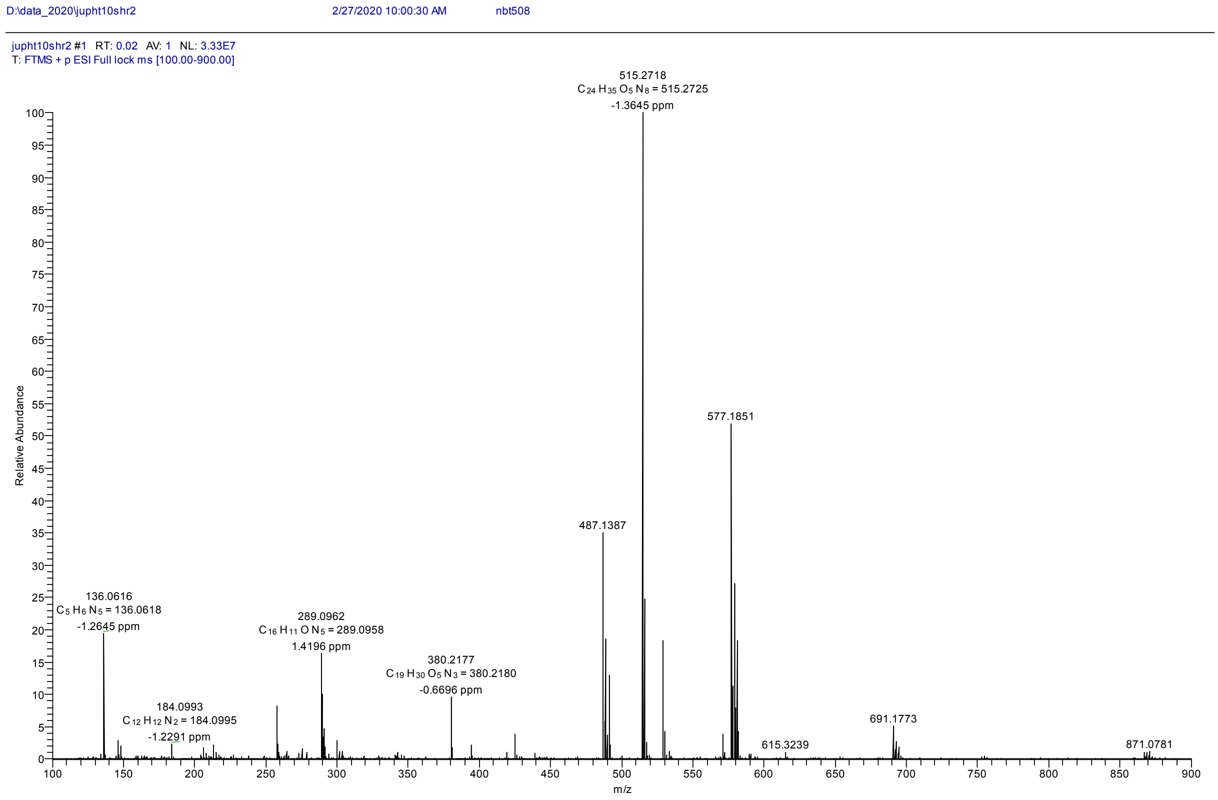

^{1}H-NMR

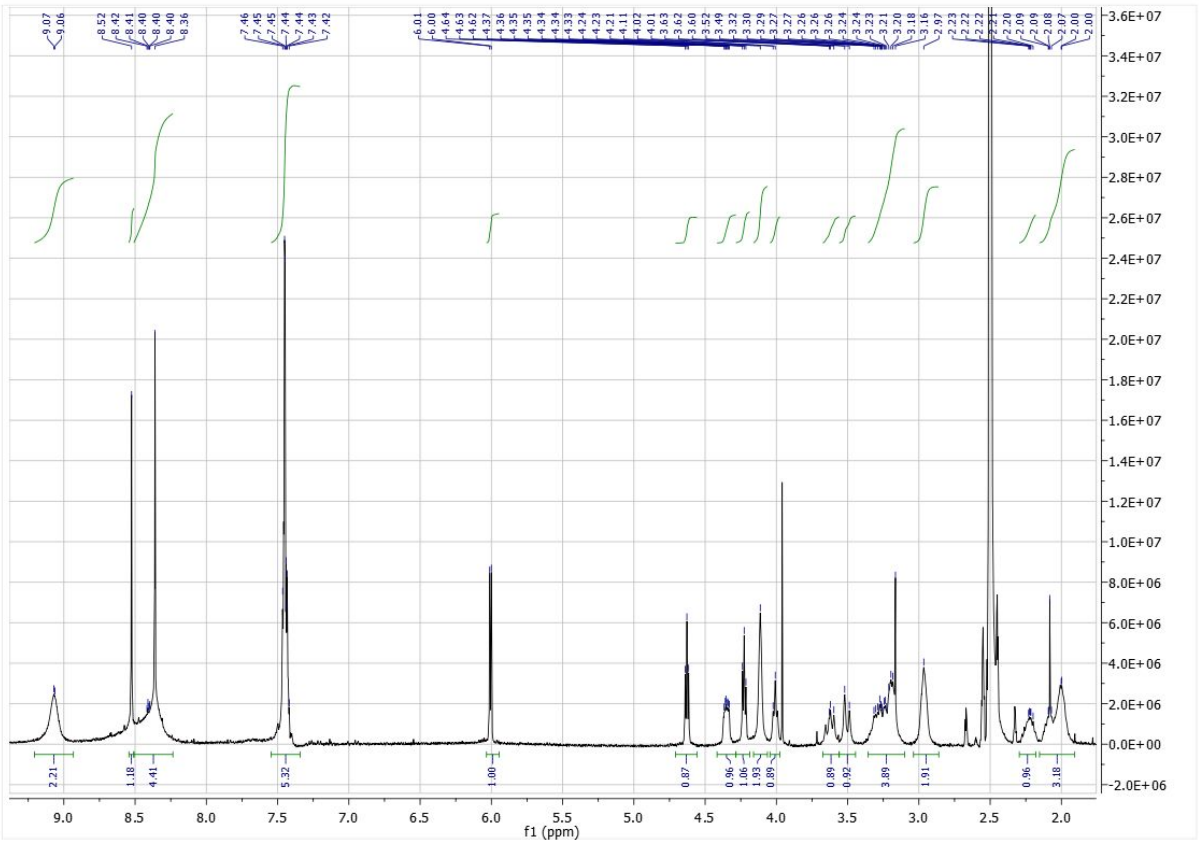

Compound **5b** = (Synthesis: **9h**)

HPLC purity

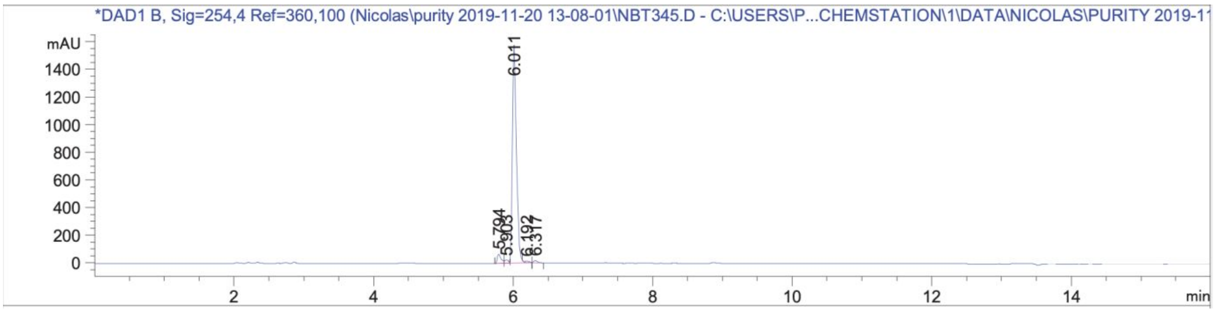


```
Signal 2: DAD1 B, Sig=254,4 Ref=360,100
 Signal has been modified after loading from rawdata file!

Peak RetTime Type  Width     Area      Height     Area
  #   [min]        [min]   [mAU*s]     [mAU]       %
----|-------|----|-------|----------|----------|--------|
   1   5.794 BV E  0.0562  255.39357   67.44485   3.6687
   2   5.903 VV E  0.0566   91.58904   23.96026   1.3157
   3   6.011 VV R  0.0641 6511.84033 1580.57532  93.5426
   4   6.192 VB E  0.0655   44.46634   10.93099   0.6388
   5   6.317 BB    0.0508   58.07191   17.44784   0.8342

Totals :                  6961.36119 1700.35926
```

Mass spectrum

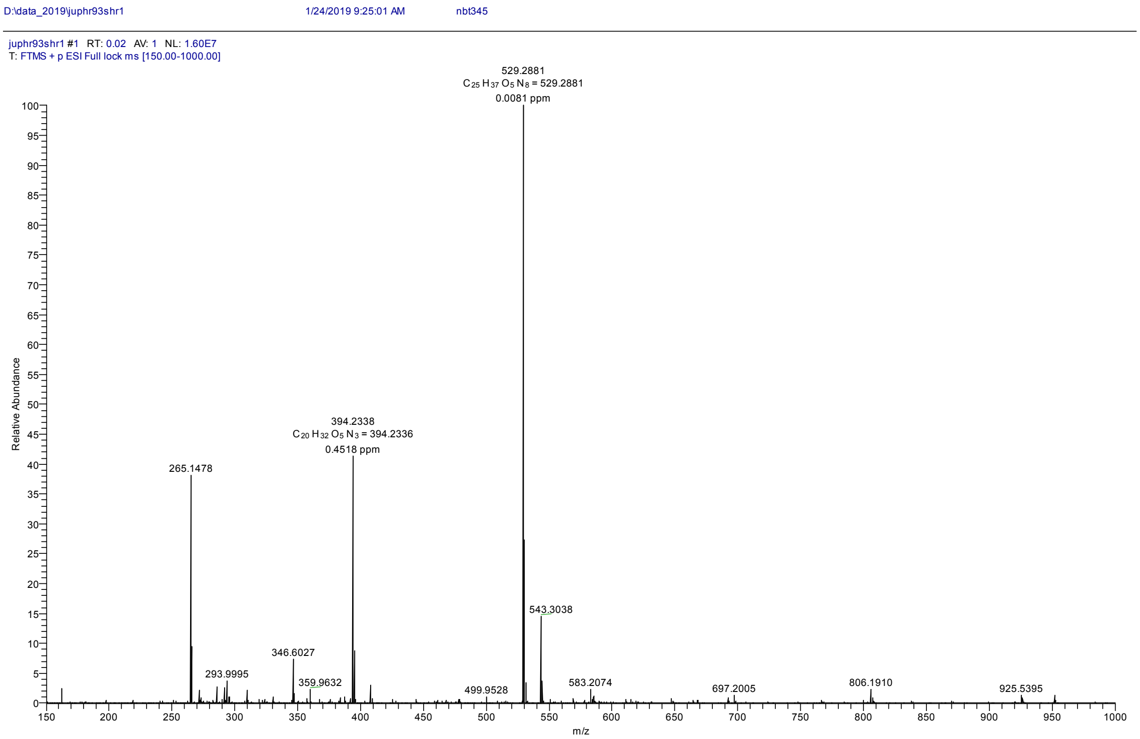

D:\data_2019\juphr93shr1
1/24/2019 9:25:01 AM
nbt345
juphr93shr1 #1 RT: 0.02 AV: 1 NL: 1.60E7
T: FTMS + p ESI Full lock ms [150.00-1000.00]
529.2881
C25 H37 O5 N8 = 529.2881
0.0081 ppm
394.2338
C20 H32 O5 N3 = 394.2336
0.4518 ppm
265.1478
346.6027
293.9995
359.9632
499.9528
543.3038
583.2074
697.2005
806.1910
925.5395
Relative Abundance
m/z

^{1}H-NMR

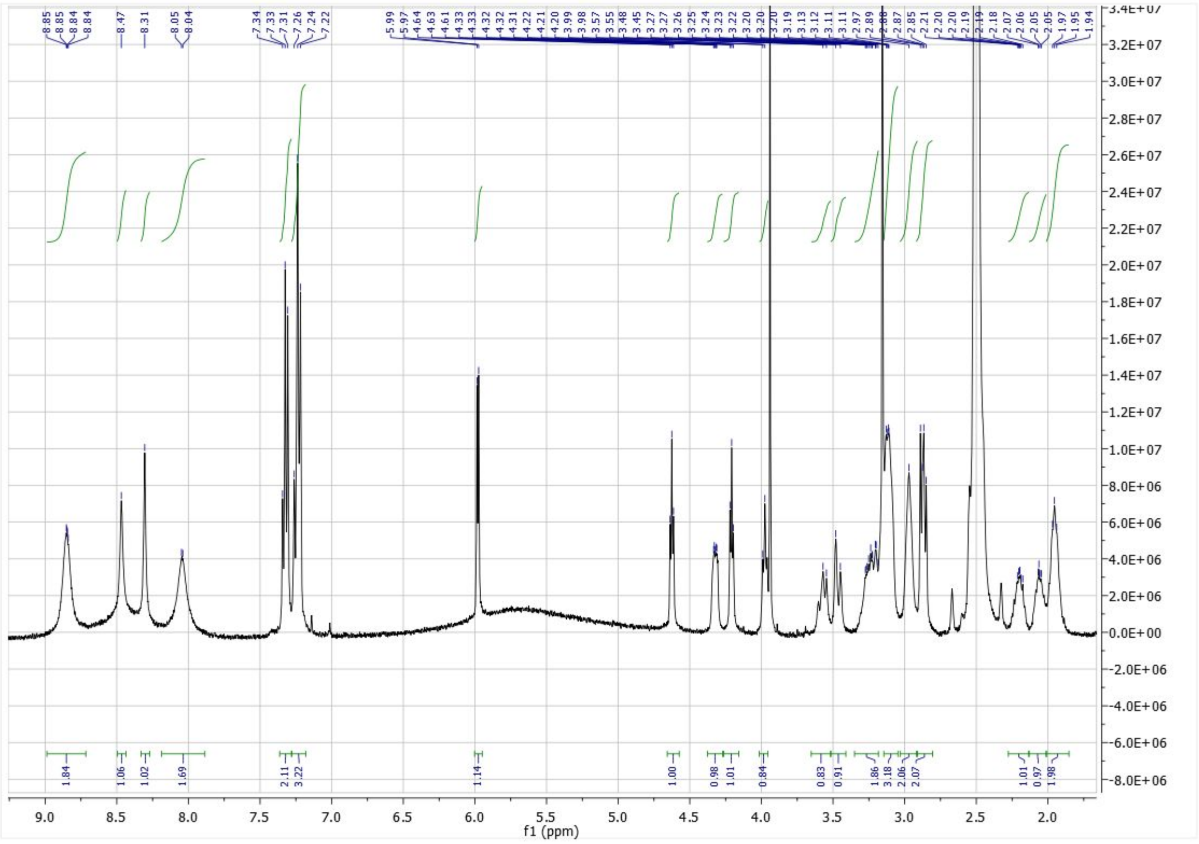

f1 (ppm)

Compound **5c** = (Synthesis: **9i**)

HPLC purity

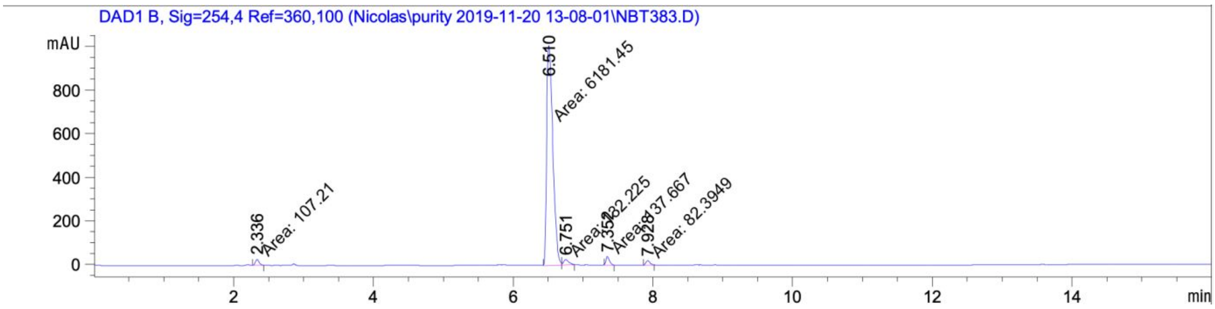

Signal 2: DAD1 B, Sig=254,4 Ref=360,100

Peak #	RetTime [min]	Type	Width [min]	Area [mAU*s]	Height [mAU]	Area %
1	2.336	MM	0.0634	107.20972	28.16707	1.6144
2	6.510	MM	0.1018	6181.44678	1011.98035	93.0808
3	6.751	MM	0.0854	132.22493	25.80116	1.9911
4	7.352	MM	0.0578	137.66734	39.71314	2.0730
5	7.928	MM	0.0674	82.39493	20.36635	1.2407
Totals :				6640.94370	1126.02807	

Mass spectrum

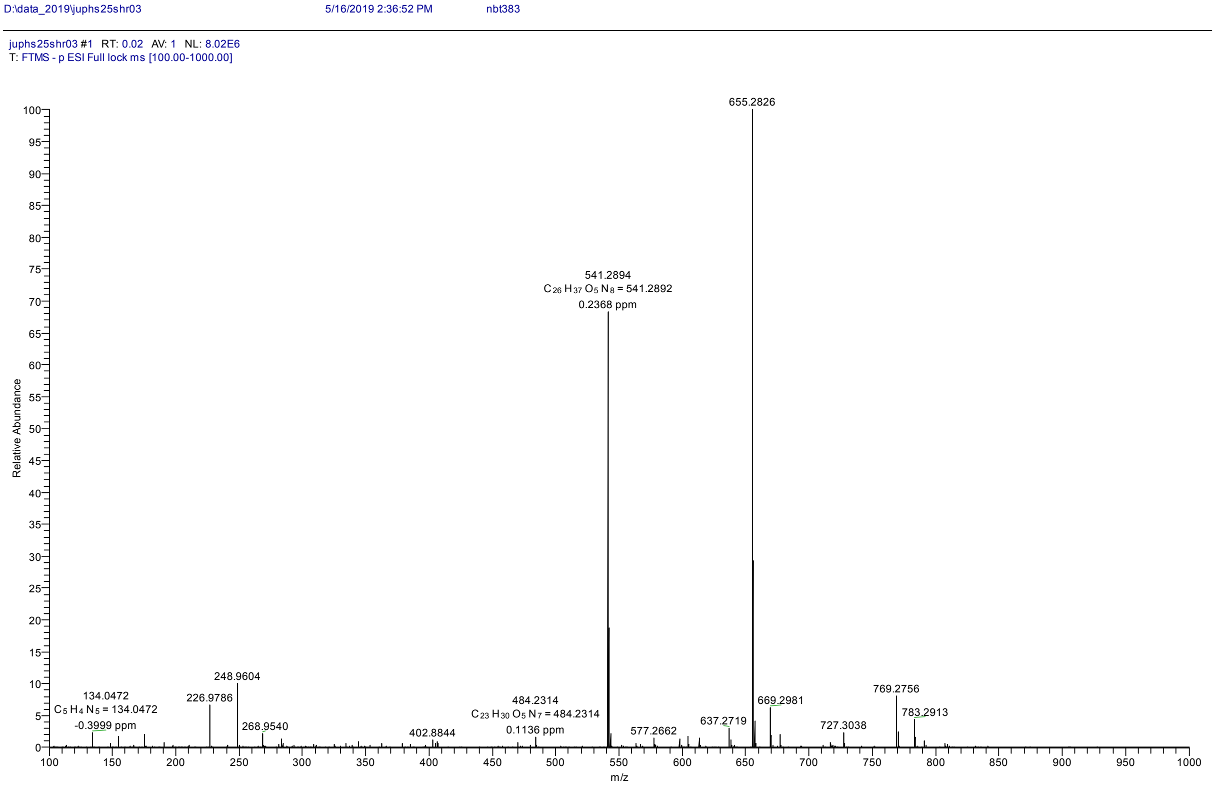

^{1}H-NMR

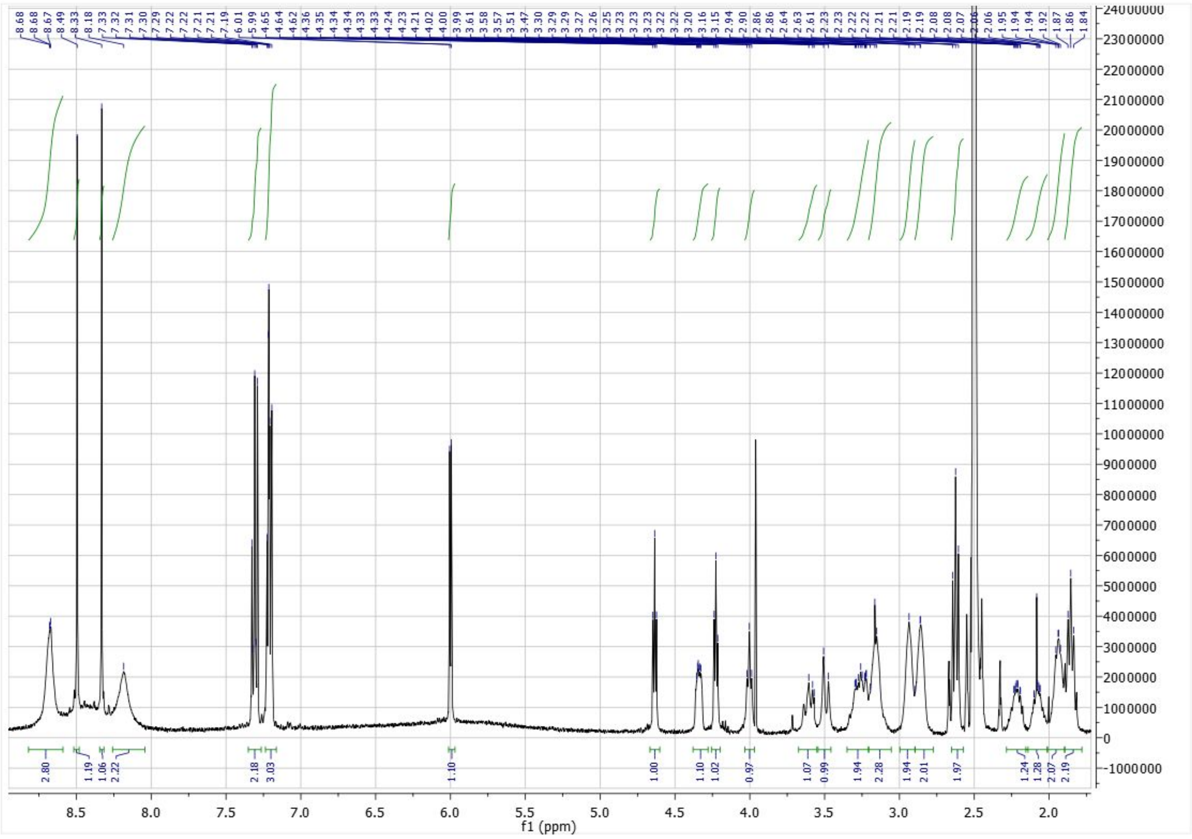

Compound **6** = (Synthesis: **50a**)

HPLC purity

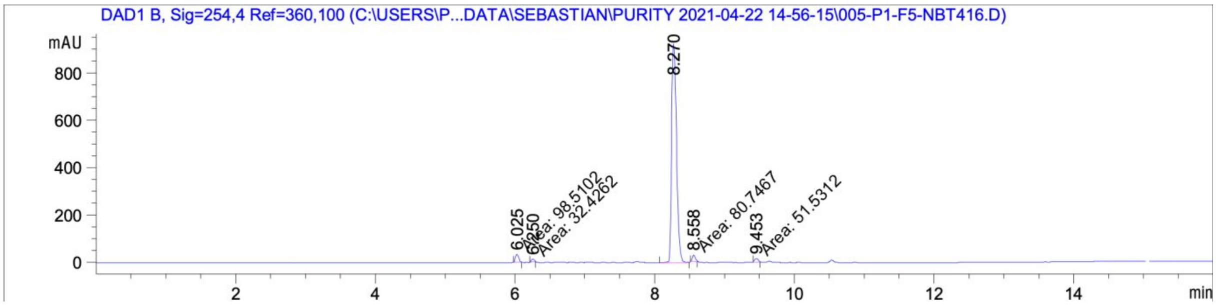

Signal 2: DAD1 B, Sig=254,4 Ref=360,100

Peak #	RetTime [min]	Type	Width [min]	Area [mAU*s]	Height [mAU]	Area %
1	6.025	MM	0.0512	98.51015	32.08501	2.0708
2	6.250	MM	0.0433	32.42623	12.47170	0.6816
3	8.270	BV	0.0769	4493.87256	921.90521	94.4669
4	8.558	MM	0.0502	80.74670	26.81119	1.6974
5	9.453	MM	0.0553	51.53121	15.54275	1.0833
Totals :				4757.08686	1008.81587	

Mass spectrum

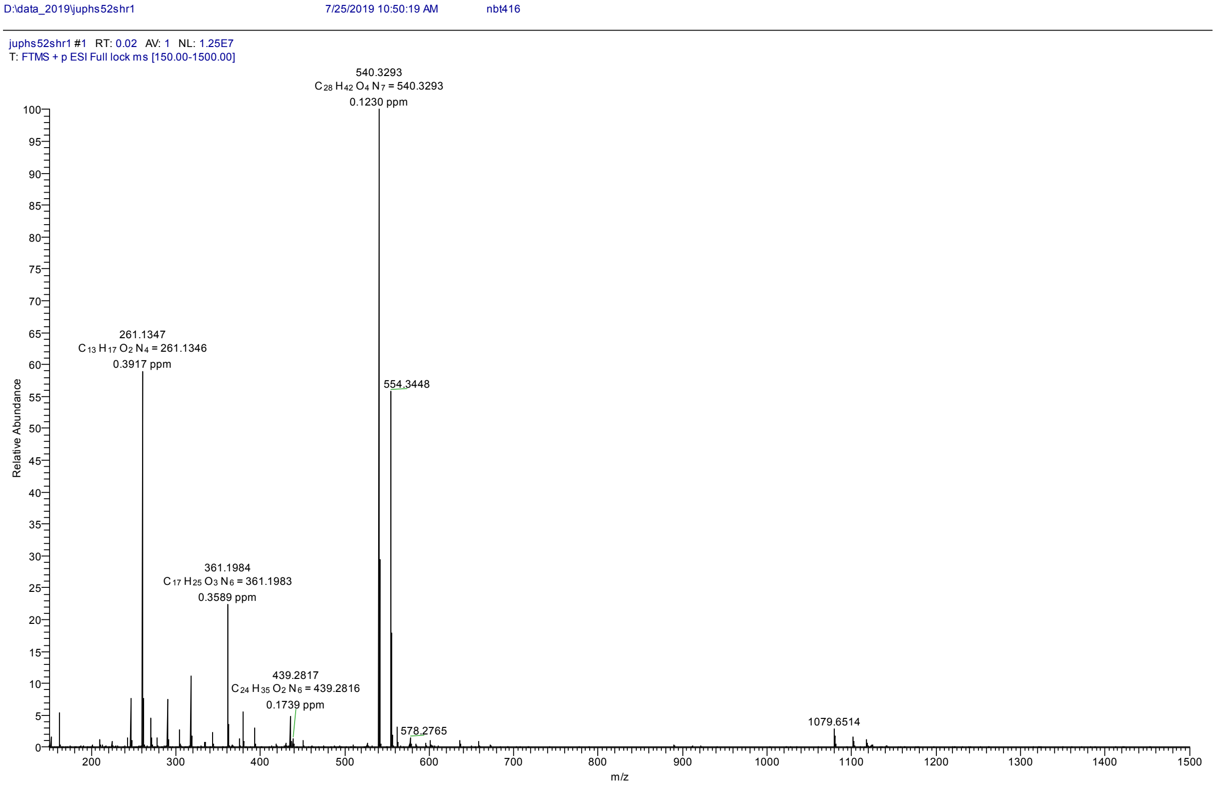
D:\data_2019\juphs52shr1
7/25/2019 10:50:19 AM
nbt416
juphs52shr1 #1 RT: 0.02 AV: 1 NL: 1.25E7
T: FTMS + p ESI Full lock ms [150.00-1500.00]
540.3293
C28 H42 O4 N7 = 540.3293
0.1230 ppm
261.1347
C13 H17 O2 N4 = 261.1346
0.3917 ppm
554.3448
361.1984
C17 H25 O3 N6 = 361.1983
0.3589 ppm
439.2817
C24 H35 O2 N6 = 439.2816
0.1739 ppm
578.2765
1079.6514
Relative Abundance
m/z

^{1}H-NMR

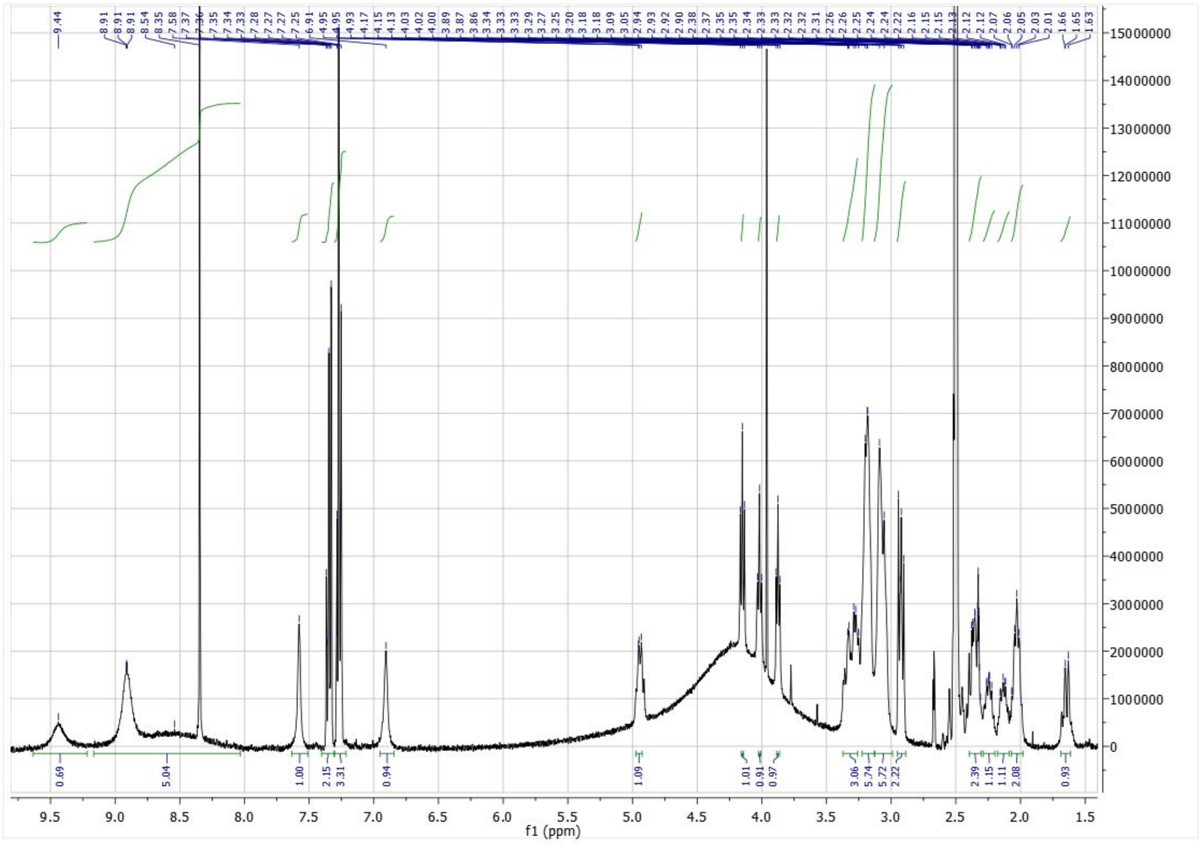
f1 (ppm)

Compound **7a** = (Synthesis: **50b**)

HPLC purity

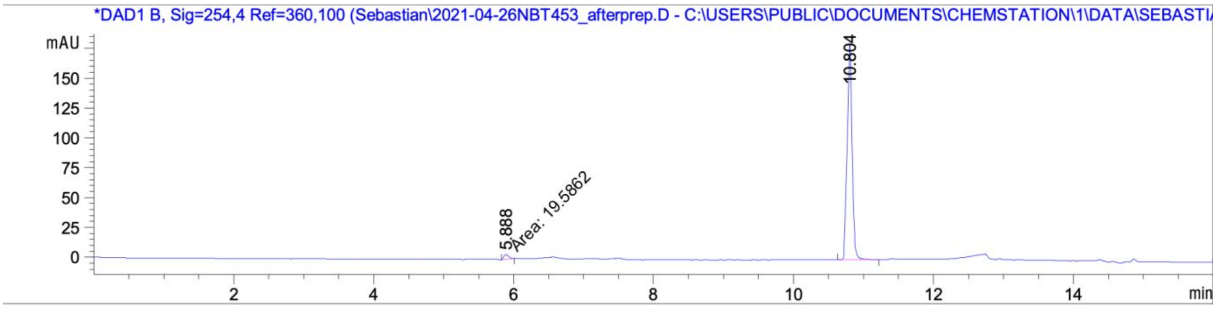


```
Signal 2: DAD1 B, Sig=254,4 Ref=360,100
  Signal has been modified after loading from rawdata file!

Peak RetTime Type  Width     Area      Height     Area
  #   [min]        [min]   [mAU*s]     [mAU]       %
----|-------|----|-------|----------|----------|--------|
   1   5.888 MM    0.0811   19.58621    4.02676   2.1605
   2  10.804 BB    0.0713  886.98206  180.56445  97.8395

Totals :                   906.56826  184.59122
```

Mass spectrum

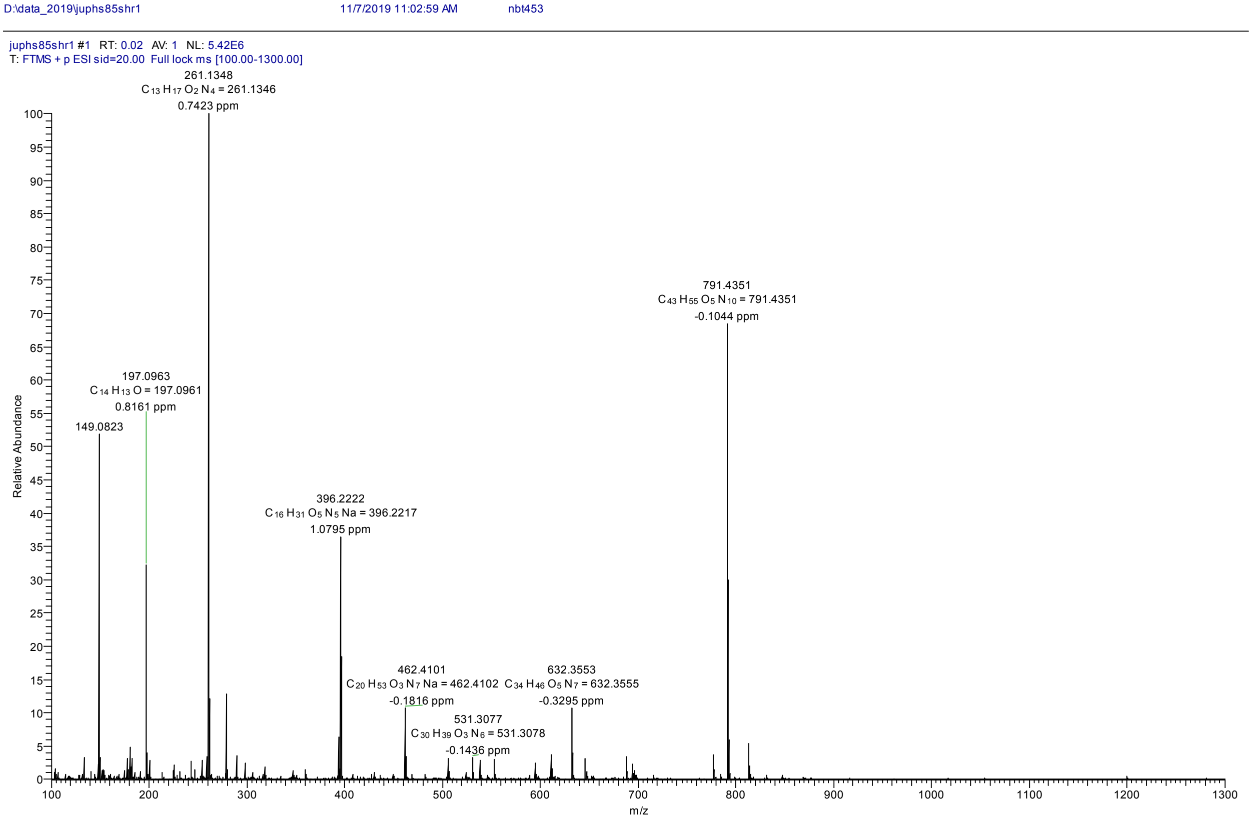

^{1}H-NMR

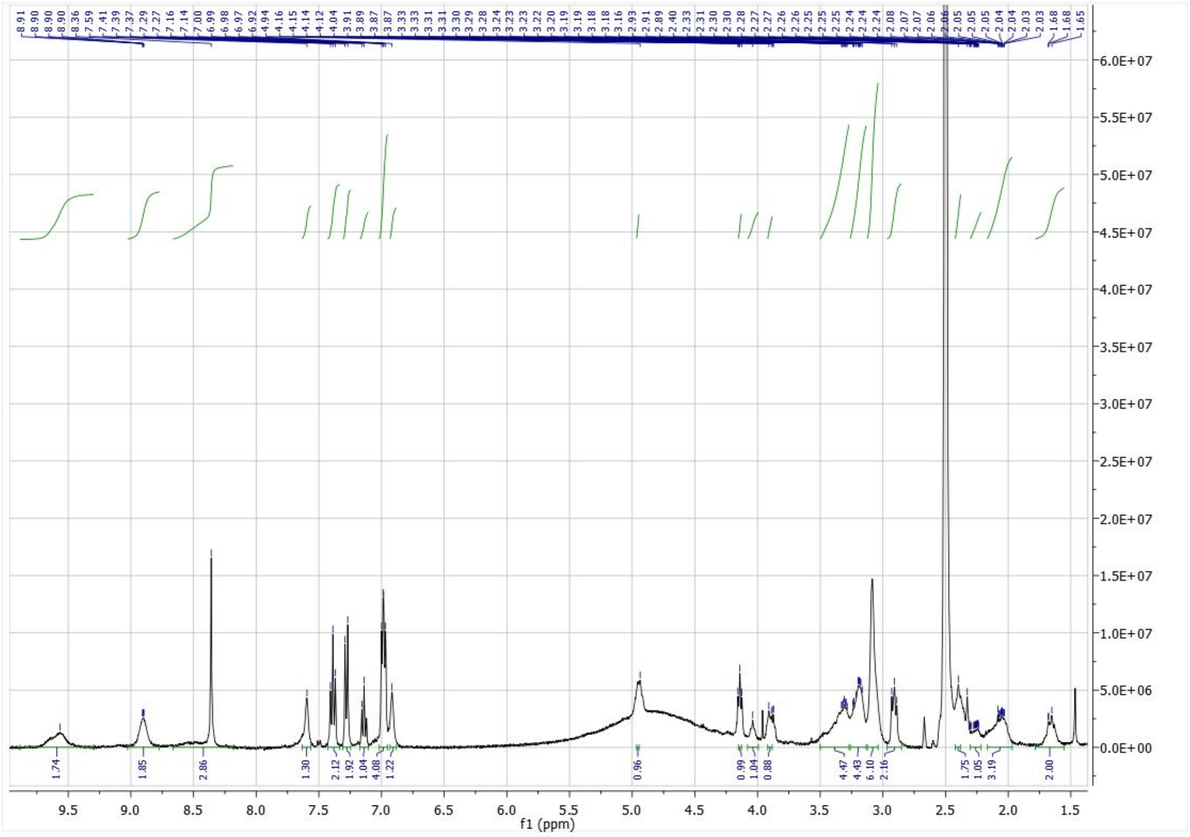

Compound **7b**

HPLC purity

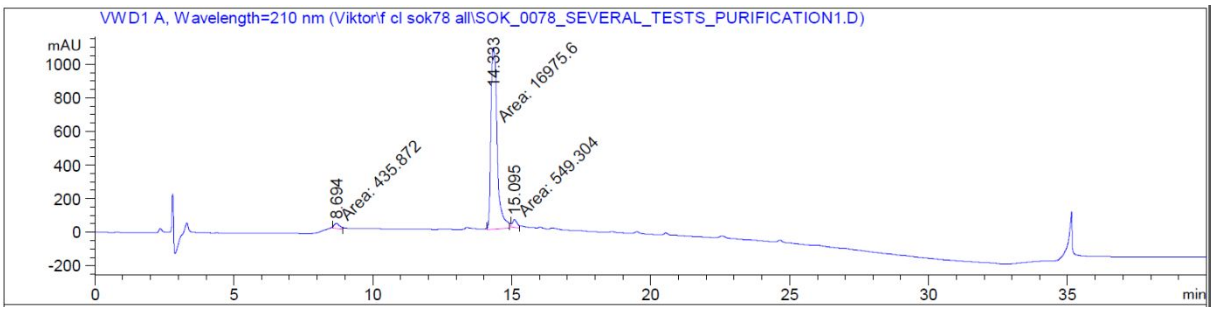

Signal 1: VWD1 A, Wavelength=210 nm

Peak #	RetTime [min]	Type	Width [min]	Area [mAU*s]	Height [mAU]	Area %
1	8.694	MM	0.2364	435.87225	30.73635	2.4268
2	14.333	MM	0.2613	1.69756e4	1082.61292	94.5149
3	15.095	MM	0.1870	549.30365	48.95205	3.0583
Totals :				1.79608e4	1162.30131	

The peak at 2.5 min and the peak at 35 min result from the solvent methanol.

^{1}H-NMR

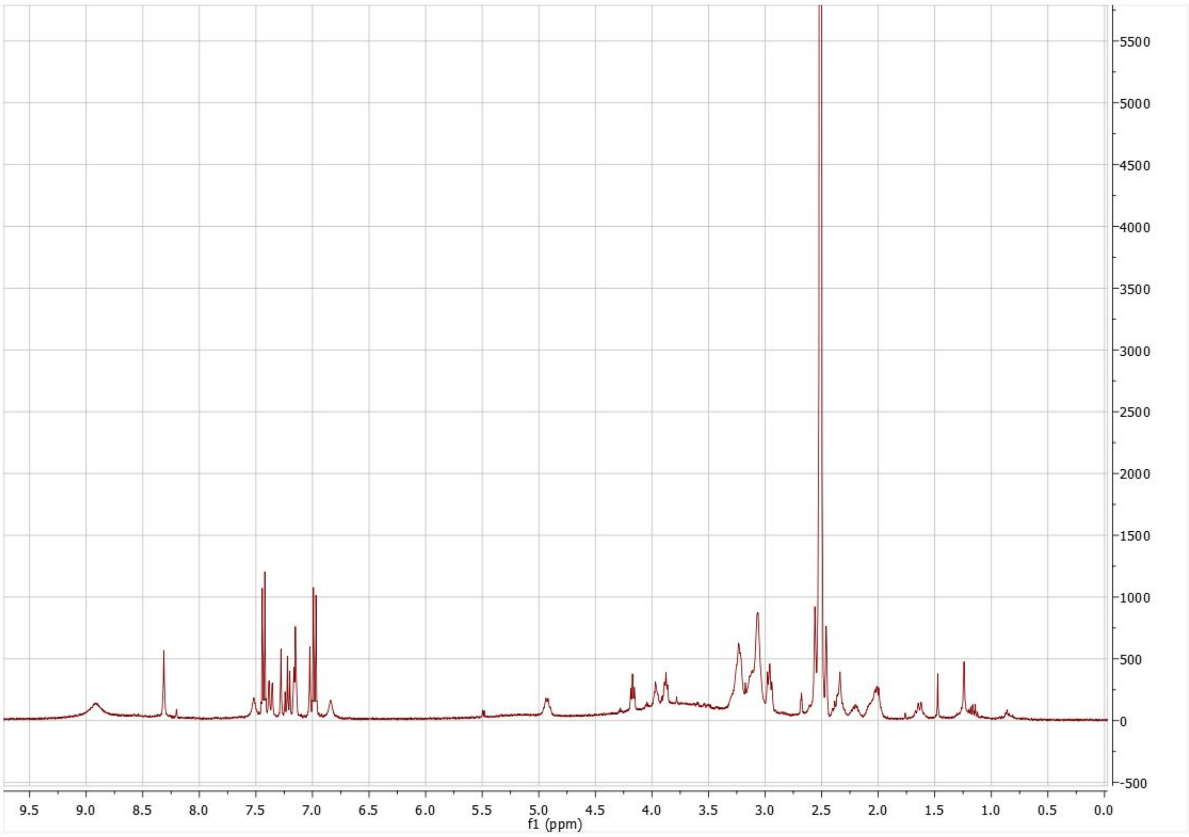

Compound **7b-N** = (Synthesis: **54b**)

HPLC purity

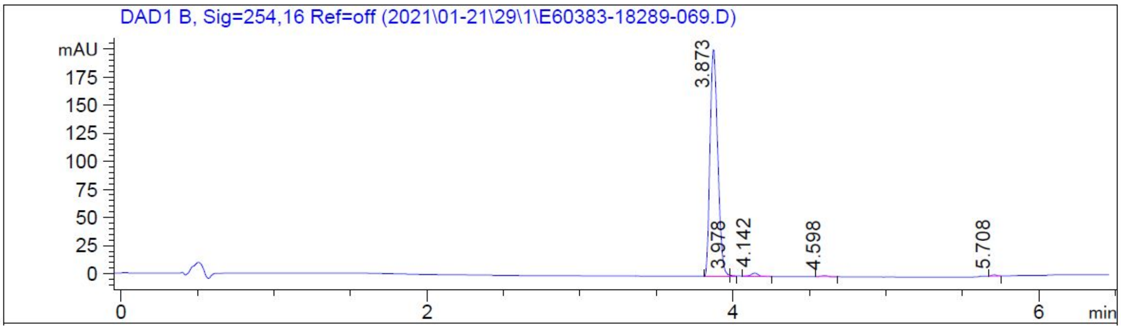

Signal 2: DAD1 B, Sig=254,16 Ref=off

#	R.T.	Type	Height	Height%	Width	Area	Area %
1	3.873	MF	204.433	97.099	0.058	707.483	97.510
2	3.978	FM	1.051	0.499	0.028	1.764	0.243
3	4.142	BB	2.981	1.416	0.052	10.323	1.423
4	4.598	BB	0.888	0.422	0.051	2.956	0.407
5	5.708	MM	1.189	0.565	0.042	3.024	0.417

Mass spectrum

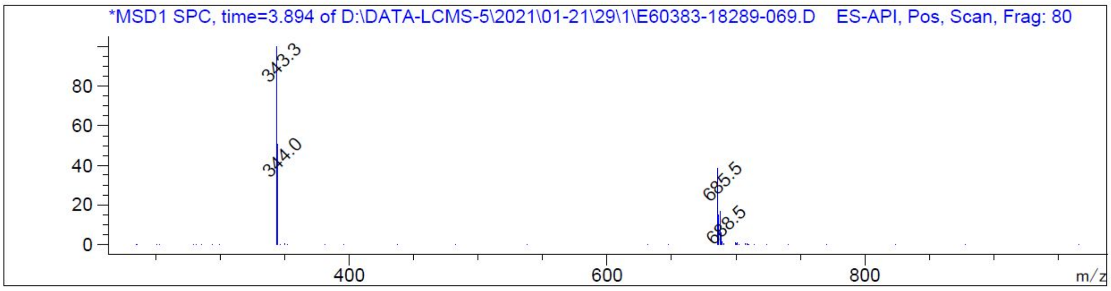

^{1}H-NMR

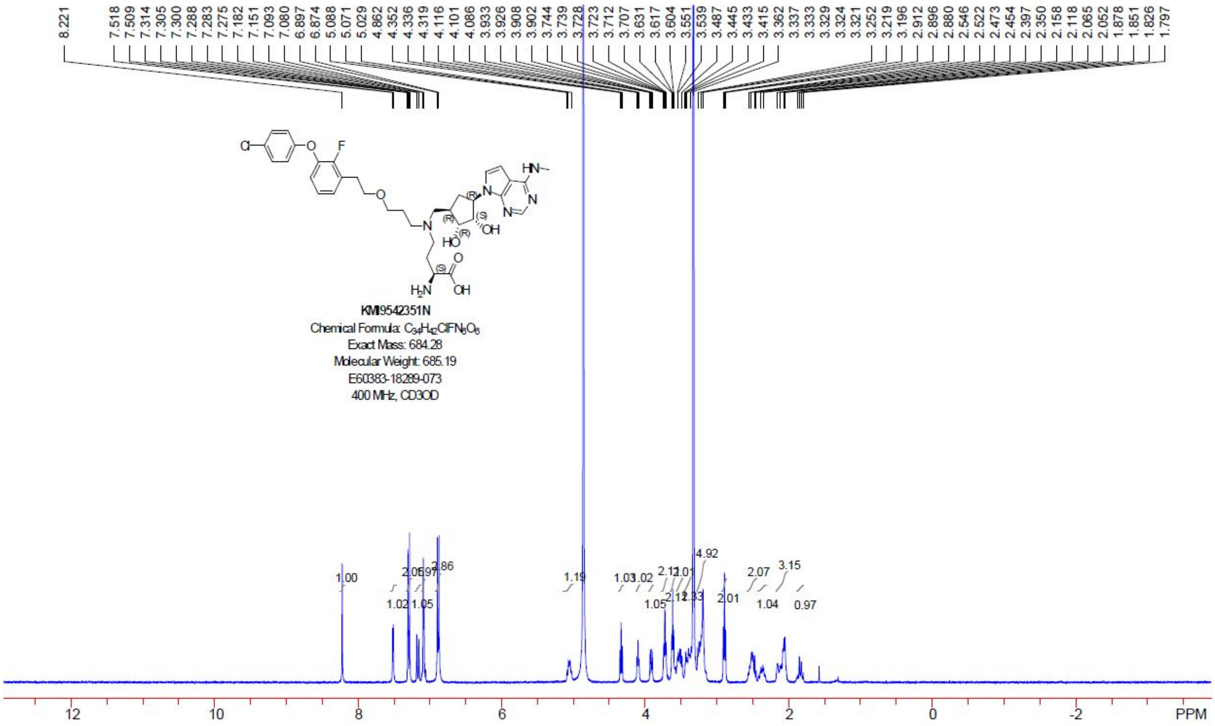

Compound **8** = (Synthesis: **54a**)

HPLC purity (UV-Vis; 254 nm). 100 %.

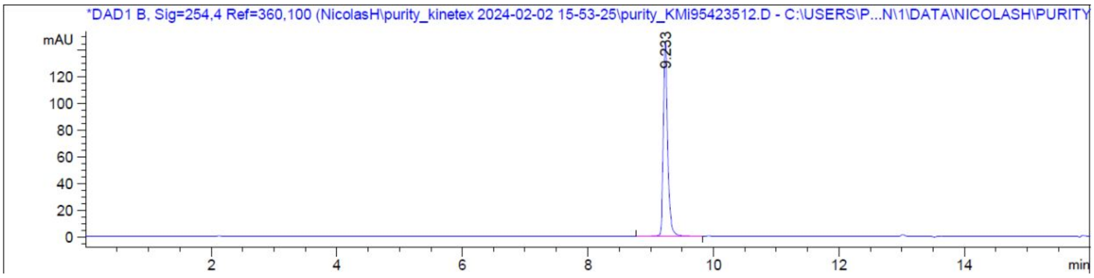


```
Signal 2: DAD1 B, Sig=254,4 Ref=360,100
 Signal has been modified after loading from rawdata file!

Peak RetTime Type  Width     Area      Height     Area
  #   [min]        [min]   [mAU*s]     [mAU]       %
----|-------|----|-------|----------|----------|--------|
   1   9.233 BB    0.0744  709.10858  146.55687 100.0000

Totals :                   709.10858  146.55687
```

Mass spectrum

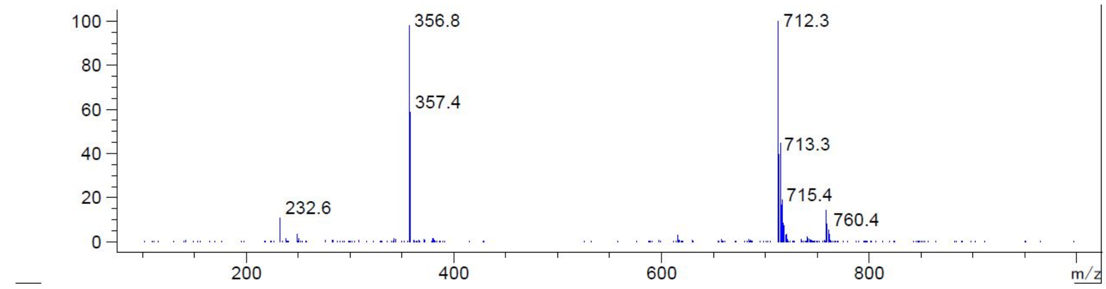

^{1}H-NMR

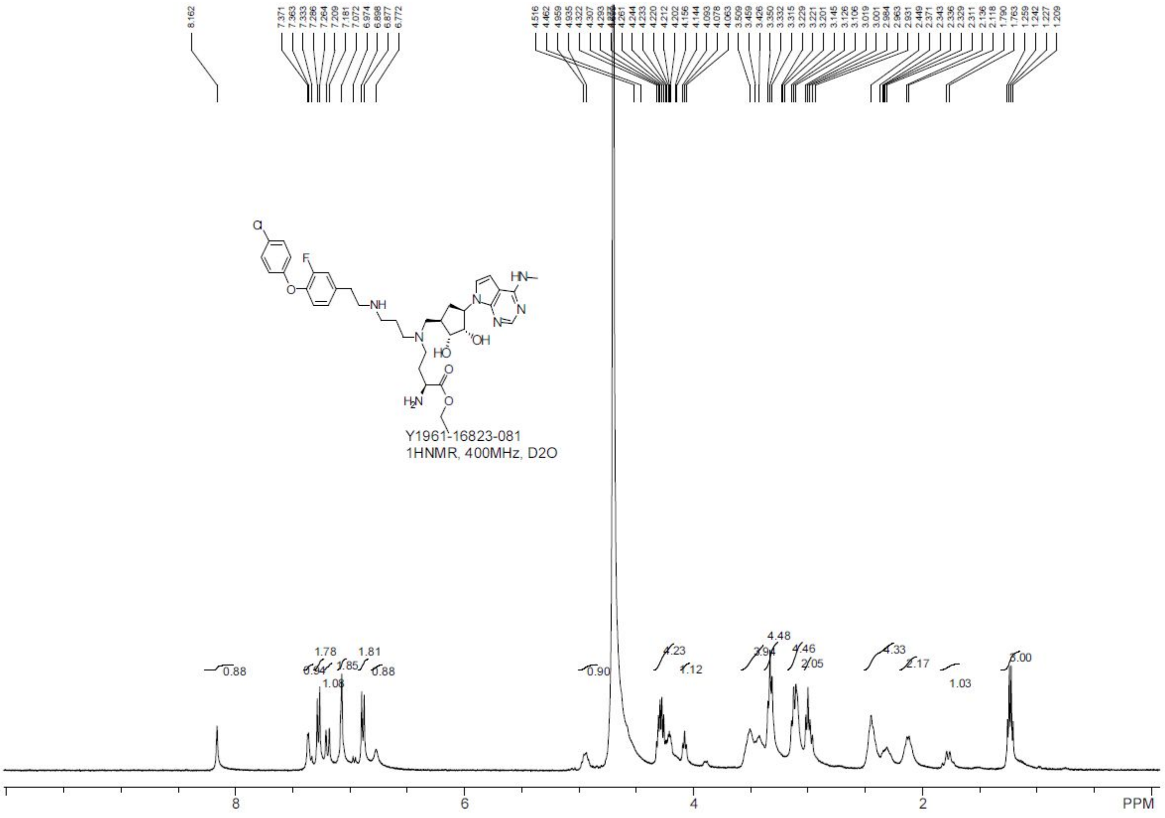

Compound **8-N** = (Synthesis: **54c**)

HPLC purity

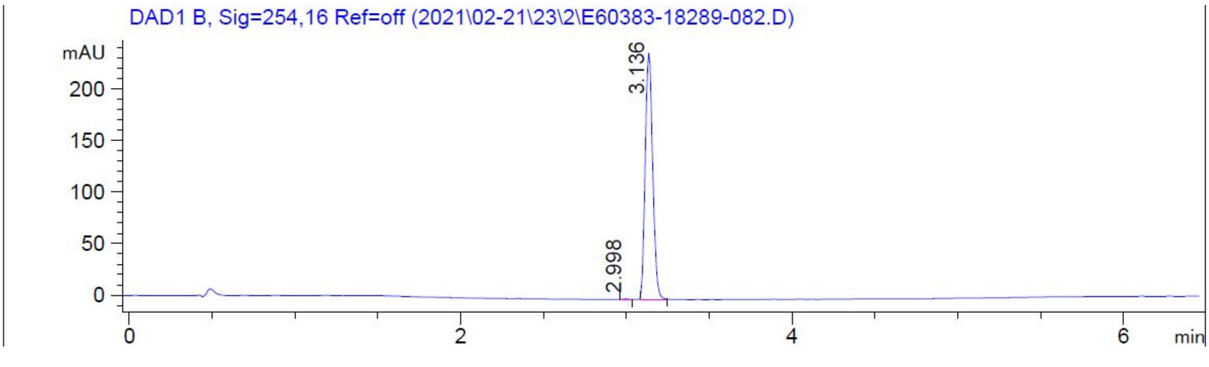

Signal 2: DAD1 B, Sig=254,16 Ref=off

#	R.T.	Type	Height	Height%	Width	Area	Area %
1	2.998	MM	0.629	0.262	0.041	1.553	0.207
2	3.136	BB	238.892	99.738	0.049	748.436	99.793

Mass spectrum

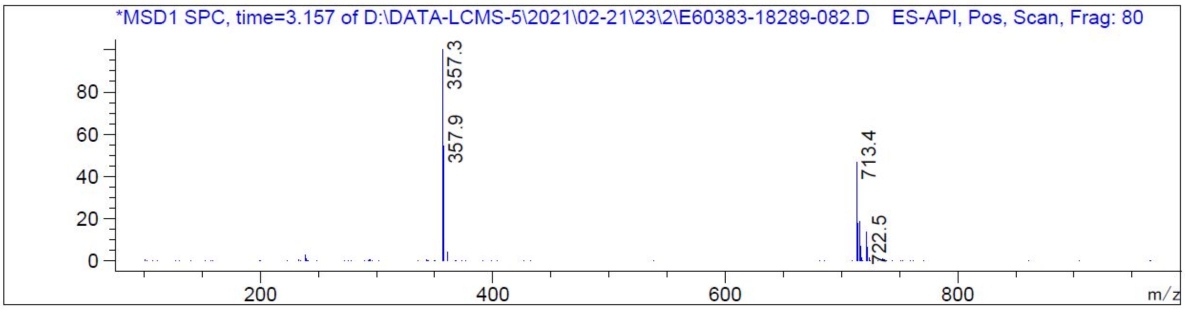

^{1}H-NMR

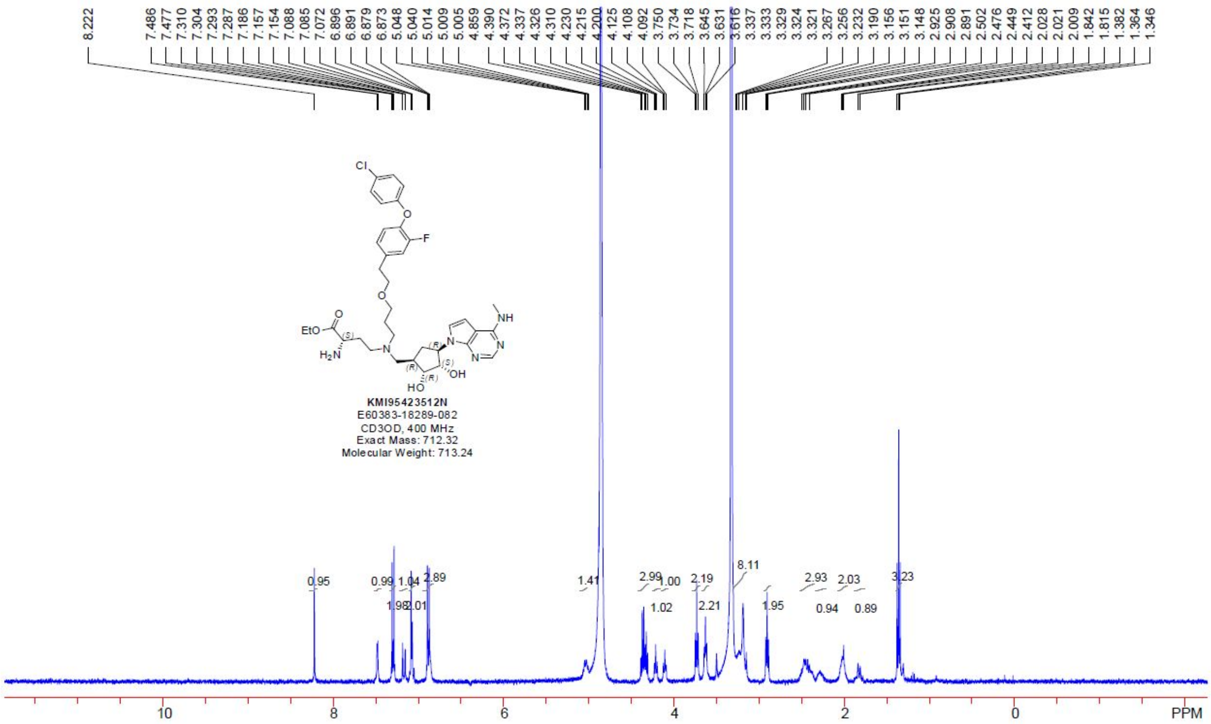

Table S1: Data collection and Refinement Statistics

	**KMT9+compound 1**	**KMT9+SAH**	**KMT9+compound 2a**
**Data collection**			
Space group	$P6_122$	$P6_122$	$P6_122$
Cell dimensions			
a, *b*, *c* (Å)	110.0, 110.0, 129.7	110.0 110.0 132.2	110.1, 110.1, 129.8
α, β, γ (°)	90.0, 90.0, 120.0	90.0, 90.0, 120.0	90.0, 90.0, 120.0
Resolution (Å)	47.64 - 1.60 (1.63 - 1.60) *	47.65 - 1.60 (1.63 - 1.60) *	47.68 – 1.59 (1.62 – 1.59) *
Mean I/sigma (I)	29.8 (2.4)	19.1 (2.2)	21.3 (1.3)
R_{merge}	0.076 (2.119)	0.122 (2.020)	0.180 (3.709)
$CC_{1/2}$	1.000 (0.765)	0.999 (0.752)	1.000 (0.543)
Completeness (%)	100.0 (100.0)	100.0 (100.0)	99.1 (97.6)
Redundancy	39.0 (39.2)	39.1 (39.1)	39.7 (38.8)
**Refinement**			
Resolution (Å)	47.64 - 1.60 (1.63 - 1.60) *	47.65 - 1.6 (1.66 - 1.60) *	47.68 – 1.59 (1.62 – 1.59) *
No. reflections	61469	62627	61819
R_{work} / R_{free}	0.157/ 0.187	0.183/0.202	0.153/0.191
No. atoms			
Protein	2456	2462	2439
Ligand	33	26	36
Solvent	117	122	169
Average *B*-factor			
Overall	32.00	25.14	27.66
Protein	32.71	24.90	27.26
Solvent	34.42	30.16	34.79
Ligand	25.38	17.18	21.45
R.m.s. deviations			
Bond lengths (Å)	0.010	0.014	0.013
Bond angles (º)	1.54	1.87	1.75
**Ramachandran Plot**			
Favored	98.40	97.76	97.10
Allowed	1.28	1.92	2.58
Not Allowed	0.32	0.32	0.32

*Single crystal was used for each dataset; *Values in parentheses are highest-resolution shell

Table S2: Data collection and Refinement Statistics

	**KMT9+compound 2b**	**KMT9+compound 2c**	**KMT9+compound 3a**
**Data collection**			
Space group	$P6_122$	$P6_122$	$P6_122$
Cell dimensions			
a, *b*, *c* (Å)	110.4, 110.4, 130.5	110.3, 110.3, 130.7	110.2, 110.2, 130.0
α, β, γ (°)	90.0, 90.0, 120.0	90.0, 90.0, 120.0	90.0, 90.0, 120.0
Resolution (Å)	47.82 – 1.50 (1.53-1.50)*	47.78 - 1.47 (1.49 - 1.47) *	47.74 – 1.39 (1.42 – 1.39)*
Mean I/sigma (I)	15.9 (1.1)	34.6 (1.3)	20.6 (0.9)
R_{merge}	0.229 (4.526)	0.065 (3.127)	0.139 (5.034)
$CC_{1/2}$	0.999 (0.455)	1.000 (0.563)	1.000 (0.389)
Completeness (%)	100.0 (99.3)	99.9 (98.1)	99.9 (98.6)
Redundancy	39.2 (37.4)	39.4 (29.9)	39.0 (36.2)
**Refinement**			
Resolution (Å)	47.82 – 1.50 (1.53-1.50)*	47.78 - 1.47 (1.49 - 1.47) *	47.74 – 1.39 (1.42 – 1.39)*
No. reflections	74984	79916	93128
R_{work} / R_{free}	0.182/0.201	0.160/0.194	0.154/0.172
No. atoms			
Protein	2450	2458	2475
Ligand	33	31	31
Solvent	134	137	204
Average *B*-factor			
Overall	?	28.00	23.29
Protein	21.57	28.22	22.47
Solvent	26.44	34.35	34.65
Ligand	14.13	18.53	14.13
R.m.s. deviations			
Bond lengths (Å)	0.012	0.023	0.012
Bond angles (º)	2.07	2.25	1.62
**Ramachandran Plot**			
Favored	98.39	99.03	98.38
Allowed	1.29	0.65	1.29
Not Allowed	0.32	0.32	0.32

*Single crystal was used for each dataset; *Values in parentheses are highest-resolution shell

Table S3: Data collection and Refinement Statistics

	**KMT9+compound 3b**	**KMT9+compound 5b**
**Data collection**		
Space group	$P6_122$	$P6_122$
Cell dimensions		
a, *b*, *c* (Å)	110.3, 110.3, 130.5	110.5, 110.5, 131.1
α, β, γ (°)	90.0, 90.0, 120.0	90.0, 90.0, 120.0
Resolution (Å)	47.76 – 1.39 (1.42 – 1.39)*	47.86 – 1.70 (1.73 – 1.70)*
Mean I/sigma (I)	28.7 (1.6)	30.2 (1.2)
R_{merge}	0.093 (3.126)	0.083 (3.855)
$CC_{1/2}$	1.000 (0.613)	1.000 (0.592)
Completeness (%)	99.9 (98.2)	99.8 (98.1)
Redundancy	39.0 (37.4)	39.6 (37.9)
**Refinement**		
Resolution (Å)	47.76 – 1.39 (1.42 – 1.39)*	47.86 – 1.70 (1.73 – 1.70)*
No. reflections	93562	52370
R_{work} / R_{free}	0.150/0.179	0.178/0.190
No. atoms		
Protein	2470	2434
Ligand	32	38
Solvent	197	111
Average *B*-factor		
Overall	24.18	35.35
Protein	23.55	35.17
Solvent	33.56	41.35
Ligand	15.53	29.37
R.m.s. deviations		
Bond lengths (Å)	0.014	0.015
Bond angles (º)	1.82	1.89
**Ramachandran Plot**		
Favored	99.03	98.38
Allowed	0.65	1.30
Not Allowed	0.32	0.32

*Single crystal was used for each dataset; *Values in parentheses are highest-resolution shell
